# APOC2 Promotes Clear Cell Renal Cell Carcinoma Progression via Activation of the JAK-STAT Signaling Pathway

**DOI:** 10.3390/cimb47110936

**Published:** 2025-11-11

**Authors:** Yongyang Yun, Xing Ji, Tianyu Wu, Yixiao Liu, Zheng Li, Zhoujie Sun, Peimin Zhou, Lei Yang, Wei Yu

**Affiliations:** 1 Department of Urology, Peking University First Hospital, No. 8, Xishiku Street, Xicheng District, Beijing 100034, China; yunyongyang@bjmu.edu.cn (Y.Y.);; 2 Institution of Urology, Peking University, No. 8, Xishiku Street, Xicheng District, Beijing 100034, China; 3 National Urological Cancer Center, No. 8, Xishiku Street, Xicheng District, Beijing 100034, China; 4 Beijing Key Laboratory of Urogenital Diseases (Male) Molecular Diagnosis and Treatment Center, No. 8, Xishiku Street, Xicheng District, Beijing 100034, China

**Keywords:** clear cell renal cell carcinoma (ccRCC), APOC2, JAK-STAT signaling pathway, STAT3, apoptosis, lipid metabolism, tumor progression

## Abstract

This study aimed to investigate the role and underlying mechanism of apolipoprotein C2 (APOC2) in the progression of clear cell renal cell carcinoma (ccRCC). Analysis of The Cancer Genome Atlas (TCGA) datasets, combined with validation in ccRCC cell lines, revealed that APOC2 was markedly upregulated in ccRCC tissues and cells and was associated with poor patient prognosis. Functional assays demonstrated that APOC2 knockdown significantly suppressed cell proliferation, colony formation, migration, and invasion, while promoting apoptosis. Mechanistic studies showed that silencing APOC2 reduced the phosphorylation levels of key components of the JAK-STAT signaling pathway, including Jak1/2 and STAT3, without affecting their total protein expression. Gene enrichment analysis further indicated the involvement of JAK-STAT signaling, and functional rescue experiments using the STAT3 agonist Colivelin partially reversed the decreased cell viability and increased apoptosis caused by APOC2 knockdown, confirming the pathway’s mediating role. Collectively, these findings suggest that APOC2 promotes ccRCC cell proliferation and inhibits apoptosis, at least in part, through activation of the JAK-STAT signaling pathway, highlighting APOC2 as a novel oncogenic regulator and potential therapeutic target, and providing new insight into the metabolic–inflammatory axis in ccRCC progression. Clinically, APOC2 may serve as a biomarker to identify ccRCC patients with hyperactivated JAK-STAT signaling and could potentially guide combination therapies involving JAK/STAT inhibitors or metabolic-targeted agents.

## 1. Introduction

Clear cell renal cell carcinoma (ccRCC) is the most common subtype of kidney cancer, accounting for approximately 75% of cases [1]. Its incidence has increased globally, particularly in developed countries, where it presents a significant clinical burden [2]. ccRCC is often asymptomatic in its early stages and tends to be diagnosed at advanced or metastatic stages [3,4]. While surgical resection is effective for localized tumors, advanced ccRCC frequently exhibits resistance to conventional therapies, and patient outcomes remain unsatisfactory despite recent advances in targeted and immune-based treatments [5,6].

Molecularly, ccRCC is characterized by frequent loss of chromosome 3p and inactivation of the VHL gene, leading to the stabilization of hypoxia-inducible factors (HIFs) and upregulation of pro-angiogenic pathways [7,8,9]. Additionally, mutations in chromatin remodeling genes such as PBRM1, SETD2, and BAP1 contribute to tumor progression and heterogeneity [10,11,12]. The clear cytoplasm typical of ccRCC reflects intracellular lipid and glycogen accumulation, suggesting metabolic reprogramming as a hallmark of the disease [13,14].

Among metabolic regulators, apolipoproteins have emerged as potential contributors to cancer biology [15,16,17]. APOC2, a key activator of lipoprotein lipase, plays an essential role in lipid metabolism [18,19]. While APOC2 overexpression has been reported in several cancers, including gastric malignancies [20,21], its role in ccRCC remains unclear. Recent findings suggest that APOC2 may influence tumor behavior by modulating oncogenic signaling pathways, including inflammation and lipid-driven proliferation [22].

The JAK/STAT signaling pathway plays a central role in regulating tumor cell proliferation, survival, immune evasion, and inflammatory responses [23,24,25].Aberrant activation of this pathway, particularly through STAT3, has been implicated in the pathogenesis and progression of ccRCC [26,27]. Recent studies have also linked metabolic stress and inflammatory lipoproteins to the activation of JAK/STAT signaling in various cancers [28,29]. Based on our enrichment analysis, we hypothesize that APOC2 may promote the malignant progression of ccRCC by activating the JAK/STAT signaling pathway, thereby enhancing tumor cell viability and growth.

Histone deacetylase inhibitors (HDACis) have recently gained attention as potential epigenetic therapies for clear cell renal cell carcinoma (ccRCC). A 2021 systematic review reported that HDACis achieved an overall response rate of about 26% and a one-year progression-free survival rate of 29% in patients with advanced ccRCC [30], suggesting that targeting epigenetic regulators such as HDACs may offer additional therapeutic benefits in this malignancy.

In this study, we investigated the functional role of APOC2 in ccRCC through a series of in vitro assays, including cell proliferation, migration, invasion, and apoptosis analyses. We further explored its regulatory impact on the JAK/STAT signaling pathway to uncover the underlying mechanisms driving its oncogenic potential. Our findings identify APOC2 as a novel oncogenic factor in ccRCC that promotes tumor progression, at least in part, via activation of the JAK/STAT pathway. These results provide new insights into the molecular functions of APOC2 in renal cancer and highlight its potential as a prognostic biomarker and therapeutic target.

## 2. Materials and Methods

### 2.1. Cell Culture and Reagents

All cell lines were obtained from the Cell Bank of the Chinese Academy of Sciences (Shanghai, China) or ATCC and stored in the departmental biobank. Cell identity was verified by short tandem repeat (STR) profiling (Microread Genetics, Beijing, China), and mycoplasma testing was routinely performed using the MycoAlert™ Mycoplasma Detection Kit (Lonza, Basel, Switzerland). Cells were cultured for fewer than 20 passages after thawing, and experiments were conducted using cells between passages 5–15. All cells were maintained in Dulbecco’s Modified Eagle’s Medium (DMEM, Gibco, Waltham, MA, USA) supplemented with 10% fetal bovine serum (FBS) and 1% penicillin–streptomycin. Cells were maintained at 37 °C in a humidified incubator with 5% CO_2_ (Heracell VIOS 160i, Thermo Fisher Scientific, Waltham, MA, USA). Cell confluence and morphology were observed under an inverted phase-contrast microscope (Leica DMi1, Leica Microsystems, Wetzlar, Germany).

### 2.2. siRNA Transfection

Small interfering RNA (siRNA) targeting APOC2 and a scrambled negative control were synthesized by Tianyi Huiyuan Life Science & Technology Co., Ltd. (Beijing, China). Cells in the logarithmic growth phase (786-O and OSRC-2) were seeded into 6-well plates (5 × 10^4^ cells/well) 24 h before transfection. Transfections were carried out using Lipofectamine^®^ RNAiMAX reagent (Invitrogen, Waltham, MA, USA) in Opti-MEM medium (Gibco, USA) according to the manufacturer’s protocol.

### 2.3. Western Blot Analysis

Proteins (30 µg per lane) were separated on 10% SDS-PAGE gels (Bio-Rad, Hercules, CA, USA) and transferred onto PVDF membranes (0.22 µm, Millipore, Burlington, MA, USA) using the Trans-Blot^®^ Turbo transfer system (Bio-Rad). Membranes were blocked with 5% non-fat milk in TBST for 1 h and incubated overnight at 4 °C with primary antibodies (1:1000; Cell Signaling Technology, Danvers, MA, USA) against APOC2, p-JAK1, JAK1, p-JAK2, JAK2, p-STAT3, STAT3, BCL-2, cleaved-Caspase 3, β-Actin, and GAPDH. After washing, HRP-conjugated secondary antibodies (1:5000; Jackson ImmunoResearch, West Grove, PA, USA) were applied for 1 h at room temperature. Protein bands were visualized using an enhanced chemiluminescence (ECL) kit (Pierce, Thermo Fisher Scientific, USA) and imaged on a ChemiDoc™ MP system (Bio-Rad). Band intensities were quantified using ImageJ software (version 1.54) (NIH, Bethesda, MD, USA). Full-length blots are provided in the Supplementary Information.

### 2.4. Transwell Migration and Invasion Assays

Cell migration and invasion were assessed using Transwell chambers with 8 μm pore polycarbonate membranes (Corning, Corning, NY, USA). For migration, 1 × 10^5^ cells were seeded into the upper chambers in serum-free medium. The lower chambers were filled with 10% FBS as a chemoattractant. For invasion assays, the upper chambers were pre-coated with Matrigel. After 48 h, cells on the lower membrane surface were fixed, stained with 0.1% crystal violet, and counted in five randomly selected fields.

### 2.5. Wound Healing Assay

Cells were plated in 6-well plates and cultured until nearly confluent. A uniform scratch was created with a sterile 200 μL pipette tip, and floating cells were removed with PBS. The remaining cells were maintained in serum-free medium. Wound closure was photographed at 0, 12, and 24 h using a Leica microscope. Migration rates were analyzed by comparing wound areas using ImageJ.

### 2.6. Cell Proliferation Assay (CCK-8)

To assess proliferation, cells were seeded in 96-well plates at a density of 5 × 10^2^ cells per well. At 24, 48, 72, and 96 h post-transfection, 10 μL of CCK-8 solution (Dojindo, Shanghai, China) was added to each well. After 2 h of incubation, absorbance at 450 nm was measured using a SpectraMax M2e microplate reader (Molecular Devices, San Jose, CA, USA).

### 2.7. Colony Formation Assay

Following transfection, 500 cells were seeded into each well of 6-well plates and cultured for 10–14 days. Colonies were fixed with 4% paraformaldehyde and stained with 0.1% crystal violet. Colonies consisting of more than 50 cells were counted under a microscope, and clonogenic efficiency was calculated.

### 2.8. Flow Cytometric Analysis of Apoptosis

Apoptotic cells were identified using an Annexin V-FITC/PI Apoptosis Detection Kit (BD Biosciences, Franklin Lakes, NJ, USA). After transfection, cells were harvested, washed with PBS, and stained with Annexin V-FITC and propidium iodide (PI) in binding buffer. After 15 min of incubation in the dark, apoptosis was quantified using flow cytometry (BD Accuri C6), and data were analyzed with FlowJo software (version 11).

### 2.9. Statistical Analysis

All experiments were independently repeated at least three times. Data are presented as the mean ± standard deviation (SD). Statistical significance was determined using GraphPad Prism 9 software. Student’s *t*-test was applied for two-group comparisons, and one-way ANOVA was used for multi-group analysis. A *p*-value < 0.05 was considered statistically significant.

## 3. Results

### 3.1. The Expression of APOC2 Was Upregulated in ccRCC Tumor Tissues and Cell Lines and Associated with Poor Prognosis

**Figure 1:**
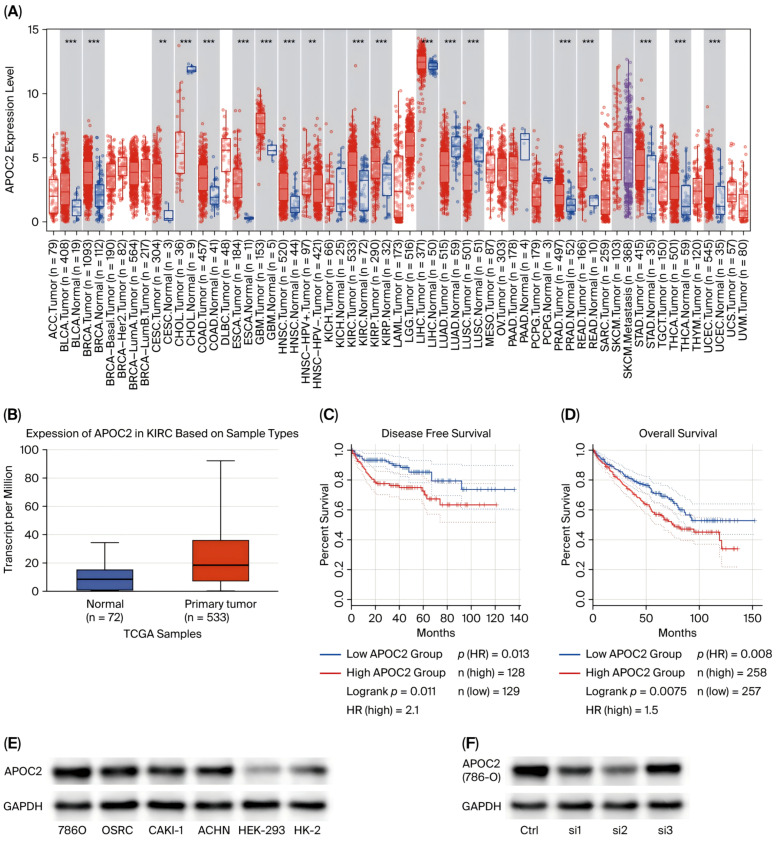
**APOC2 is upregulated in ccRCC and associated with poor prognosis.** (**A**) Pan-cancer analysis of APOC2 expression across multiple tumor types in the TCGA database. (**B**) Unpaired comparison of APOC2 mRNA expression between 533 ccRCC tumor tissues and 72 normal kidney tissues. (**C**,**D**) Kaplan–Meier survival analysis showing that high APOC2 expression is significantly associated with worse overall survival (OS) and disease-free survival (DFS) in ccRCC patients. (**E**) Western blot analysis confirming elevated APOC2 protein levels in four ccRCC cell lines (CAKI-1, OSRC-2, 786-O, and ACHN) compared with normal renal epithelial cells (HEK-293 and HK2). (**F**) Verification of APOC2 knockdown efficiency by siRNA in 786-O and OSRC-2 cells using Western blot. ** *p* < 0.01, *** *p* < 0.001.

Analysis of data from the TCGA database revealed that APOC2 is significantly upregulated in multiple tumor types, including clear cell renal cell carcinoma (ccRCC), bladder urothelial carcinoma, colorectal cancer, breast cancer, and esophageal carcinoma (Figure 1A). A subsequent unpaired analysis of 533 ccRCC tumor samples and 72 normal kidney tissues further confirmed that APOC2 mRNA levels were markedly higher in ccRCC tissues compared to normal counterparts (Figure 1B). The results presented here are, in whole or in part, based upon data generated by the TCGA Research Network: https://www.cancer.gov/tcga, accessed on 28 October 2025.

To validate these findings at the cellular level, we examined APOC2 expression in normal renal epithelial cell lines (HEK-293 and HK2) and four ccRCC cell lines (CAKI-1, OSRC-2, 786-O, and ACHN). Western blot analysis demonstrated that APOC2 protein expression was significantly elevated in all ccRCC cell lines relative to normal controls (Figure 1E).

Furthermore, to assess the clinical relevance of APOC2 expression, survival analysis using the Kaplan–Meier Plotter based on TCGA data was conducted. The results showed that patients with high APOC2 expression had markedly shorter overall survival (OS) and disease-free survival (DFS) compared with those with low expression, suggesting that elevated APOC2 expression may be associated with poor prognosis in ccRCC (Figure 1C,D).

### 3.2. APOC2 Knockdown Suppresses Tumorigenic Behaviors in ccRCC Cell Lines

**Figure 2:**
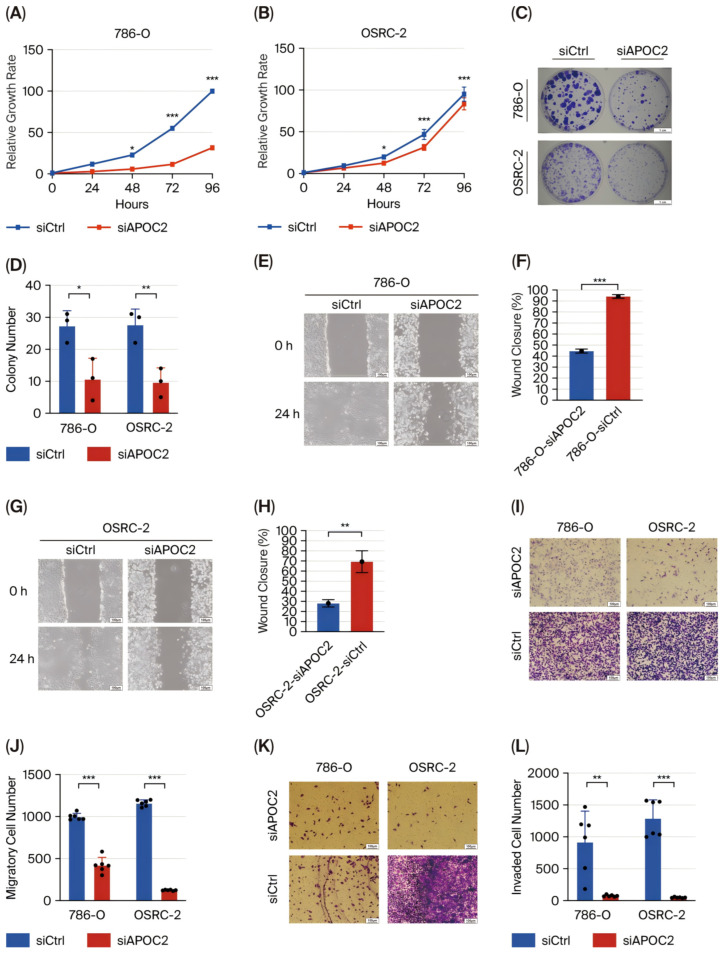

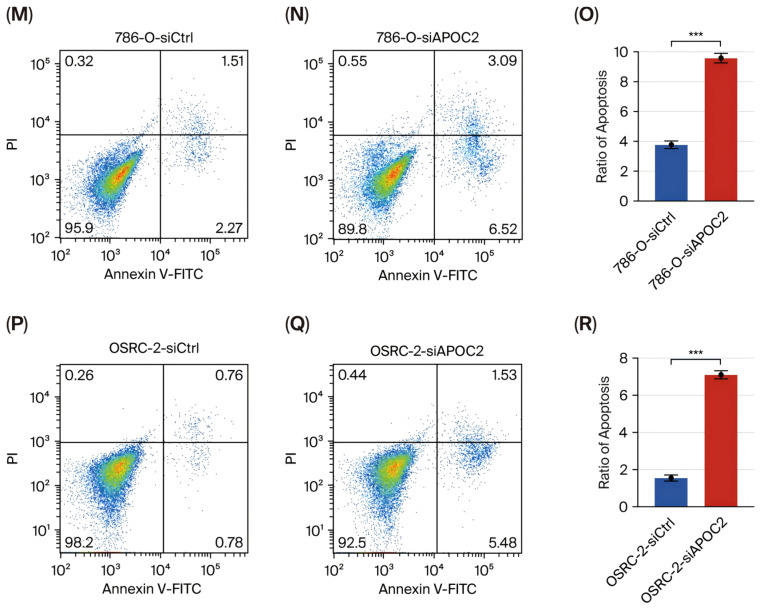
**APOC2 knockdown suppresses proliferation, motility, and invasion, and induces apoptosis in ccRCC cells.** (**A**,**B**) CCK-8 assay showing significantly reduced cell proliferation in APOC2-knockdown cell lines (**C**,**D**) Colony formation assay demonstrating a marked reduction in the number and size of colonies upon APOC2 silencing. (**E**–**H**) Wound healing assays reveal impaired wound closure ability in APOC2-knockdown cells (**I**–**L**) Transwell assays showing significantly reduced cell migration (**I**,**J**) and invasion (**K**,**L**) after APOC2 knockdown in both cell lines. (**M**–**R**) Flow cytometric analysis of apoptosis shows increased apoptotic cell populations in APOC2-silenced cells. * *p* < 0.05, ** *p* < 0.01, *** *p* < 0.001.

**Figure 3:**
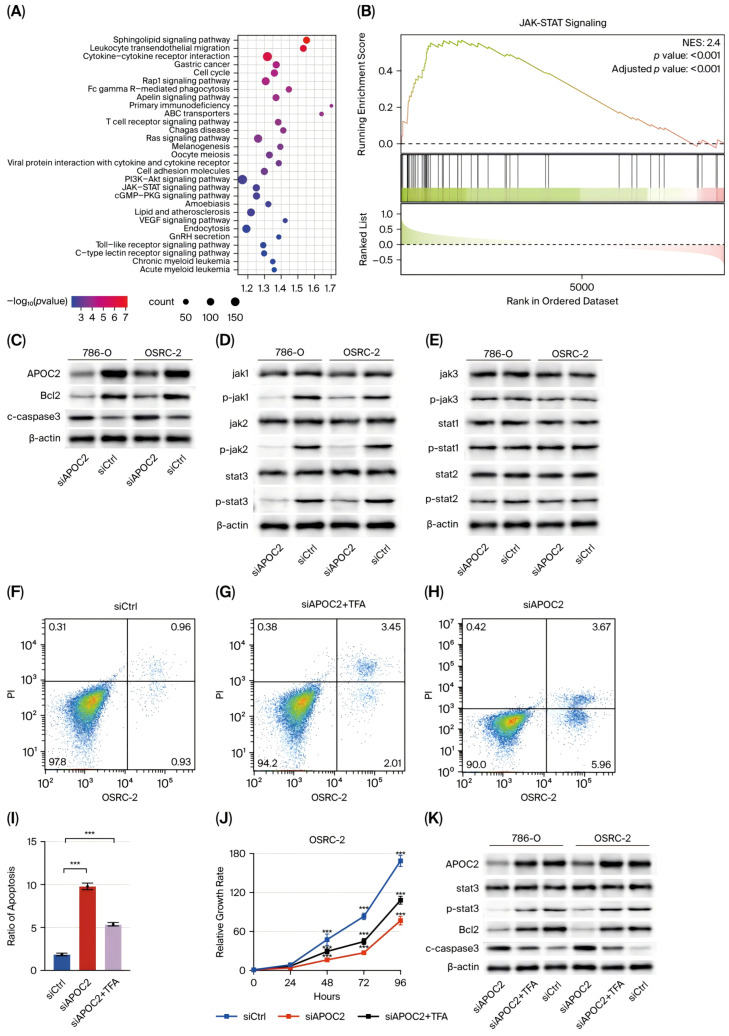
**APOC2 promotes ccRCC progression via activation of the JAK-STAT signaling pathway.** (**A**) KEGG pathway enrichment analysis based on APOC2 expression levels. (**B**) GSEA indicates a strong positive correlation between APOC2 expression and activation of the JAK-STAT signaling pathway. (**C**) Western blot showing that APOC2 knockdown decreases BCL-2 and increases cleaved-Caspase 3 levels. (**D**,**E**) Western blot analysis showing that phosphorylation levels of JAK1, JAK2, and STAT3 are markedly reduced after APOC2 silencing, while total protein levels remain unchanged. (**F**–**I**) Flow cytometric analysis showing that Colivelin treatment partially reverses APOC2-knockdown-induced apoptosis. (**J**) CCK-8 assay showing that the STAT3 agonist Colivelin TFA significantly restores cell viability in APOC2-knockdown cells. (**K**) Western blot confirming that Colivelin rescues the phosphorylation of STAT3 and reverses the expression changes of BCL-2 and cleaved-Caspase 3 in APOC2-knockdown cells. *** *p* < 0.001.

To elucidate the regulatory role of APOC2 in the biological behavior of clear cell renal cell carcinoma (ccRCC) cells, we established APOC2 knockdown models in 786-O and OSRC-2 cell lines. Western blot analysis confirmed a significant reduction in APOC2 protein levels following siRNA transfection, indicating effective knockdown (Figure 1F). Subsequently, cell proliferation was continuously monitored using the CCK-8 assay at multiple time points. Cells with APOC2 knockdown exhibited consistently reduced proliferation over a 96 h period, with statistically significant differences compared with control cells (Figure 2A,B). Consistently, colony formation assays demonstrated that APOC2 silencing markedly reduced the number and size of colonies (Figure 2C,D), suggesting a promotive role of APOC2 in ccRCC cell proliferation. To further investigate its impact on cell motility, wound healing assays were performed. APOC2 knockdown significantly decreased wound closure ability in both cell lines (Figure 2E–H), while Transwell assays revealed that silencing APOC2 also inhibited cell migration and invasion through the membrane (Figure 2I–L). These results collectively suggest that APOC2 may promote ccRCC progression by enhancing cell motility and invasive capacity. Moreover, flow cytometric analysis showed a significant increase in apoptosis rates in the APOC2-silenced groups (Figure 2M–R), further supporting the notion that APOC2 may act as a tumor promoter gene. This anti-apoptotic effect may be associated with alterations in cell cycle regulation or suppression of apoptotic signaling. Additionally, Western blot analysis revealed that apoptosis-related proteins were significantly altered following APOC2 knockdown. Specifically, the anti-apoptotic protein BCL-2 was downregulated, while the expression of cleaved-Caspase 3 was upregulated (Figure 3C), indicating an enhanced apoptotic response upon APOC2 silencing. These results further support the notion that APOC2 inhibits apoptosis in ccRCC cells and may function as a tumor promoter through anti-apoptotic mechanisms.

In summary, APOC2 exhibits potential tumor-promoting properties in ccRCC. Its downregulation suppressed cell proliferation, migration, invasion, and enhanced apoptosis, suggesting that APOC2 may play an oncogenic role in driving ccRCC progression.

### 3.3. JAK-STAT Pathway Mediates APOC2-Induced Oncogenic Effects in ccRCC

To further elucidate the oncogenic mechanism of APOC2 in clear cell renal cell carcinoma (ccRCC), KEGG pathway enrichment analysis was performed based on APOC2 expression levels. The results indicated that high APOC2 expression was significantly associated with several key pathways, including cytokine–cytokine receptor interaction, cell cycle, PI3K-Akt signaling pathway, JAK-STAT signaling pathway, and endocytosis (Figure 3A). In addition, GSEA revealed a strong positive correlation between APOC2 expression and activation of the JAK-STAT signaling pathway (Figure 3B). Given the critical role of this pathway in regulating cell proliferation, apoptosis, migration, and metabolism, we hypothesized that APOC2 may promote the malignant progression of ccRCC by activating the JAK-STAT pathway.

To test this hypothesis, we knocked down APOC2 in 786-O and OSRC-2 cells and evaluated the expression of pathway-related proteins. Western blot analysis showed that levels of phosphorylated Jak1, Jak2, and Stat3 were markedly reduced, while total Jak1, Jak2, and Stat3 levels remained unchanged (Figure 3D,E), suggesting that APOC2 may regulate the activation state of the pathway rather than its expression.

To further validate this regulatory relationship, a functional rescue experiment was conducted using the Stat3 agonist Colivelin TFA. In APOC2-knockdown cells, treatment with Colivelin significantly restored cell viability and reduced apoptosis levels, as measured by the CCK-8 assay and flow cytometry, respectively (Figure 3F–J). Furthermore, Western blot analysis was performed to assess the restoration of JAK-STAT signaling and apoptosis-related proteins upon Colivelin treatment. In APOC2-knockdown cells, Colivelin effectively rescued the phosphorylation levels of STAT3 without altering total STAT3 expression, confirming reactivation of the pathway. In addition, the expression of the anti-apoptotic protein BCL-2 was upregulated, while cleaved-Caspase 3 levels were downregulated (Figure 3K), suggesting that Colivelin-mediated activation of the JAK-STAT pathway attenuated the pro-apoptotic effects induced by APOC2 silencing.

Collectively, these findings suggest that APOC2 promotes ccRCC cell proliferation and suppresses apoptosis, at least in part, through activation of the JAK-STAT signaling pathway. Importantly, the biological effects induced by APOC2 knockdown, including reduced cell viability and increased apoptosis, can be partially reversed by pharmacological activation of this pathway. These results underscore the pivotal role of JAK-STAT signaling in mediating APOC2-driven tumor progression in ccRCC.

## 4. Discussion

Clear cell renal cell carcinoma (ccRCC) is characterized by marked metabolic reprogramming, immune evasion, and aberrant activation of oncogenic signaling pathways [31,32]. Among the various dysregulated pathways, the Janus kinase/signal transducer and activator of transcription (JAK-STAT) signaling cascade has been recognized as a critical mediator of tumor progression, particularly through STAT3, which promotes proliferation, angiogenesis, immune suppression, and epithelial–mesenchymal transition (EMT) [33,34]. Aberrant STAT3 activation has been associated with advanced disease stage, resistance to targeted therapy, and poor prognosis in ccRCC patients [35]. STAT3 also regulates transcription of genes involved in anti-apoptosis (BCL2, MCL1), cell cycle progression (Cyclin D1, MYC), and metastasis (MMP2, MMP9), making it a central hub in renal tumorigenesis [36].

In our study, APOC2 was identified as a potential upstream regulator of JAK-STAT signaling in ccRCC. APOC2, a component of very low-density lipoproteins (VLDL), is traditionally known for activating lipoprotein lipase and facilitating triglyceride hydrolysis [18]. However, accumulating evidence has revealed its role in tumor biology, particularly in lipid metabolism-dependent cancers. APOC2 has been reported to promote tumor cell proliferation and invasion in gastric and hematological malignancies [20,37]. Its overexpression is often associated with hypertriglyceridemia in cancer patients, a phenotype linked to immune suppression and tumor growth. Moreover, APOC2 expression correlates with poor prognosis in multiple tumor types, underscoring its clinical relevance.

The link between lipid metabolism and JAK-STAT signaling is increasingly appreciated. Lipid metabolism-related proteins, including apolipoproteins and fatty acid transporters, have been shown to modulate STAT3 activation through both direct receptor interactions and indirect metabolic rewiring [38]. APOC2 may function in a similar manner, possibly via interaction with lipid transport receptors or cytokine receptors in the tumor microenvironment [22,39]. Additionally, APOC2 might induce proinflammatory cytokines such as IL-6 or IL-10, which are well-established activators of the JAK2/STAT3 axis [40]. This hypothesis is supported by previous findings that lipoprotein-rich environments can induce STAT3 activation via IL-6 family cytokines.

Functionally, our data showed that APOC2 knockdown inhibited ccRCC cell proliferation, migration, and colony formation, while increasing apoptosis. These phenotypes mirror those observed following STAT3 inhibition in renal and other solid tumors [41,42]. Furthermore, APOC2 silencing led to downregulation of p-JAK1, p-JAK2, and p-STAT3 without affecting total STAT3 levels, suggesting that APOC2 modulates pathway activation rather than gene expression. Treatment with an STAT3-specific activator partially reversed the tumor-suppressive effects of APOC2 knockdown, supporting the hypothesis that JAK2/STAT3 is a key downstream effector of APOC2.

Epigenetic therapy represents a promising approach for ccRCC. A phase I/II trial combining the HDAC inhibitor vorinostat with bevacizumab achieved an 18% objective response rate and a 6-month PFS of about 48% in metastatic ccRCC [43]. HDAC inhibitors also synergize with tyrosine kinase inhibitors to suppress RCC cell growth [44]. Beyond cytotoxicity, they reduce IL-10 secretion via STAT3 inhibition in cutaneous T-cell lymphoma [45] and, in renal cancer models, decrease myeloid-derived suppressor cells while enhancing PD-1 blockade efficacy [46]. These findings highlight the dual antitumor and immunomodulatory potential of HDAC inhibition in ccRCC.

Recent studies have also demonstrated that STAT3 activation is involved in shaping the tumor immune microenvironment, particularly by promoting the expression of PD-L1, reducing NK cell infiltration, and polarizing macrophages toward the M2 phenotype [47,48]. APOC2 may therefore contribute to immune escape in ccRCC by reinforcing STAT3-driven immunosuppression, although further studies are needed to explore this hypothesis. Single-cell RNA sequencing and spatial transcriptomics could help delineate APOC2 expression in different cellular compartments of the tumor and its impact on immune crosstalk.

In addition, dietary and metabolic factors may influence HDAC activity and downstream signaling in renal cancer. As APOC2 is closely associated with lipid metabolism, variations in dietary lipid intake or metabolic state could potentially modulate the APOC2–JAK/STAT axis and affect tumor progression. Although this relationship remains to be fully elucidated, integrating metabolic and epigenetic regulation may offer new perspectives for therapeutic intervention in ccRCC.

In summary, our findings reveal a previously unrecognized link between APOC2 and JAK-STAT signaling in ccRCC. Given the widespread involvement of both APOC2 and STAT3 in metabolic and inflammatory pathways, targeting this axis may offer novel therapeutic opportunities. Moreover, APOC2 may serve as a prognostic biomarker and a metabolic vulnerability in renal cancer. Future work should focus on defining the precise molecular intermediates connecting APOC2 to STAT3 activation, and whether this mechanism operates in other lipid-rich malignancies.

## 5. Conclusions

Our study identifies APOC2 as a novel oncogenic regulator in clear cell renal cell carcinoma. We demonstrate that APOC2 is markedly upregulated in tumor tissues and cell lines and correlates with unfavorable prognosis. Functionally, APOC2 promotes cell proliferation, migration, and invasion while inhibiting apoptosis. Mechanistically, APOC2 activates the JAK-STAT signaling pathway, as evidenced by reduced phosphorylation of JAK1/2 and STAT3 upon APOC2 silencing and partial restoration of these effects by the STAT3 agonist Colivelin. These findings establish a mechanistic link between lipid metabolism and inflammatory signaling in renal cancer, highlighting APOC2 as a promising prognostic biomarker and therapeutic target. Further studies will explore APOC2-targeted strategies and its clinical utility in the personalized treatment of ccRCC.

## Data Availability

Publicly available datasets were analyzed in this study. These data can be found in The Cancer Genome Atlas (TCGA, https://portal.gdc.cancer.gov/, accessed on 28 October 2025). The original contributions presented in this study are included in the article. Further inquiries can be directed to the corresponding author.

## Supplementary Materials

The following supporting information can be downloaded at: https://www.mdpi.com/article/10.3390/cimb47110936/s1.
