# Supplementary figures and images for: APOC2 Promotes Clear Cell Renal Cell Carcinoma Progression via Activation of the JAK-STAT Signaling Pathway (part 2 of 2)
Source: Curr Issues Mol Biol. 2025 Nov 11;47(11):936. doi: 10.3390/cimb47110936 (PMC12651258; doi:10.3390/cimb47110936)

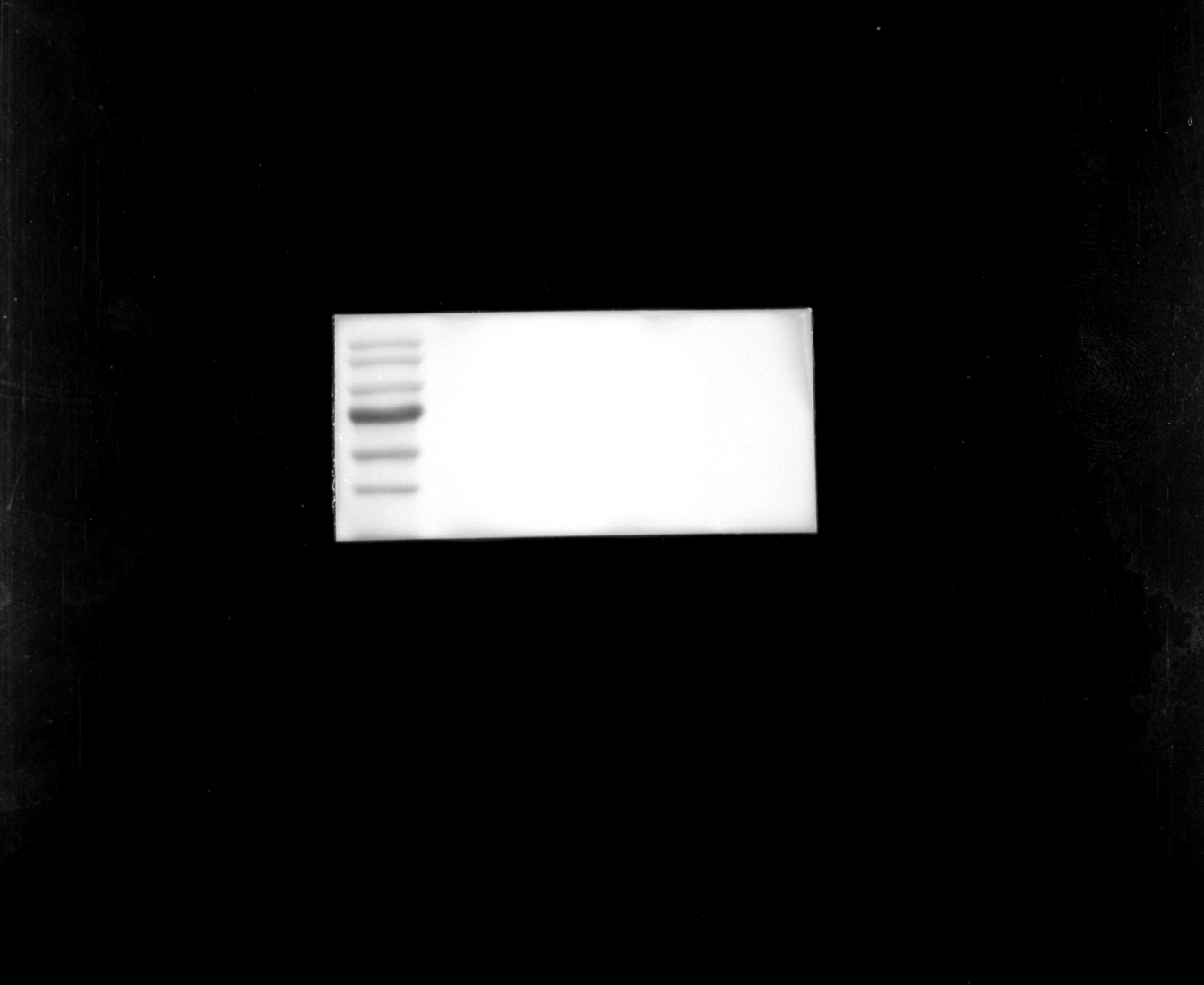

Supplement: Supplementary file 1 [file cimb-47-00936-s001.zip › cimb-3956315-supplementary/APOC2_ccRCC_RawWB_FullMembranes/cropped display images/4/Fig 3C β-Actin/0.Tif]

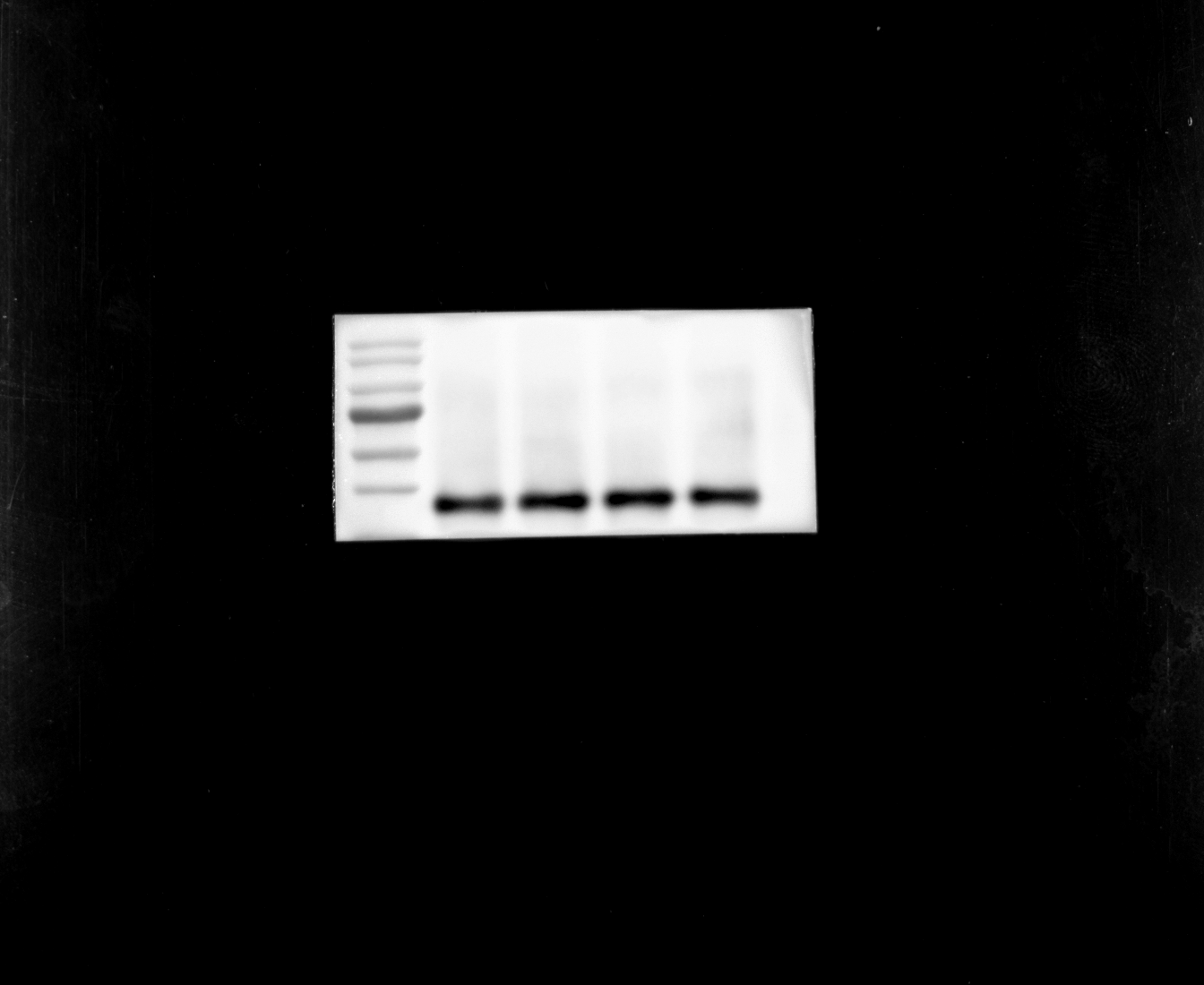

Supplement: Supplementary file 1 [file cimb-47-00936-s001.zip › cimb-3956315-supplementary/APOC2_ccRCC_RawWB_FullMembranes/cropped display images/4/Fig 3C β-Actin/1.Tif]

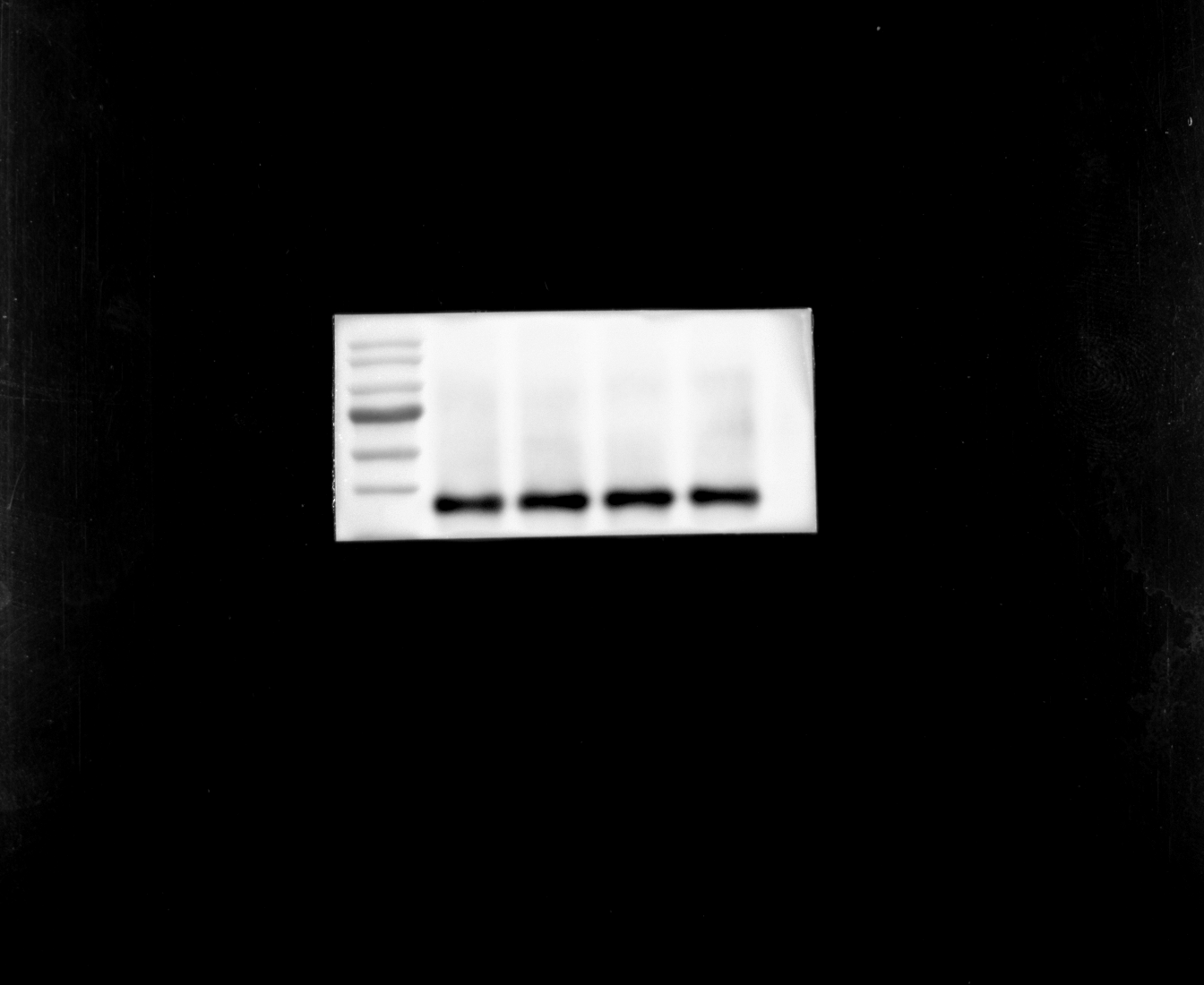

Supplement: Supplementary file 1 [file cimb-47-00936-s001.zip › cimb-3956315-supplementary/APOC2_ccRCC_RawWB_FullMembranes/cropped display images/4/Fig 3C β-Actin/2.Tif]

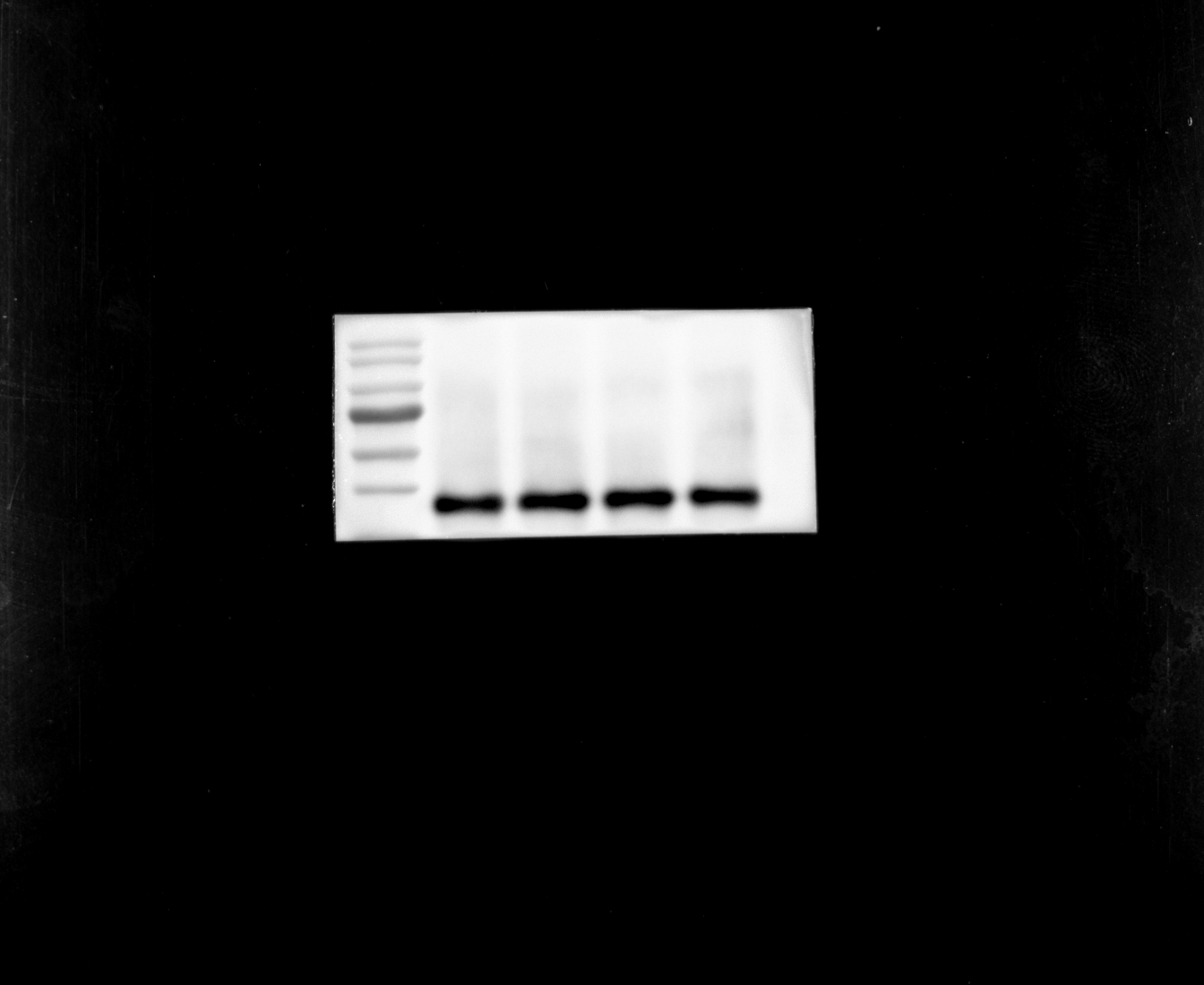

Supplement: Supplementary file 1 [file cimb-47-00936-s001.zip › cimb-3956315-supplementary/APOC2_ccRCC_RawWB_FullMembranes/cropped display images/4/Fig 3C β-Actin/3.Tif]

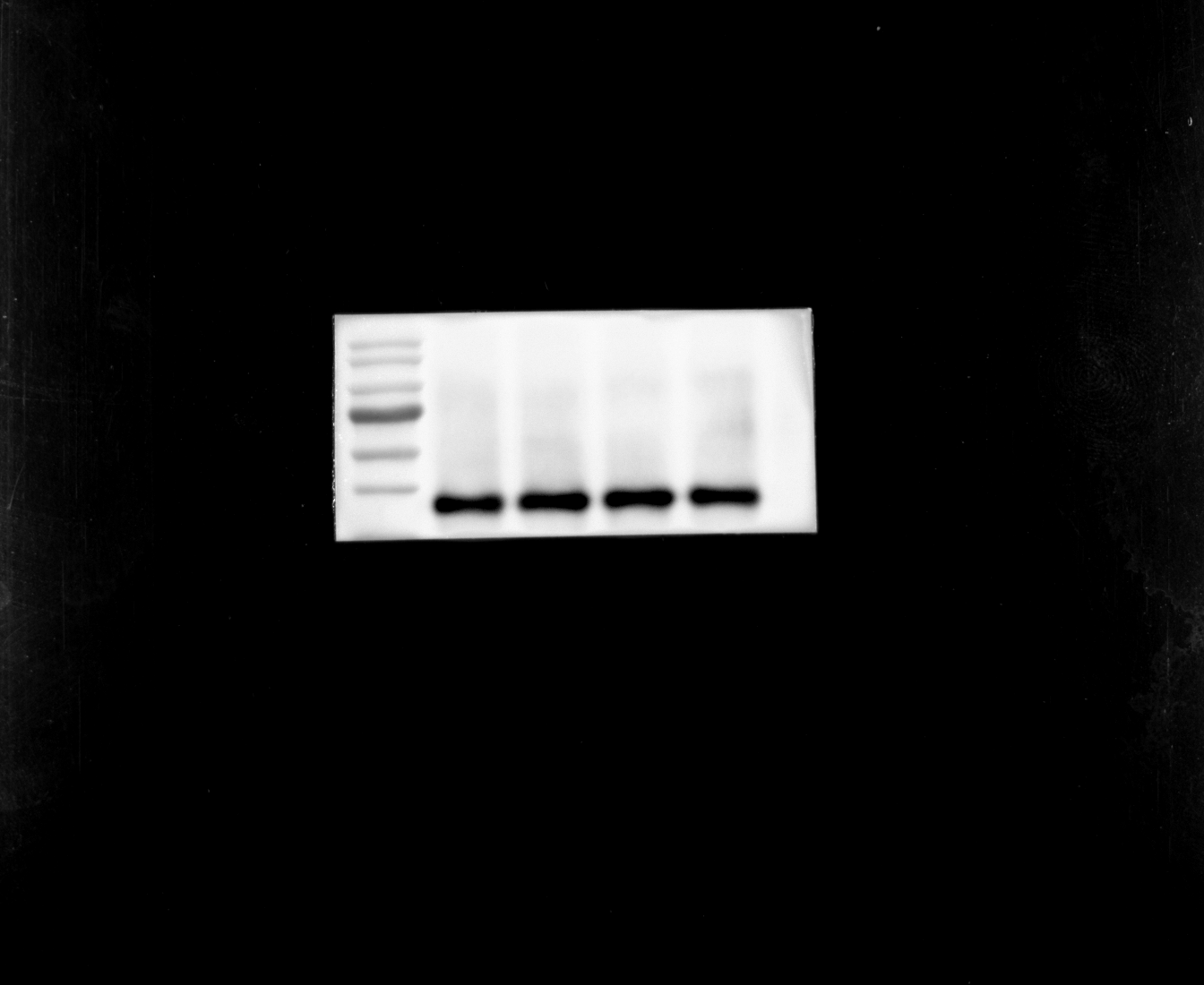

Supplement: Supplementary file 1 [file cimb-47-00936-s001.zip › cimb-3956315-supplementary/APOC2_ccRCC_RawWB_FullMembranes/cropped display images/4/Fig 3C β-Actin/4.Tif]

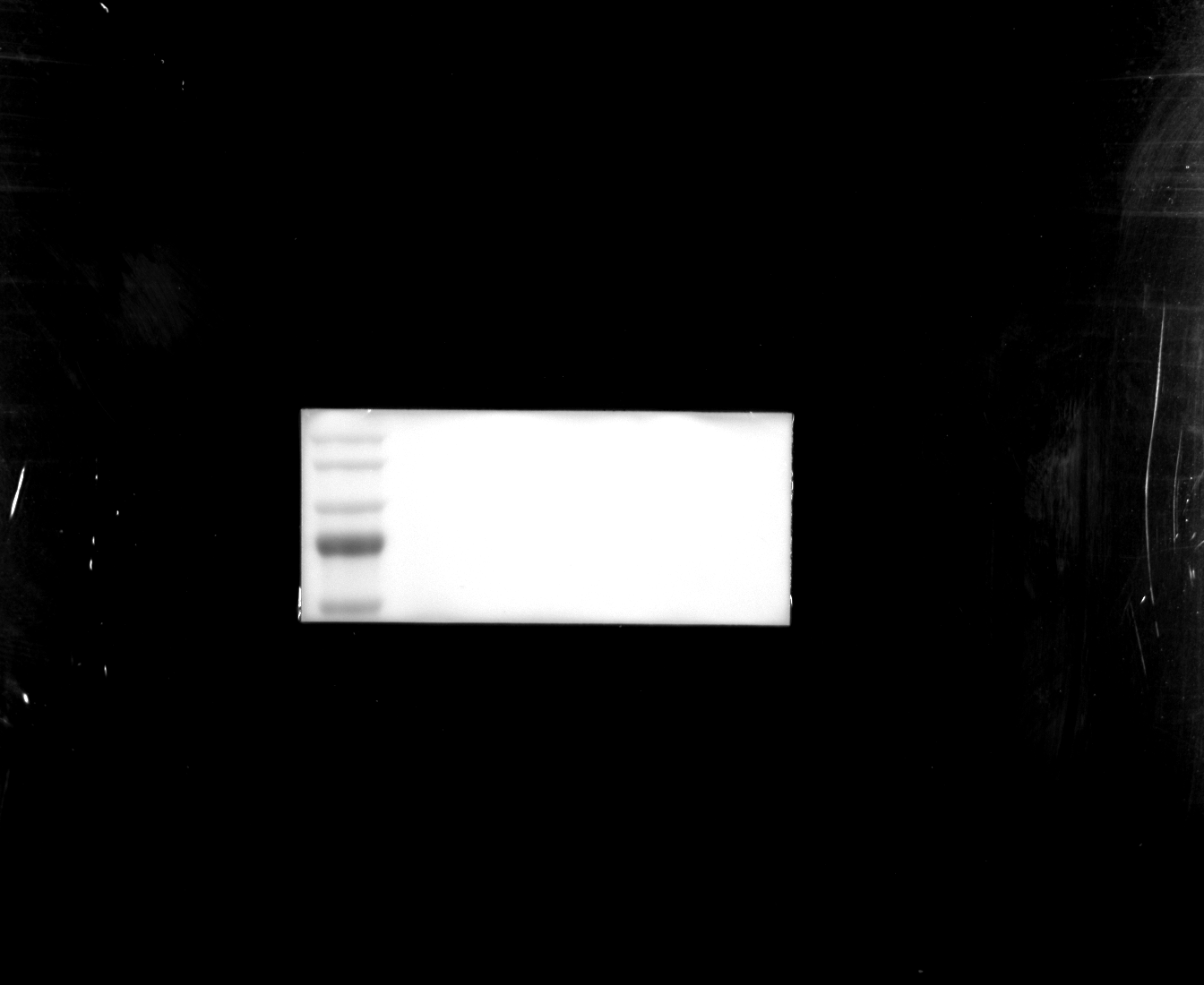

Supplement: Supplementary file 1 [file cimb-47-00936-s001.zip › cimb-3956315-supplementary/APOC2_ccRCC_RawWB_FullMembranes/cropped display images/5/Fig 3D jak1/0.Tif]

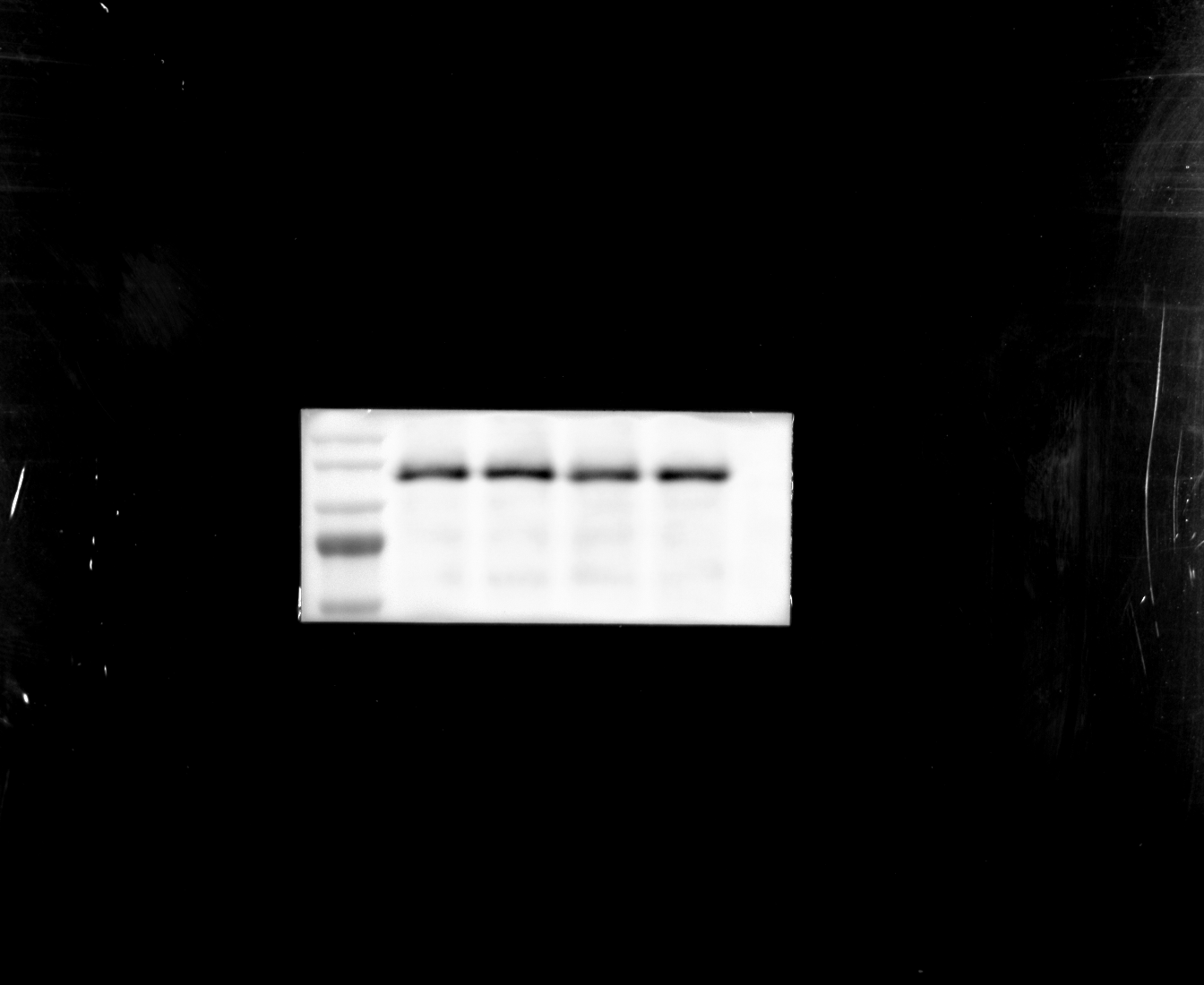

Supplement: Supplementary file 1 [file cimb-47-00936-s001.zip › cimb-3956315-supplementary/APOC2_ccRCC_RawWB_FullMembranes/cropped display images/5/Fig 3D jak1/1.Tif]

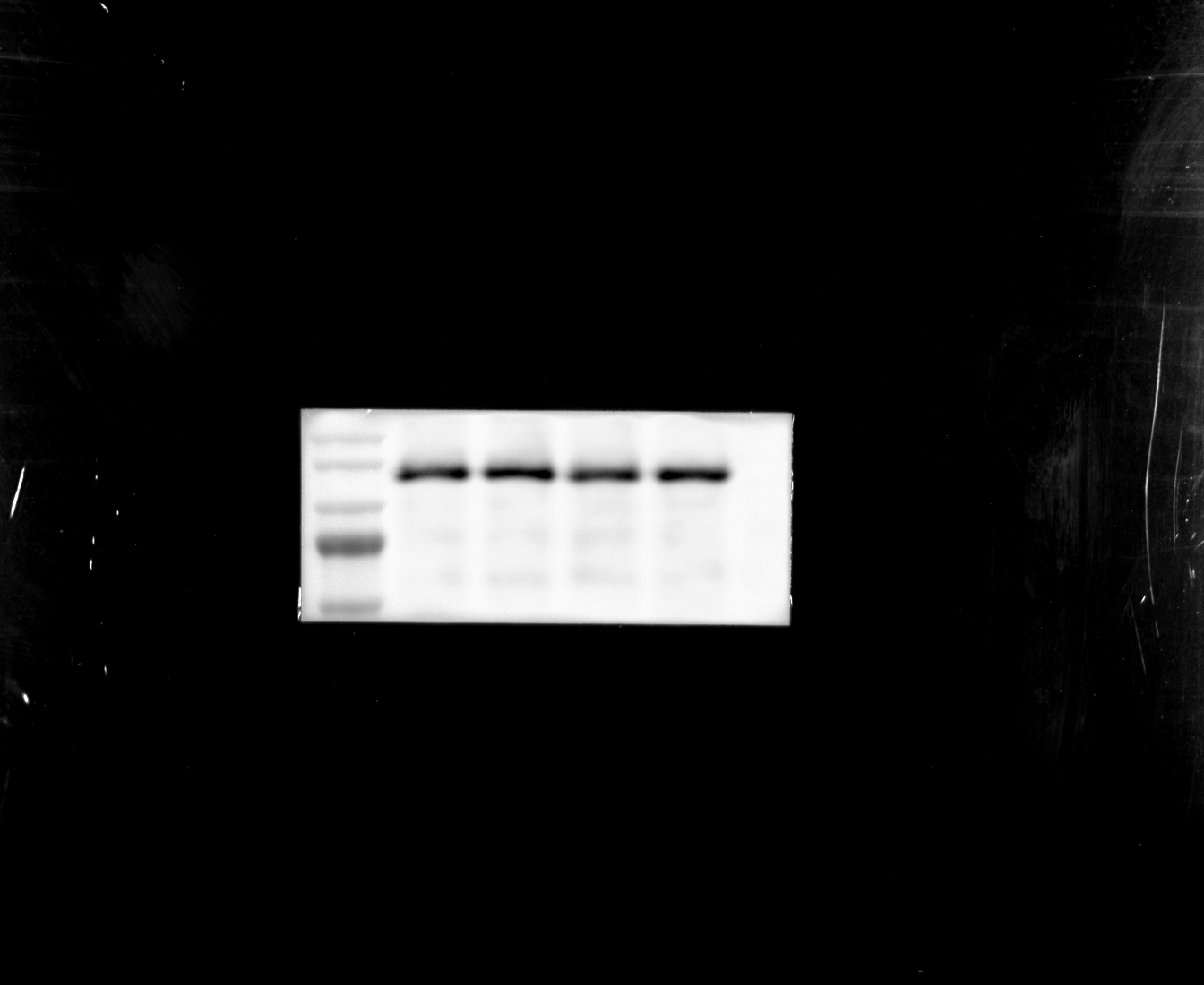

Supplement: Supplementary file 1 [file cimb-47-00936-s001.zip › cimb-3956315-supplementary/APOC2_ccRCC_RawWB_FullMembranes/cropped display images/5/Fig 3D jak1/2.Tif]

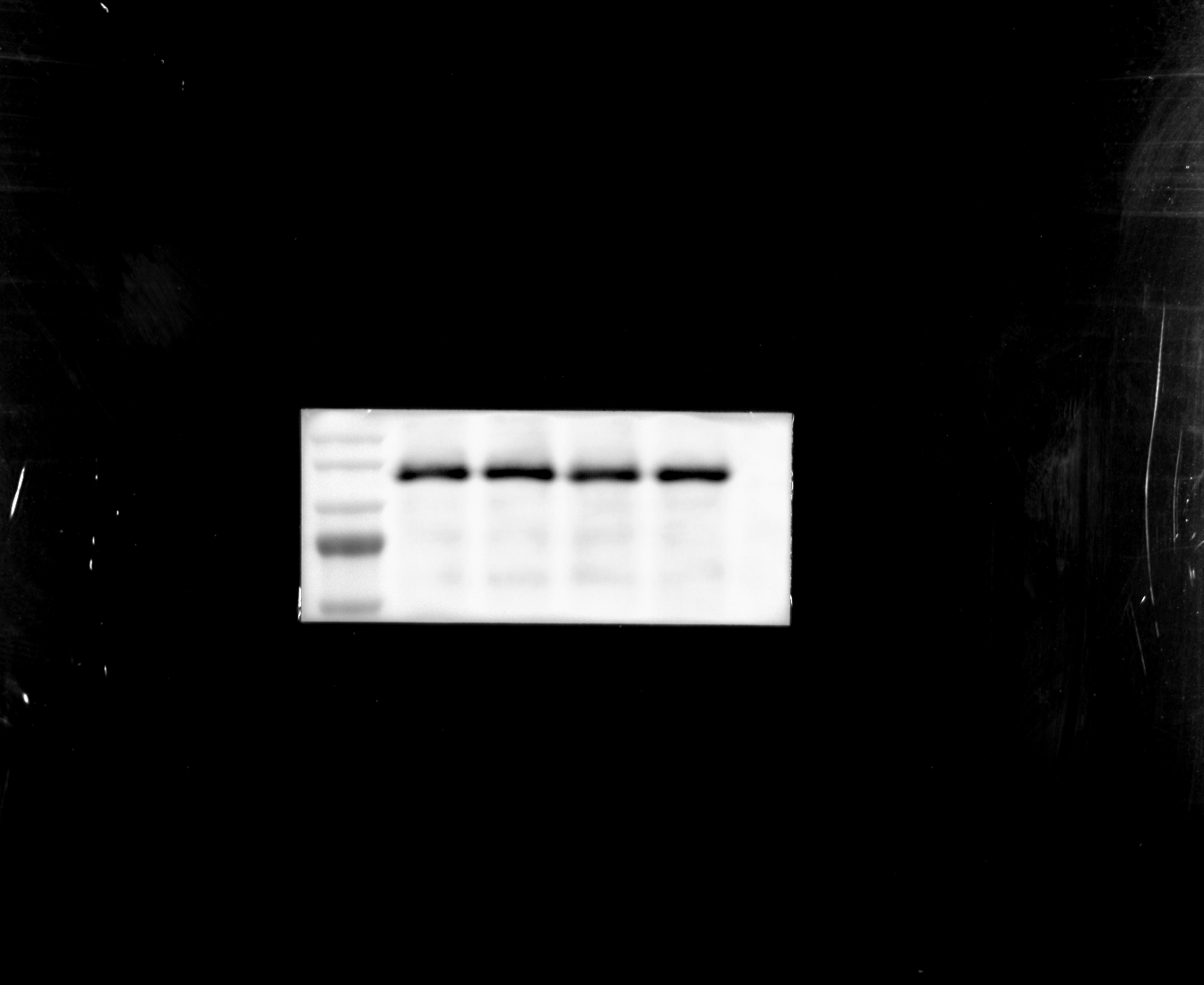

Supplement: Supplementary file 1 [file cimb-47-00936-s001.zip › cimb-3956315-supplementary/APOC2_ccRCC_RawWB_FullMembranes/cropped display images/5/Fig 3D jak1/3.Tif]

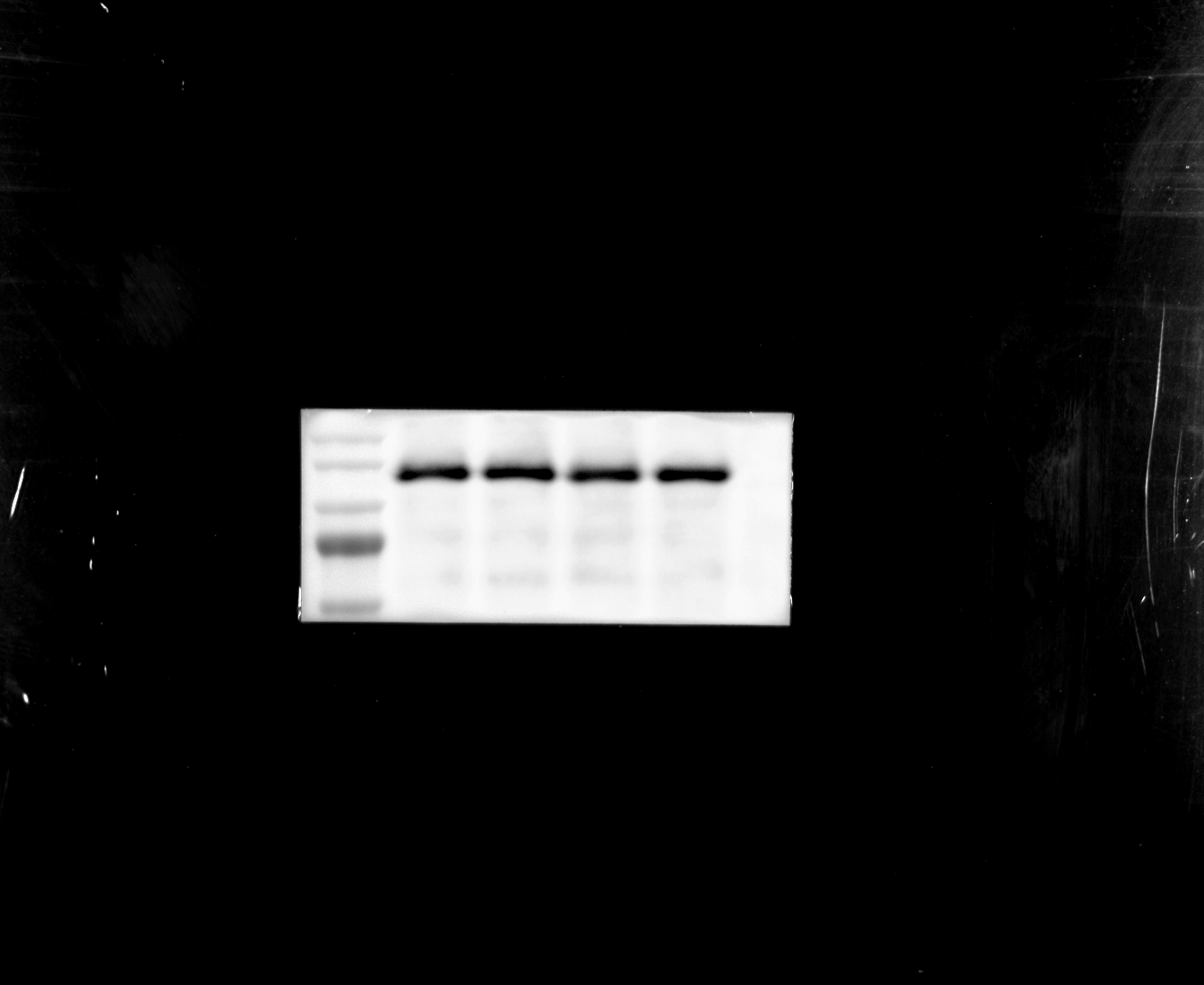

Supplement: Supplementary file 1 [file cimb-47-00936-s001.zip › cimb-3956315-supplementary/APOC2_ccRCC_RawWB_FullMembranes/cropped display images/5/Fig 3D jak1/4.Tif]

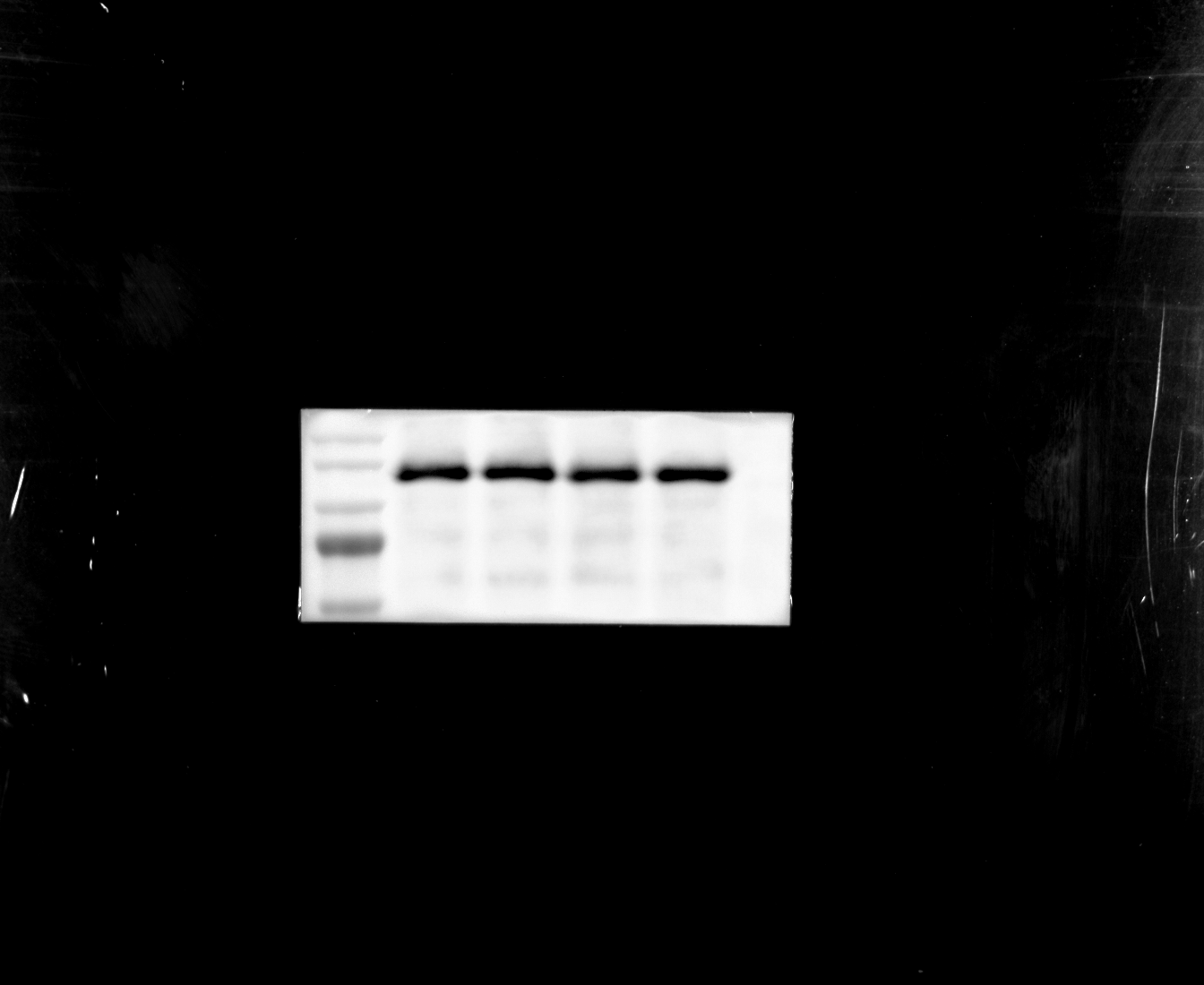

Supplement: Supplementary file 1 [file cimb-47-00936-s001.zip › cimb-3956315-supplementary/APOC2_ccRCC_RawWB_FullMembranes/cropped display images/5/Fig 3D jak1/5.Tif]

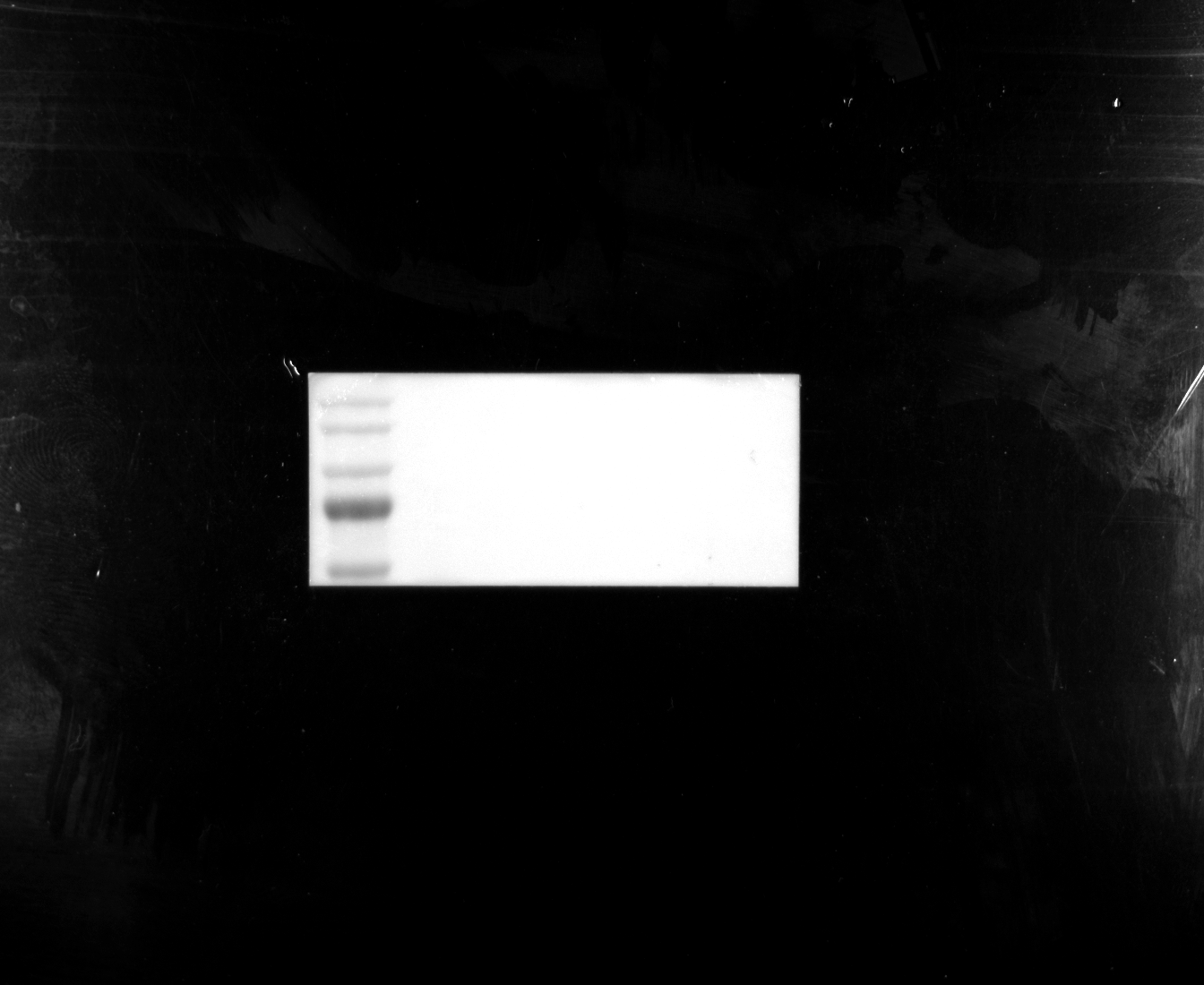

Supplement: Supplementary file 1 [file cimb-47-00936-s001.zip › cimb-3956315-supplementary/APOC2_ccRCC_RawWB_FullMembranes/cropped display images/5/Fig 3D p-jak1/0.Tif]

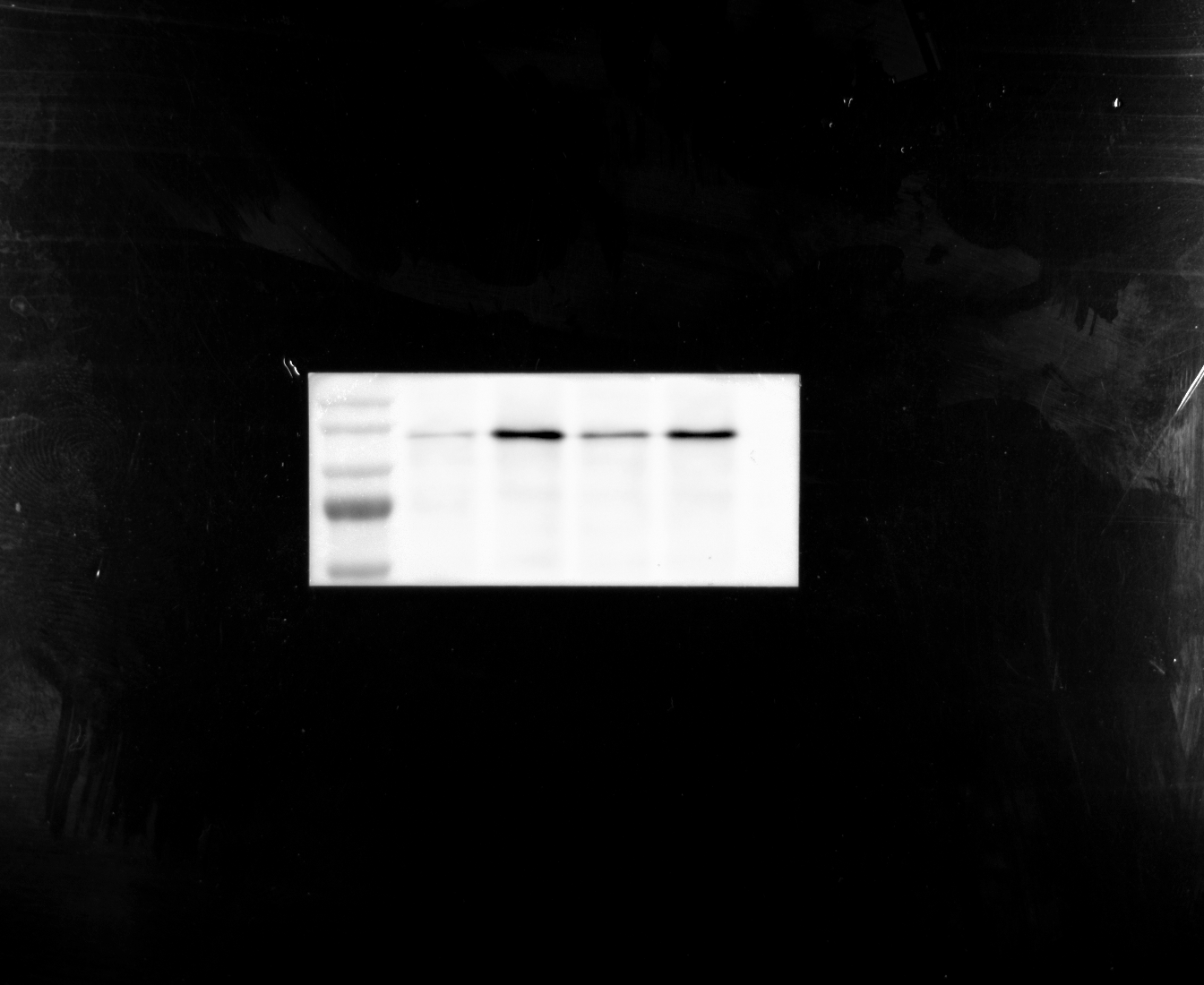

Supplement: Supplementary file 1 [file cimb-47-00936-s001.zip › cimb-3956315-supplementary/APOC2_ccRCC_RawWB_FullMembranes/cropped display images/5/Fig 3D p-jak1/1.Tif]

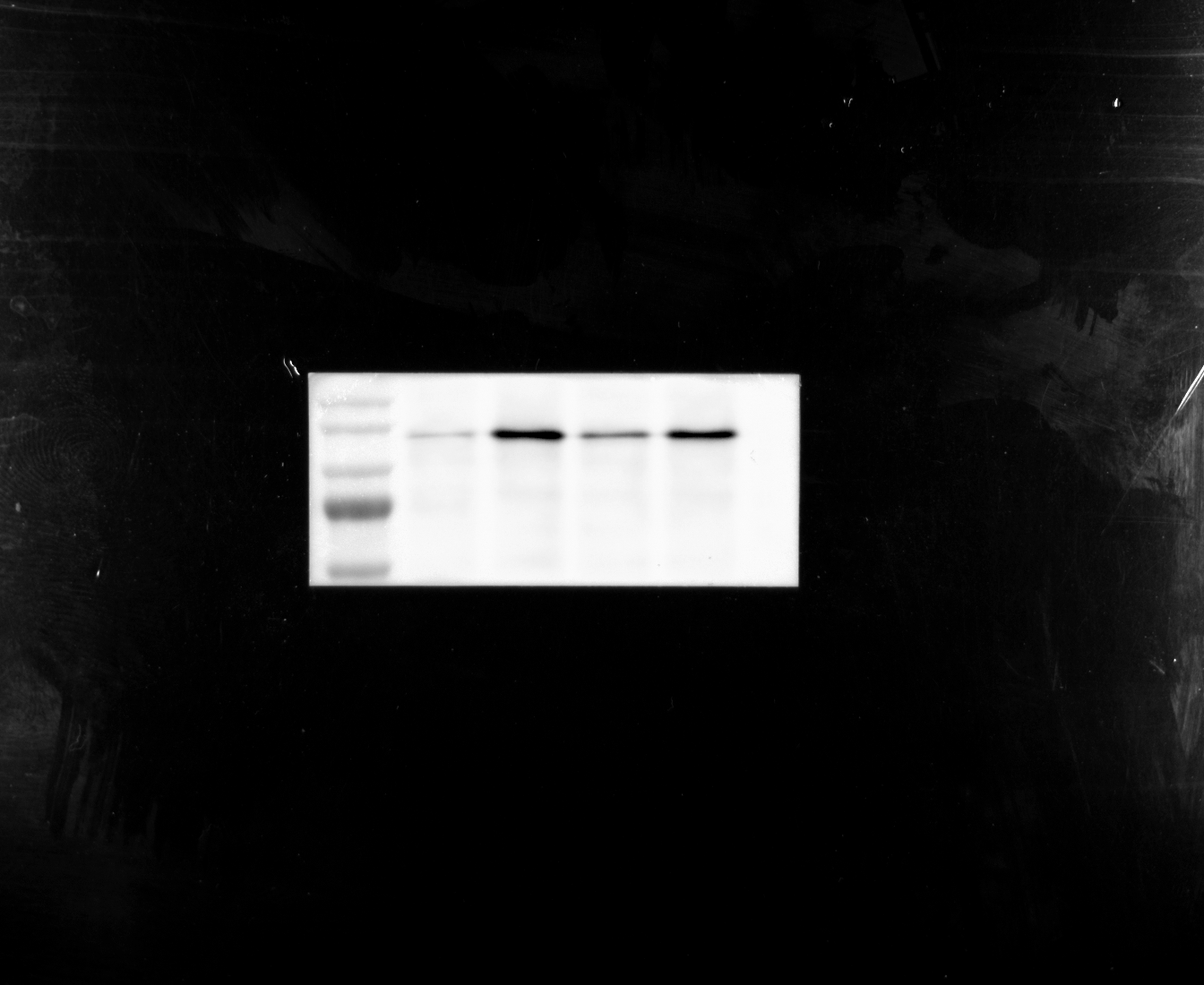

Supplement: Supplementary file 1 [file cimb-47-00936-s001.zip › cimb-3956315-supplementary/APOC2_ccRCC_RawWB_FullMembranes/cropped display images/5/Fig 3D p-jak1/2.Tif]

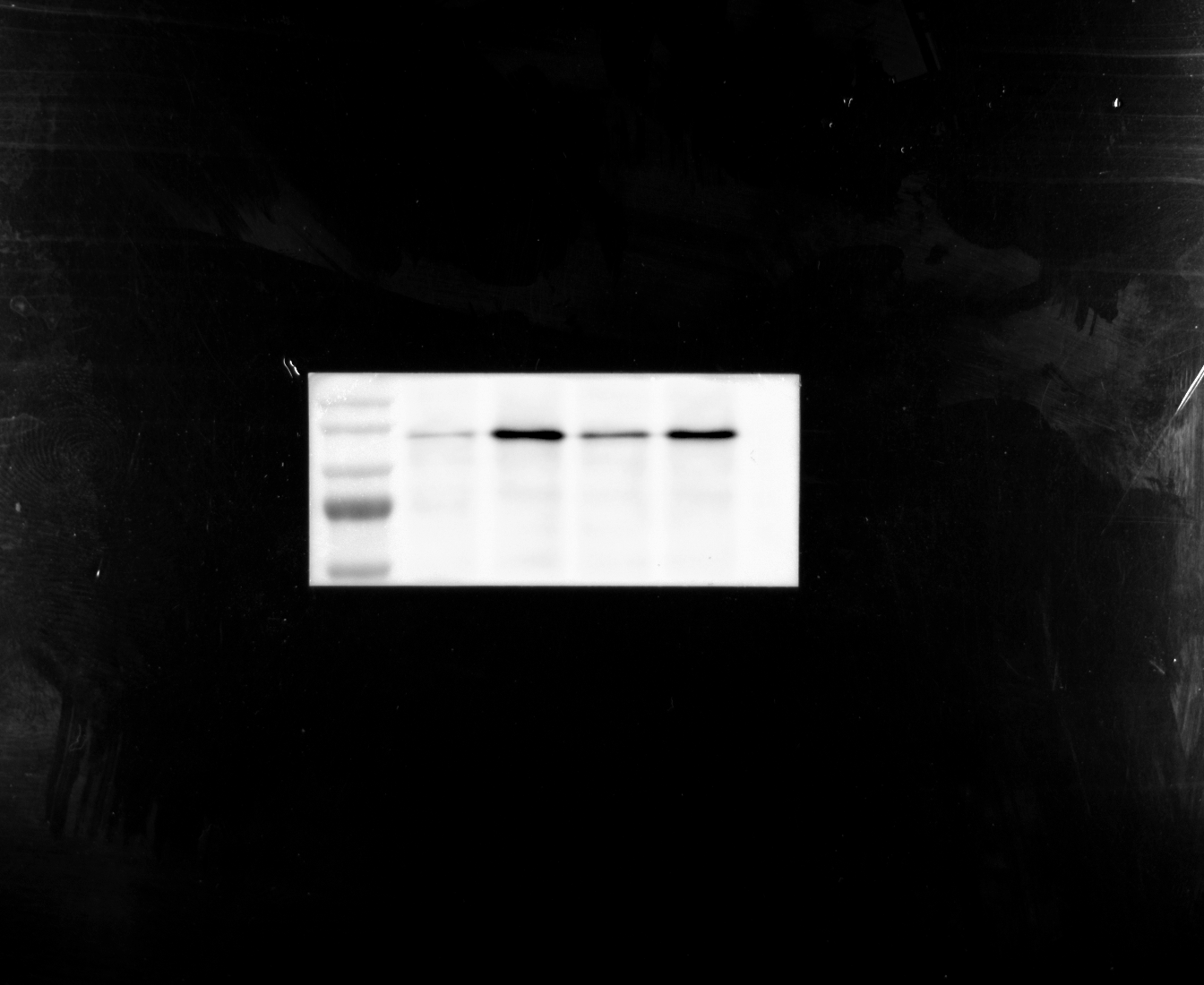

Supplement: Supplementary file 1 [file cimb-47-00936-s001.zip › cimb-3956315-supplementary/APOC2_ccRCC_RawWB_FullMembranes/cropped display images/5/Fig 3D p-jak1/3.Tif]

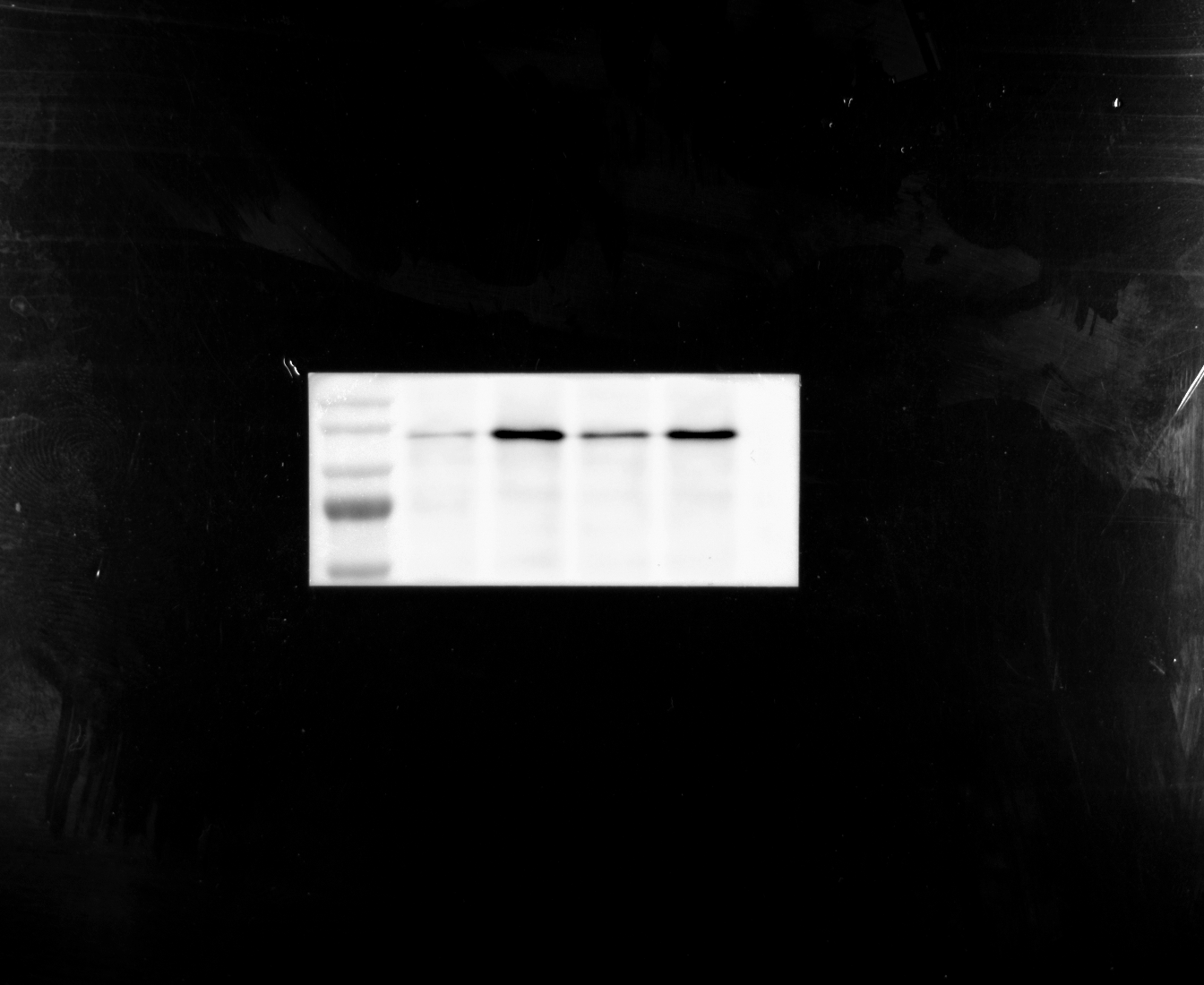

Supplement: Supplementary file 1 [file cimb-47-00936-s001.zip › cimb-3956315-supplementary/APOC2_ccRCC_RawWB_FullMembranes/cropped display images/5/Fig 3D p-jak1/4.Tif]

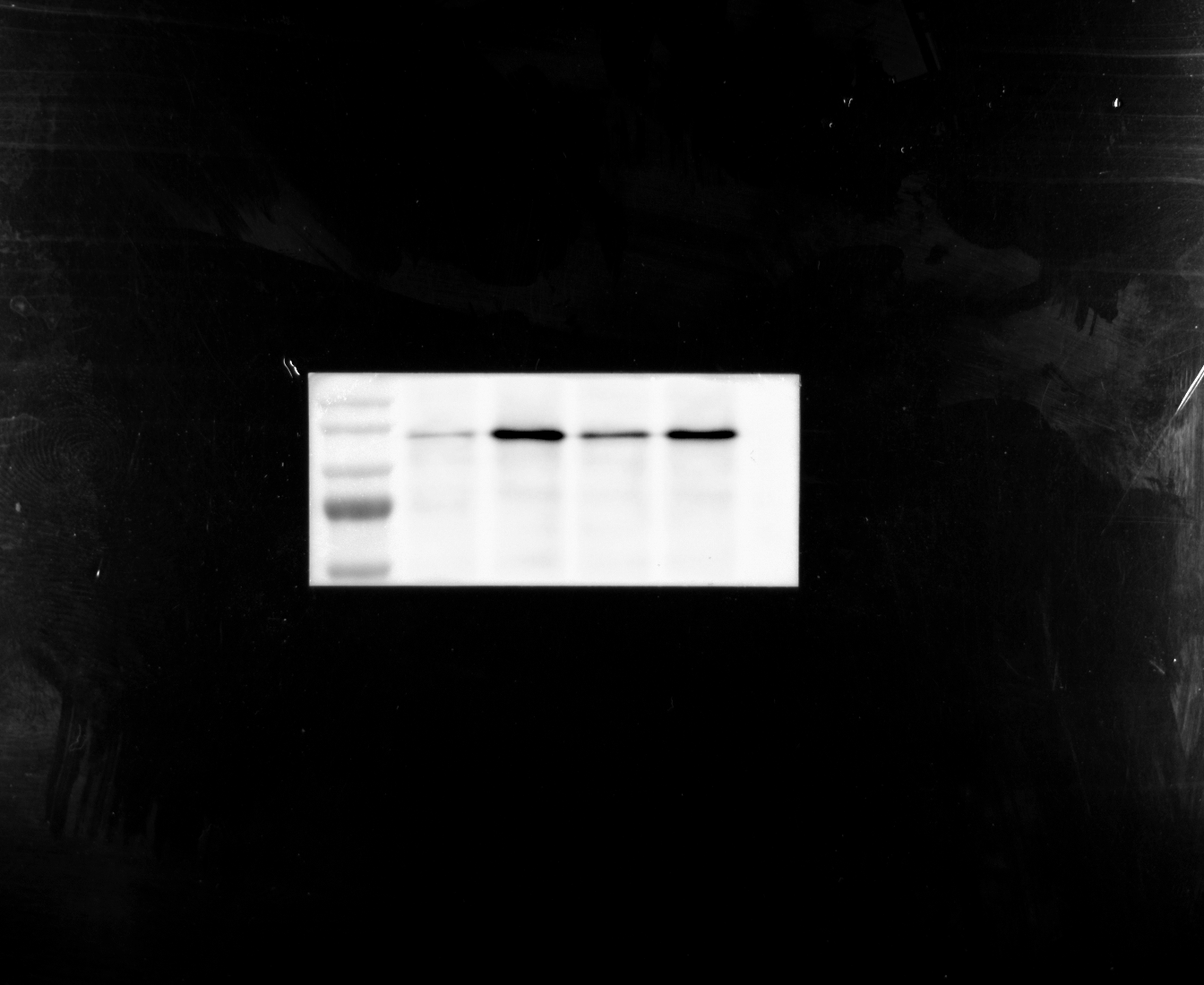

Supplement: Supplementary file 1 [file cimb-47-00936-s001.zip › cimb-3956315-supplementary/APOC2_ccRCC_RawWB_FullMembranes/cropped display images/5/Fig 3D p-jak1/5.Tif]

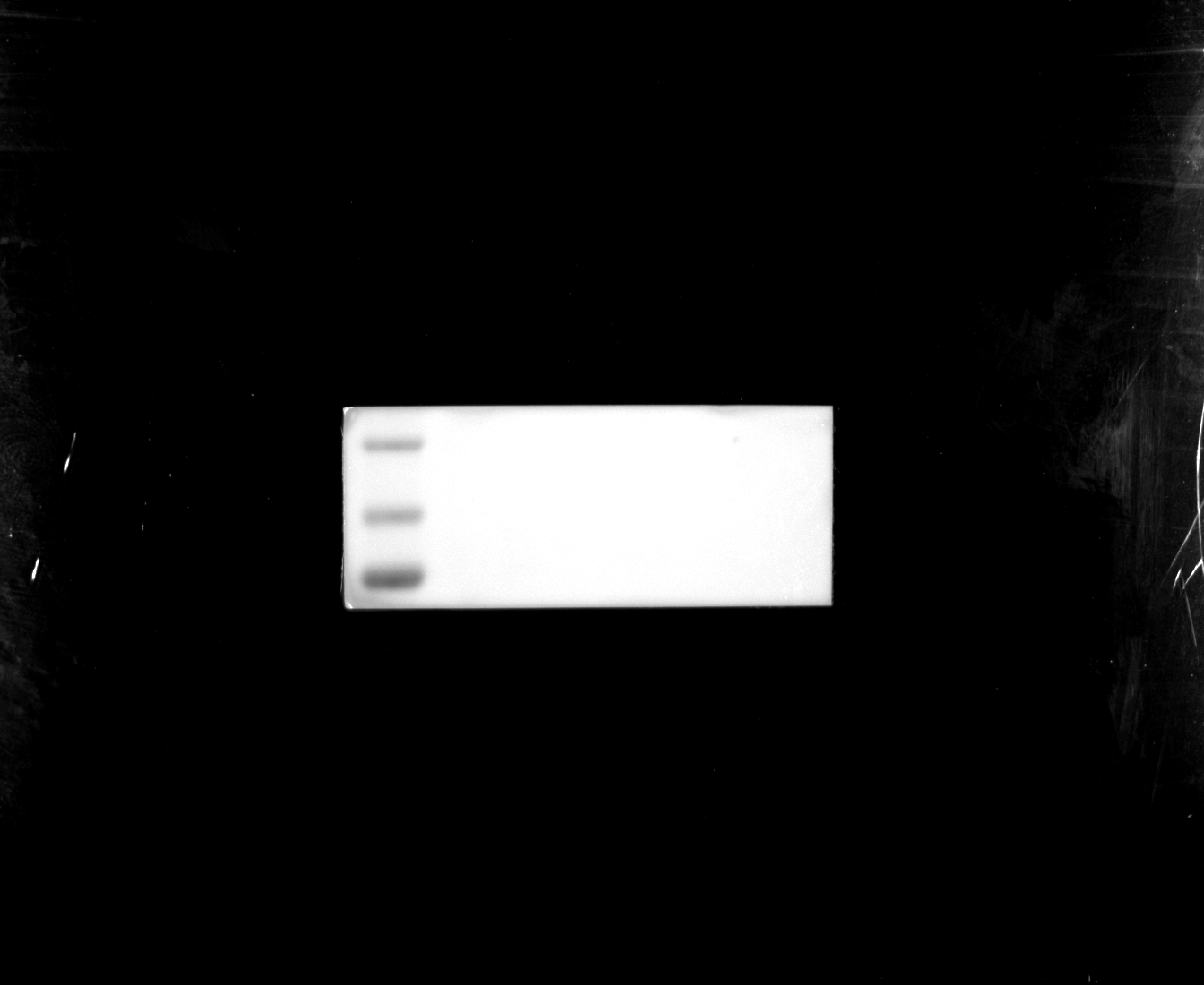

Supplement: Supplementary file 1 [file cimb-47-00936-s001.zip › cimb-3956315-supplementary/APOC2_ccRCC_RawWB_FullMembranes/cropped display images/5/β- actin/0.Tif]

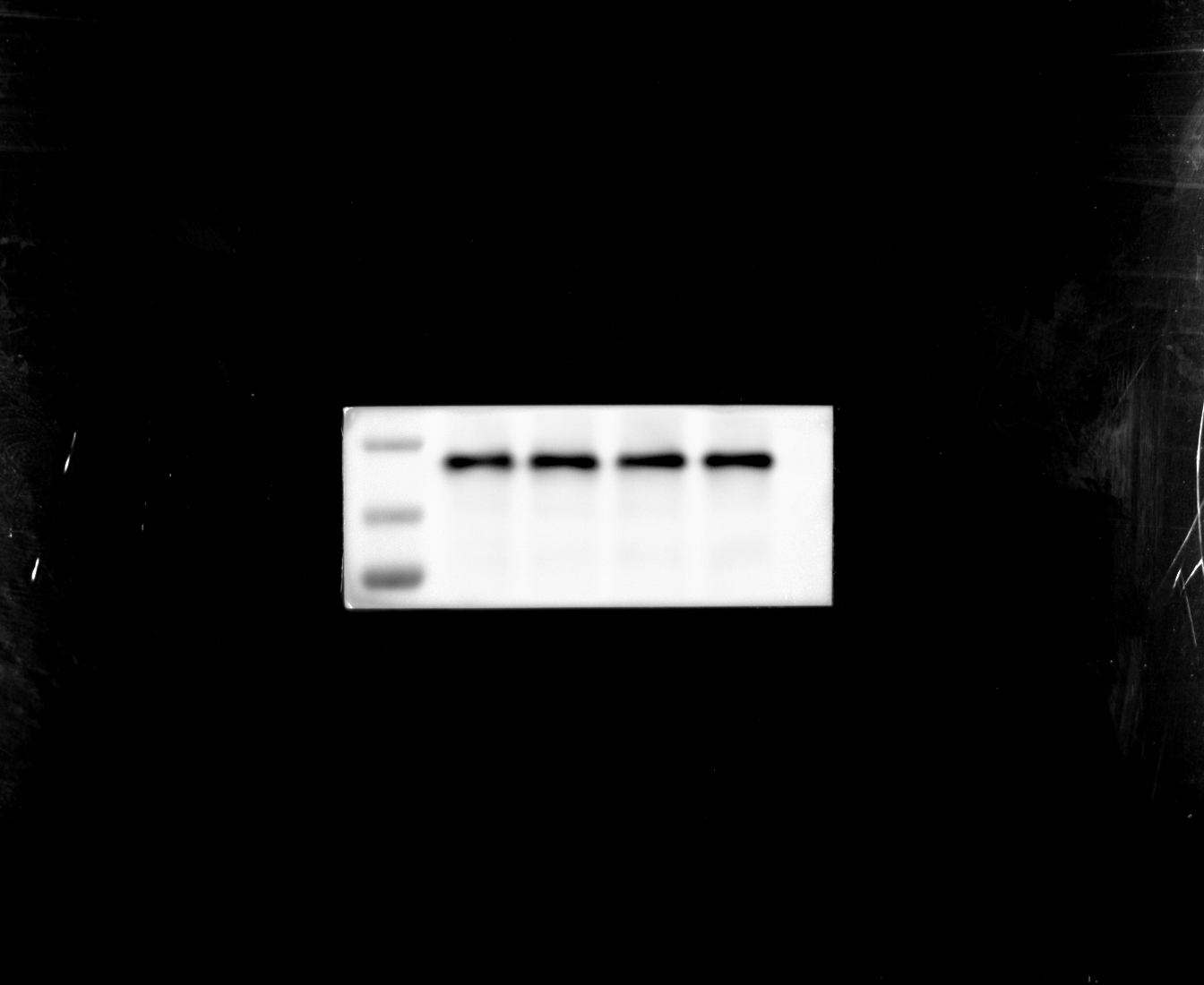

Supplement: Supplementary file 1 [file cimb-47-00936-s001.zip › cimb-3956315-supplementary/APOC2_ccRCC_RawWB_FullMembranes/cropped display images/5/β- actin/1.Tif]

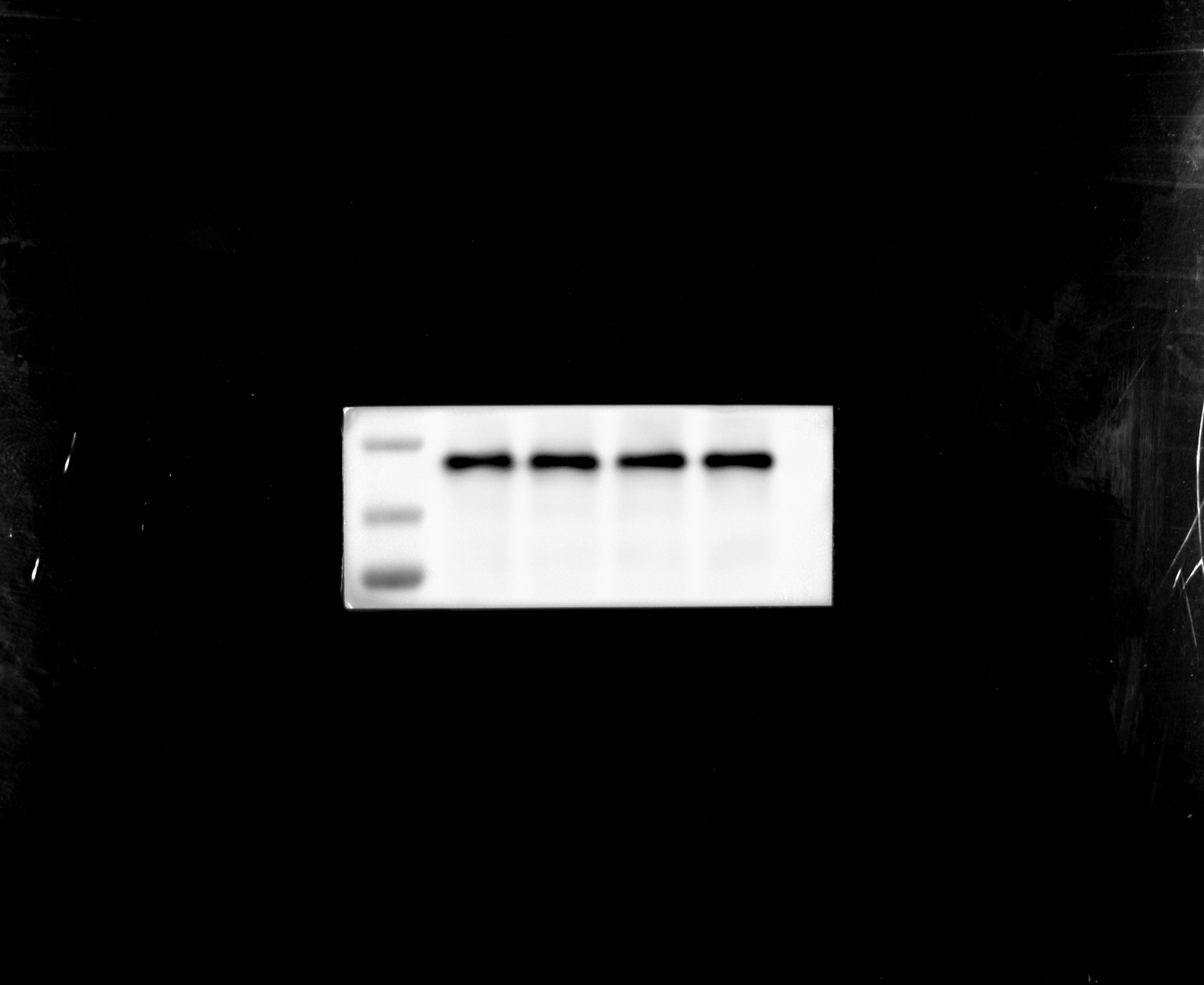

Supplement: Supplementary file 1 [file cimb-47-00936-s001.zip › cimb-3956315-supplementary/APOC2_ccRCC_RawWB_FullMembranes/cropped display images/5/β- actin/2.Tif]

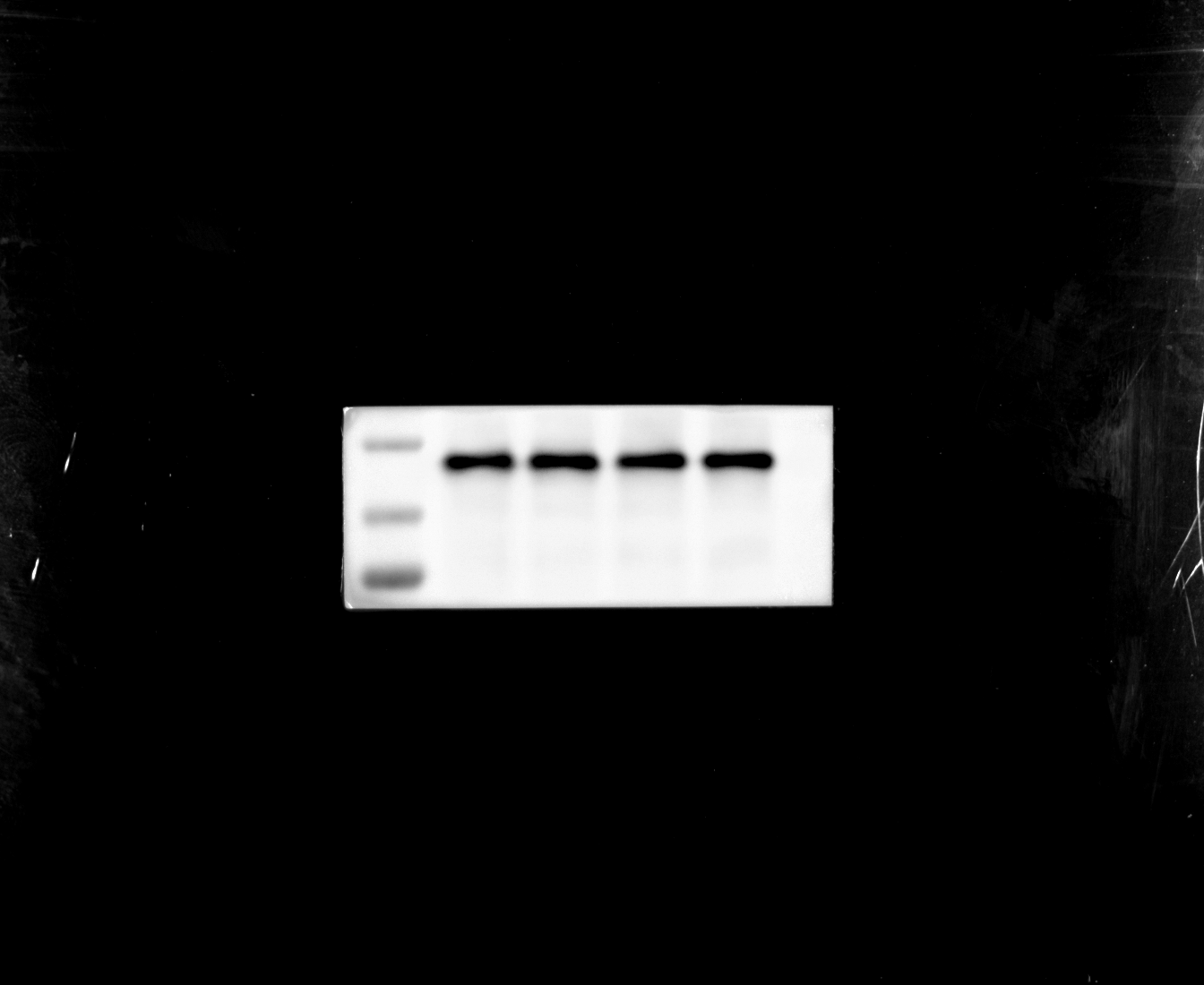

Supplement: Supplementary file 1 [file cimb-47-00936-s001.zip › cimb-3956315-supplementary/APOC2_ccRCC_RawWB_FullMembranes/cropped display images/5/β- actin/3.Tif]

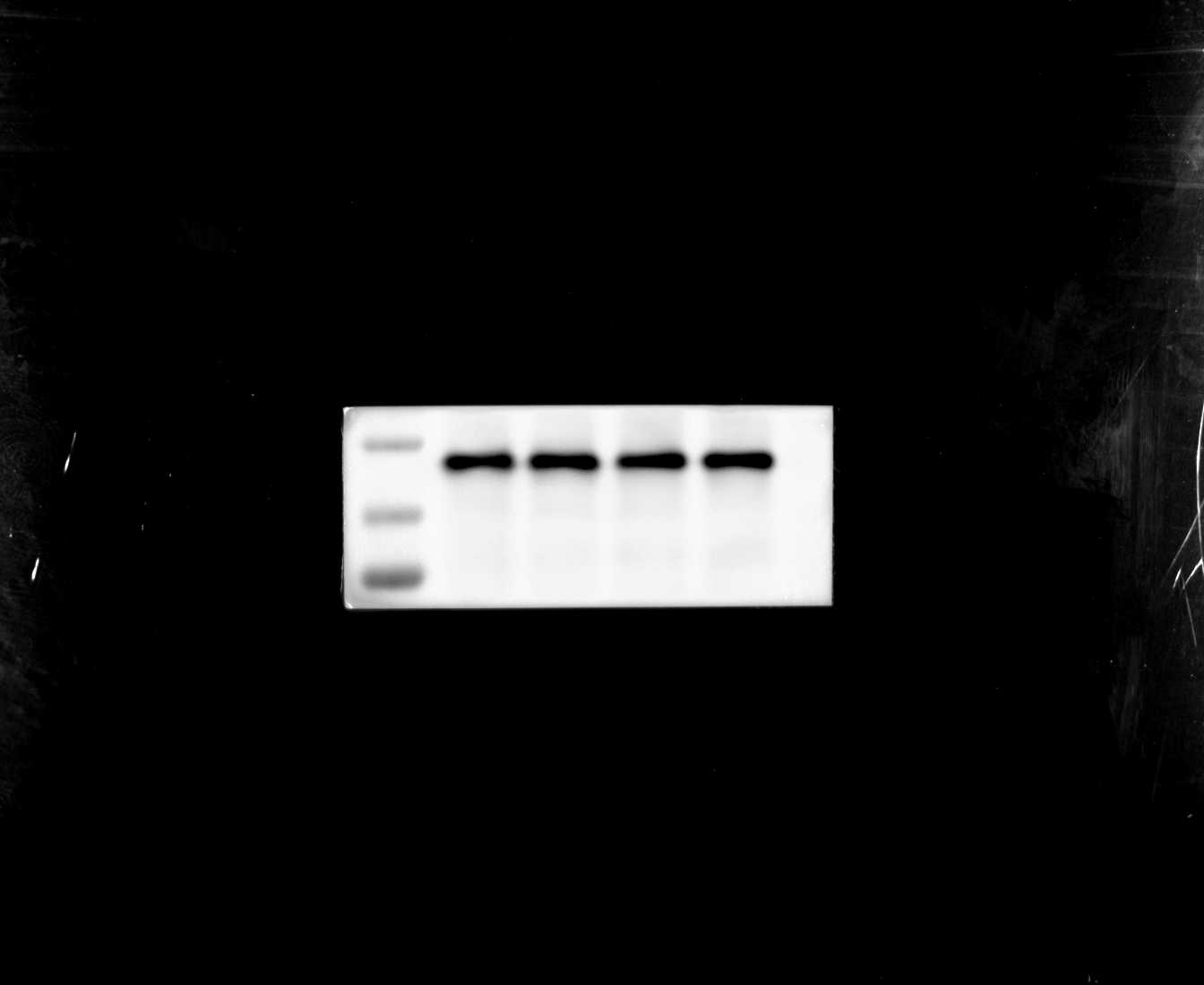

Supplement: Supplementary file 1 [file cimb-47-00936-s001.zip › cimb-3956315-supplementary/APOC2_ccRCC_RawWB_FullMembranes/cropped display images/5/β- actin/4.Tif]

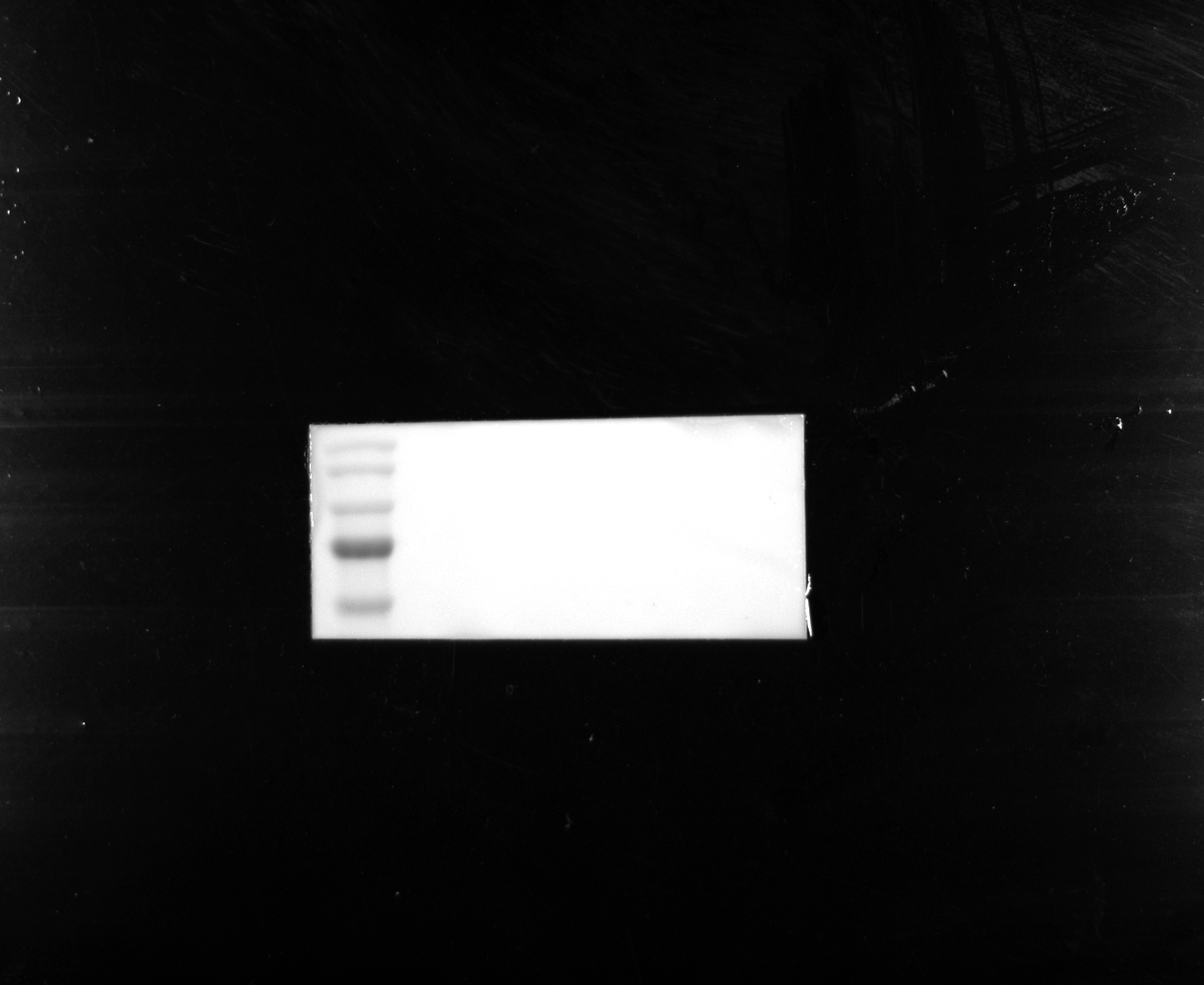

Supplement: Supplementary file 1 [file cimb-47-00936-s001.zip › cimb-3956315-supplementary/APOC2_ccRCC_RawWB_FullMembranes/cropped display images/6/Fig 3D jak2/0.Tif]

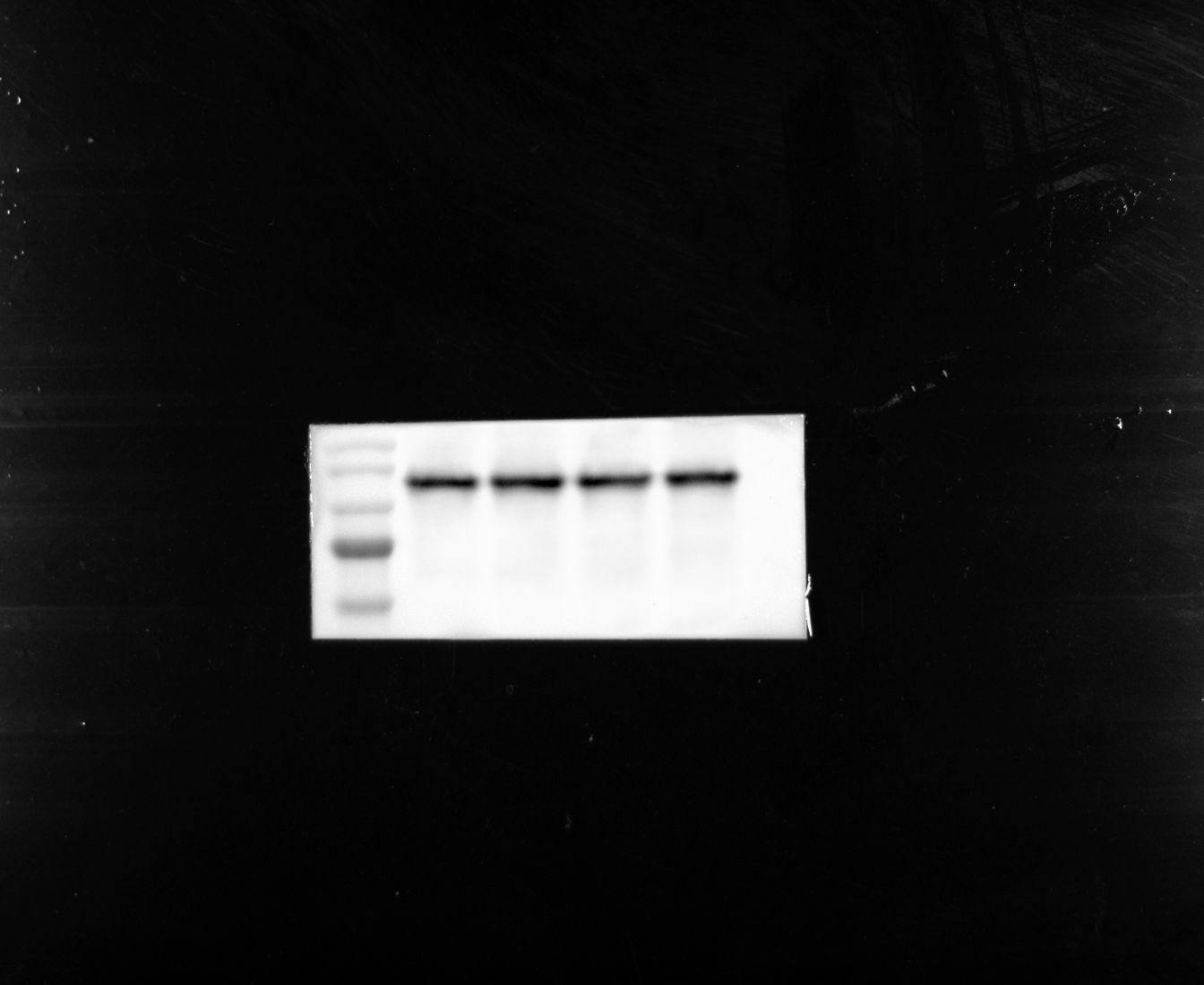

Supplement: Supplementary file 1 [file cimb-47-00936-s001.zip › cimb-3956315-supplementary/APOC2_ccRCC_RawWB_FullMembranes/cropped display images/6/Fig 3D jak2/1.Tif]

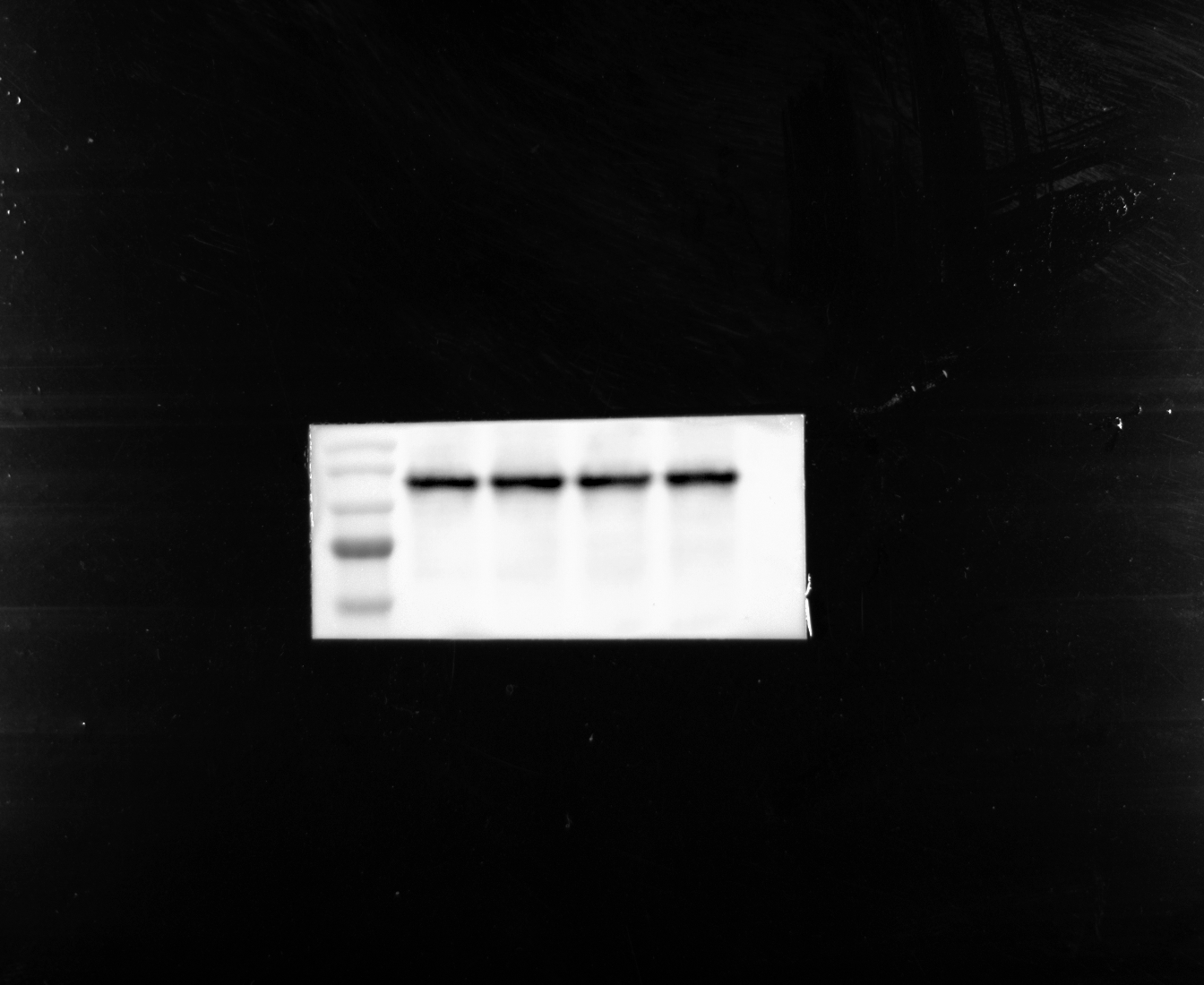

Supplement: Supplementary file 1 [file cimb-47-00936-s001.zip › cimb-3956315-supplementary/APOC2_ccRCC_RawWB_FullMembranes/cropped display images/6/Fig 3D jak2/2.Tif]

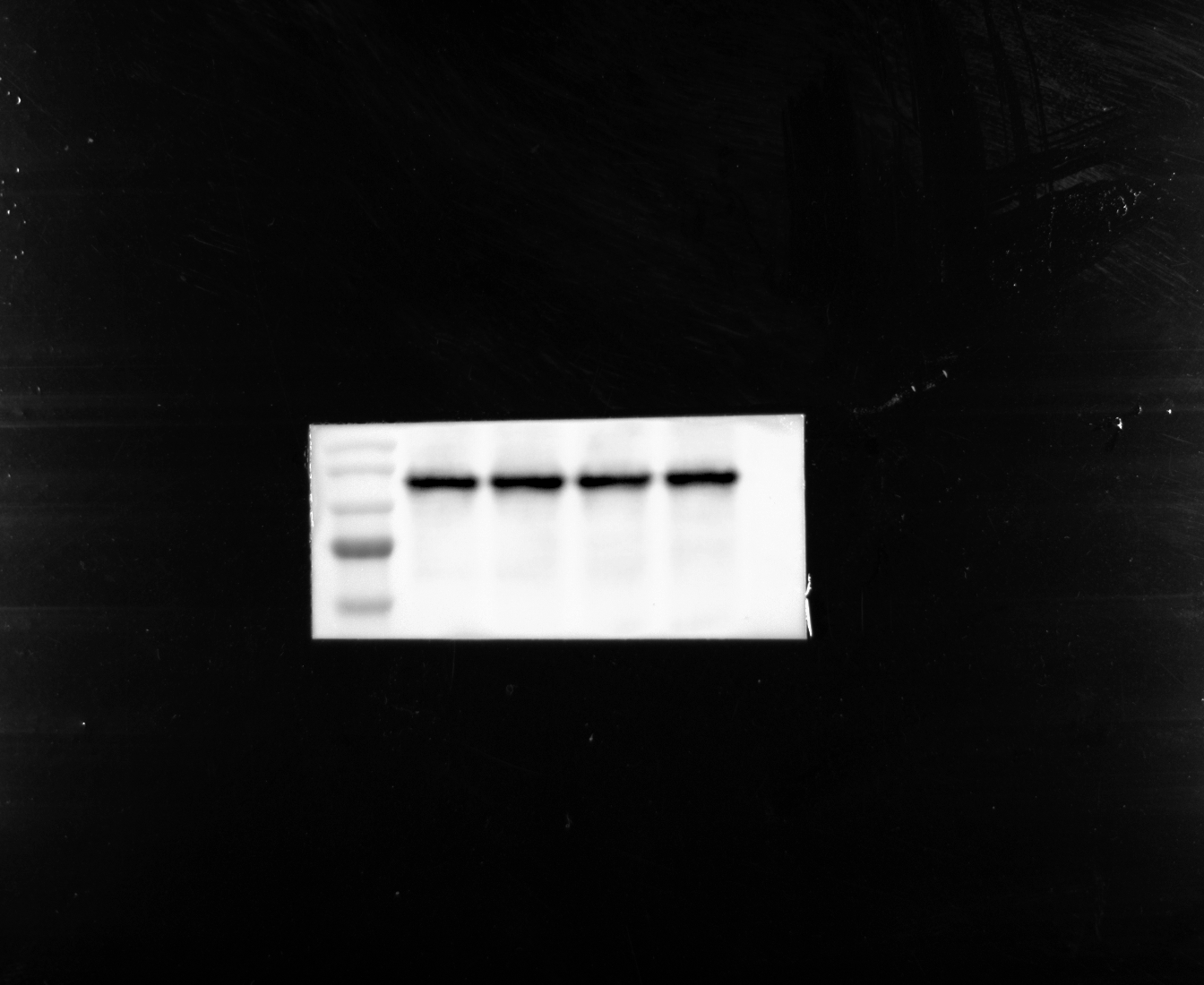

Supplement: Supplementary file 1 [file cimb-47-00936-s001.zip › cimb-3956315-supplementary/APOC2_ccRCC_RawWB_FullMembranes/cropped display images/6/Fig 3D jak2/3.Tif]

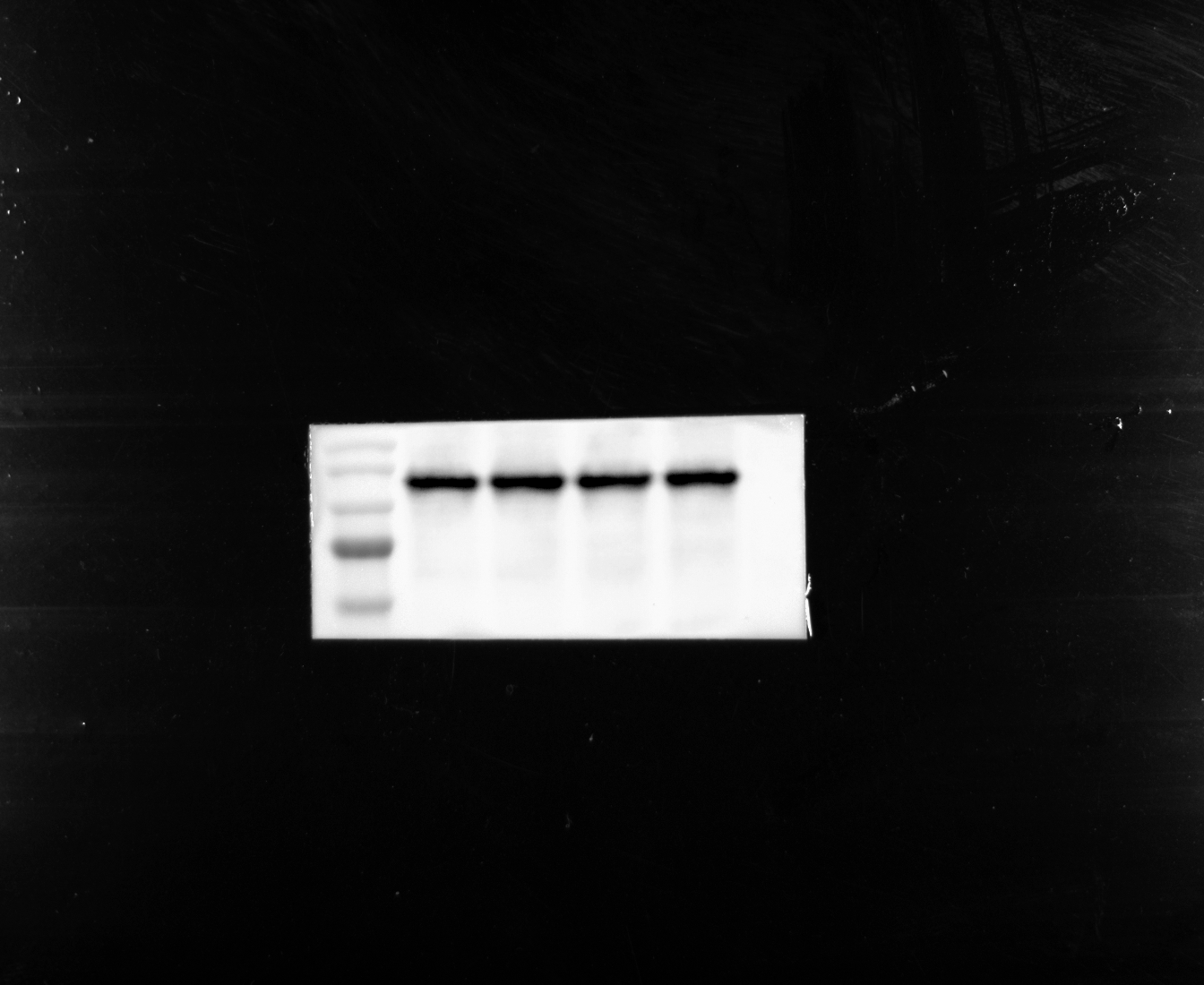

Supplement: Supplementary file 1 [file cimb-47-00936-s001.zip › cimb-3956315-supplementary/APOC2_ccRCC_RawWB_FullMembranes/cropped display images/6/Fig 3D jak2/4.Tif]

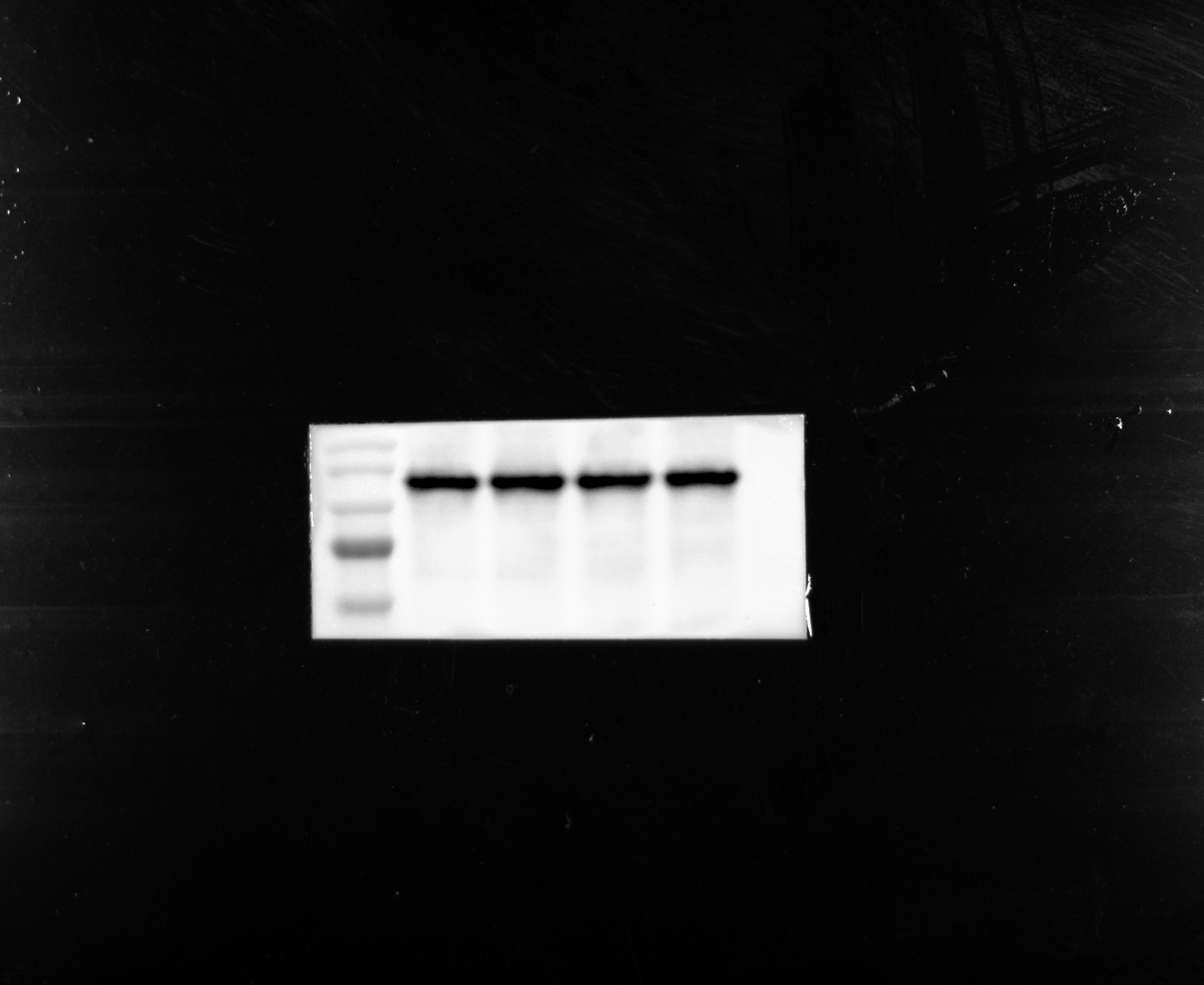

Supplement: Supplementary file 1 [file cimb-47-00936-s001.zip › cimb-3956315-supplementary/APOC2_ccRCC_RawWB_FullMembranes/cropped display images/6/Fig 3D jak2/5.Tif]

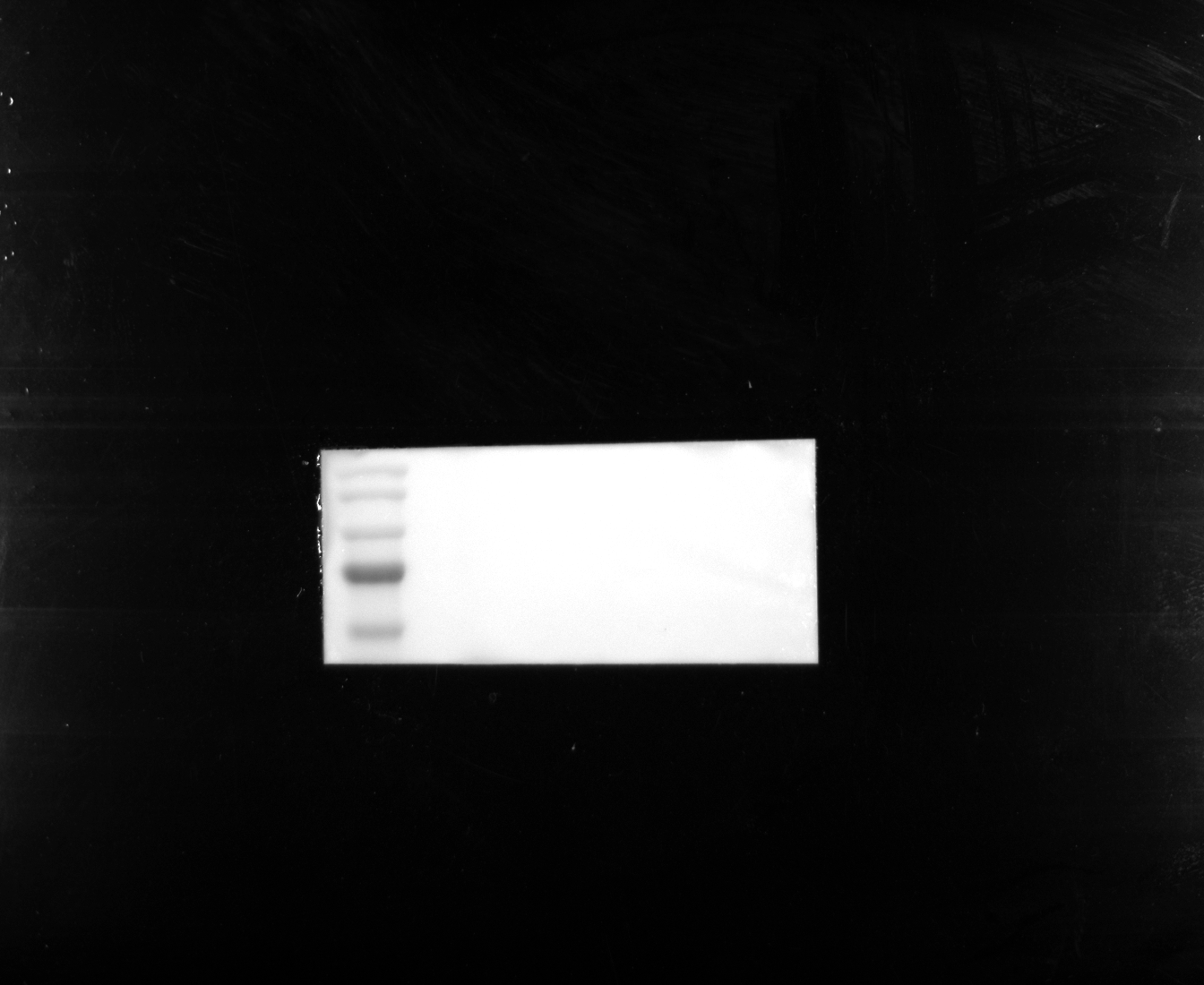

Supplement: Supplementary file 1 [file cimb-47-00936-s001.zip › cimb-3956315-supplementary/APOC2_ccRCC_RawWB_FullMembranes/cropped display images/6/Fig 3D p-jak2/0.Tif]

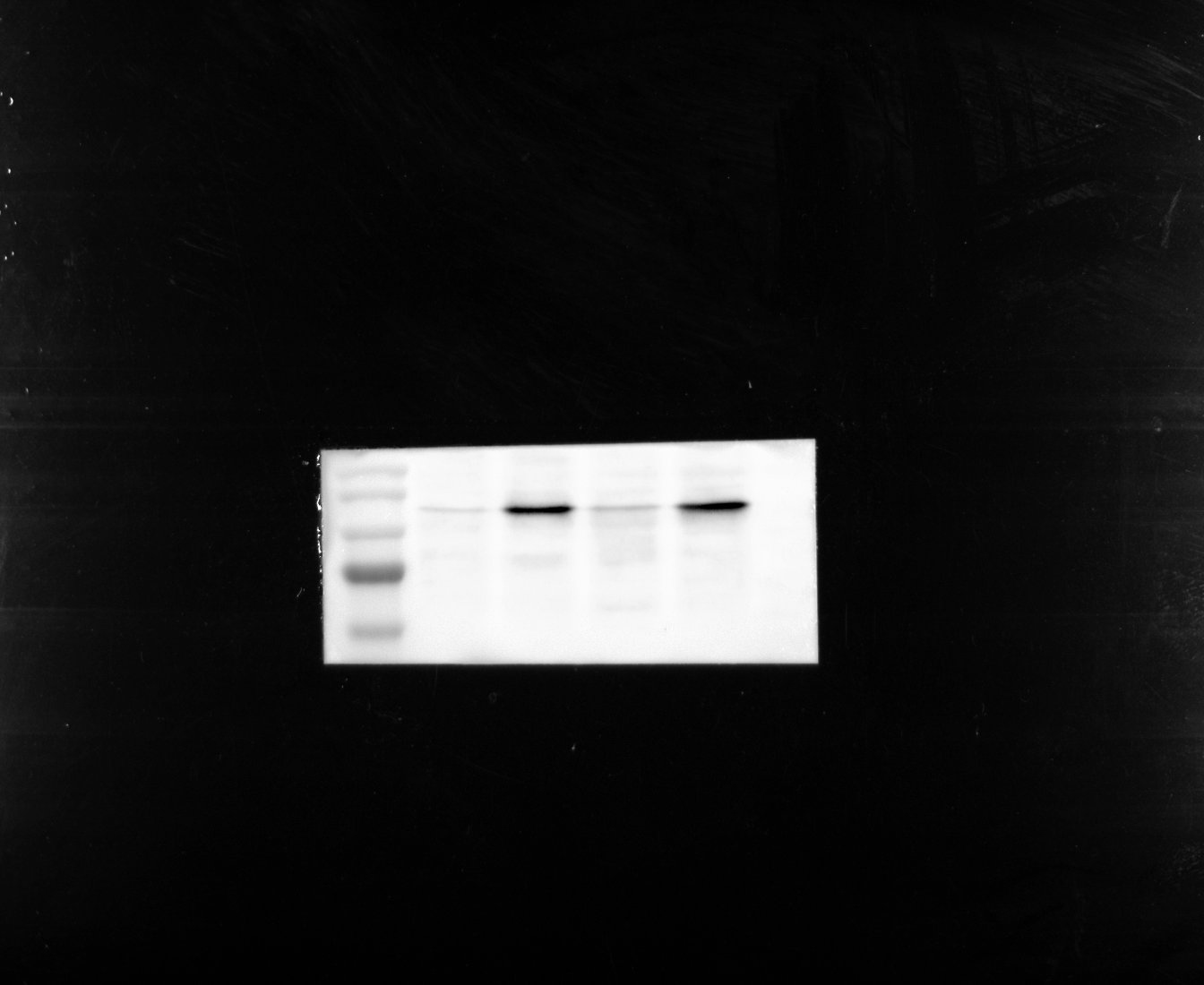

Supplement: Supplementary file 1 [file cimb-47-00936-s001.zip › cimb-3956315-supplementary/APOC2_ccRCC_RawWB_FullMembranes/cropped display images/6/Fig 3D p-jak2/1.Tif]

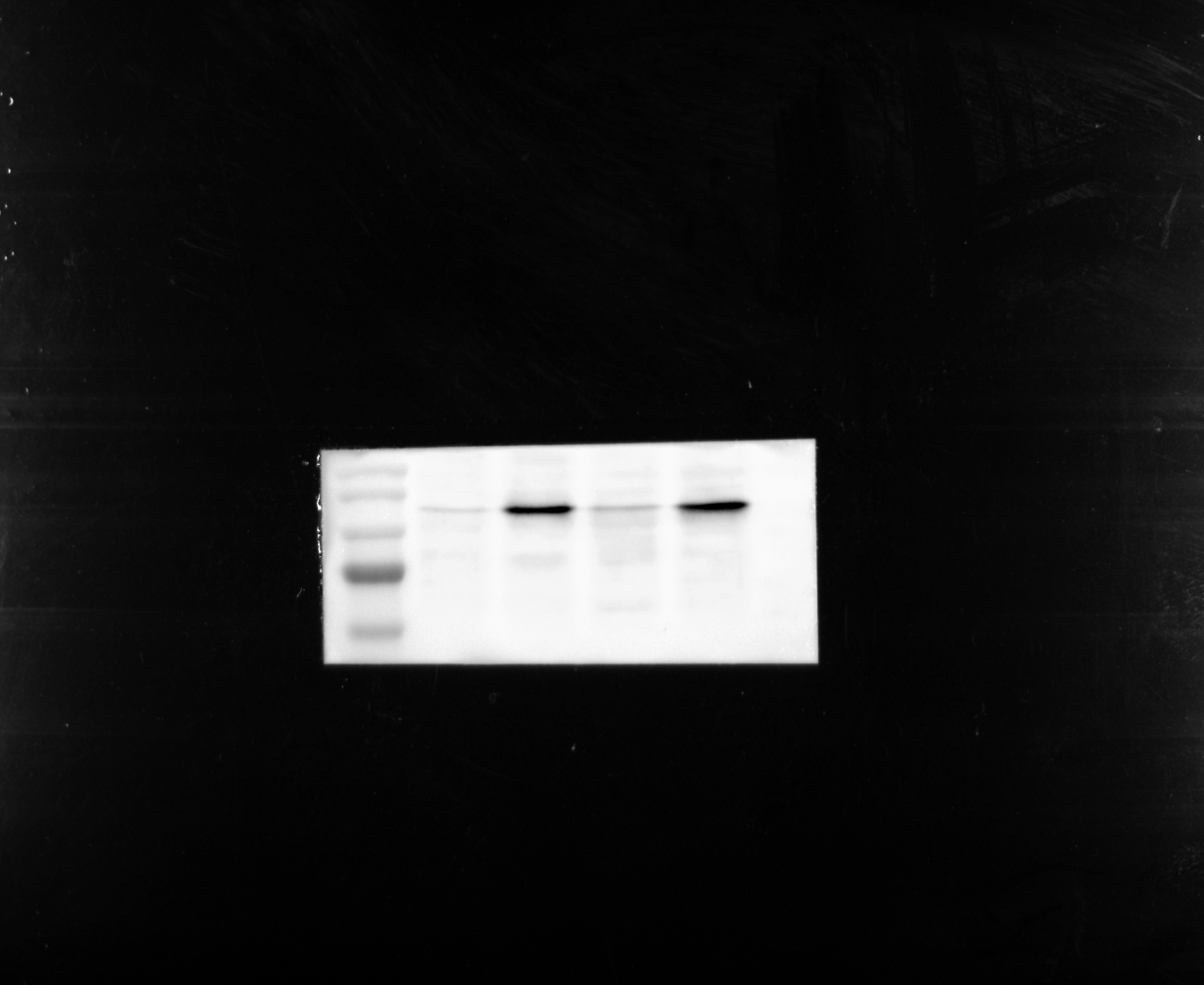

Supplement: Supplementary file 1 [file cimb-47-00936-s001.zip › cimb-3956315-supplementary/APOC2_ccRCC_RawWB_FullMembranes/cropped display images/6/Fig 3D p-jak2/2.Tif]

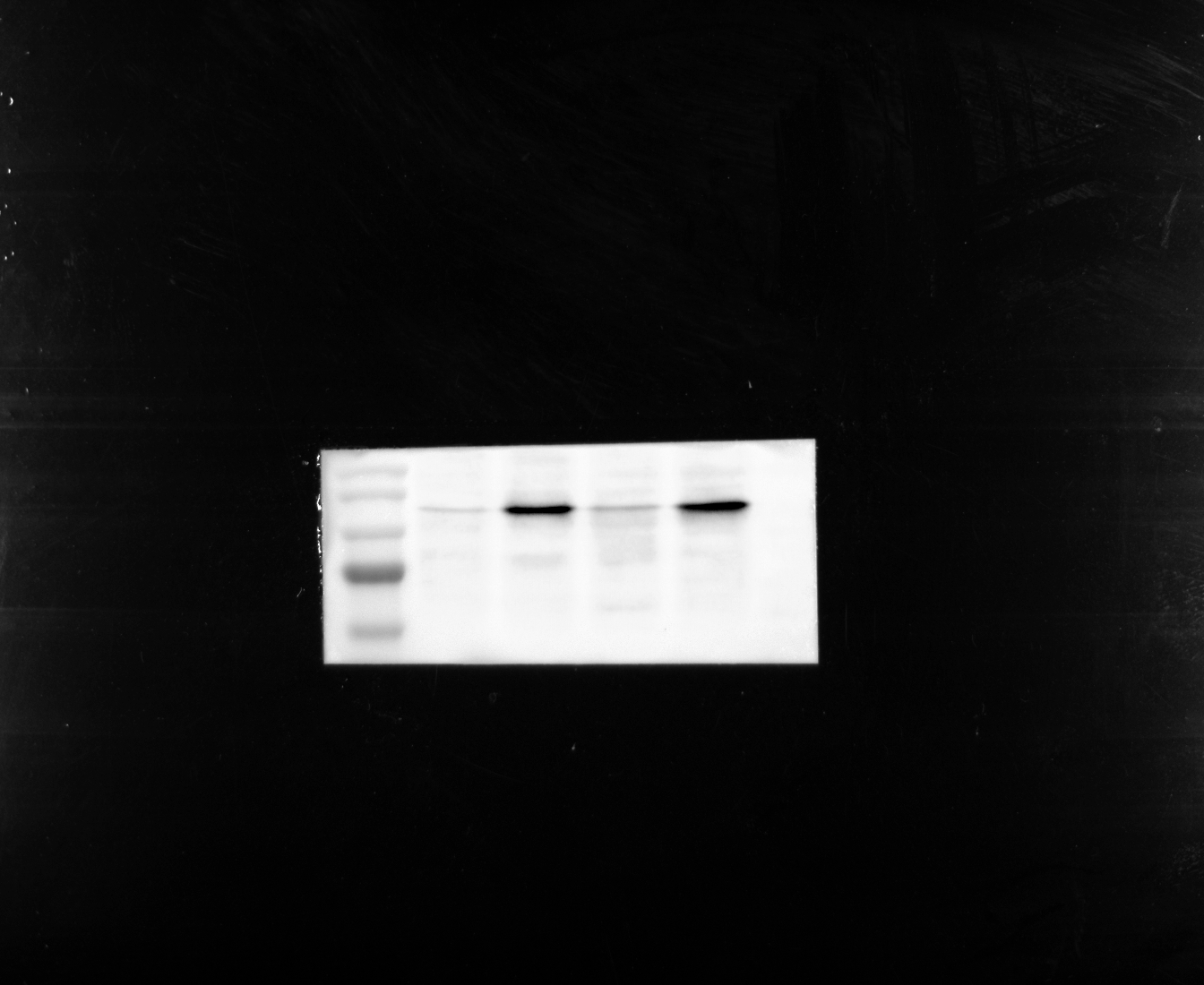

Supplement: Supplementary file 1 [file cimb-47-00936-s001.zip › cimb-3956315-supplementary/APOC2_ccRCC_RawWB_FullMembranes/cropped display images/6/Fig 3D p-jak2/3.Tif]

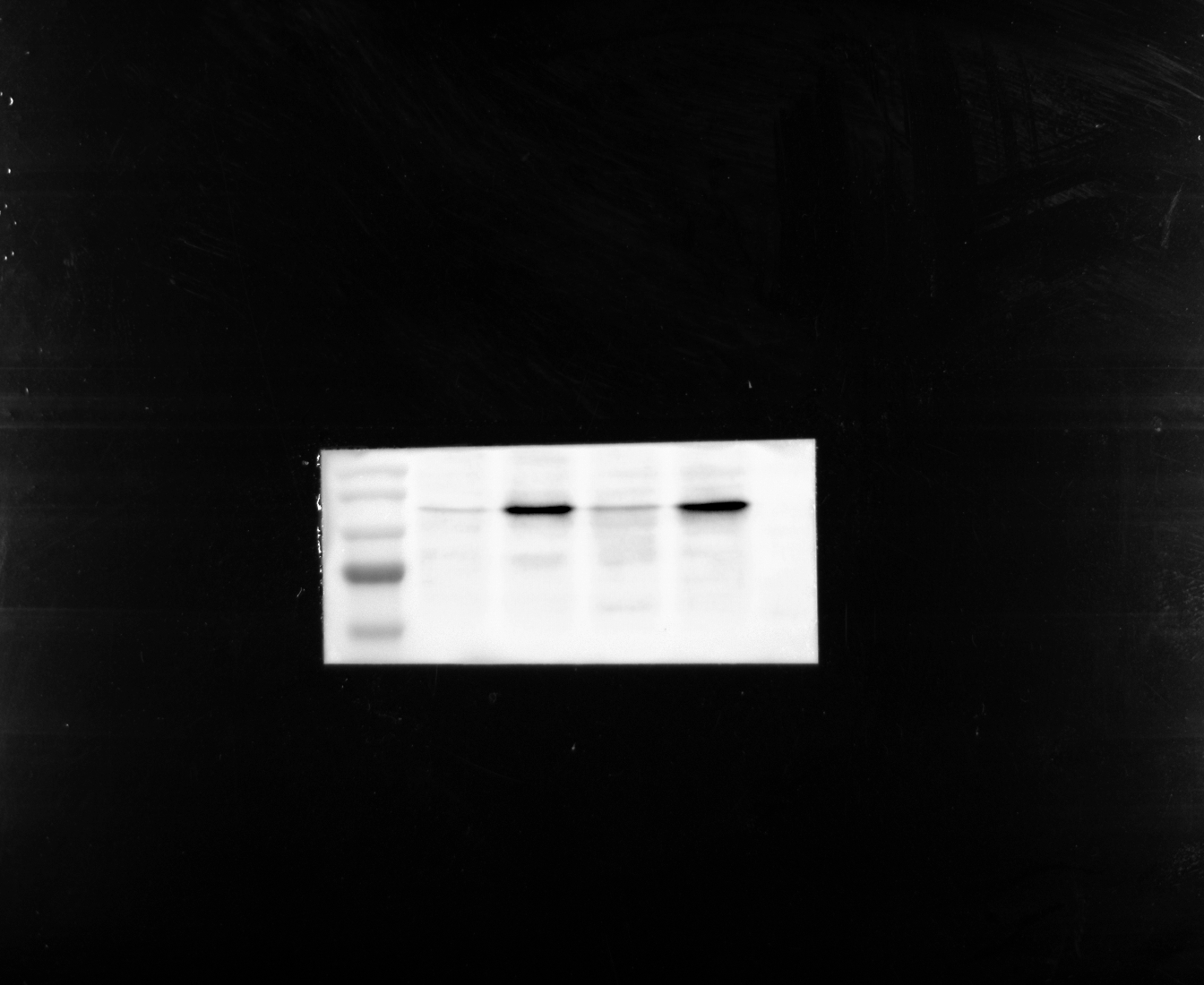

Supplement: Supplementary file 1 [file cimb-47-00936-s001.zip › cimb-3956315-supplementary/APOC2_ccRCC_RawWB_FullMembranes/cropped display images/6/Fig 3D p-jak2/4.Tif]

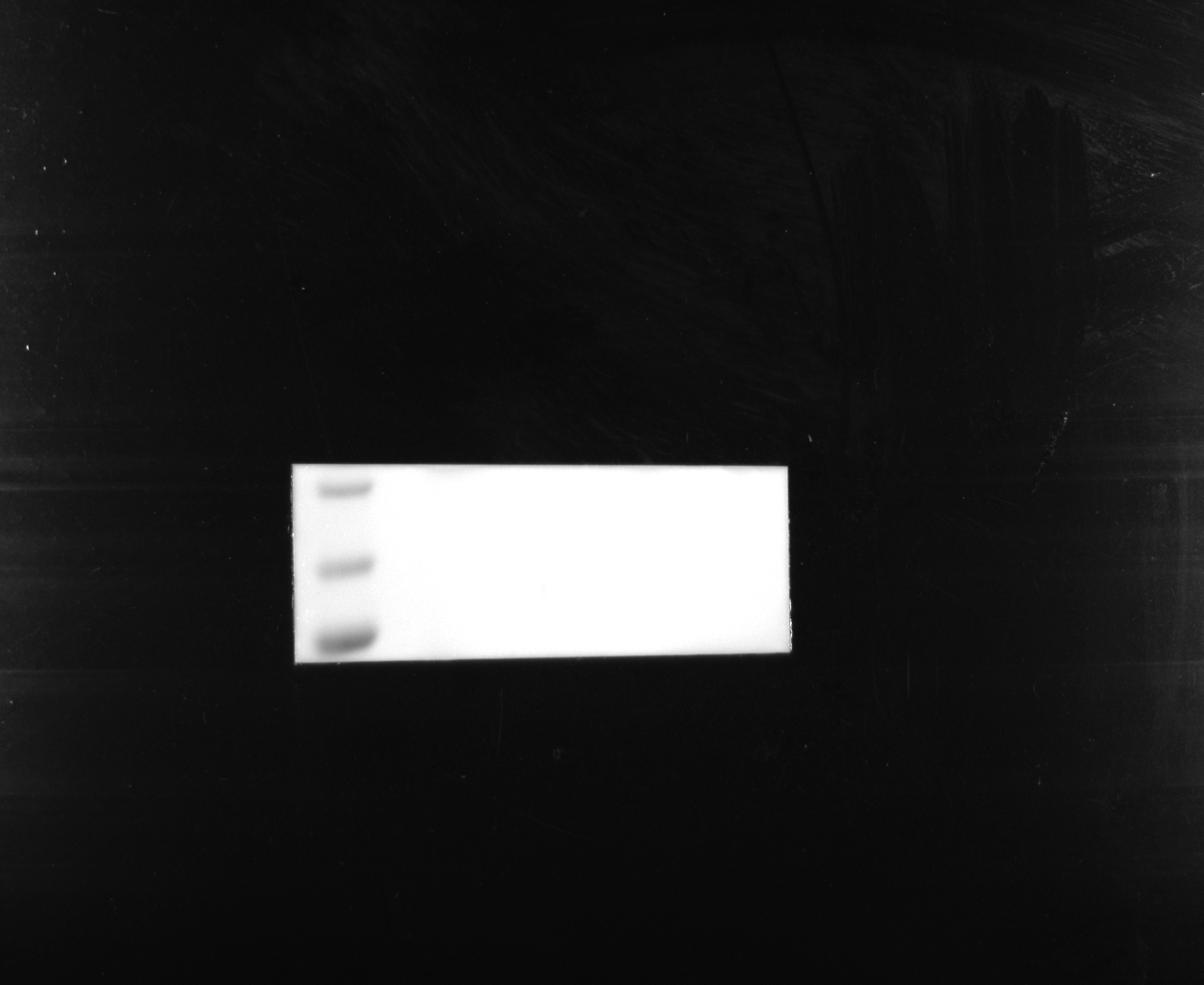

Supplement: Supplementary file 1 [file cimb-47-00936-s001.zip › cimb-3956315-supplementary/APOC2_ccRCC_RawWB_FullMembranes/cropped display images/6/β- actin/0.Tif]

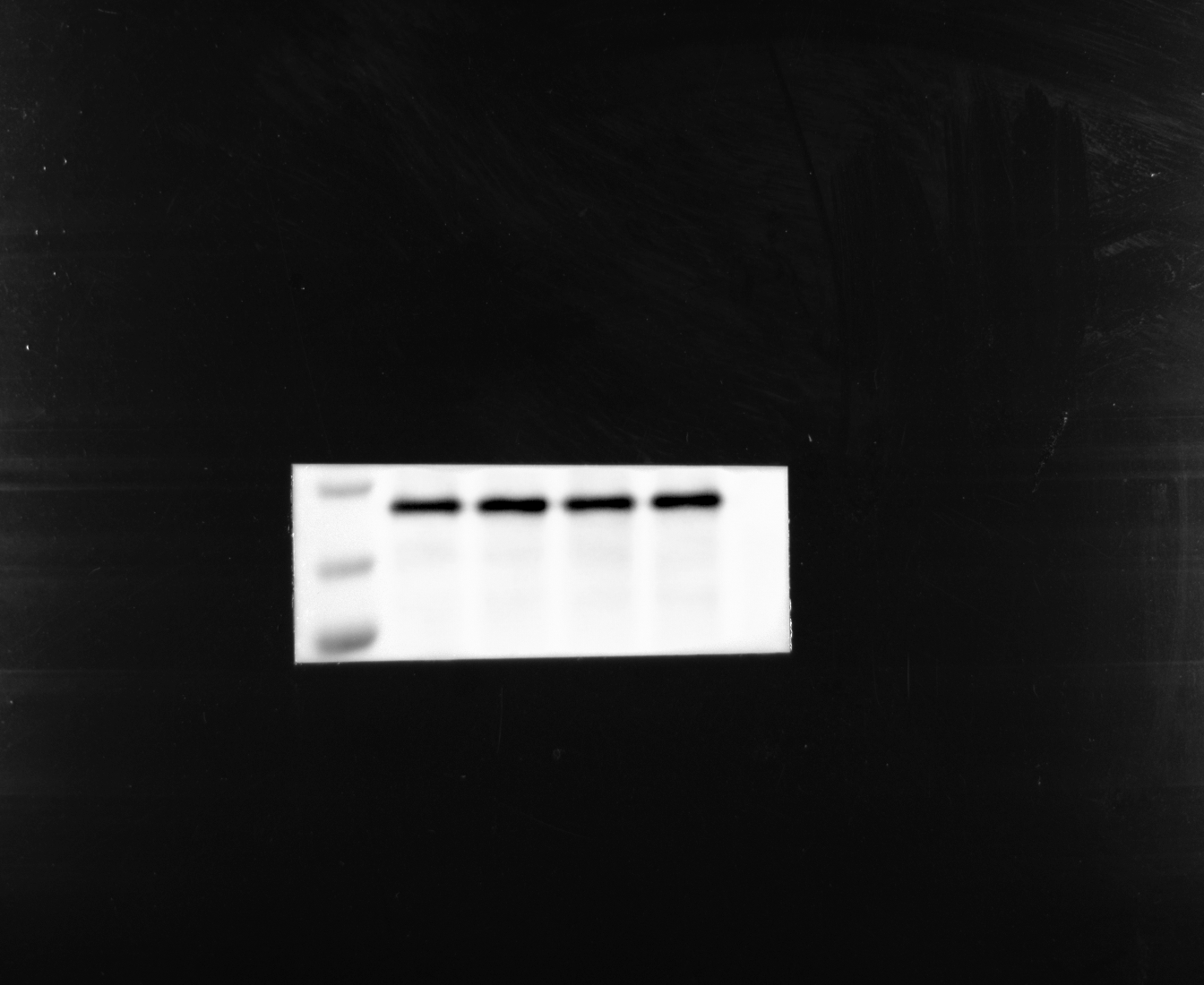

Supplement: Supplementary file 1 [file cimb-47-00936-s001.zip › cimb-3956315-supplementary/APOC2_ccRCC_RawWB_FullMembranes/cropped display images/6/β- actin/1.Tif]

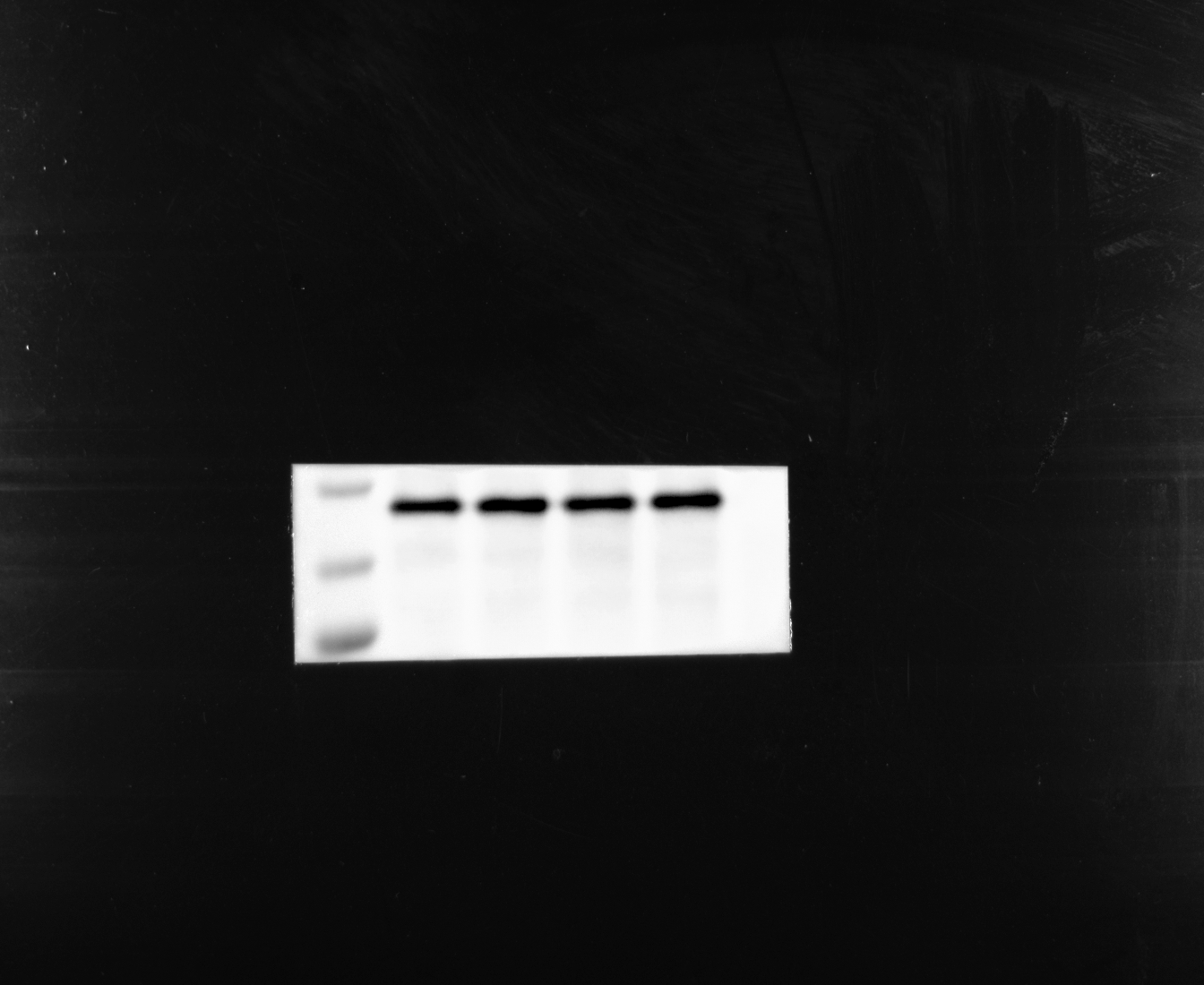

Supplement: Supplementary file 1 [file cimb-47-00936-s001.zip › cimb-3956315-supplementary/APOC2_ccRCC_RawWB_FullMembranes/cropped display images/6/β- actin/2.Tif]

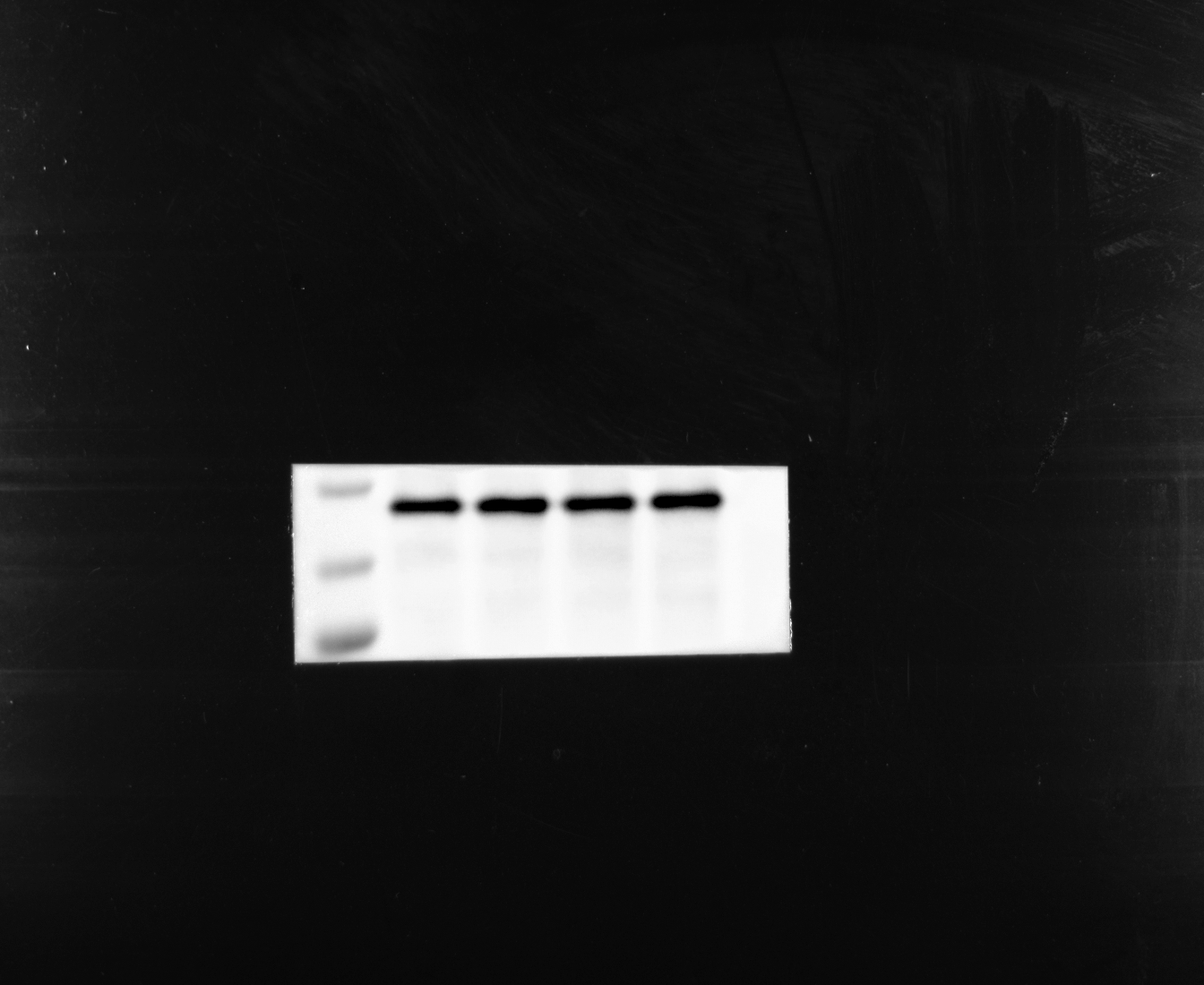

Supplement: Supplementary file 1 [file cimb-47-00936-s001.zip › cimb-3956315-supplementary/APOC2_ccRCC_RawWB_FullMembranes/cropped display images/6/β- actin/3.Tif]

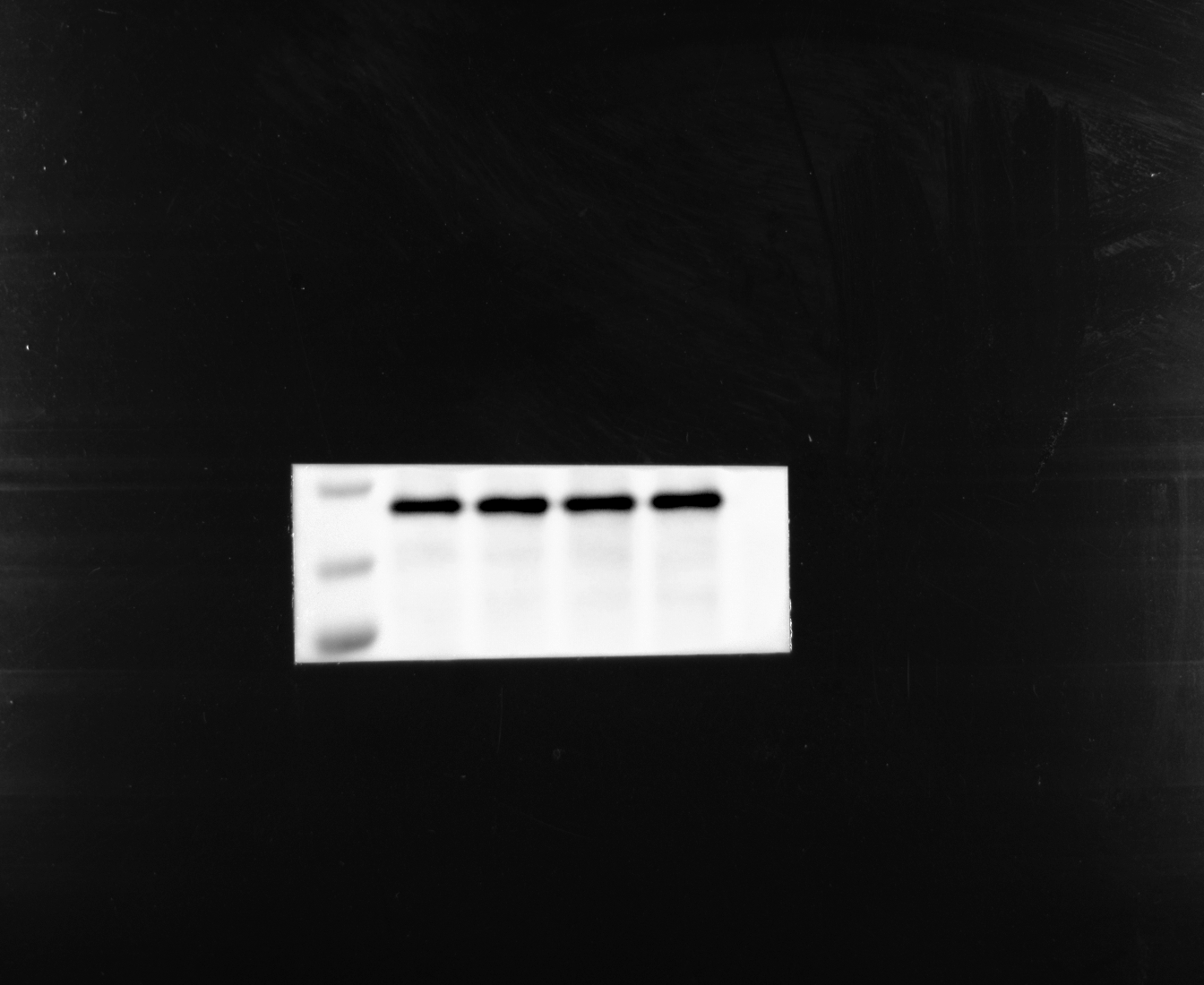

Supplement: Supplementary file 1 [file cimb-47-00936-s001.zip › cimb-3956315-supplementary/APOC2_ccRCC_RawWB_FullMembranes/cropped display images/6/β- actin/4.Tif]

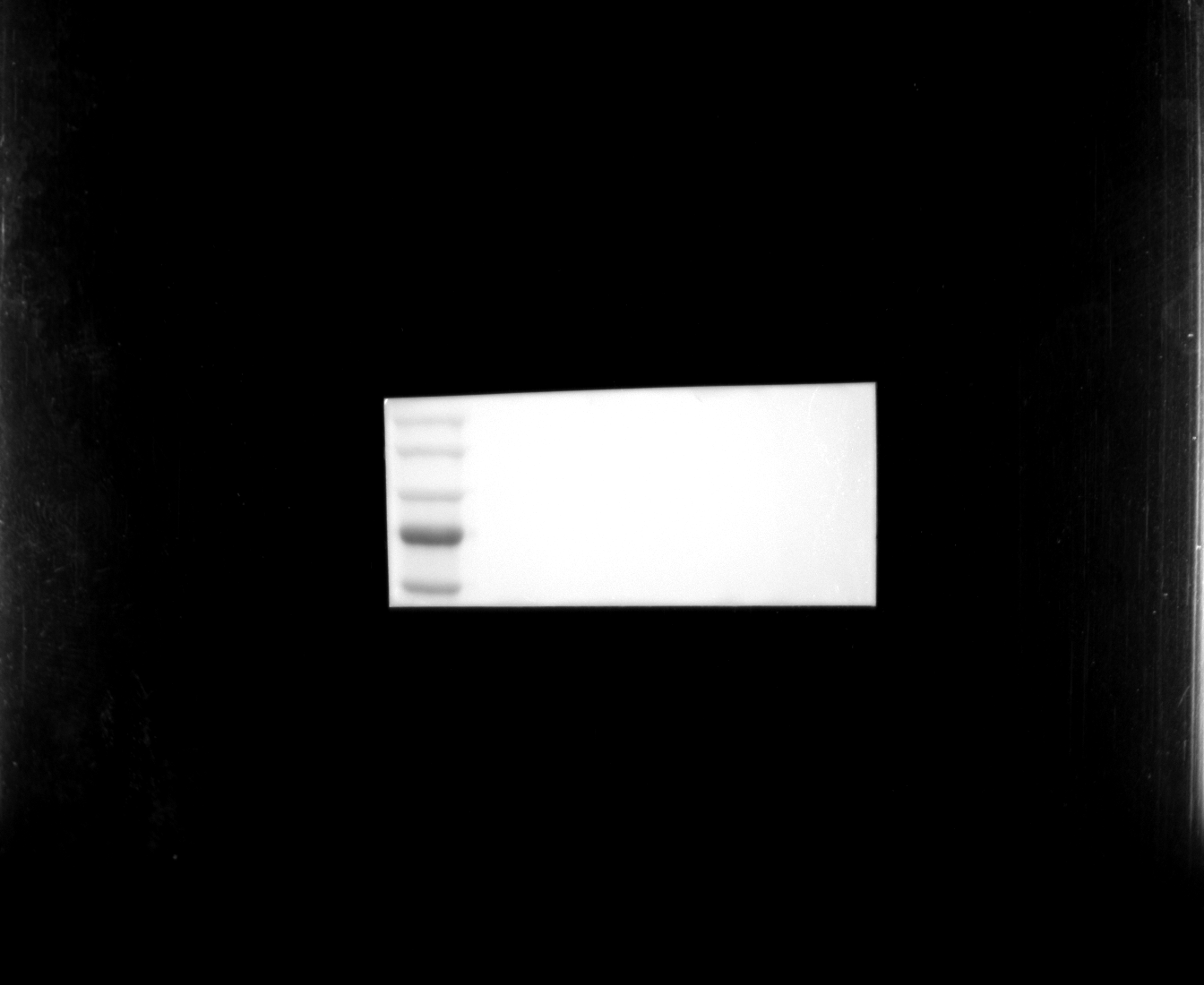

Supplement: Supplementary file 1 [file cimb-47-00936-s001.zip › cimb-3956315-supplementary/APOC2_ccRCC_RawWB_FullMembranes/cropped display images/7/Fig 3E jak3/0.Tif]

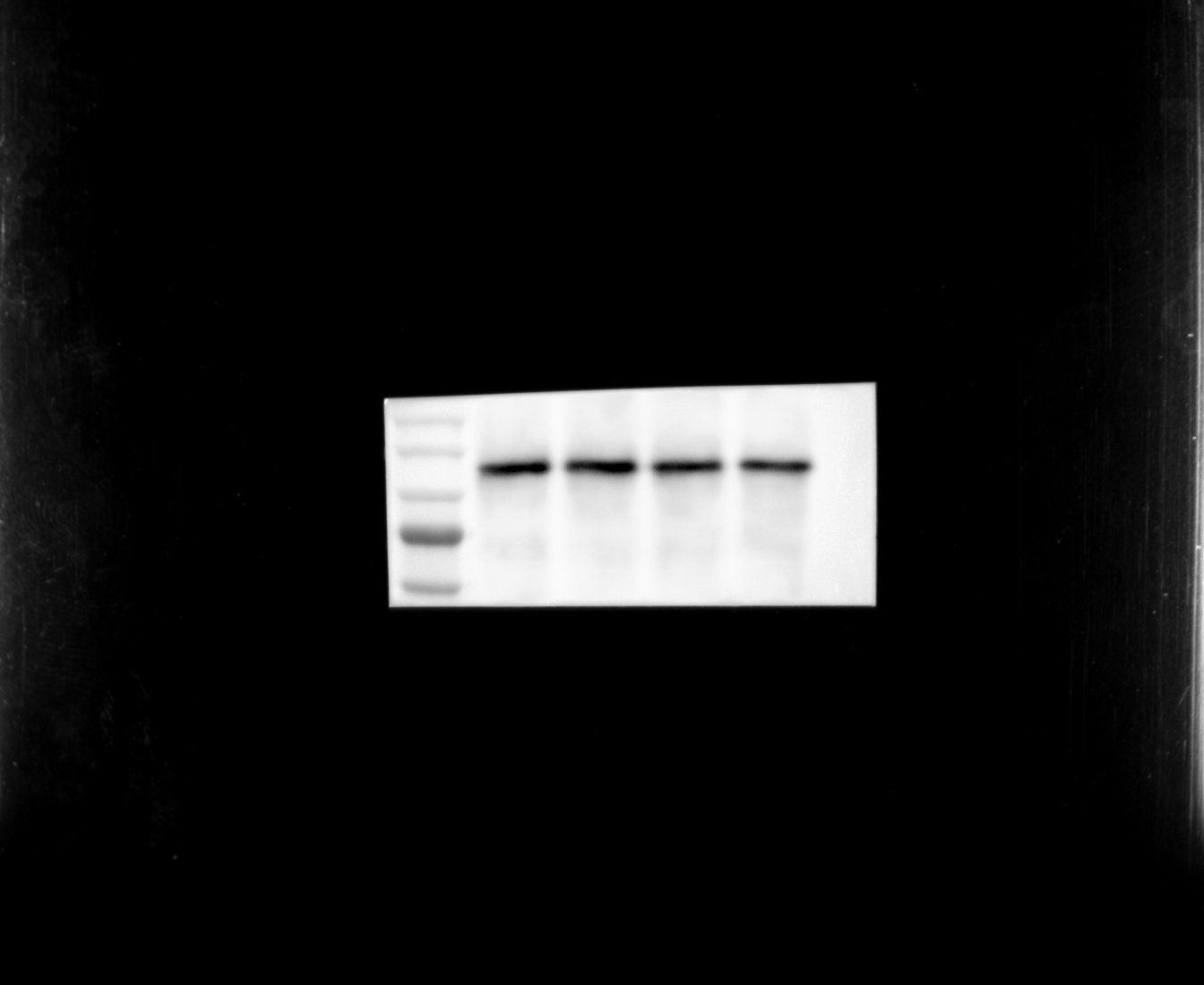

Supplement: Supplementary file 1 [file cimb-47-00936-s001.zip › cimb-3956315-supplementary/APOC2_ccRCC_RawWB_FullMembranes/cropped display images/7/Fig 3E jak3/1.Tif]

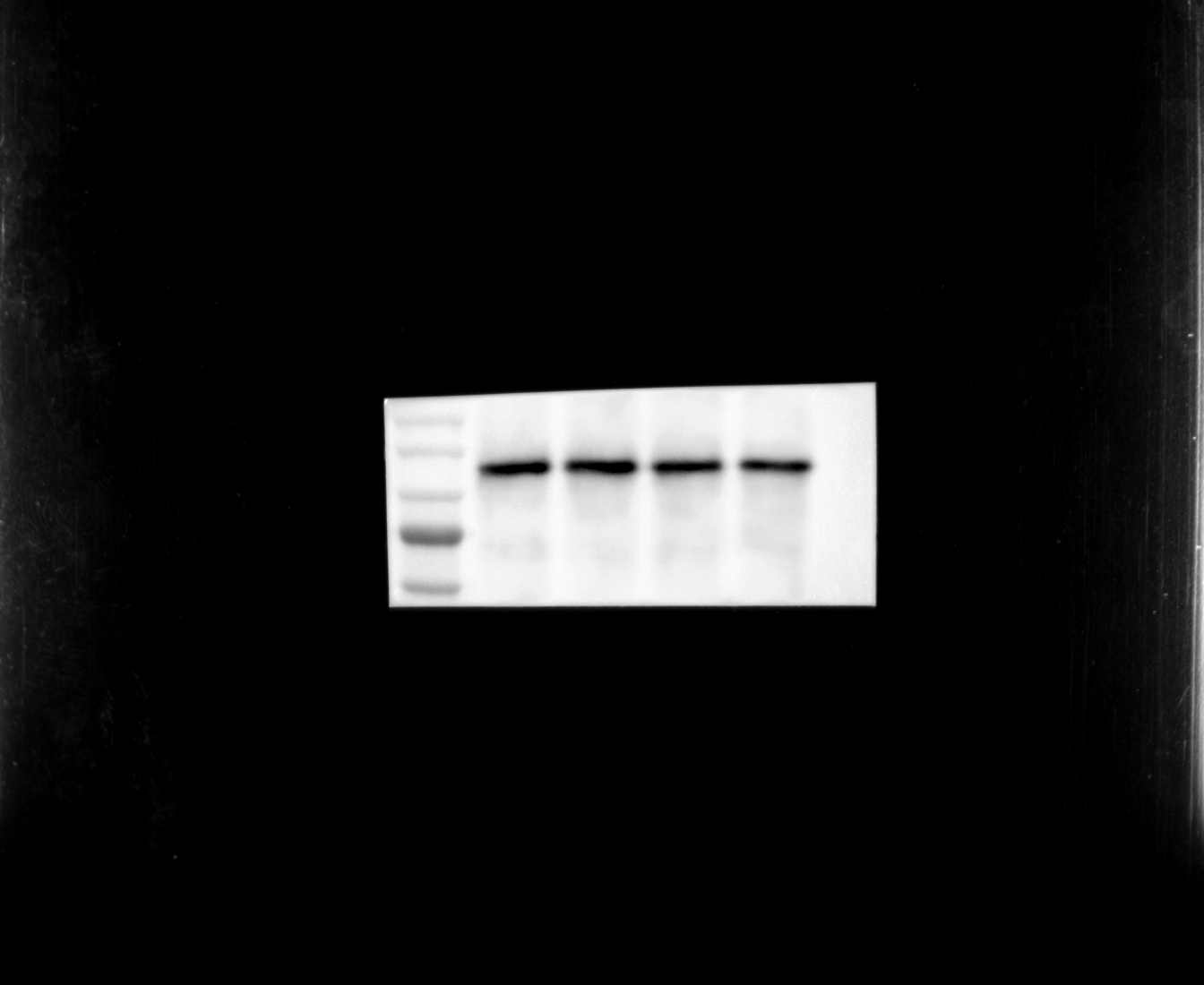

Supplement: Supplementary file 1 [file cimb-47-00936-s001.zip › cimb-3956315-supplementary/APOC2_ccRCC_RawWB_FullMembranes/cropped display images/7/Fig 3E jak3/2.Tif]

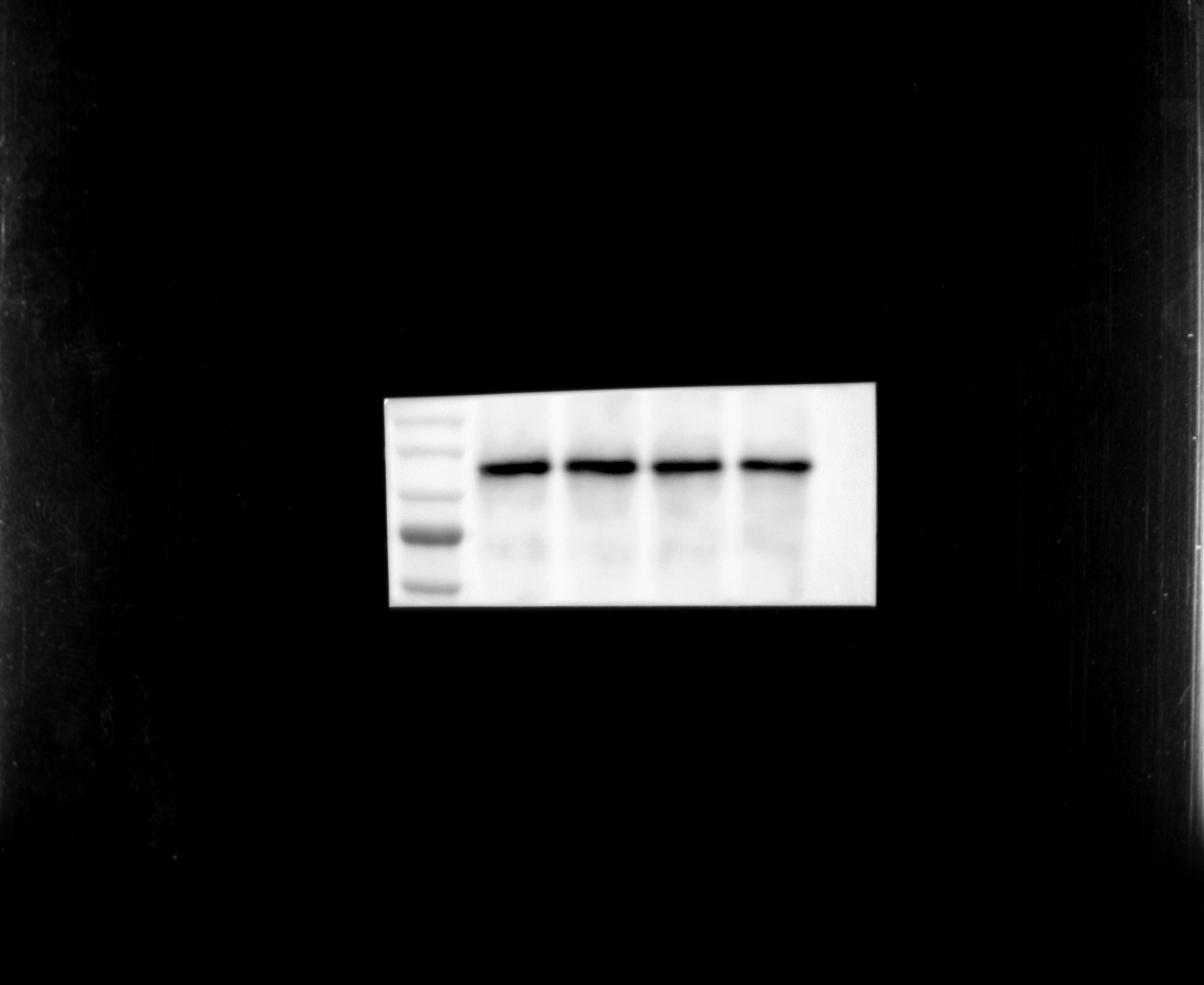

Supplement: Supplementary file 1 [file cimb-47-00936-s001.zip › cimb-3956315-supplementary/APOC2_ccRCC_RawWB_FullMembranes/cropped display images/7/Fig 3E jak3/3.Tif]

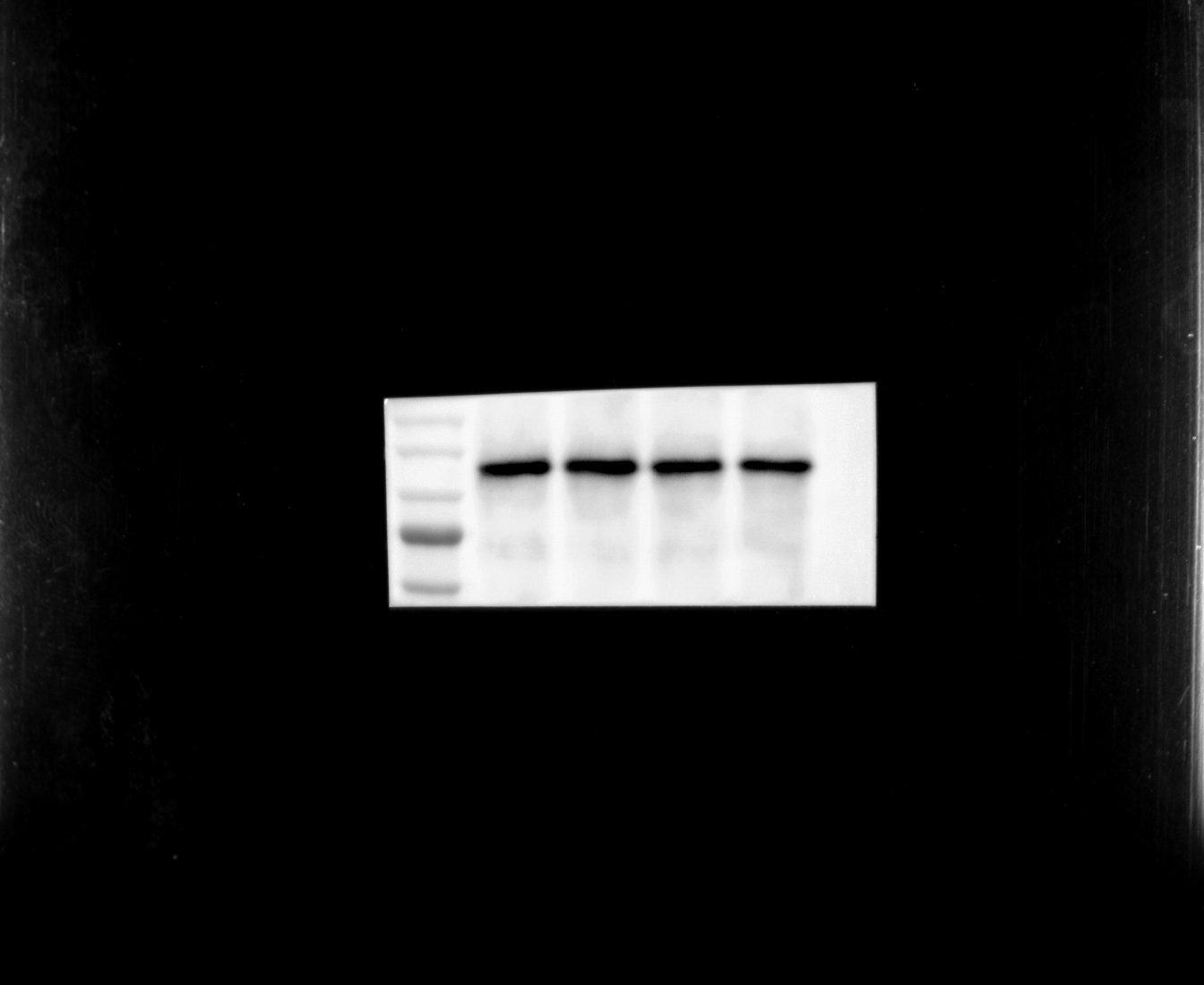

Supplement: Supplementary file 1 [file cimb-47-00936-s001.zip › cimb-3956315-supplementary/APOC2_ccRCC_RawWB_FullMembranes/cropped display images/7/Fig 3E jak3/4.Tif]

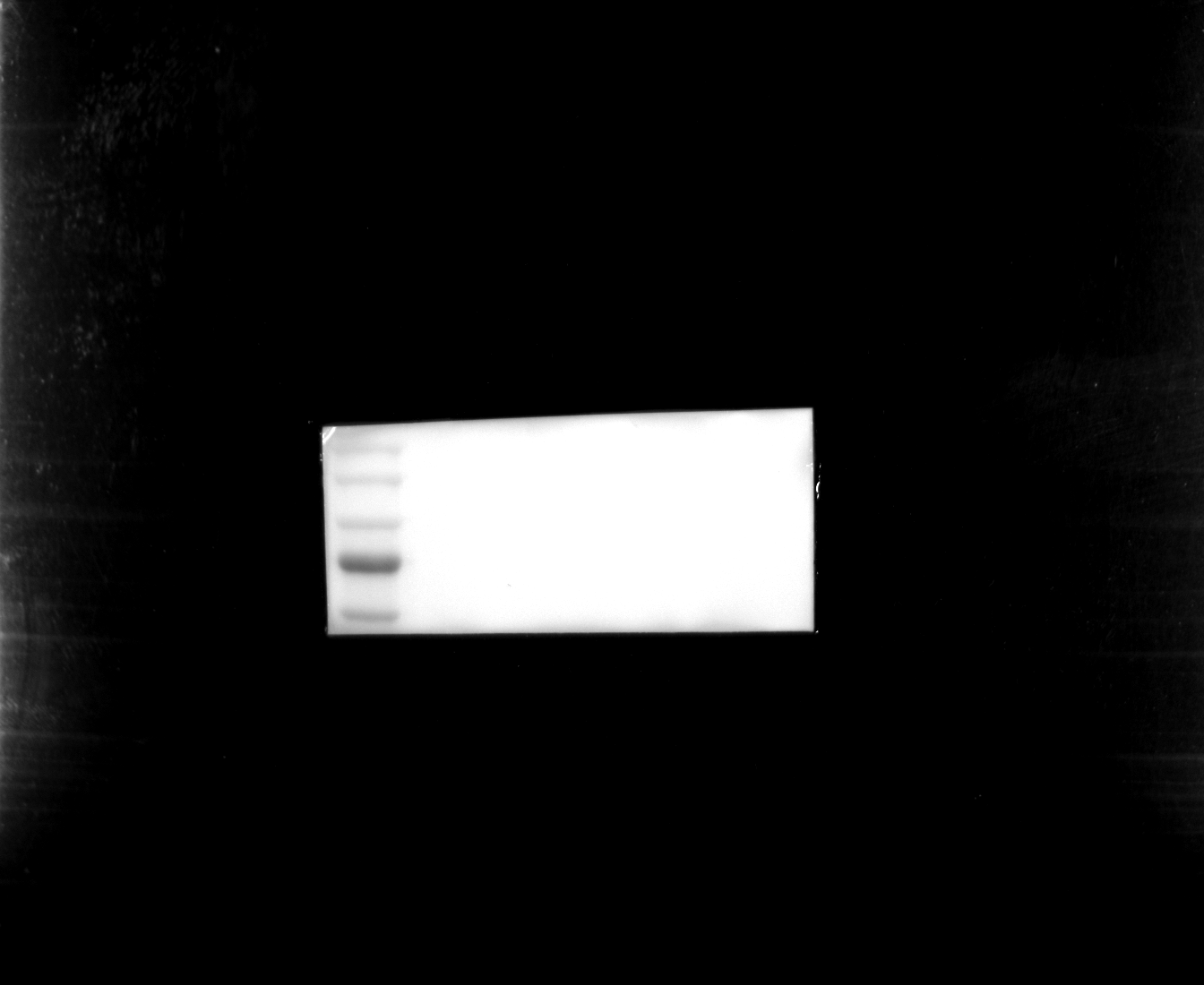

Supplement: Supplementary file 1 [file cimb-47-00936-s001.zip › cimb-3956315-supplementary/APOC2_ccRCC_RawWB_FullMembranes/cropped display images/7/Fig 3E p-jak3/0.Tif]

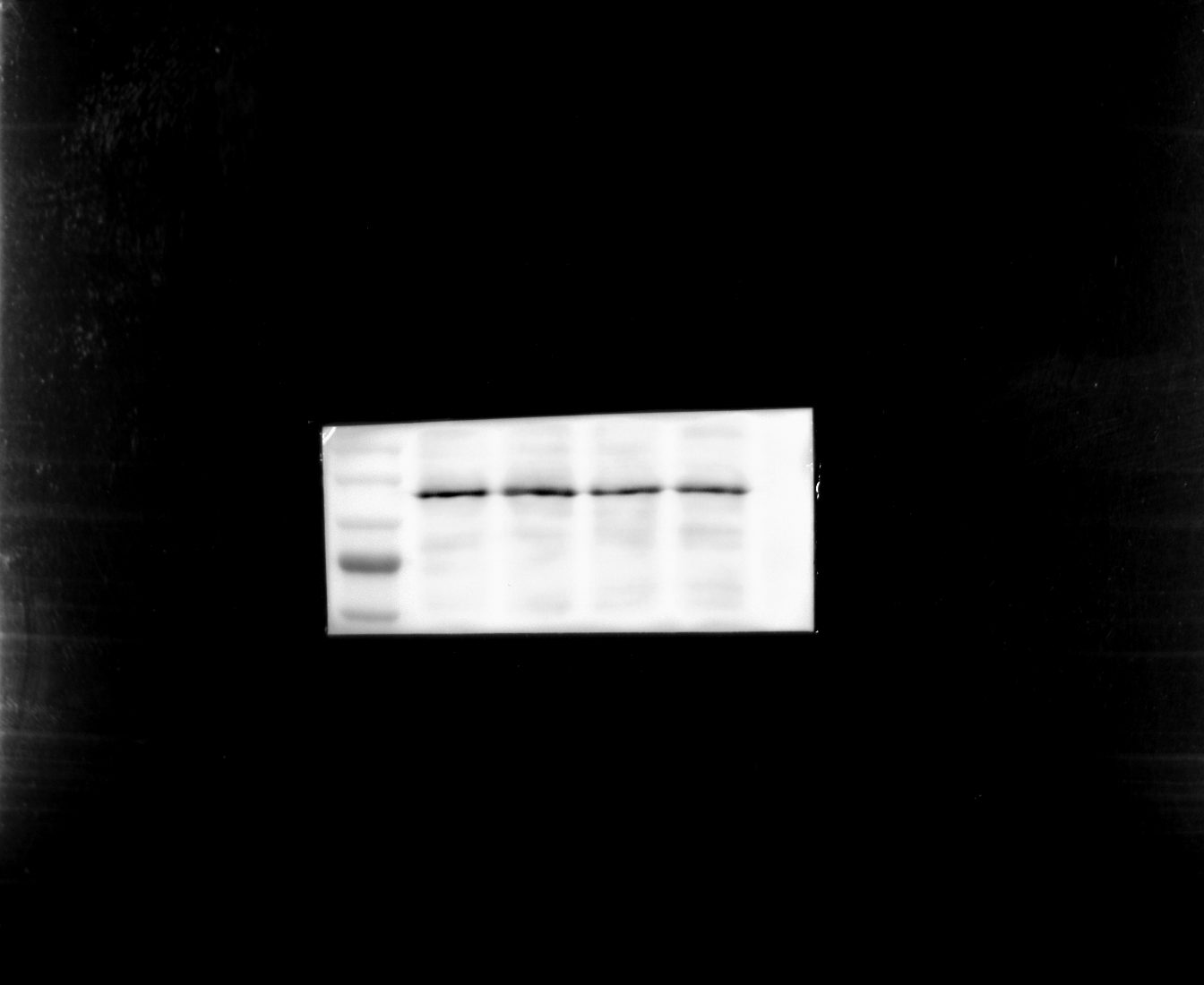

Supplement: Supplementary file 1 [file cimb-47-00936-s001.zip › cimb-3956315-supplementary/APOC2_ccRCC_RawWB_FullMembranes/cropped display images/7/Fig 3E p-jak3/1.Tif]

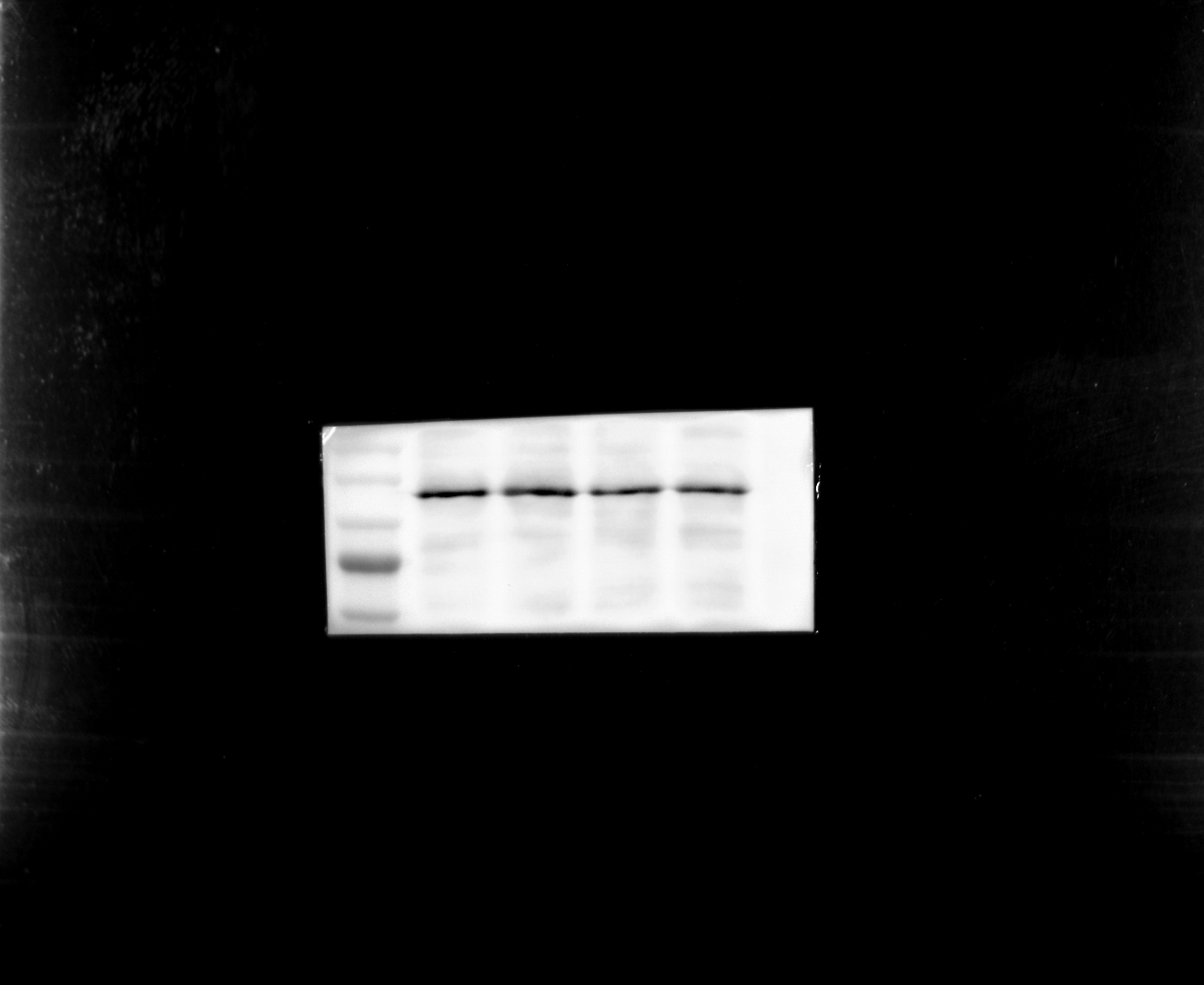

Supplement: Supplementary file 1 [file cimb-47-00936-s001.zip › cimb-3956315-supplementary/APOC2_ccRCC_RawWB_FullMembranes/cropped display images/7/Fig 3E p-jak3/2.Tif]

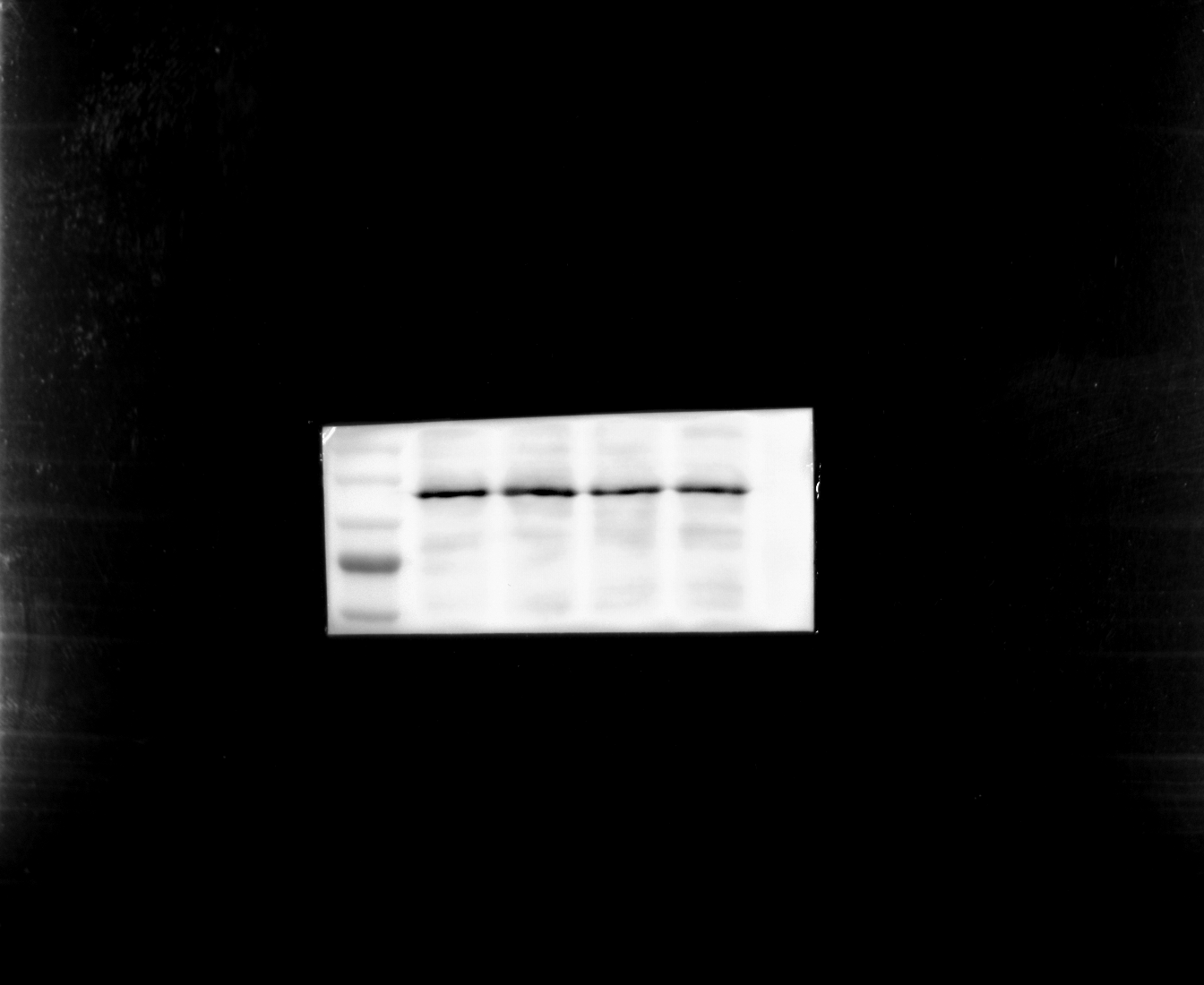

Supplement: Supplementary file 1 [file cimb-47-00936-s001.zip › cimb-3956315-supplementary/APOC2_ccRCC_RawWB_FullMembranes/cropped display images/7/Fig 3E p-jak3/3.Tif]

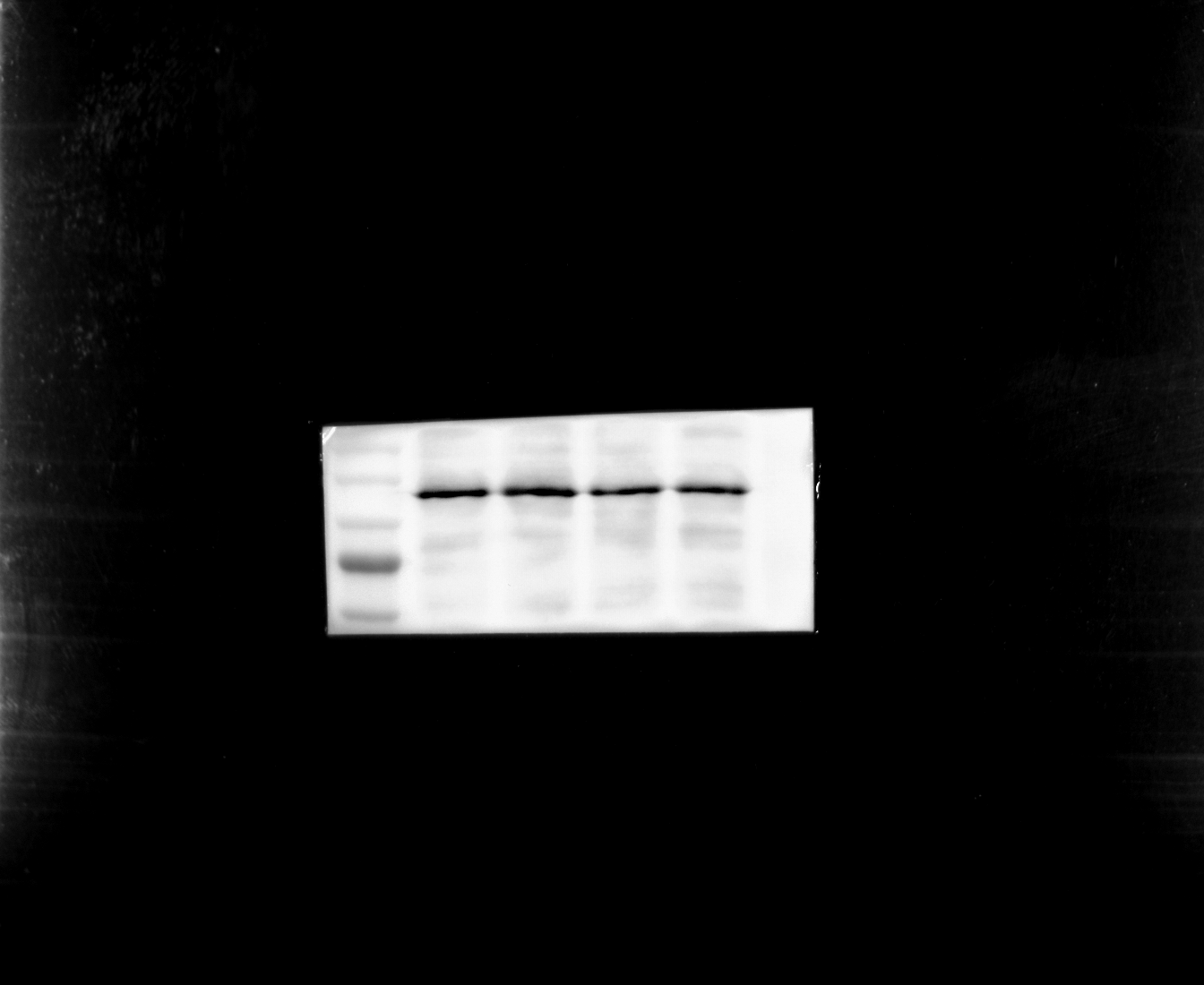

Supplement: Supplementary file 1 [file cimb-47-00936-s001.zip › cimb-3956315-supplementary/APOC2_ccRCC_RawWB_FullMembranes/cropped display images/7/Fig 3E p-jak3/4.Tif]

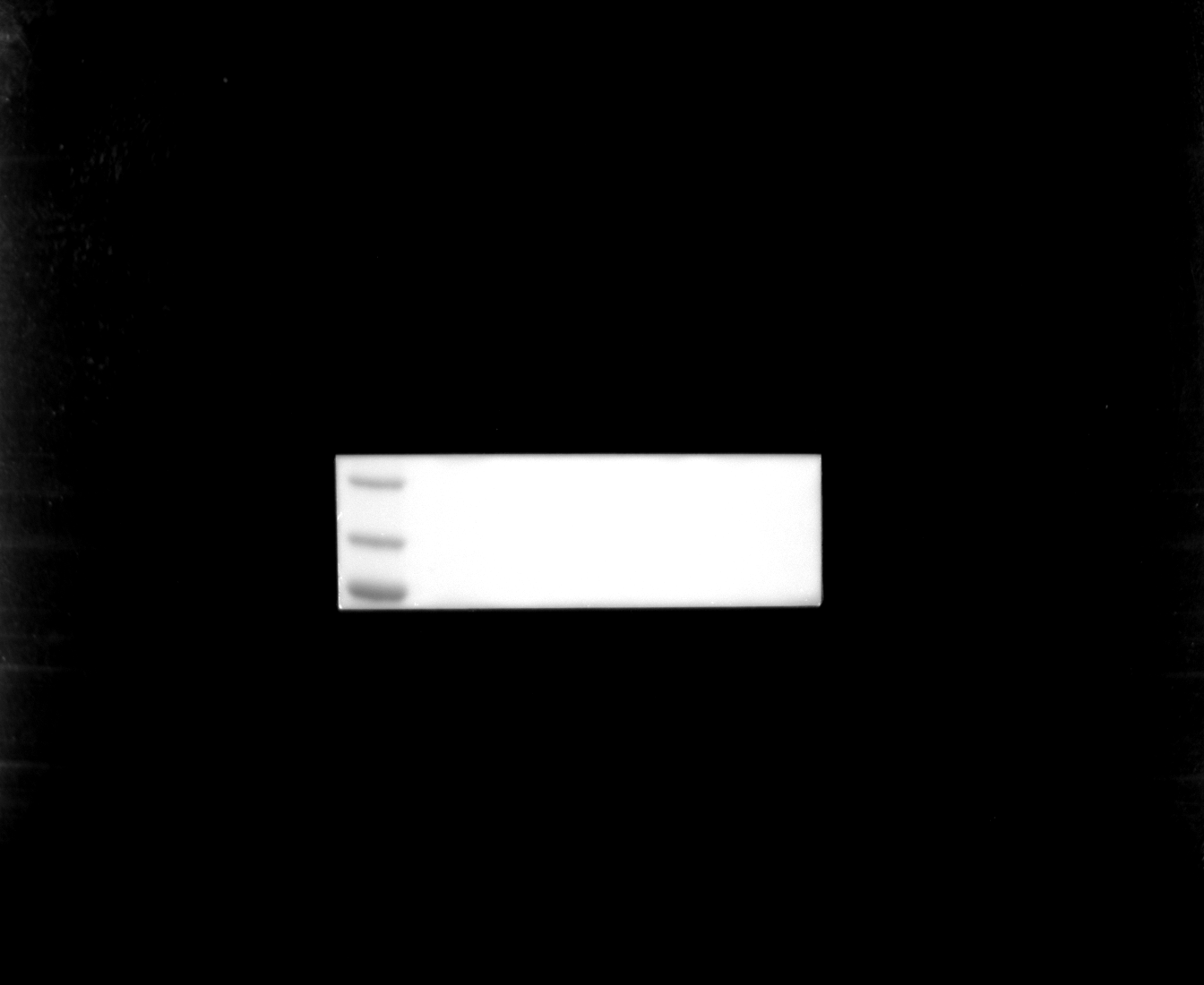

Supplement: Supplementary file 1 [file cimb-47-00936-s001.zip › cimb-3956315-supplementary/APOC2_ccRCC_RawWB_FullMembranes/cropped display images/7/β- actin/0.Tif]

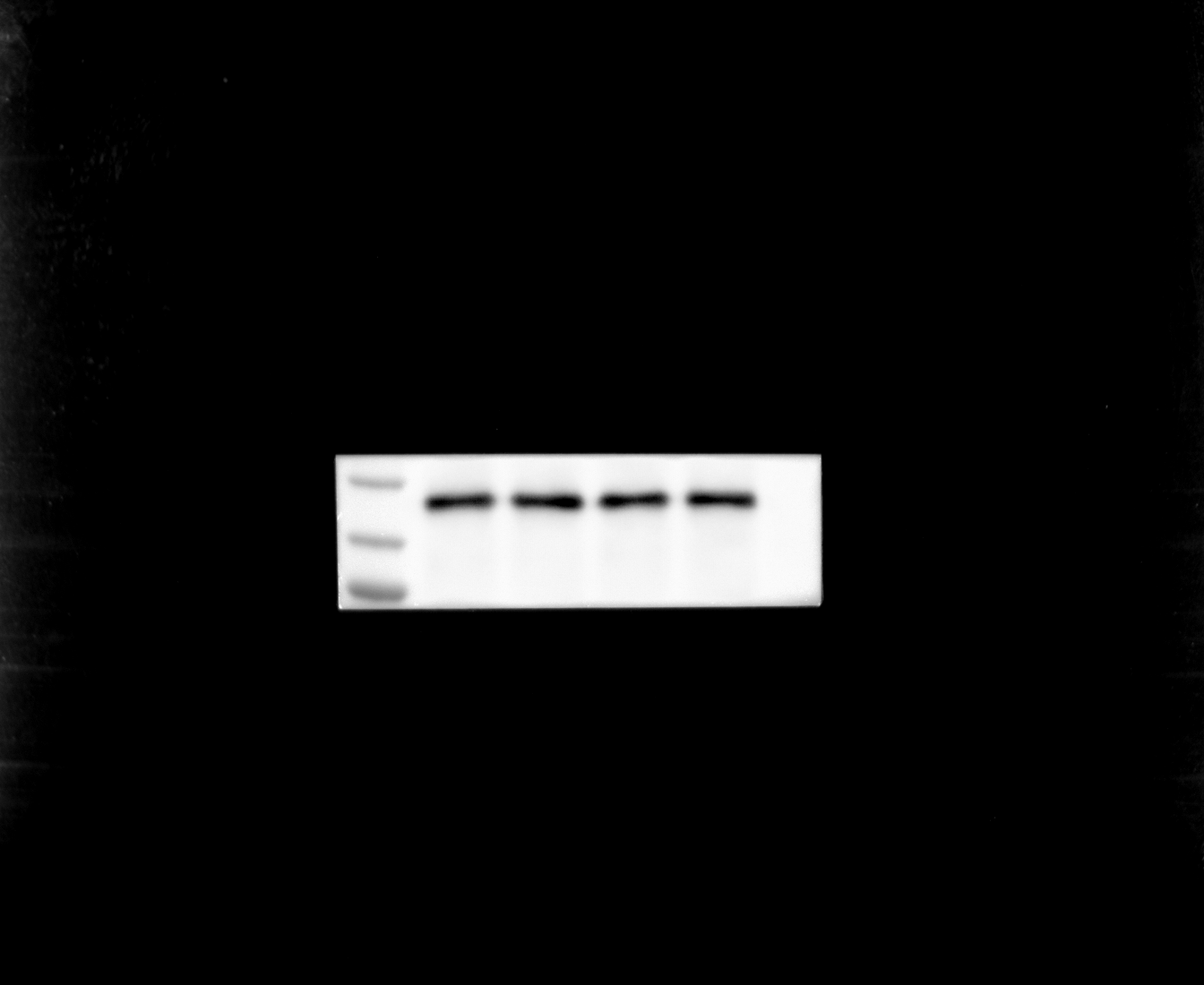

Supplement: Supplementary file 1 [file cimb-47-00936-s001.zip › cimb-3956315-supplementary/APOC2_ccRCC_RawWB_FullMembranes/cropped display images/7/β- actin/1.Tif]

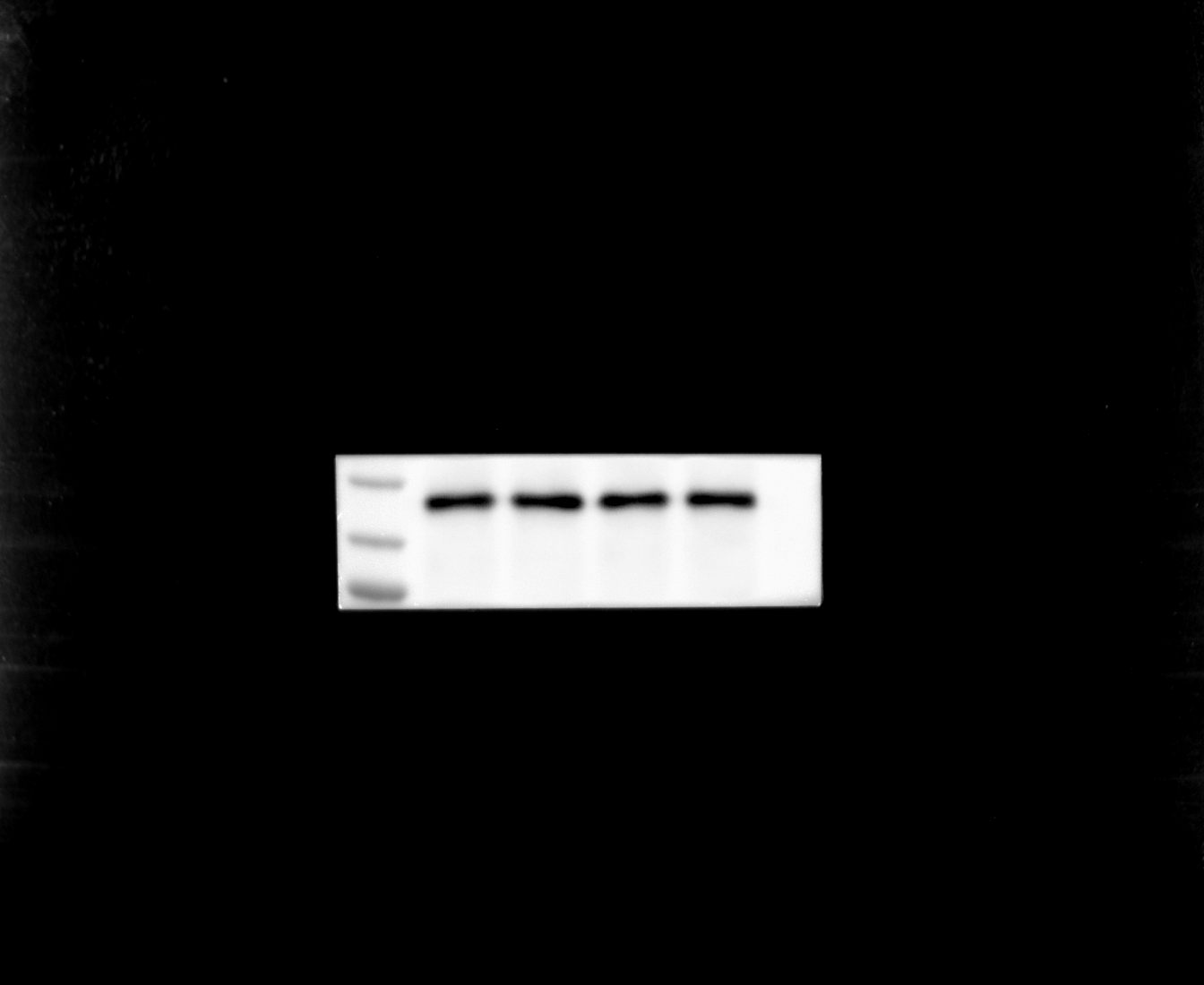

Supplement: Supplementary file 1 [file cimb-47-00936-s001.zip › cimb-3956315-supplementary/APOC2_ccRCC_RawWB_FullMembranes/cropped display images/7/β- actin/2.Tif]

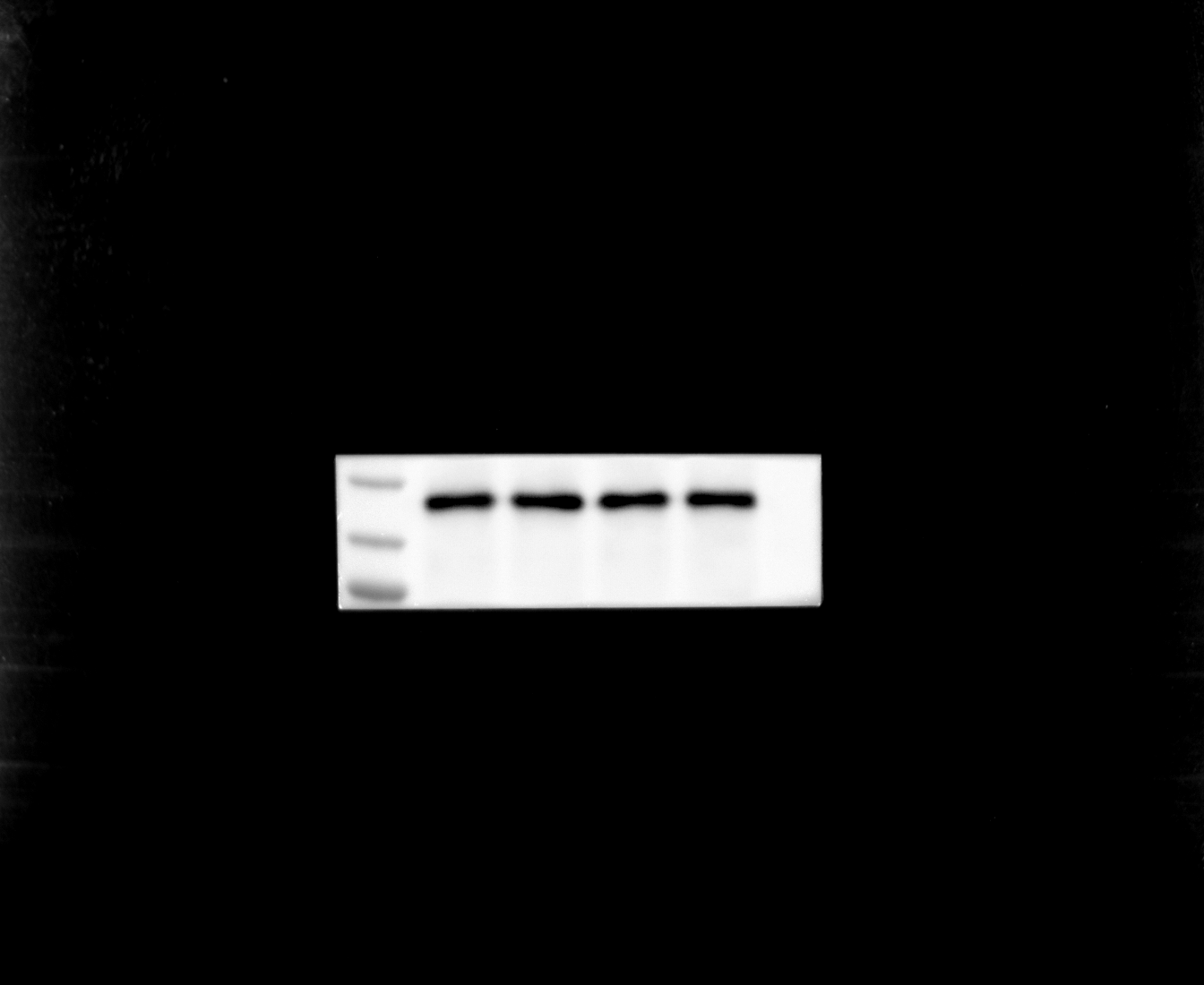

Supplement: Supplementary file 1 [file cimb-47-00936-s001.zip › cimb-3956315-supplementary/APOC2_ccRCC_RawWB_FullMembranes/cropped display images/7/β- actin/3.Tif]

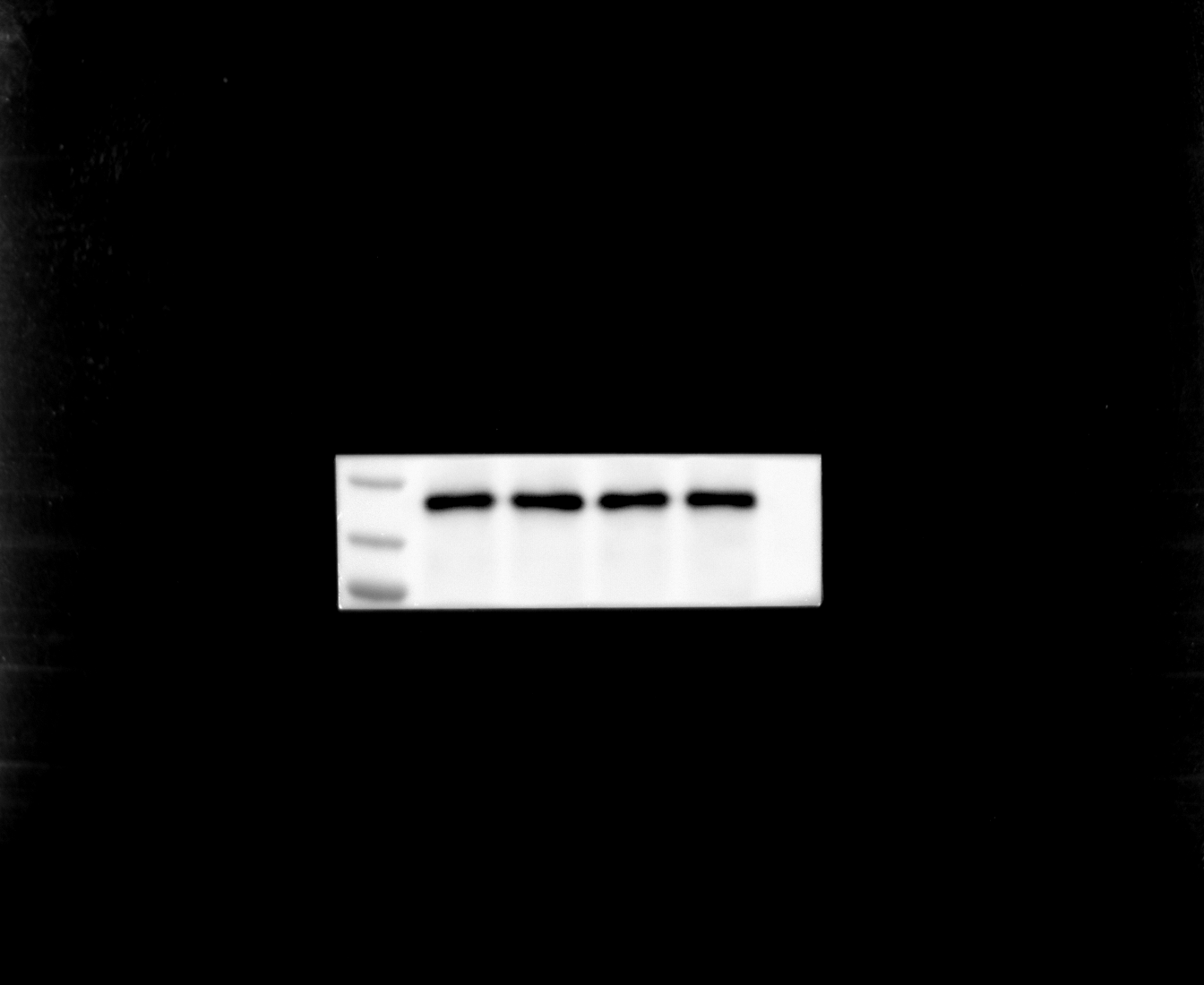

Supplement: Supplementary file 1 [file cimb-47-00936-s001.zip › cimb-3956315-supplementary/APOC2_ccRCC_RawWB_FullMembranes/cropped display images/7/β- actin/4.Tif]

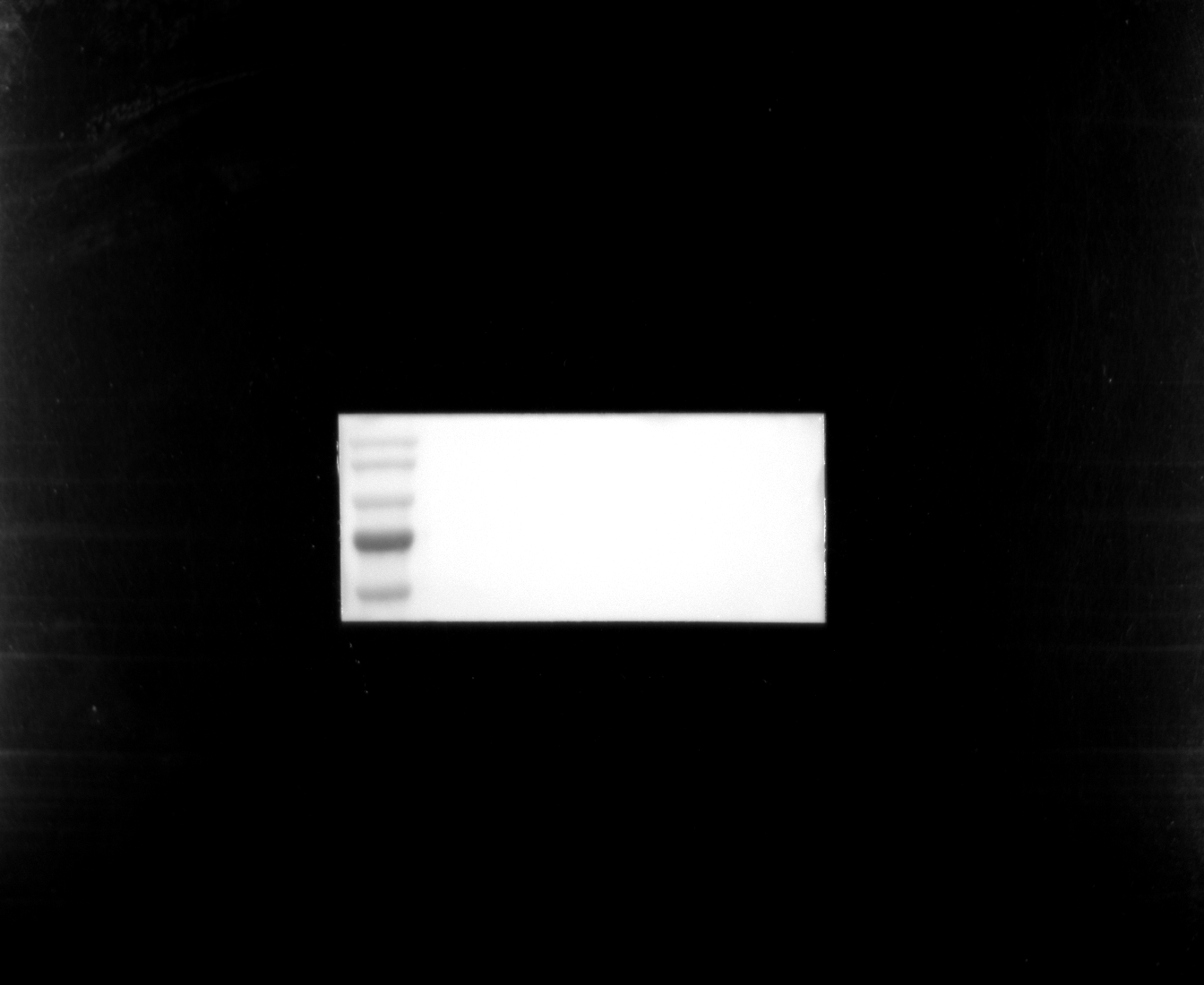

Supplement: Supplementary file 1 [file cimb-47-00936-s001.zip › cimb-3956315-supplementary/APOC2_ccRCC_RawWB_FullMembranes/cropped display images/8/Fig 3E p-stat1/0.Tif]

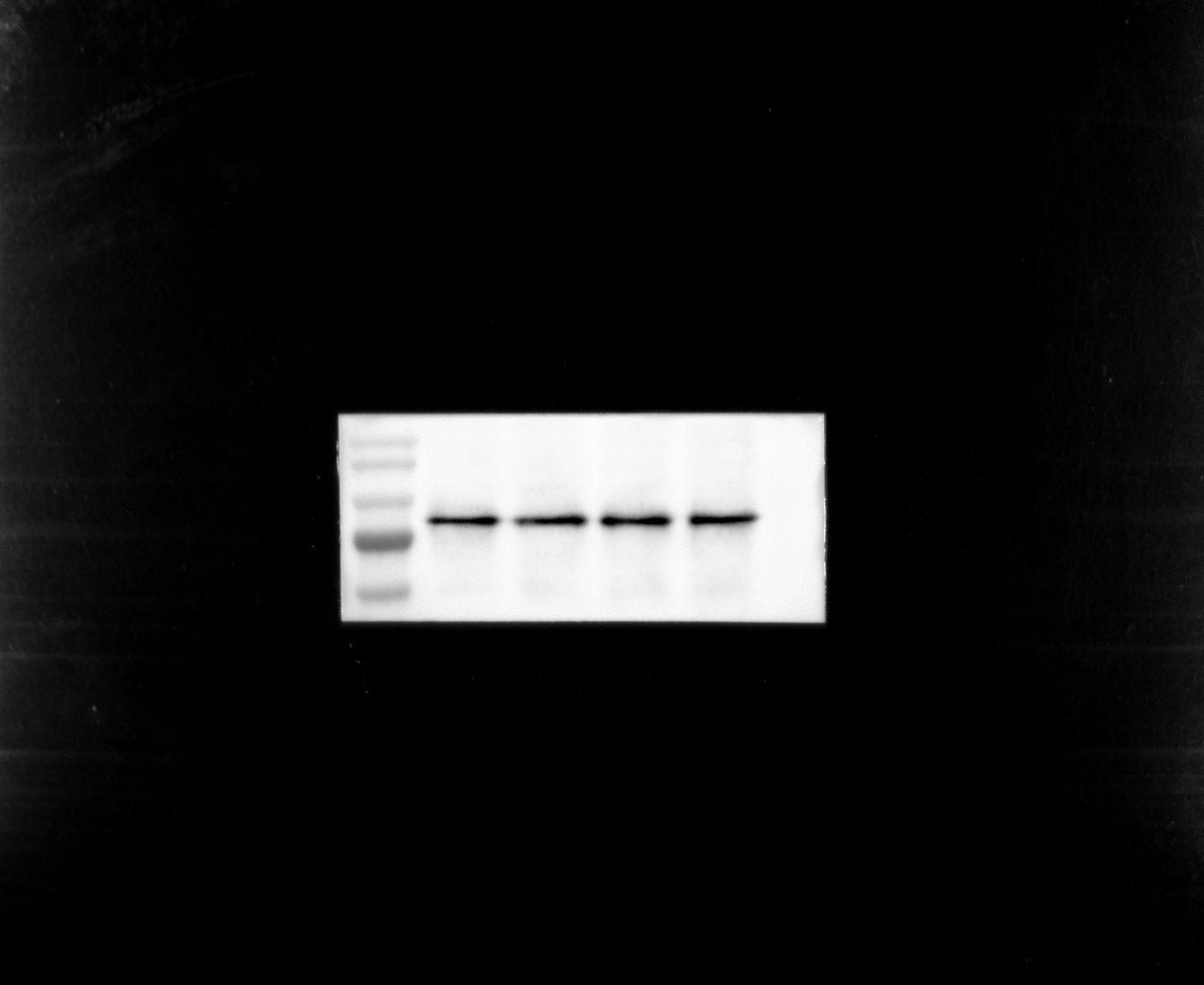

Supplement: Supplementary file 1 [file cimb-47-00936-s001.zip › cimb-3956315-supplementary/APOC2_ccRCC_RawWB_FullMembranes/cropped display images/8/Fig 3E p-stat1/1.Tif]

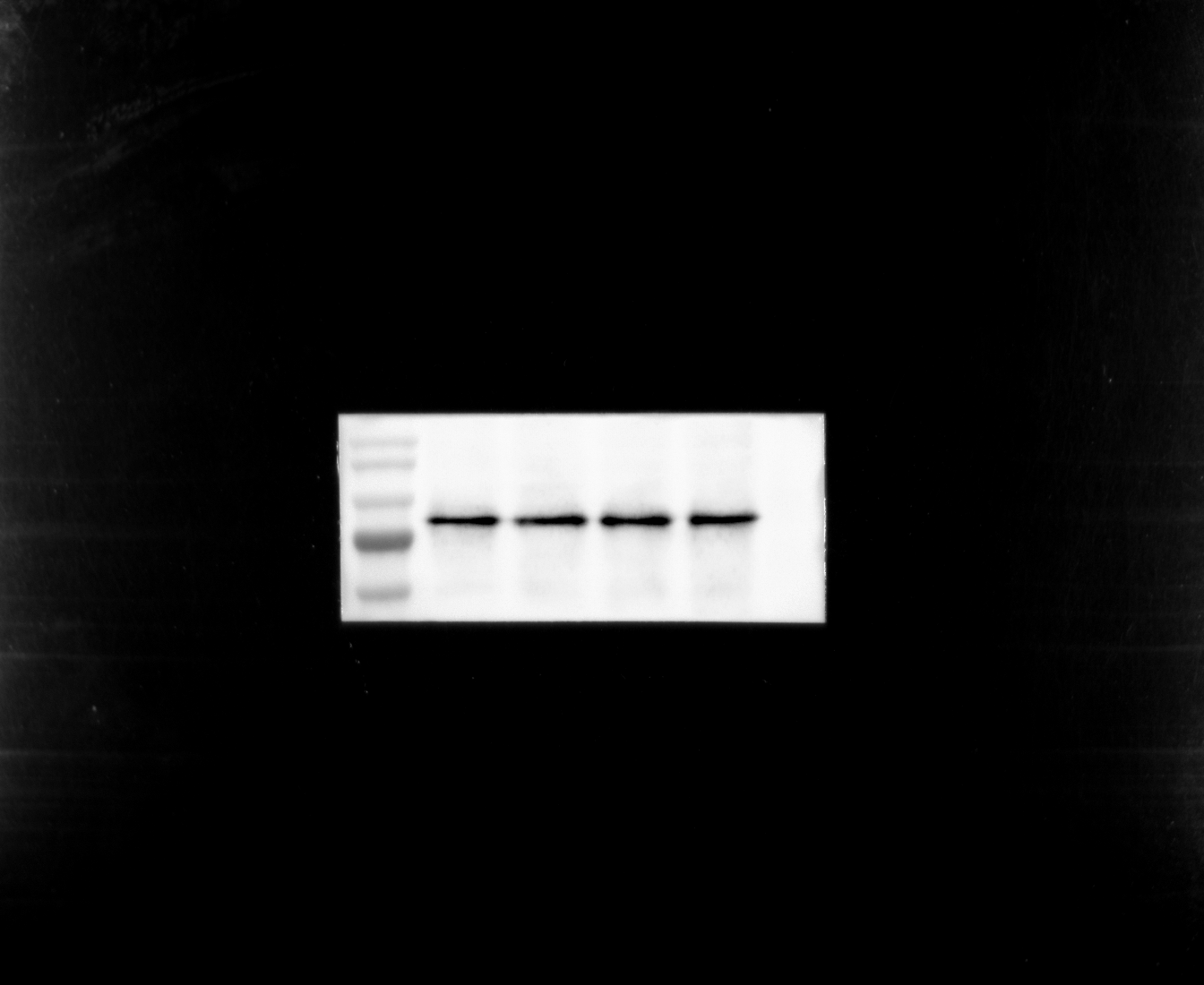

Supplement: Supplementary file 1 [file cimb-47-00936-s001.zip › cimb-3956315-supplementary/APOC2_ccRCC_RawWB_FullMembranes/cropped display images/8/Fig 3E p-stat1/2.Tif]

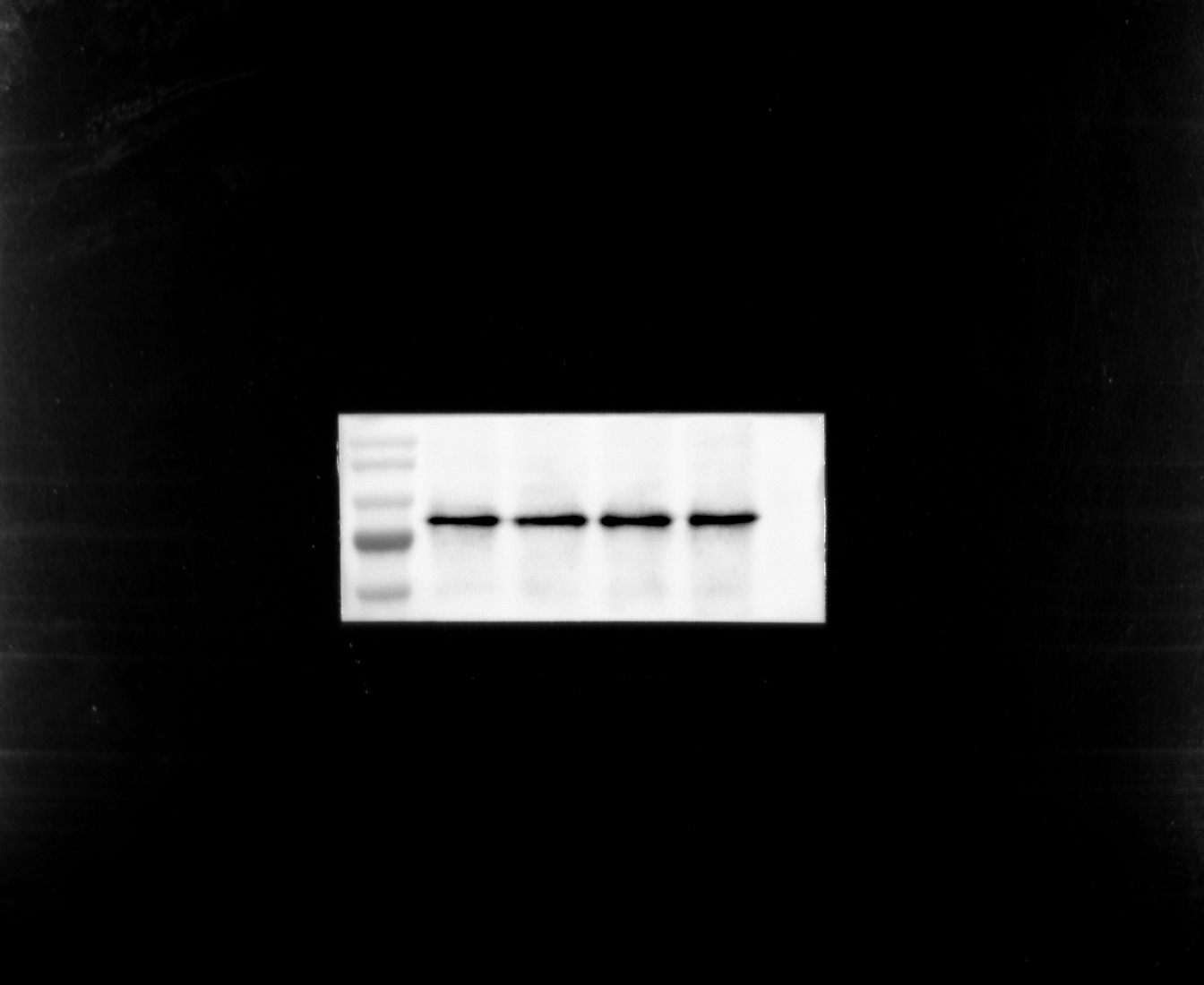

Supplement: Supplementary file 1 [file cimb-47-00936-s001.zip › cimb-3956315-supplementary/APOC2_ccRCC_RawWB_FullMembranes/cropped display images/8/Fig 3E p-stat1/3.Tif]

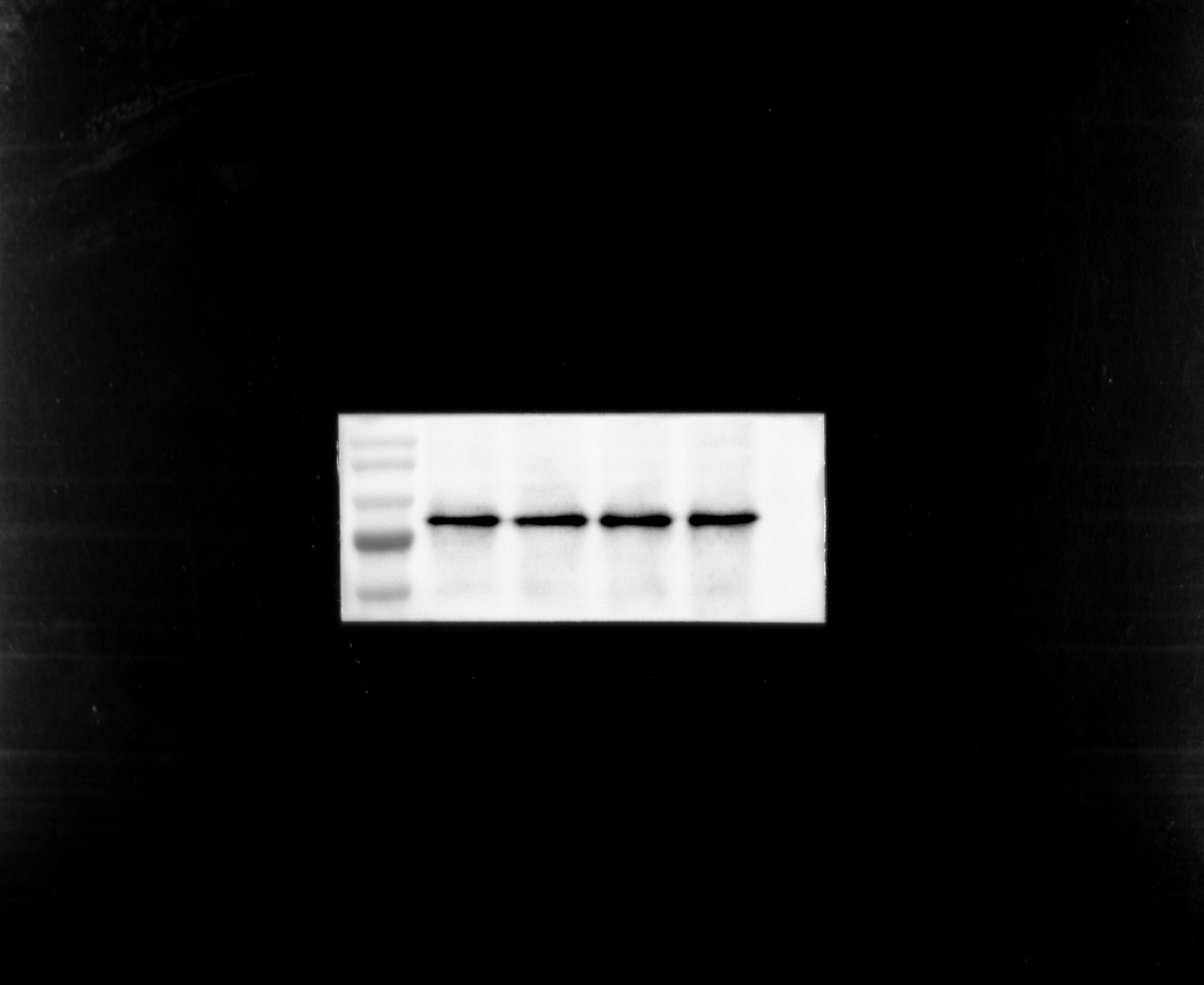

Supplement: Supplementary file 1 [file cimb-47-00936-s001.zip › cimb-3956315-supplementary/APOC2_ccRCC_RawWB_FullMembranes/cropped display images/8/Fig 3E p-stat1/4.Tif]

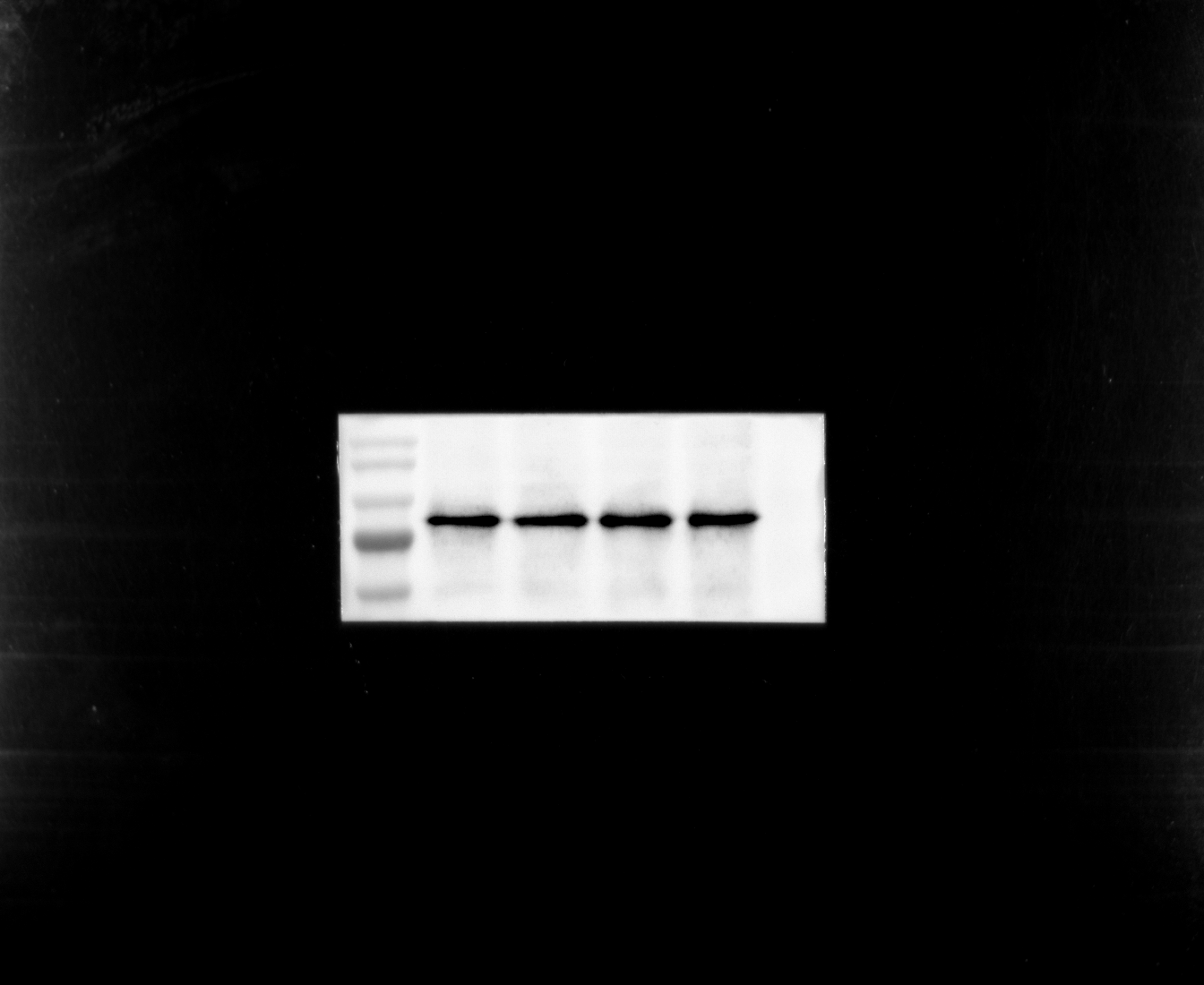

Supplement: Supplementary file 1 [file cimb-47-00936-s001.zip › cimb-3956315-supplementary/APOC2_ccRCC_RawWB_FullMembranes/cropped display images/8/Fig 3E p-stat1/5.Tif]

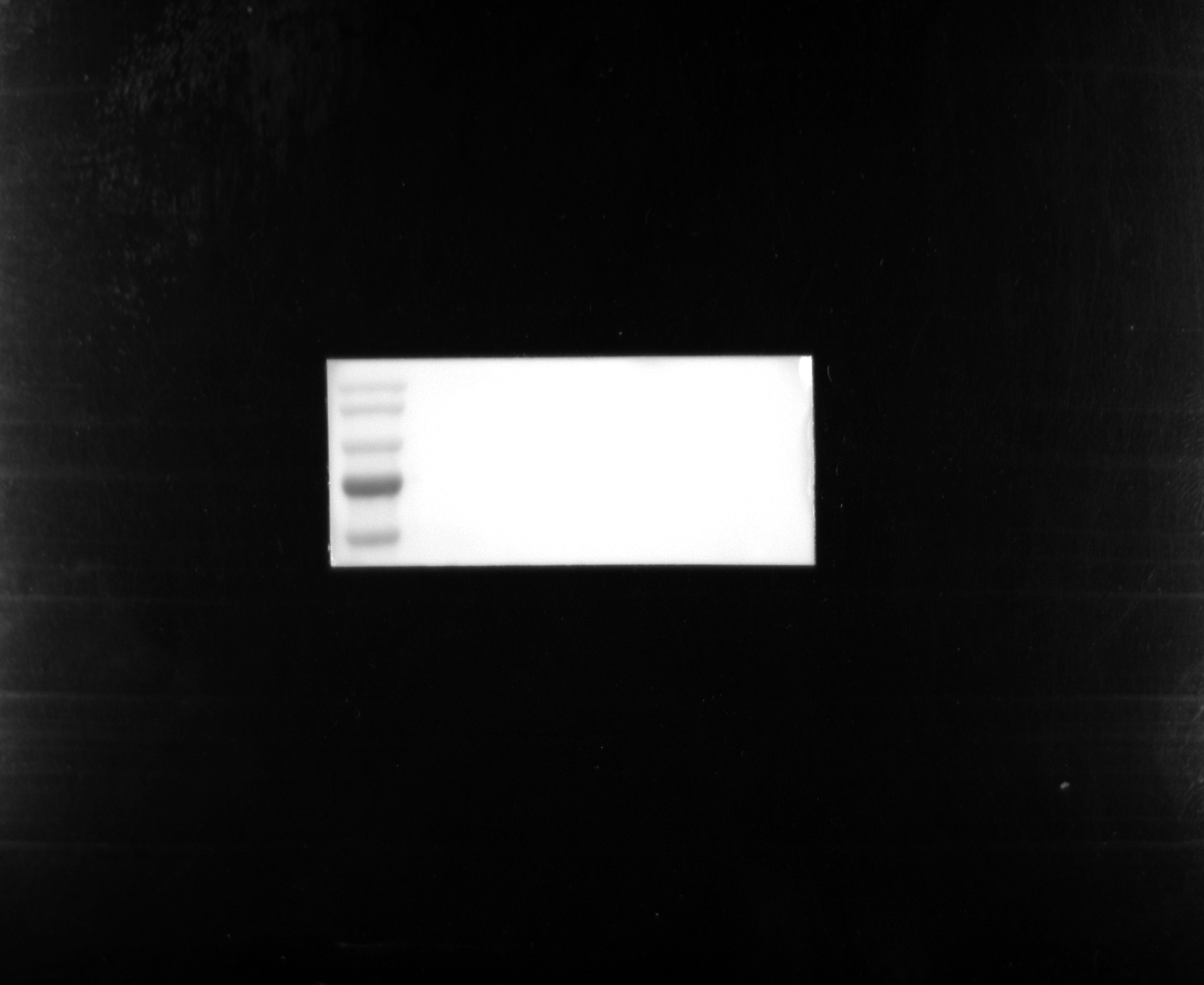

Supplement: Supplementary file 1 [file cimb-47-00936-s001.zip › cimb-3956315-supplementary/APOC2_ccRCC_RawWB_FullMembranes/cropped display images/8/Fig 3E stat1/0.Tif]

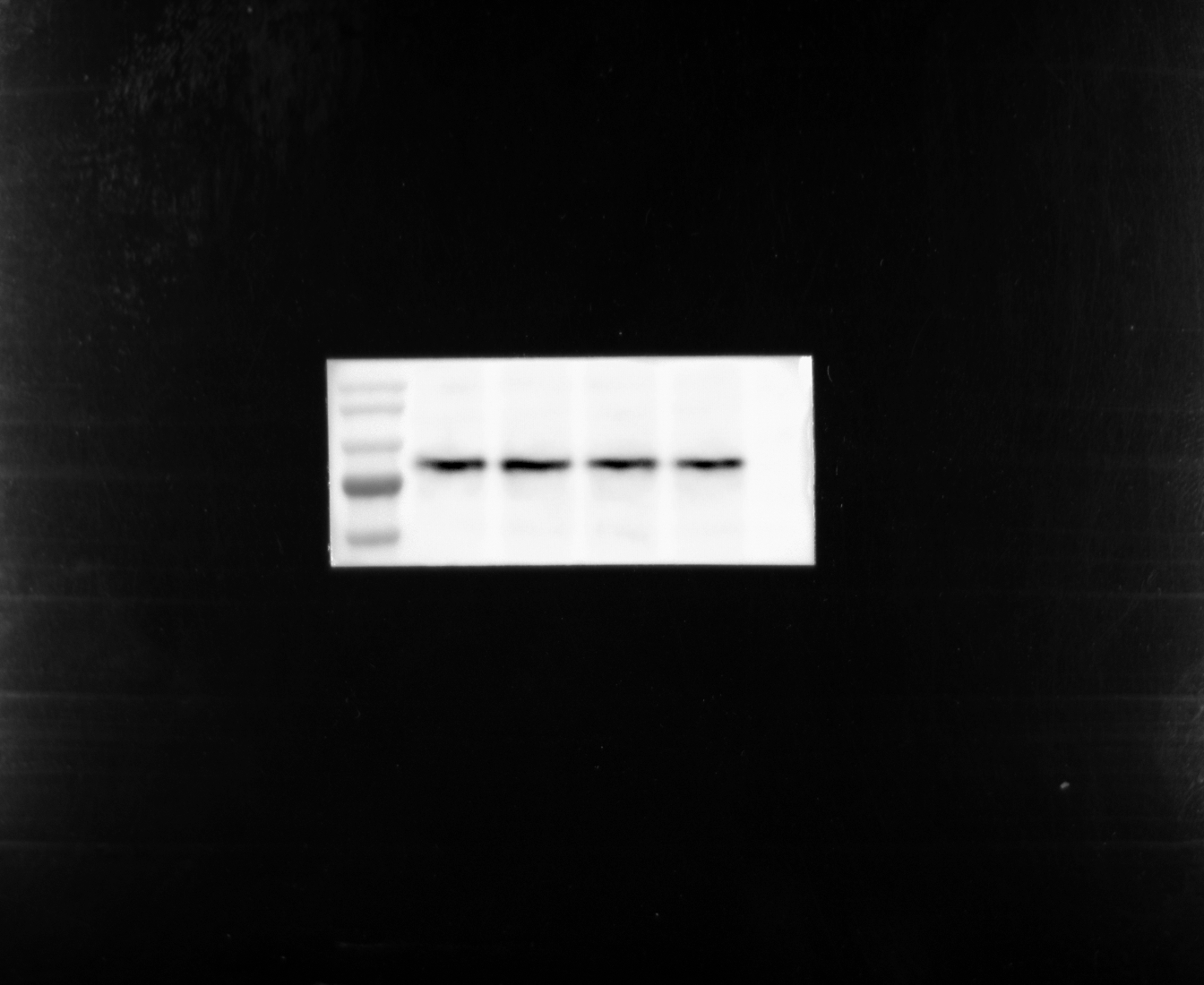

Supplement: Supplementary file 1 [file cimb-47-00936-s001.zip › cimb-3956315-supplementary/APOC2_ccRCC_RawWB_FullMembranes/cropped display images/8/Fig 3E stat1/1.Tif]

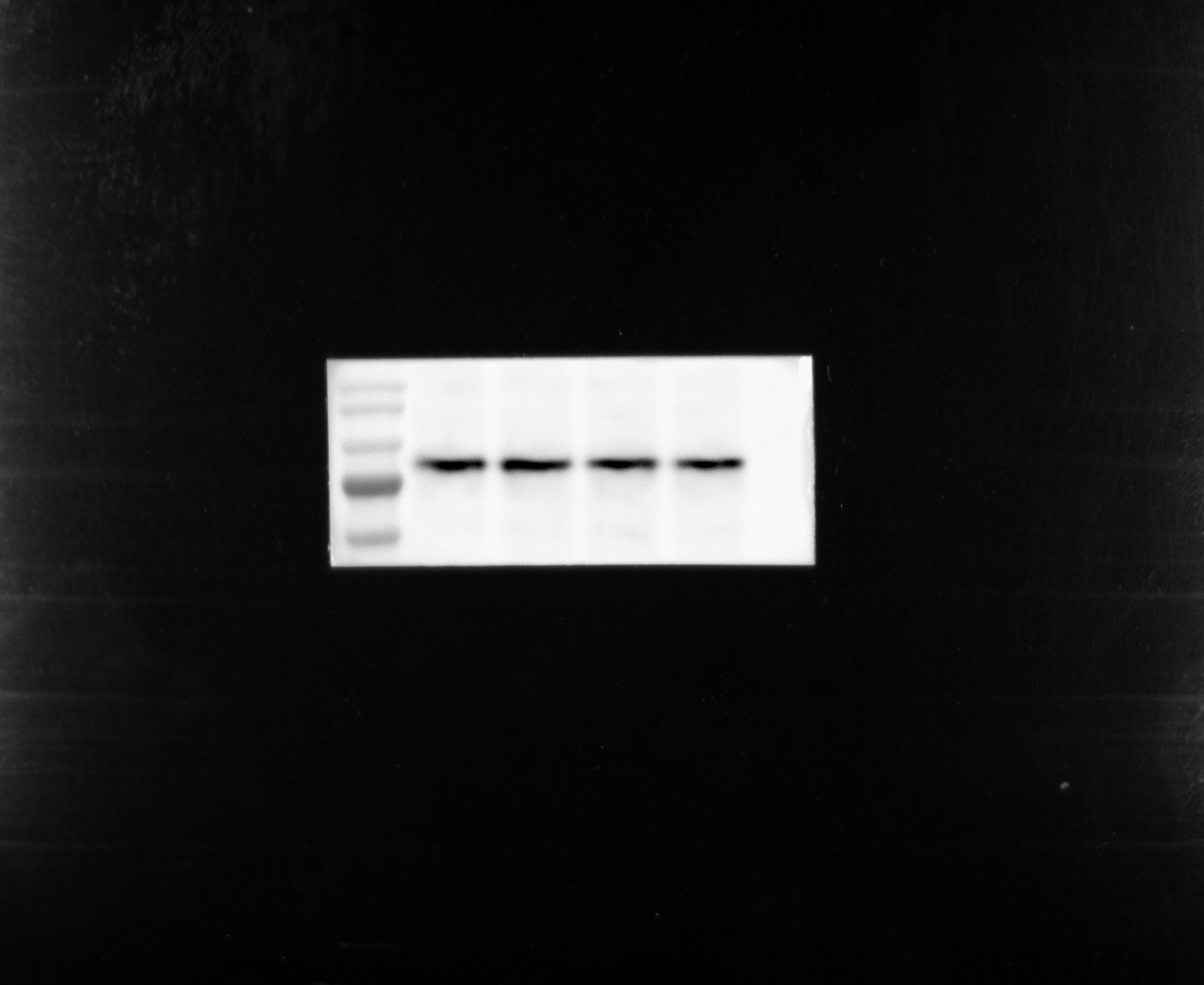

Supplement: Supplementary file 1 [file cimb-47-00936-s001.zip › cimb-3956315-supplementary/APOC2_ccRCC_RawWB_FullMembranes/cropped display images/8/Fig 3E stat1/2.Tif]

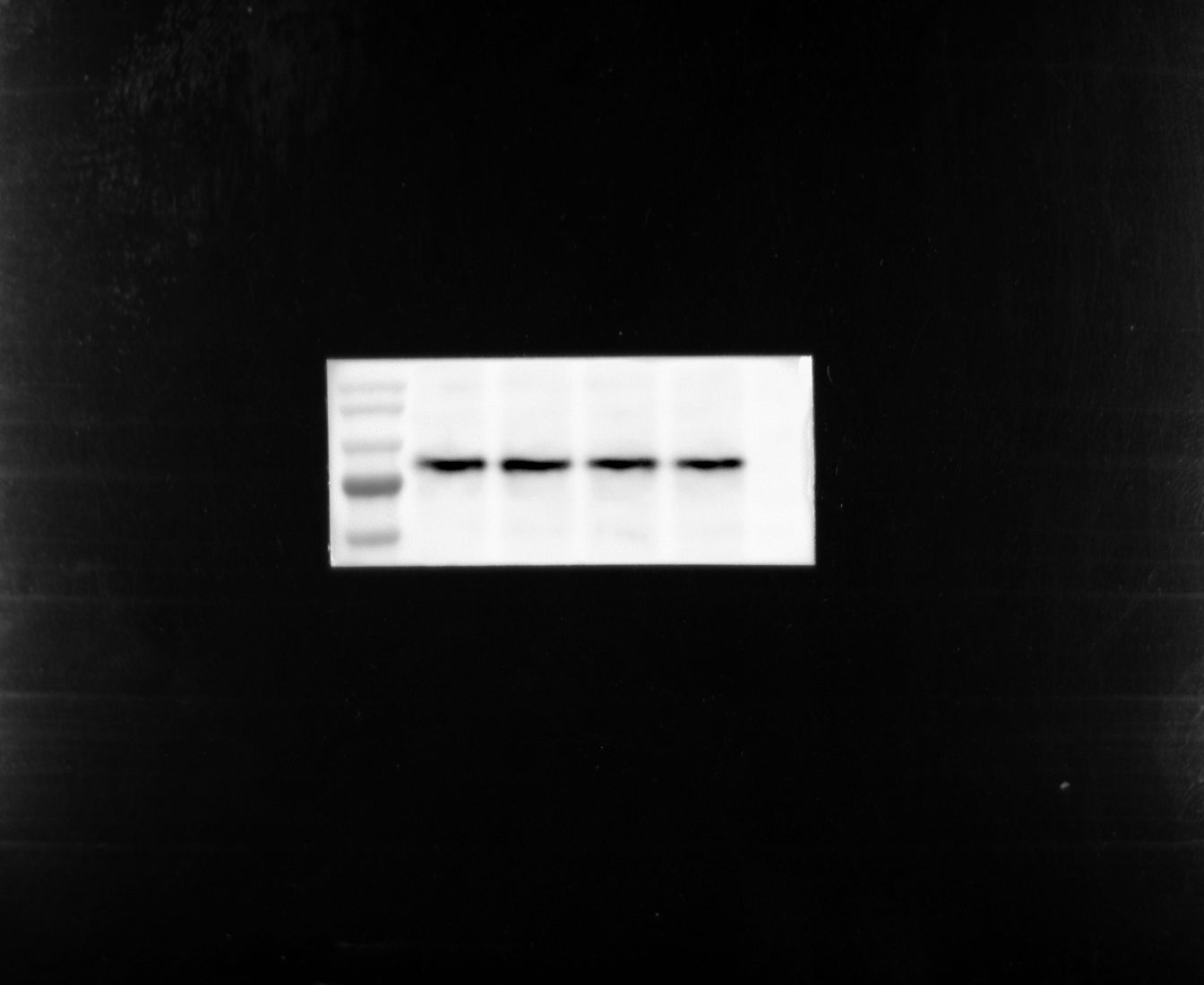

Supplement: Supplementary file 1 [file cimb-47-00936-s001.zip › cimb-3956315-supplementary/APOC2_ccRCC_RawWB_FullMembranes/cropped display images/8/Fig 3E stat1/3.Tif]

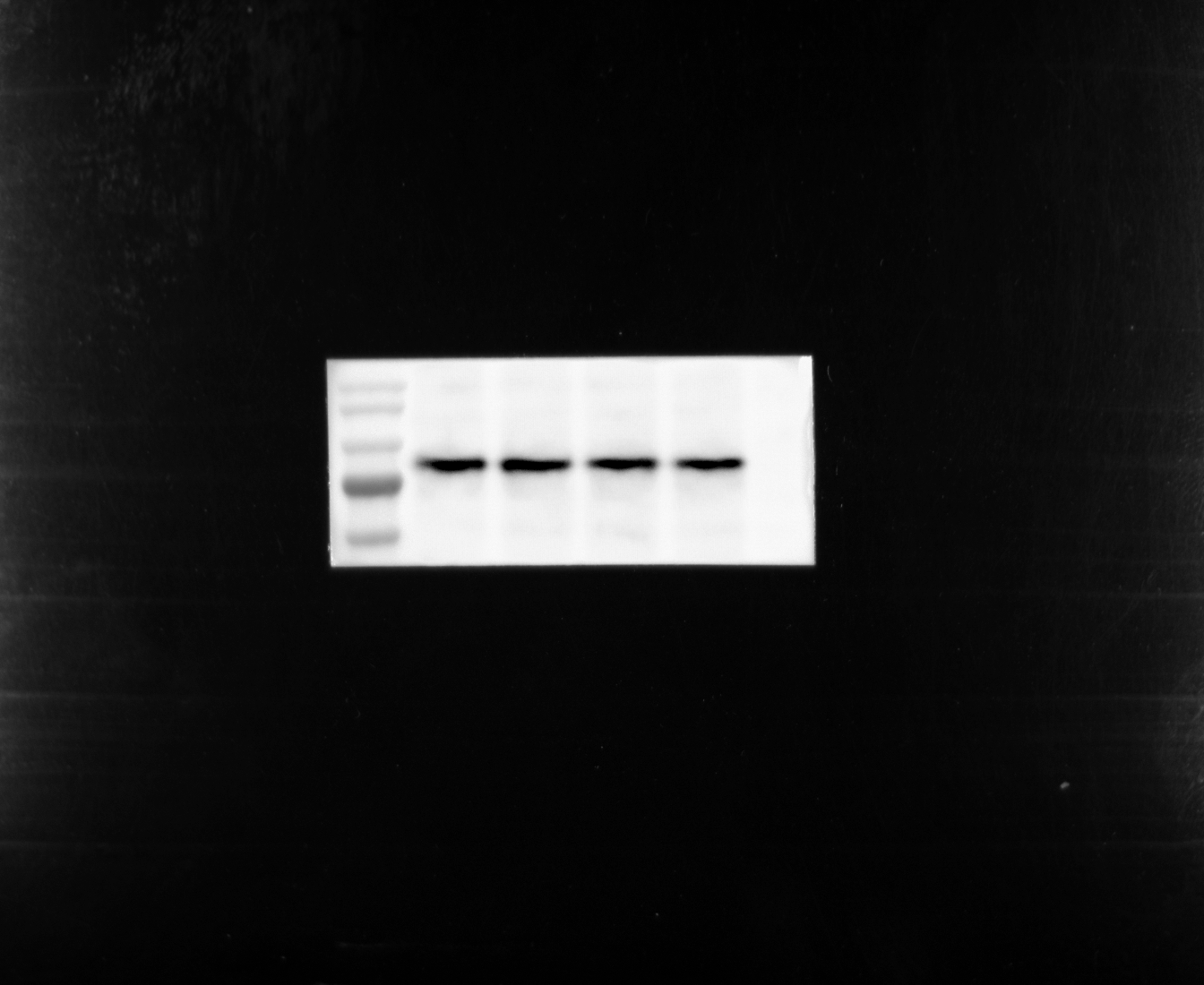

Supplement: Supplementary file 1 [file cimb-47-00936-s001.zip › cimb-3956315-supplementary/APOC2_ccRCC_RawWB_FullMembranes/cropped display images/8/Fig 3E stat1/4.Tif]

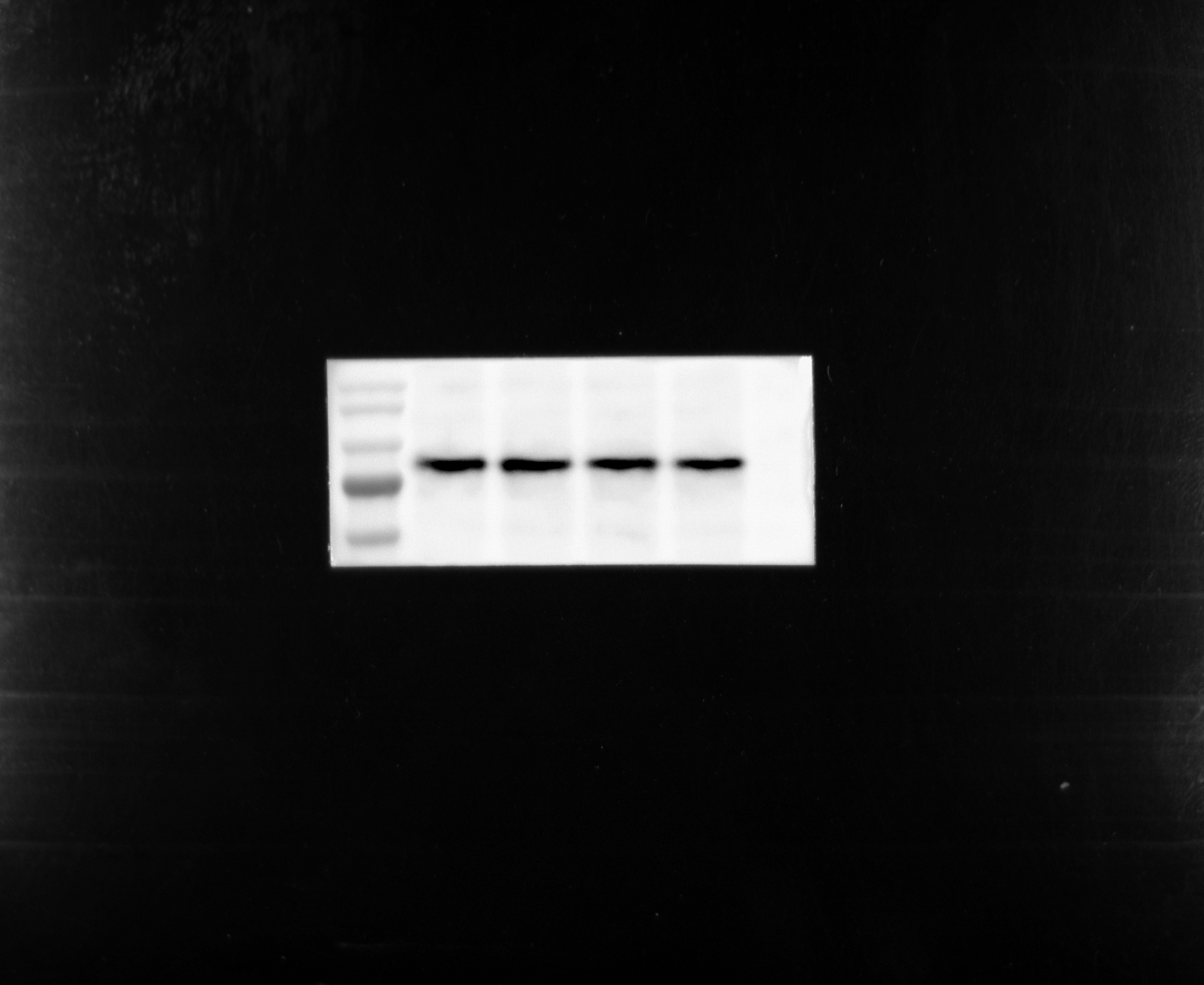

Supplement: Supplementary file 1 [file cimb-47-00936-s001.zip › cimb-3956315-supplementary/APOC2_ccRCC_RawWB_FullMembranes/cropped display images/8/Fig 3E stat1/5.Tif]

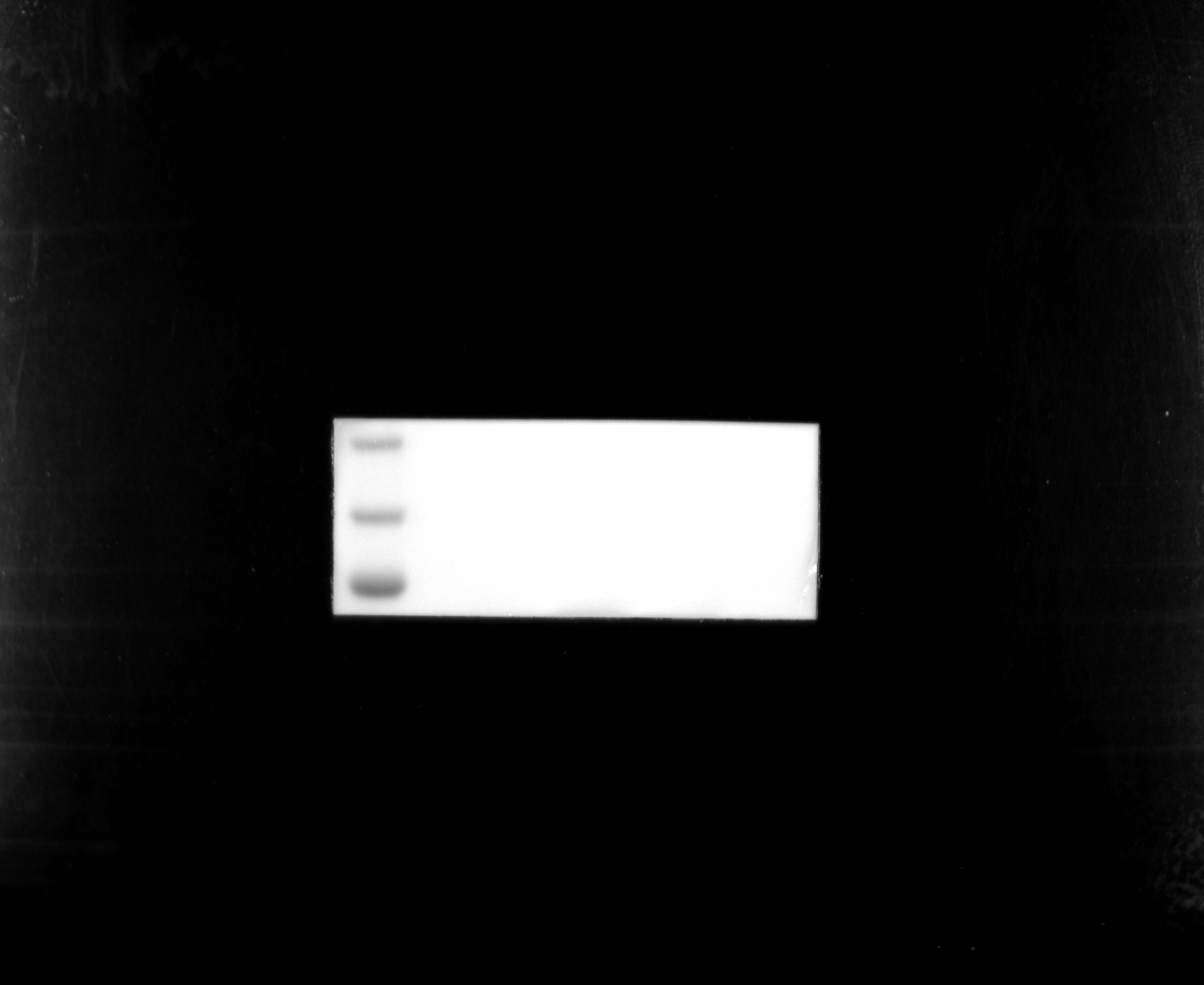

Supplement: Supplementary file 1 [file cimb-47-00936-s001.zip › cimb-3956315-supplementary/APOC2_ccRCC_RawWB_FullMembranes/cropped display images/8/β- actin/0.Tif]

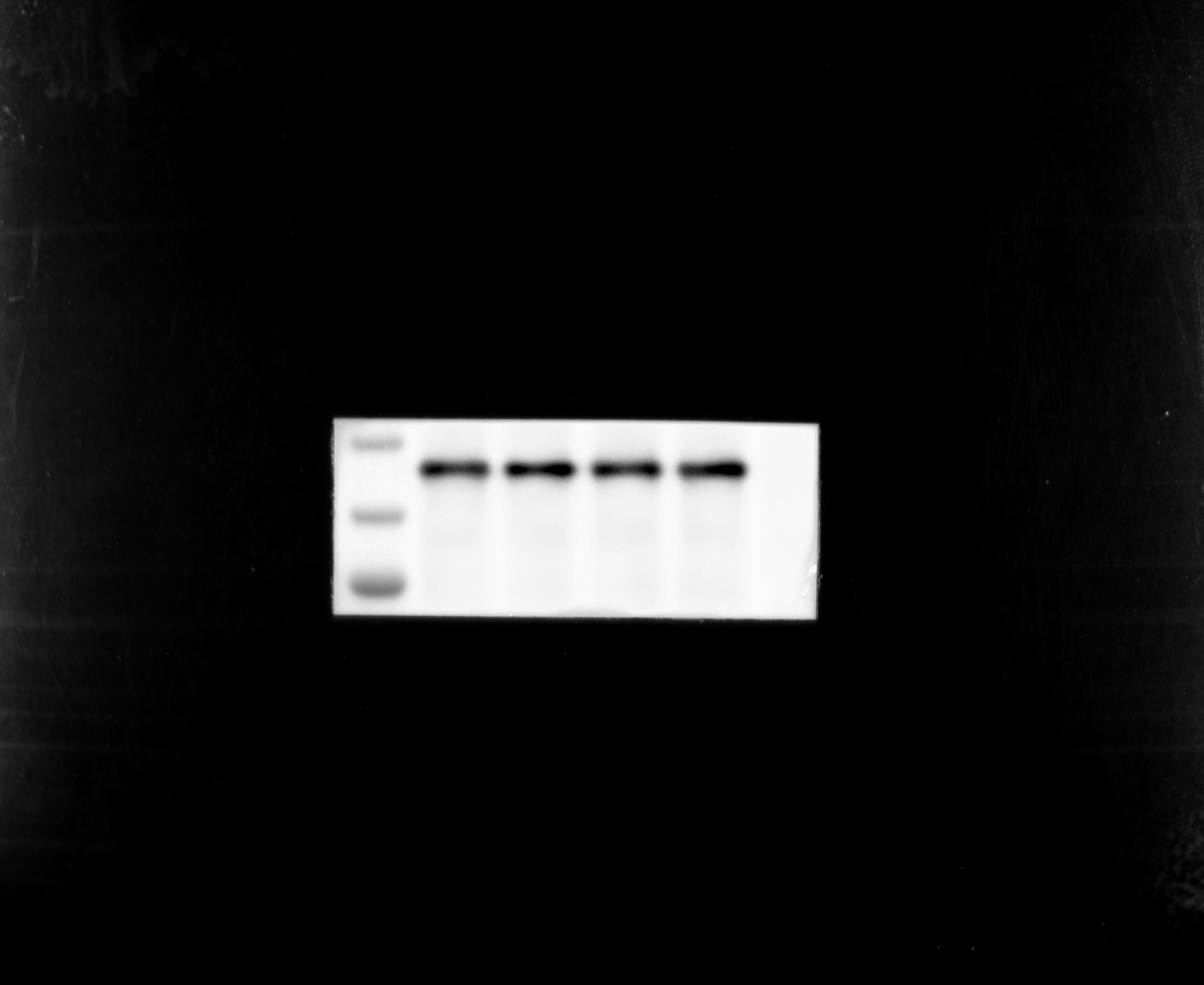

Supplement: Supplementary file 1 [file cimb-47-00936-s001.zip › cimb-3956315-supplementary/APOC2_ccRCC_RawWB_FullMembranes/cropped display images/8/β- actin/1.Tif]

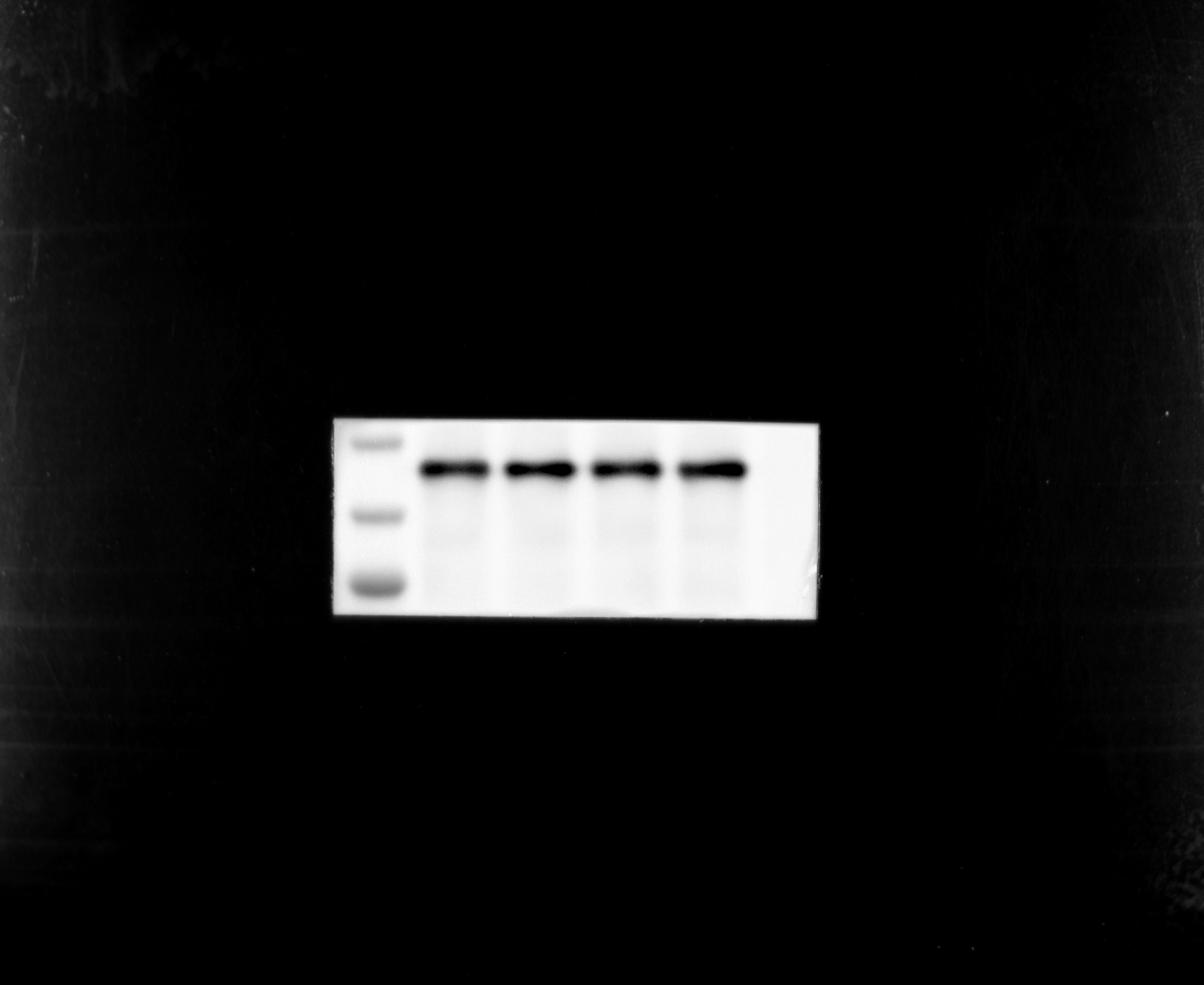

Supplement: Supplementary file 1 [file cimb-47-00936-s001.zip › cimb-3956315-supplementary/APOC2_ccRCC_RawWB_FullMembranes/cropped display images/8/β- actin/2.Tif]

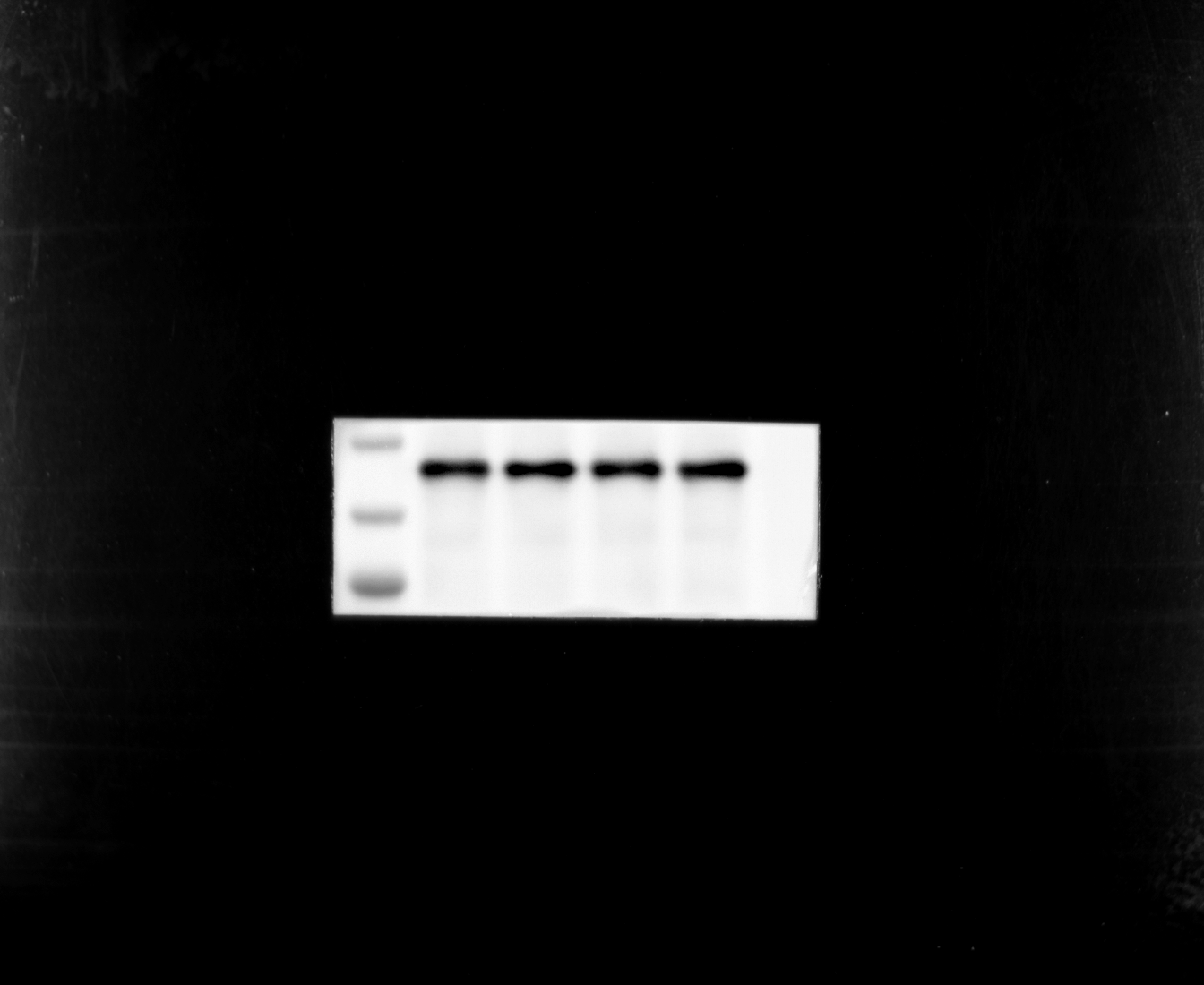

Supplement: Supplementary file 1 [file cimb-47-00936-s001.zip › cimb-3956315-supplementary/APOC2_ccRCC_RawWB_FullMembranes/cropped display images/8/β- actin/3.Tif]

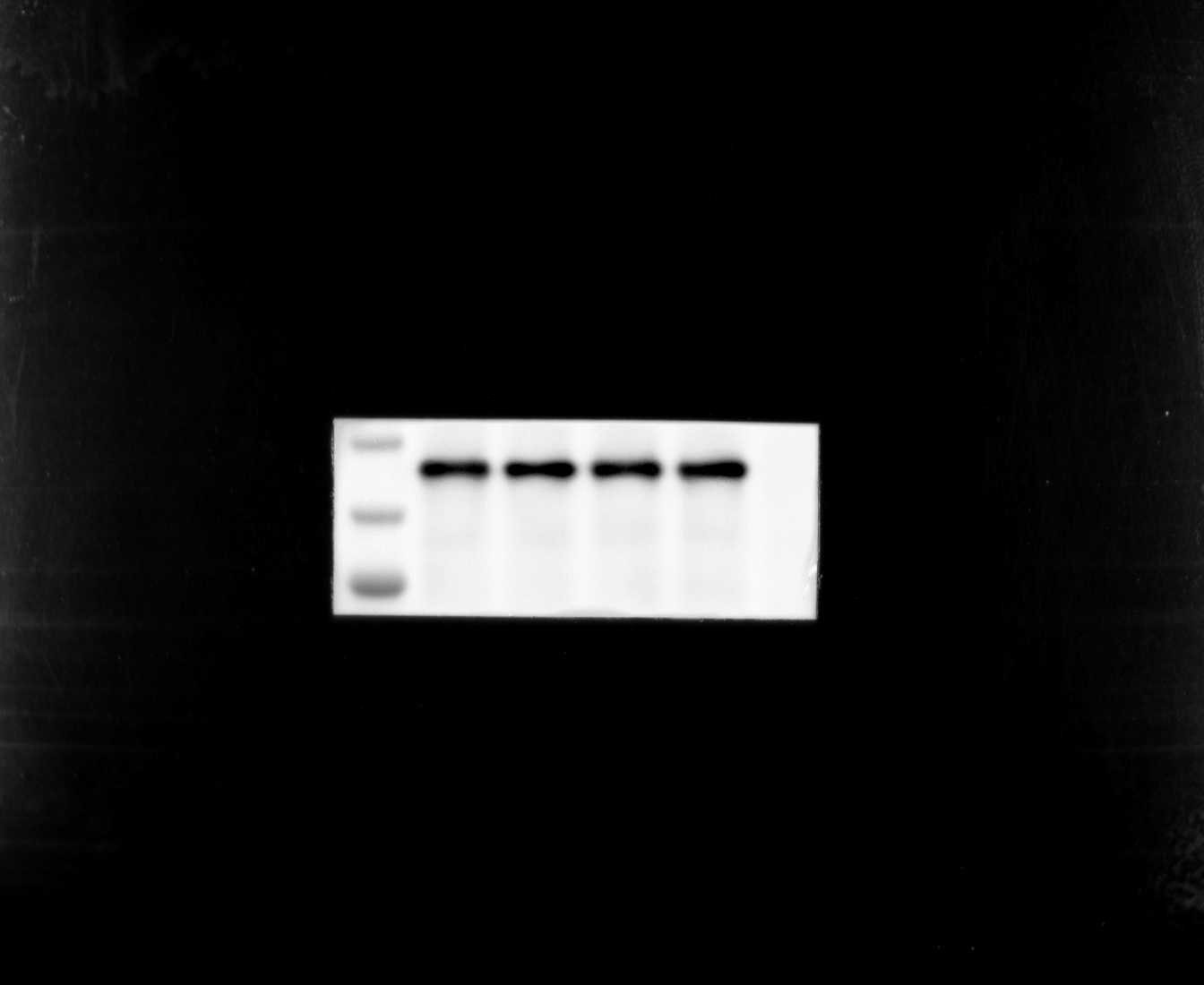

Supplement: Supplementary file 1 [file cimb-47-00936-s001.zip › cimb-3956315-supplementary/APOC2_ccRCC_RawWB_FullMembranes/cropped display images/8/β- actin/4.Tif]

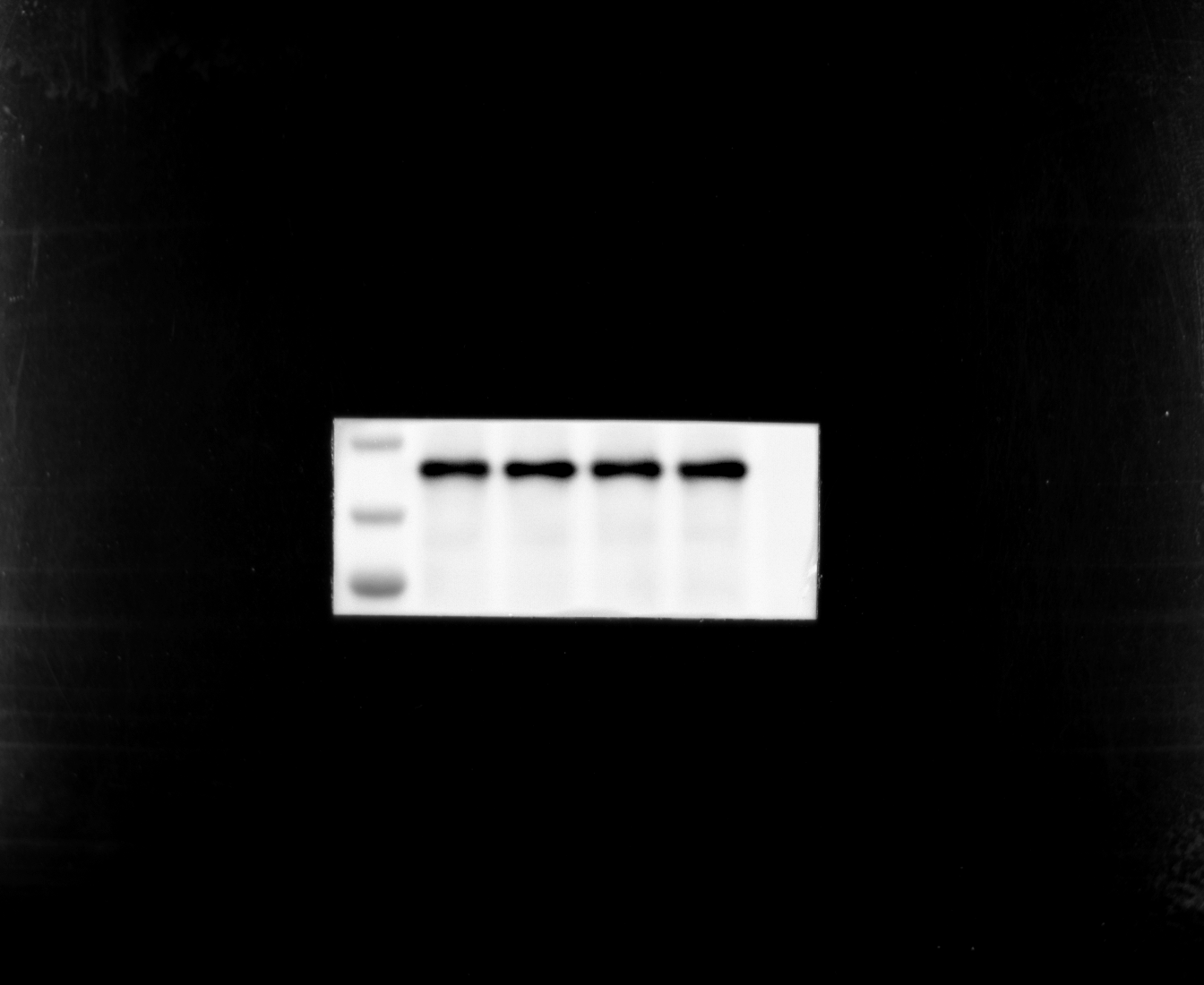

Supplement: Supplementary file 1 [file cimb-47-00936-s001.zip › cimb-3956315-supplementary/APOC2_ccRCC_RawWB_FullMembranes/cropped display images/8/β- actin/5.Tif]

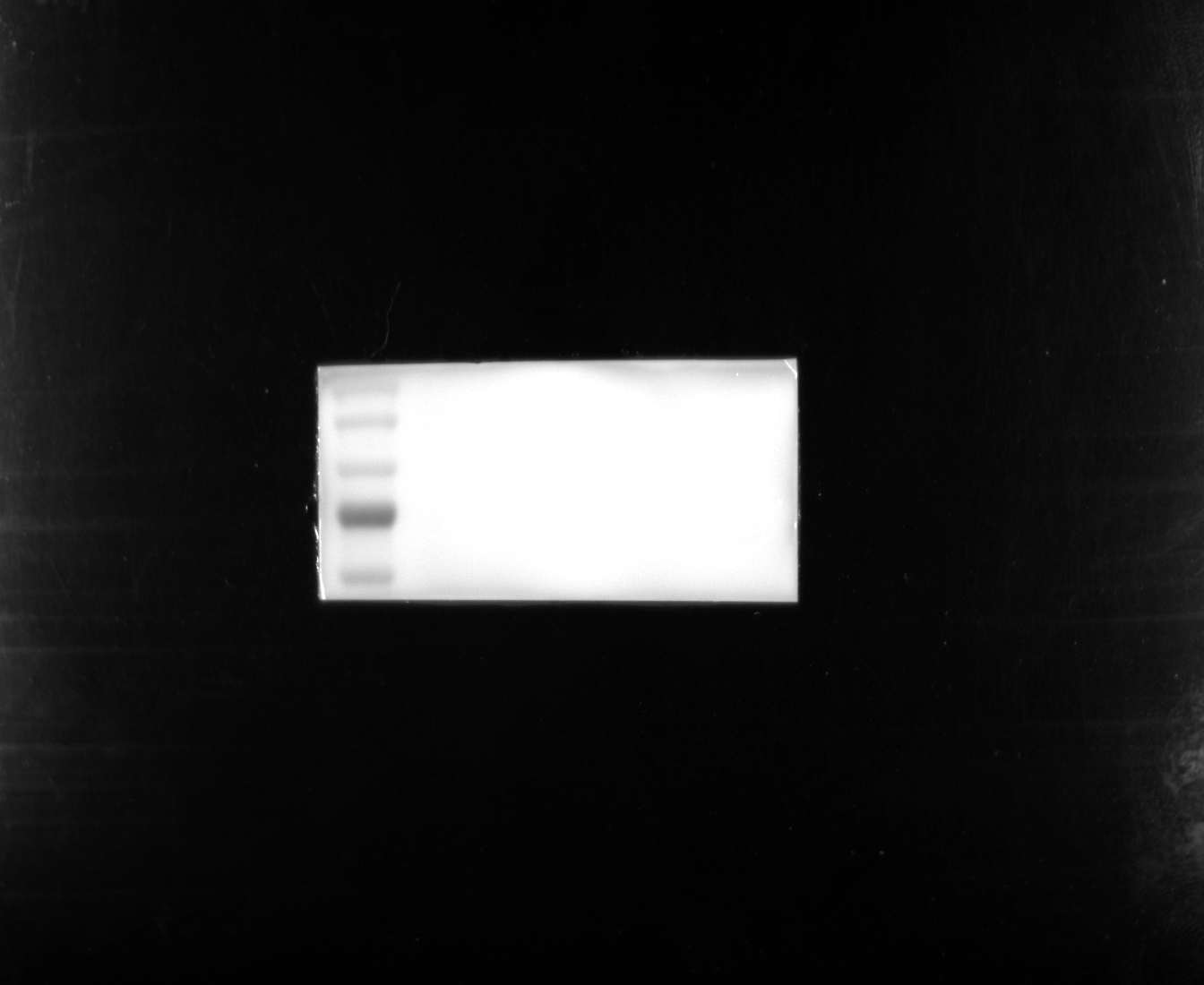

Supplement: Supplementary file 1 [file cimb-47-00936-s001.zip › cimb-3956315-supplementary/APOC2_ccRCC_RawWB_FullMembranes/cropped display images/9/Fig 3E p-stat2/0.Tif]

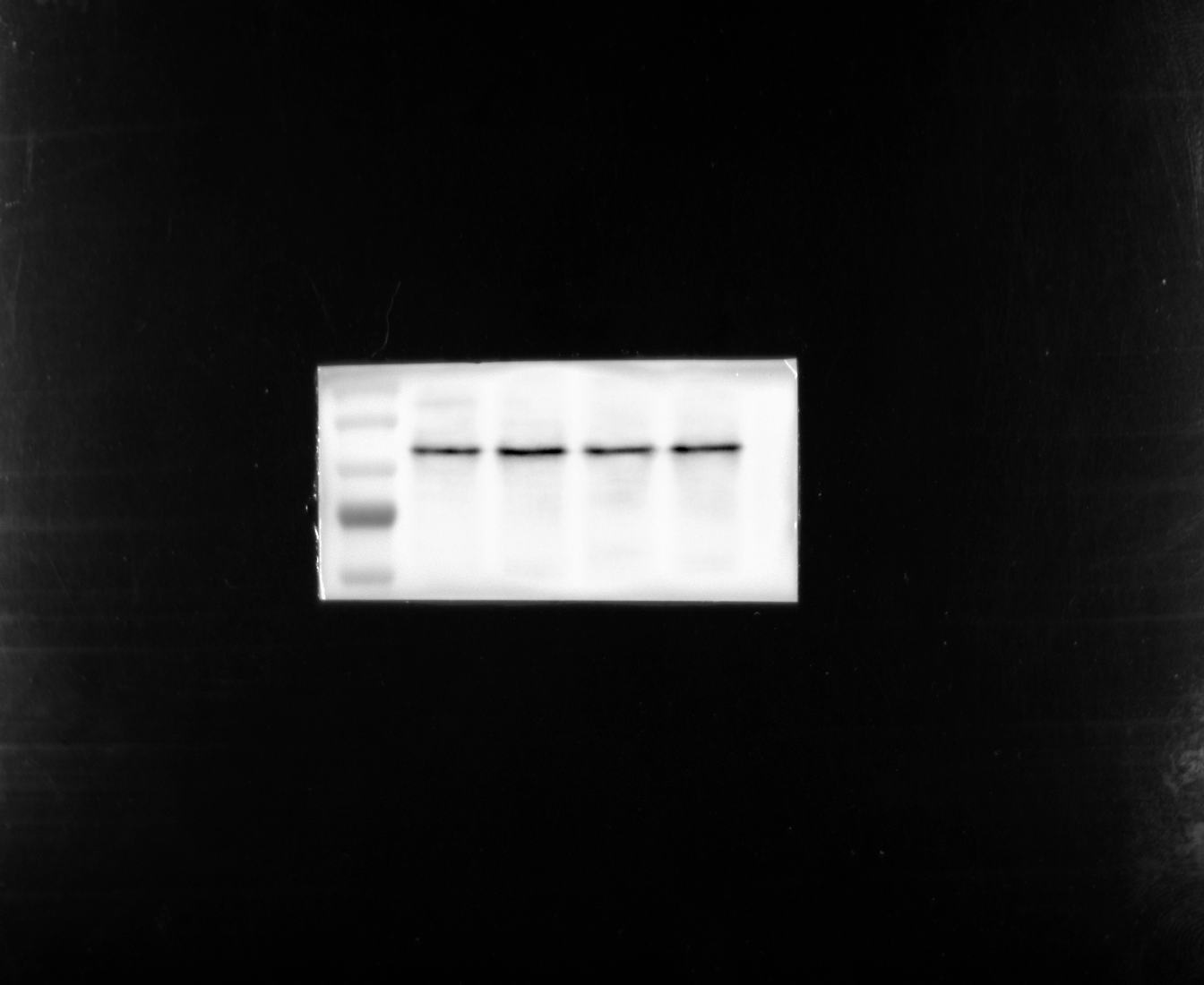

Supplement: Supplementary file 1 [file cimb-47-00936-s001.zip › cimb-3956315-supplementary/APOC2_ccRCC_RawWB_FullMembranes/cropped display images/9/Fig 3E p-stat2/1.Tif]

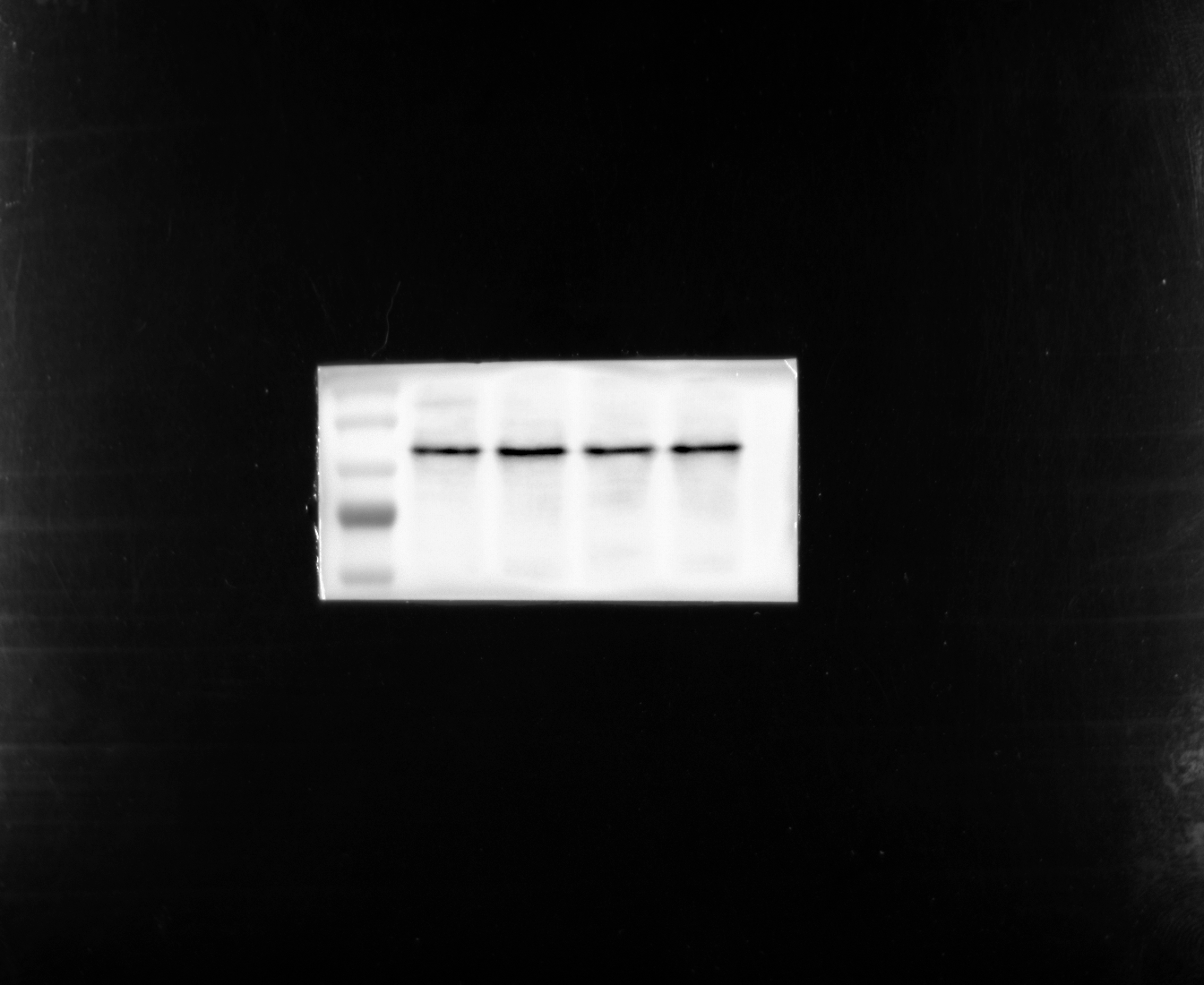

Supplement: Supplementary file 1 [file cimb-47-00936-s001.zip › cimb-3956315-supplementary/APOC2_ccRCC_RawWB_FullMembranes/cropped display images/9/Fig 3E p-stat2/2.Tif]

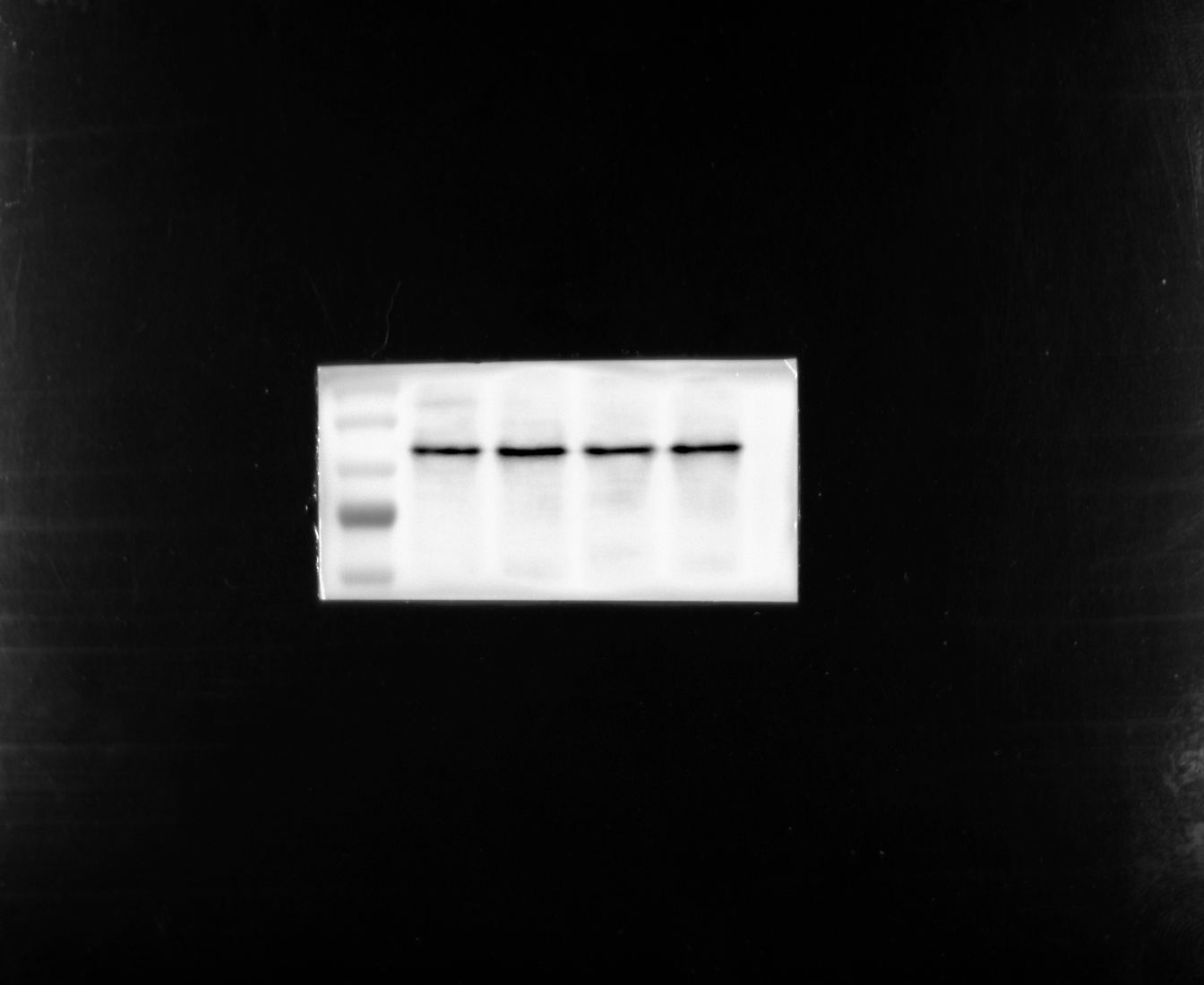

Supplement: Supplementary file 1 [file cimb-47-00936-s001.zip › cimb-3956315-supplementary/APOC2_ccRCC_RawWB_FullMembranes/cropped display images/9/Fig 3E p-stat2/3.Tif]

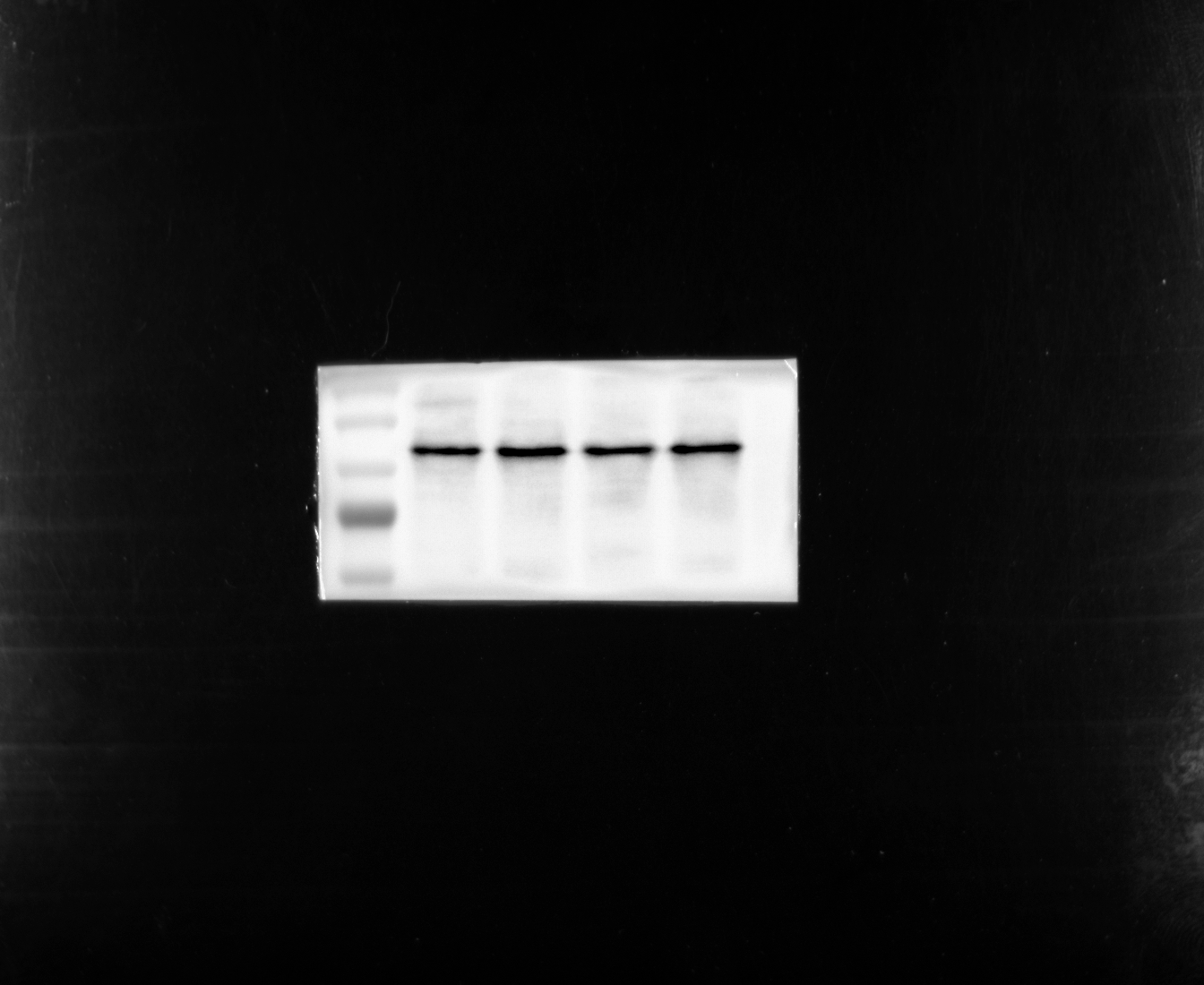

Supplement: Supplementary file 1 [file cimb-47-00936-s001.zip › cimb-3956315-supplementary/APOC2_ccRCC_RawWB_FullMembranes/cropped display images/9/Fig 3E p-stat2/4.Tif]

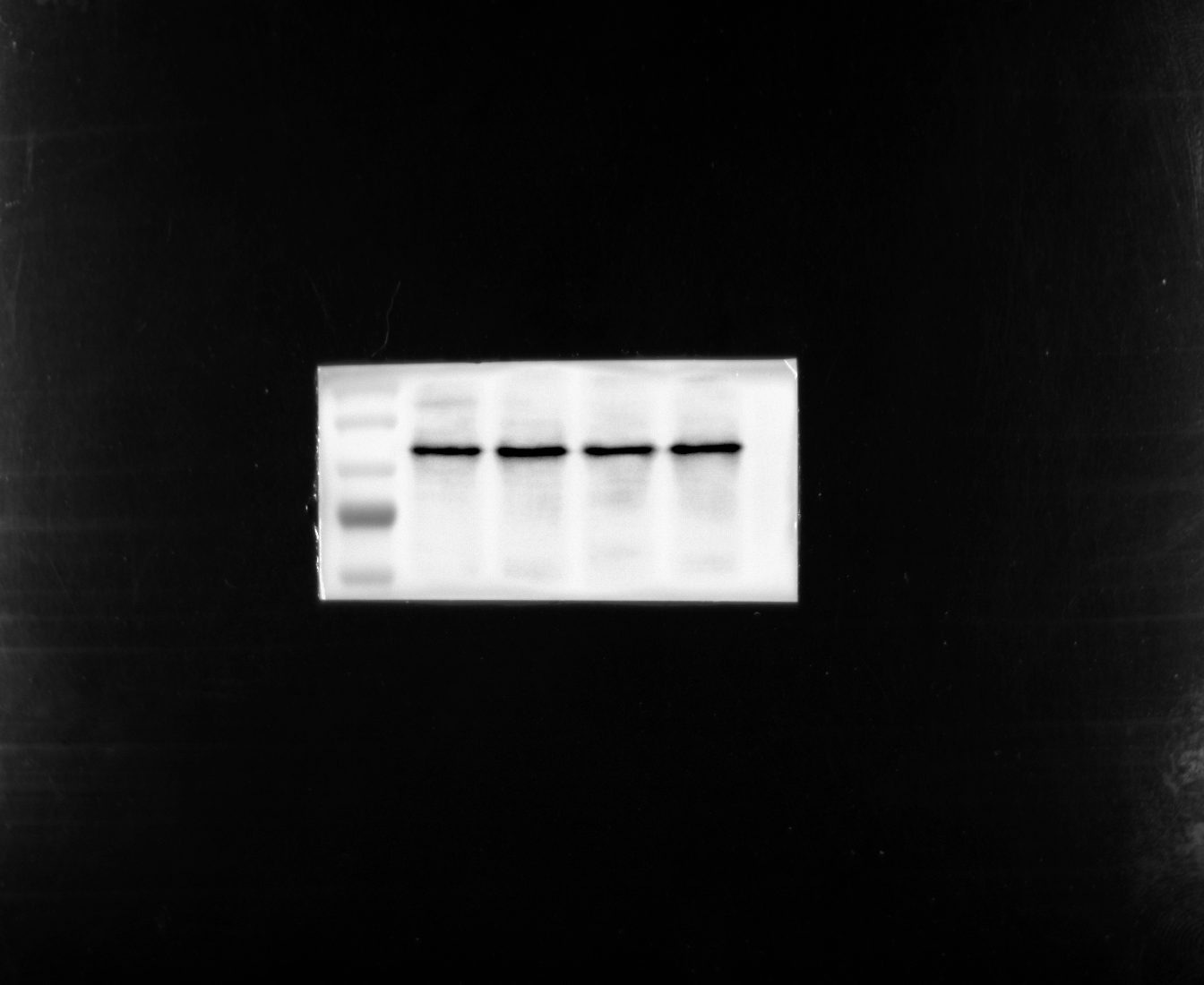

Supplement: Supplementary file 1 [file cimb-47-00936-s001.zip › cimb-3956315-supplementary/APOC2_ccRCC_RawWB_FullMembranes/cropped display images/9/Fig 3E p-stat2/5.Tif]

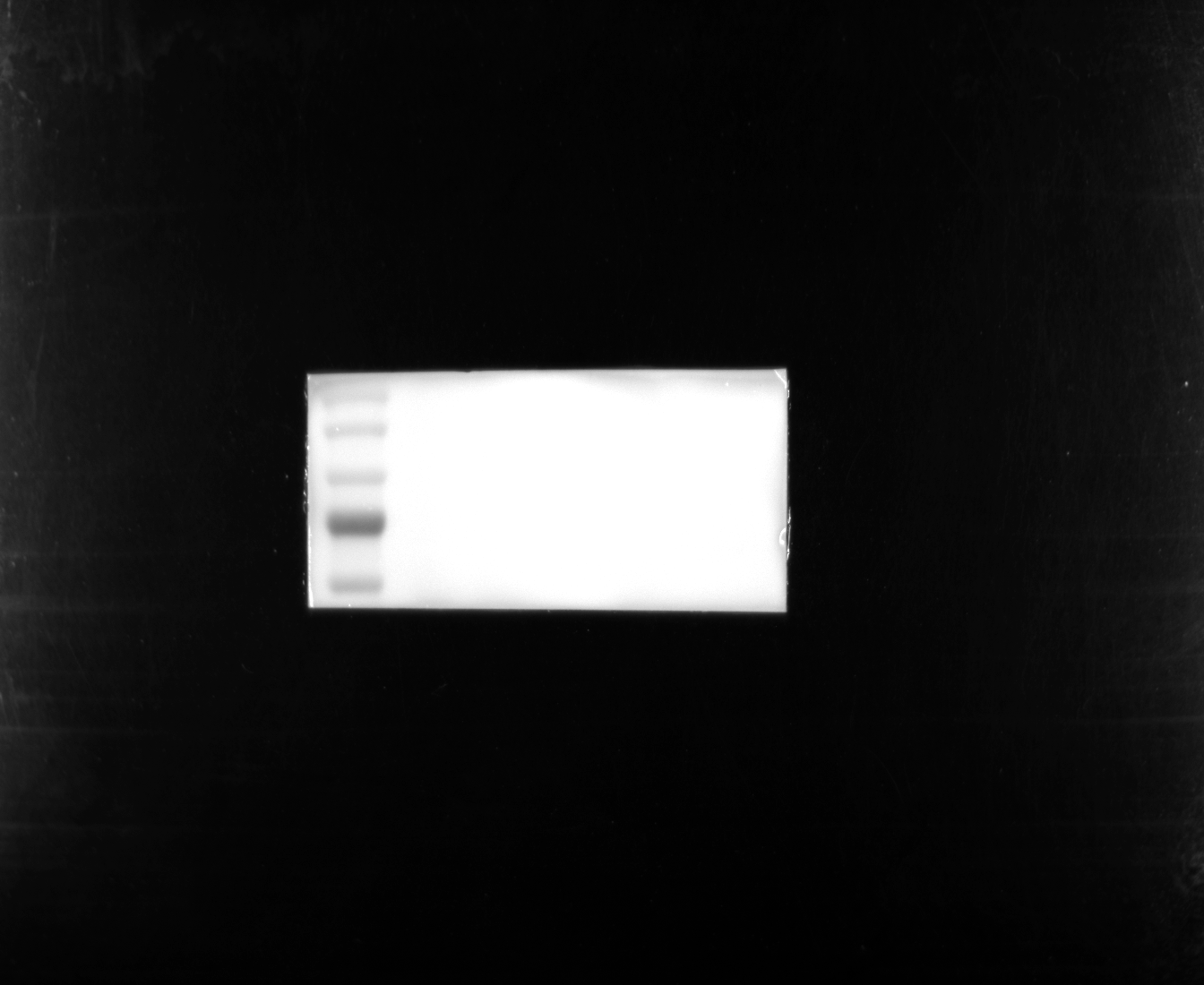

Supplement: Supplementary file 1 [file cimb-47-00936-s001.zip › cimb-3956315-supplementary/APOC2_ccRCC_RawWB_FullMembranes/cropped display images/9/Fig 3E stat2/0.Tif]

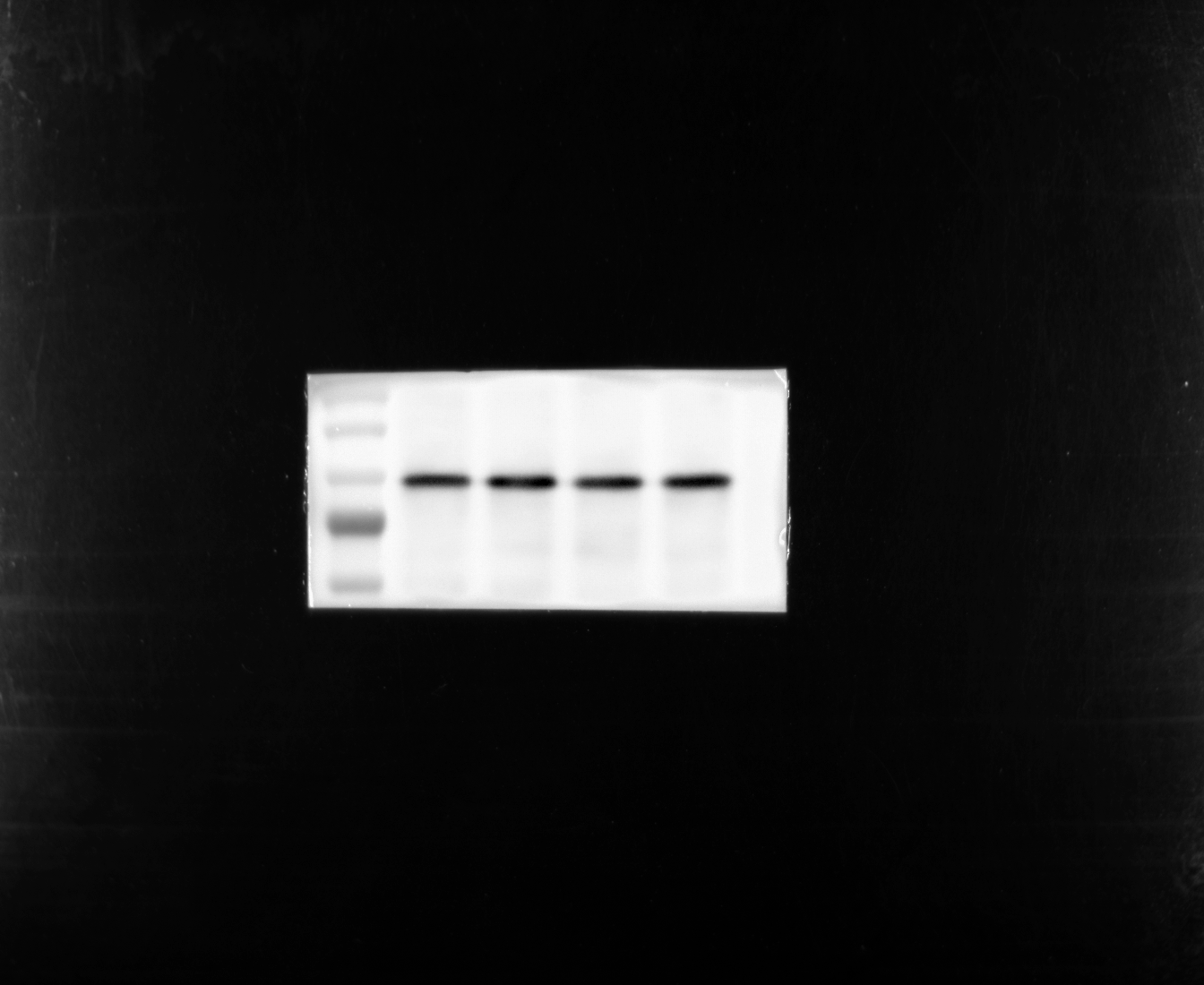

Supplement: Supplementary file 1 [file cimb-47-00936-s001.zip › cimb-3956315-supplementary/APOC2_ccRCC_RawWB_FullMembranes/cropped display images/9/Fig 3E stat2/1.Tif]

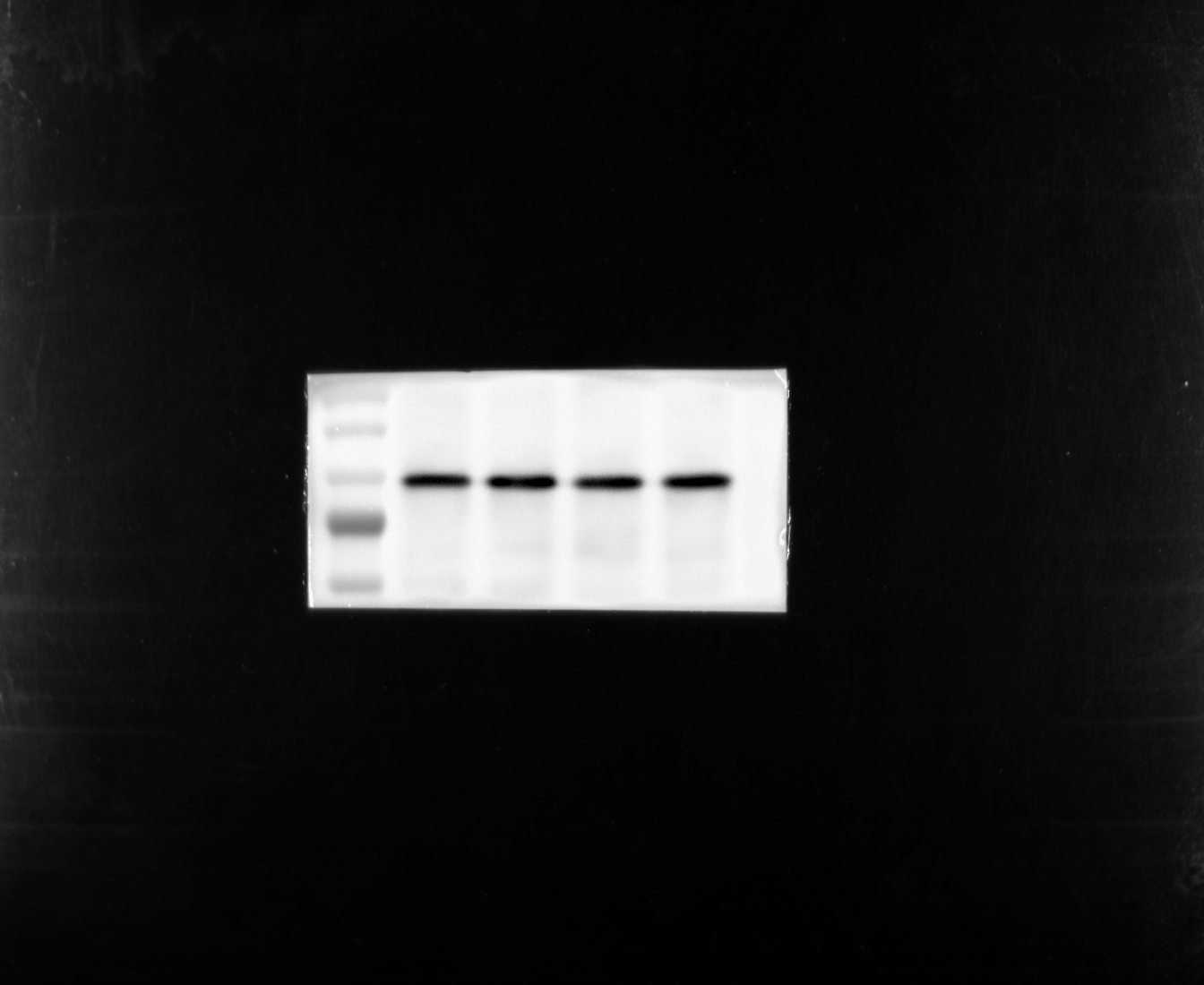

Supplement: Supplementary file 1 [file cimb-47-00936-s001.zip › cimb-3956315-supplementary/APOC2_ccRCC_RawWB_FullMembranes/cropped display images/9/Fig 3E stat2/2.Tif]

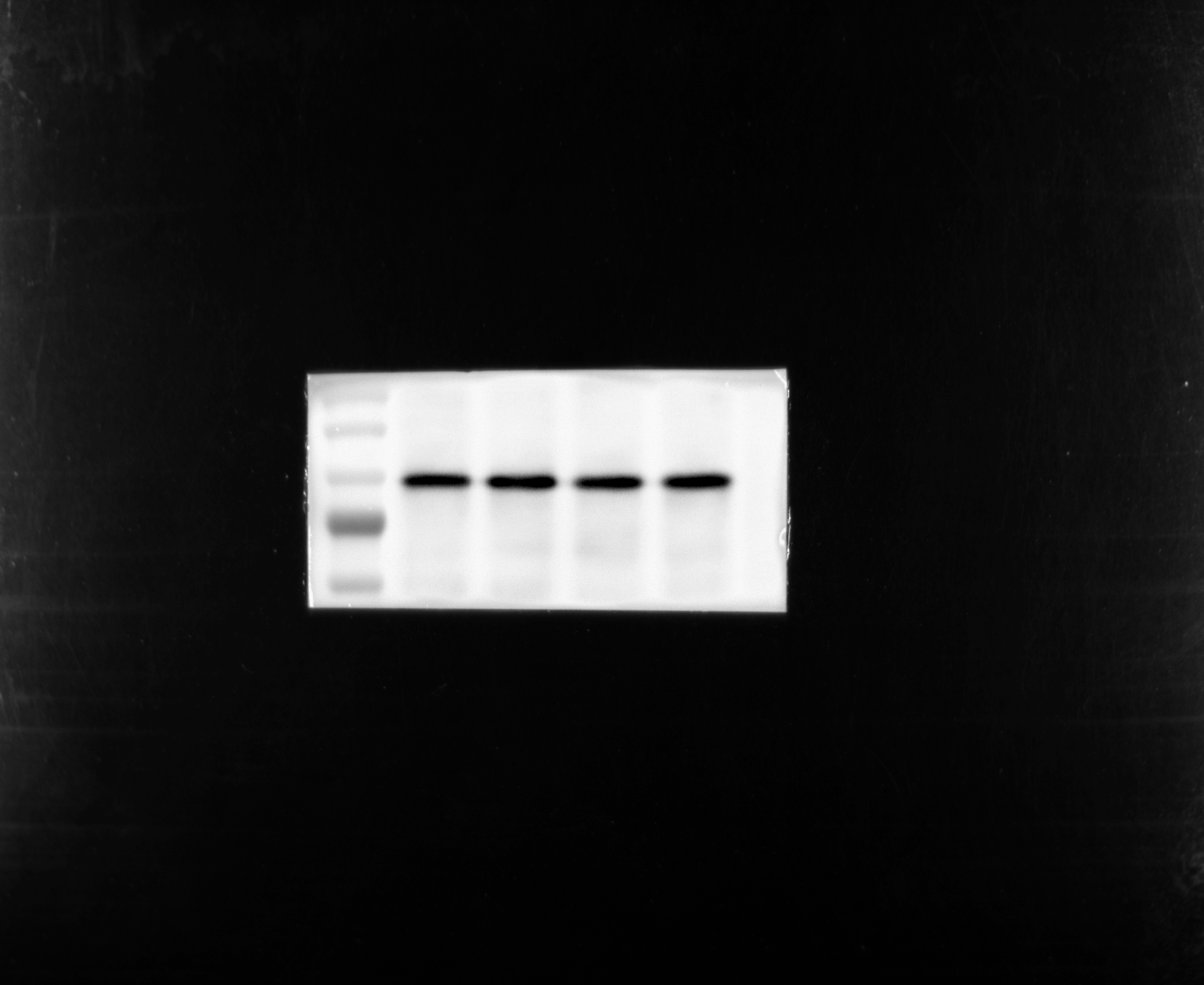

Supplement: Supplementary file 1 [file cimb-47-00936-s001.zip › cimb-3956315-supplementary/APOC2_ccRCC_RawWB_FullMembranes/cropped display images/9/Fig 3E stat2/3.Tif]

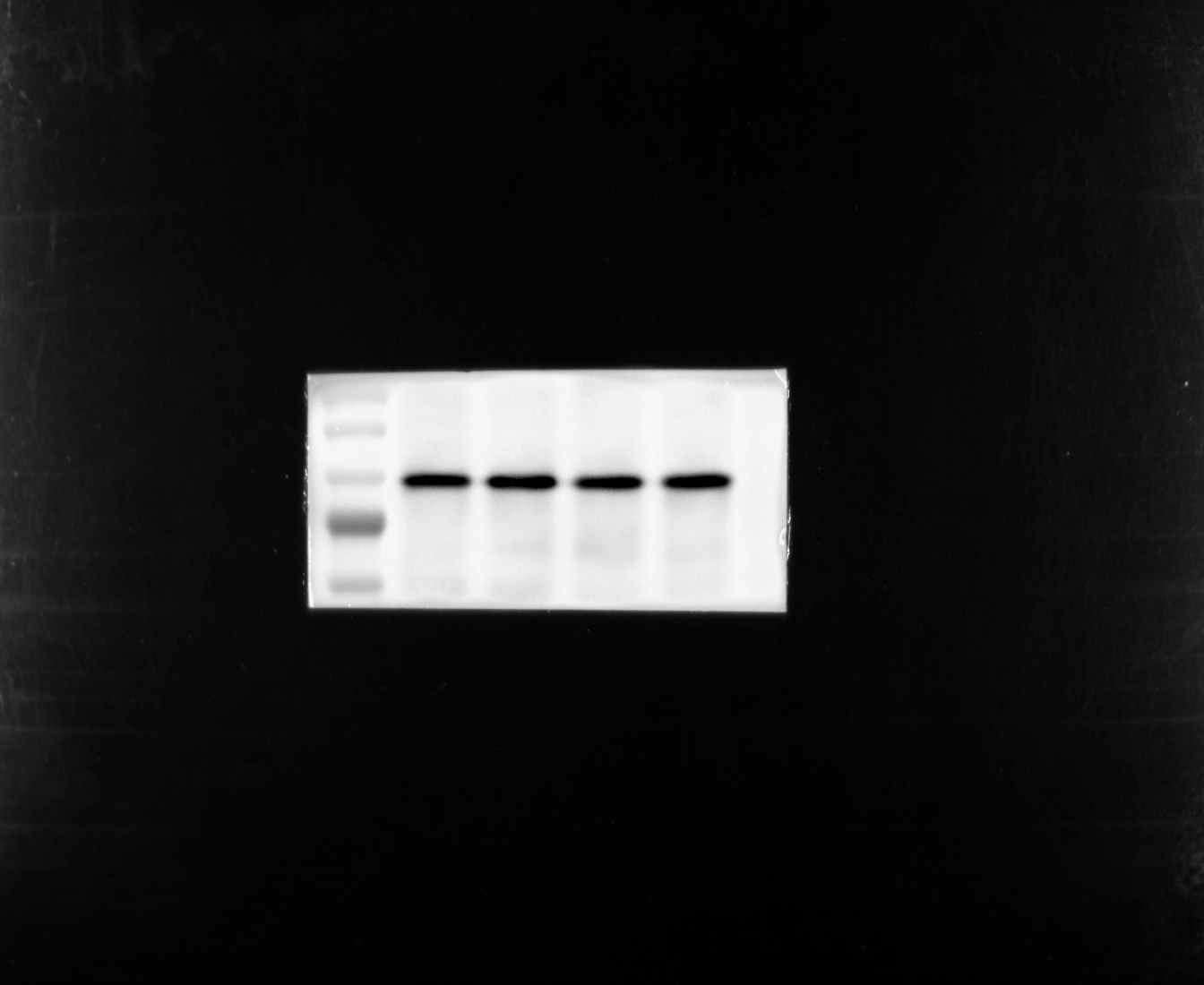

Supplement: Supplementary file 1 [file cimb-47-00936-s001.zip › cimb-3956315-supplementary/APOC2_ccRCC_RawWB_FullMembranes/cropped display images/9/Fig 3E stat2/4.Tif]

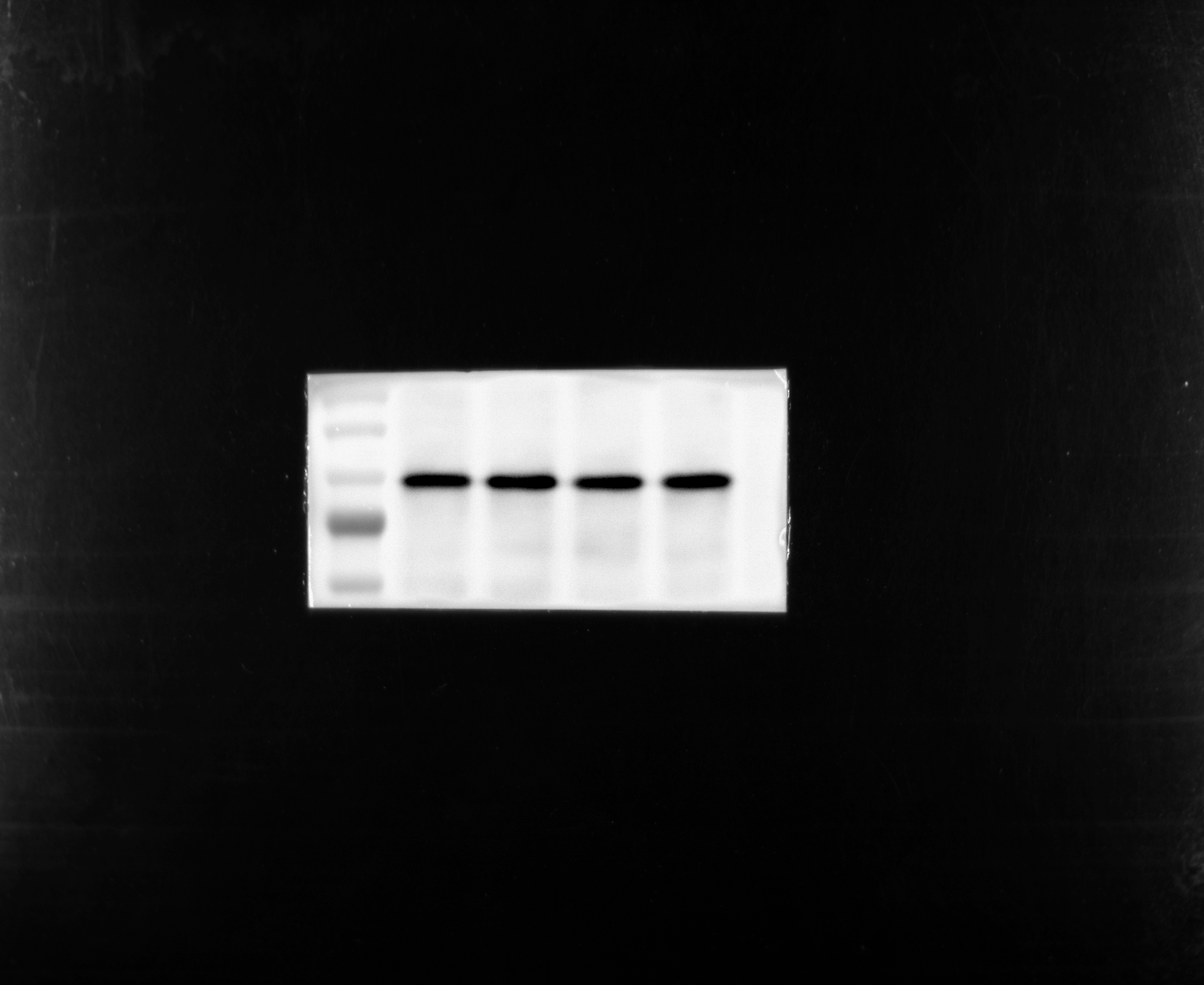

Supplement: Supplementary file 1 [file cimb-47-00936-s001.zip › cimb-3956315-supplementary/APOC2_ccRCC_RawWB_FullMembranes/cropped display images/9/Fig 3E stat2/5.Tif]

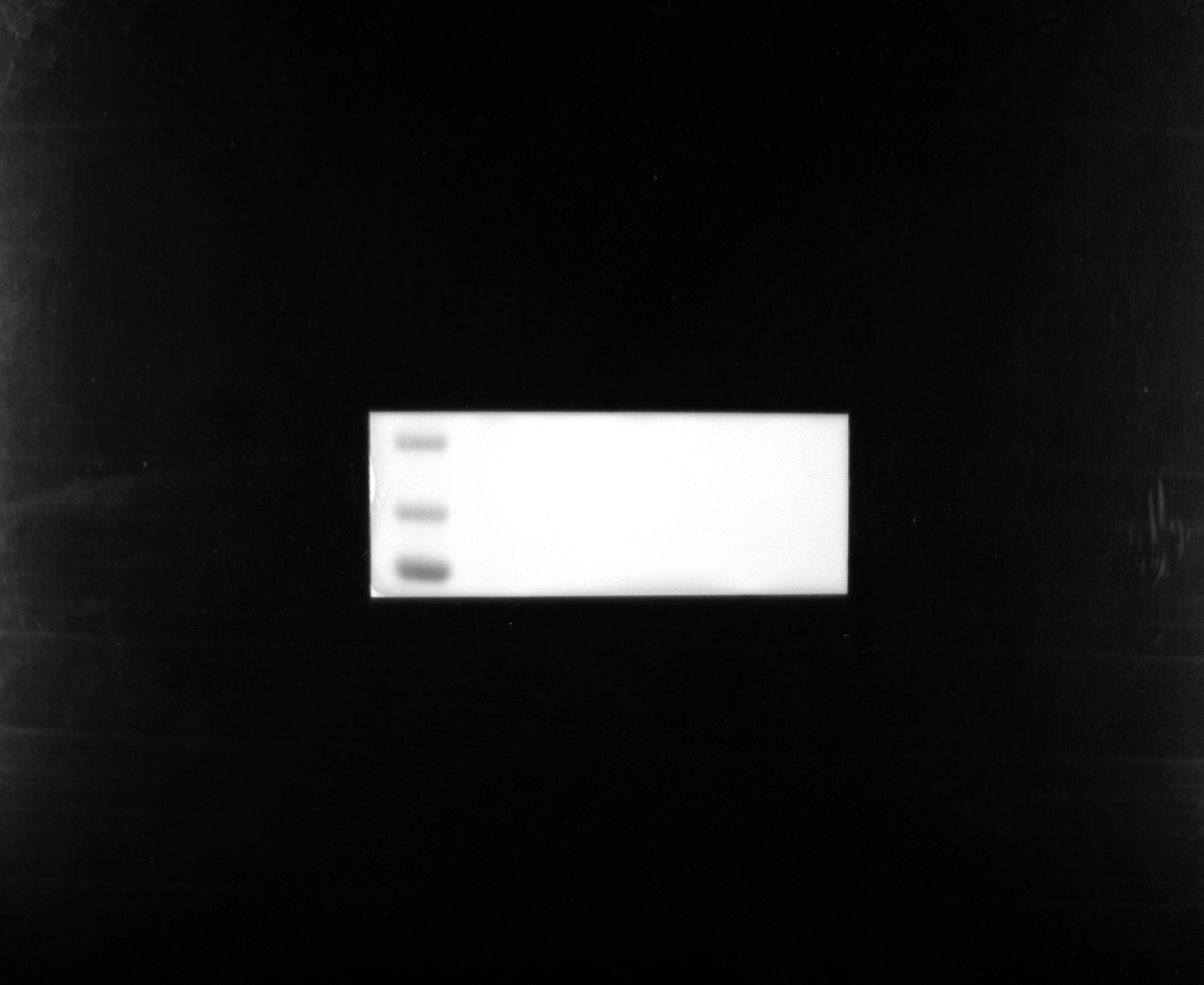

Supplement: Supplementary file 1 [file cimb-47-00936-s001.zip › cimb-3956315-supplementary/APOC2_ccRCC_RawWB_FullMembranes/cropped display images/9/Fig 3E β- actin/0.Tif]

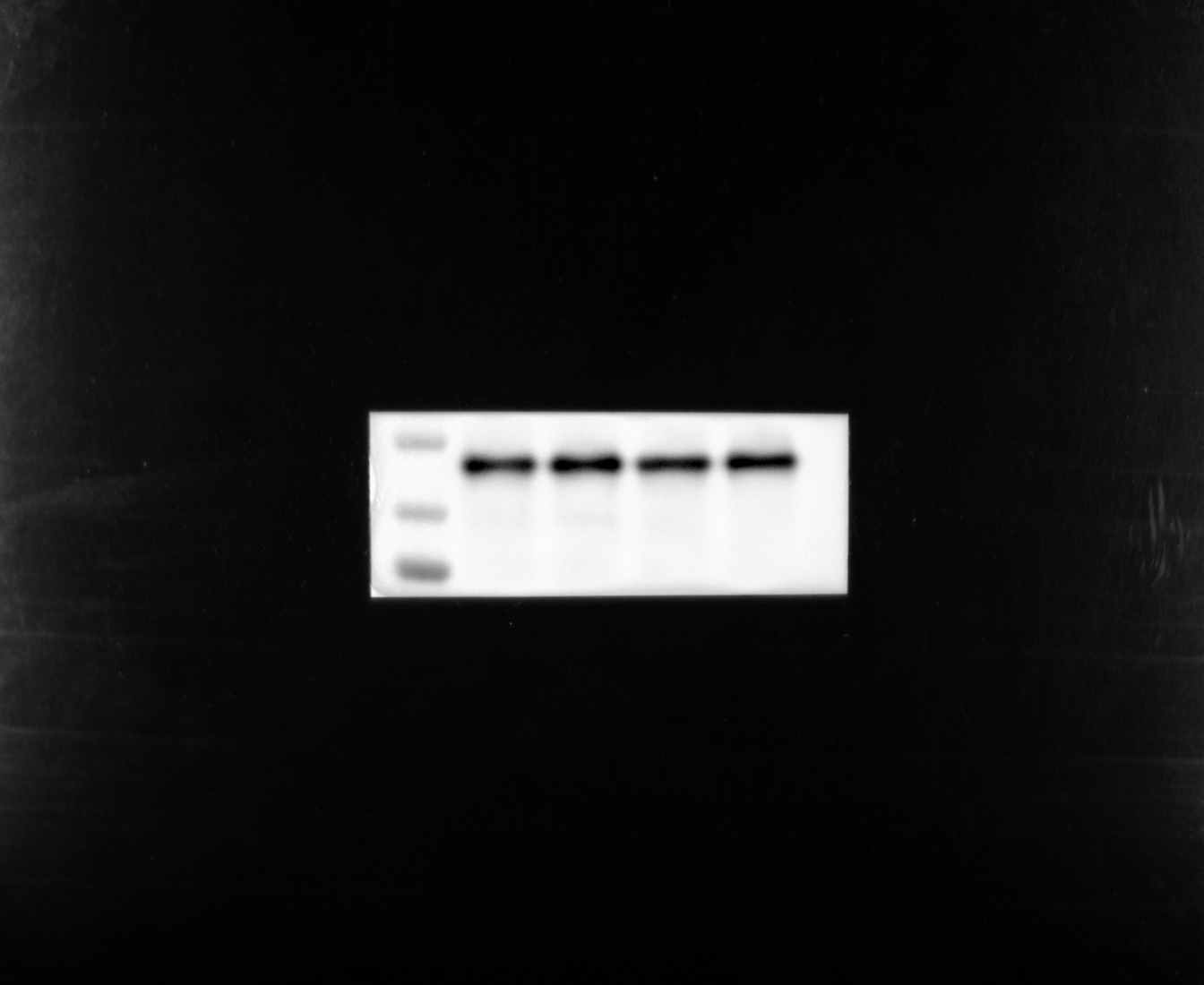

Supplement: Supplementary file 1 [file cimb-47-00936-s001.zip › cimb-3956315-supplementary/APOC2_ccRCC_RawWB_FullMembranes/cropped display images/9/Fig 3E β- actin/1.Tif]

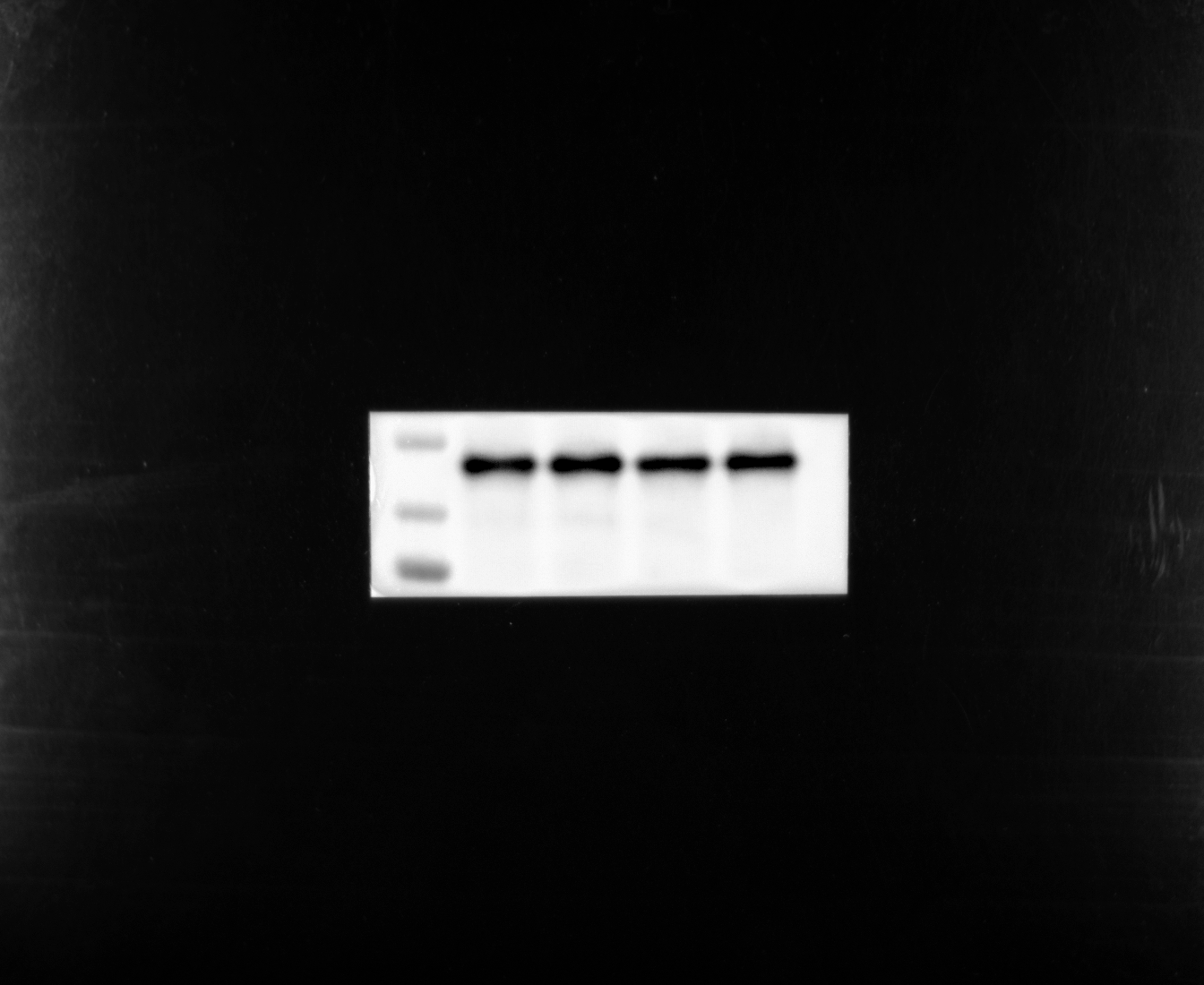

Supplement: Supplementary file 1 [file cimb-47-00936-s001.zip › cimb-3956315-supplementary/APOC2_ccRCC_RawWB_FullMembranes/cropped display images/9/Fig 3E β- actin/2.Tif]

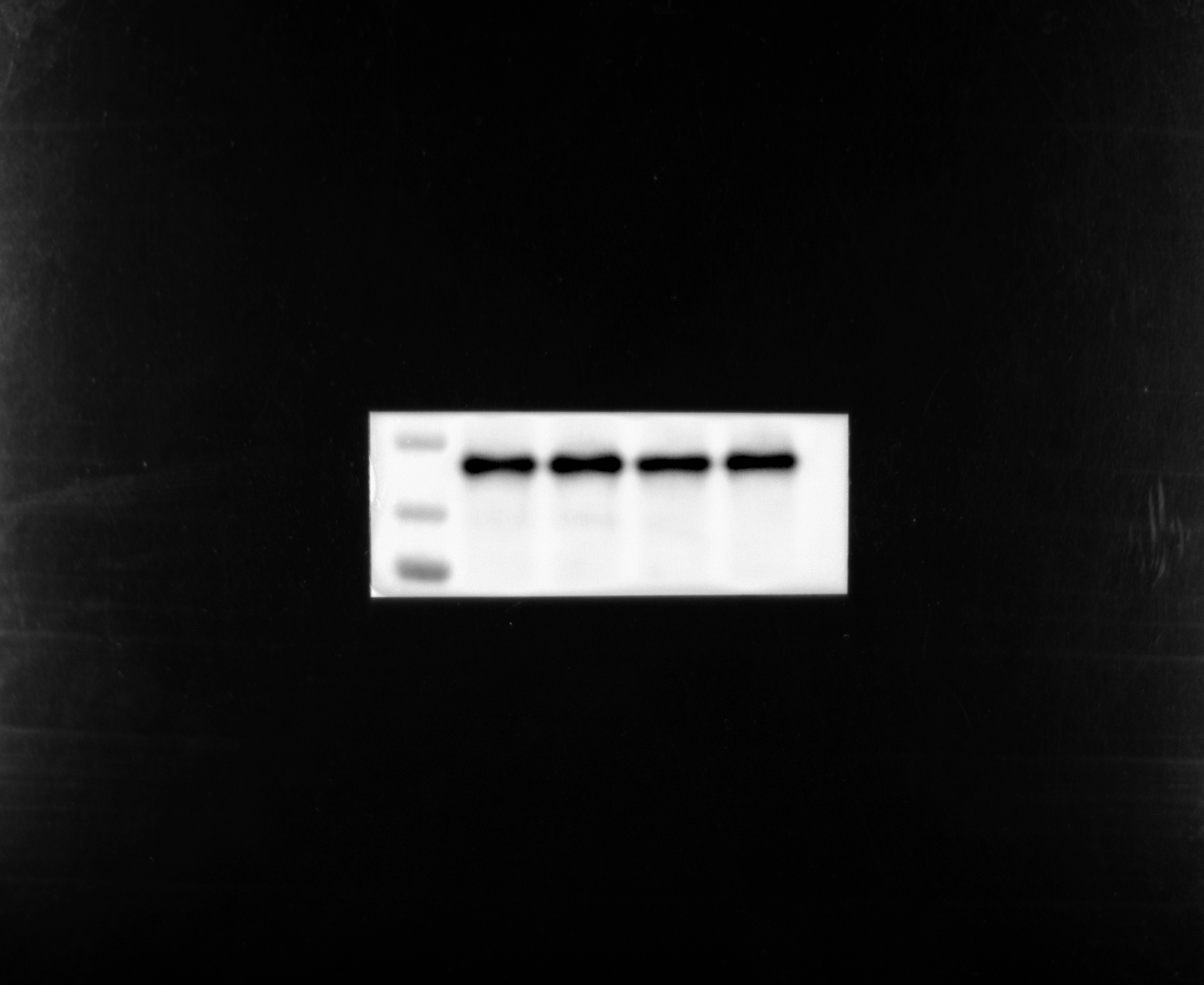

Supplement: Supplementary file 1 [file cimb-47-00936-s001.zip › cimb-3956315-supplementary/APOC2_ccRCC_RawWB_FullMembranes/cropped display images/9/Fig 3E β- actin/3.Tif]

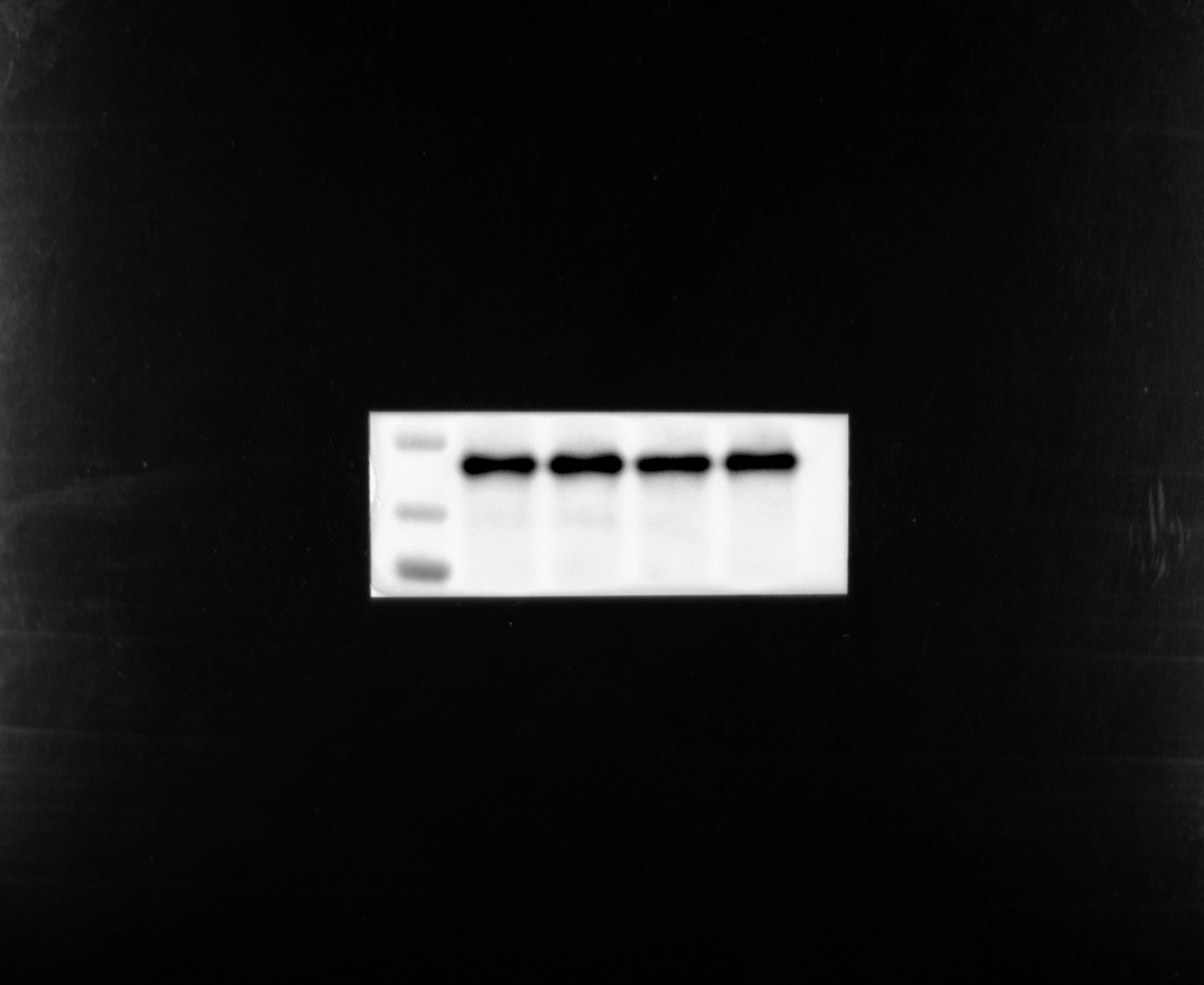

Supplement: Supplementary file 1 [file cimb-47-00936-s001.zip › cimb-3956315-supplementary/APOC2_ccRCC_RawWB_FullMembranes/cropped display images/9/Fig 3E β- actin/4.Tif]

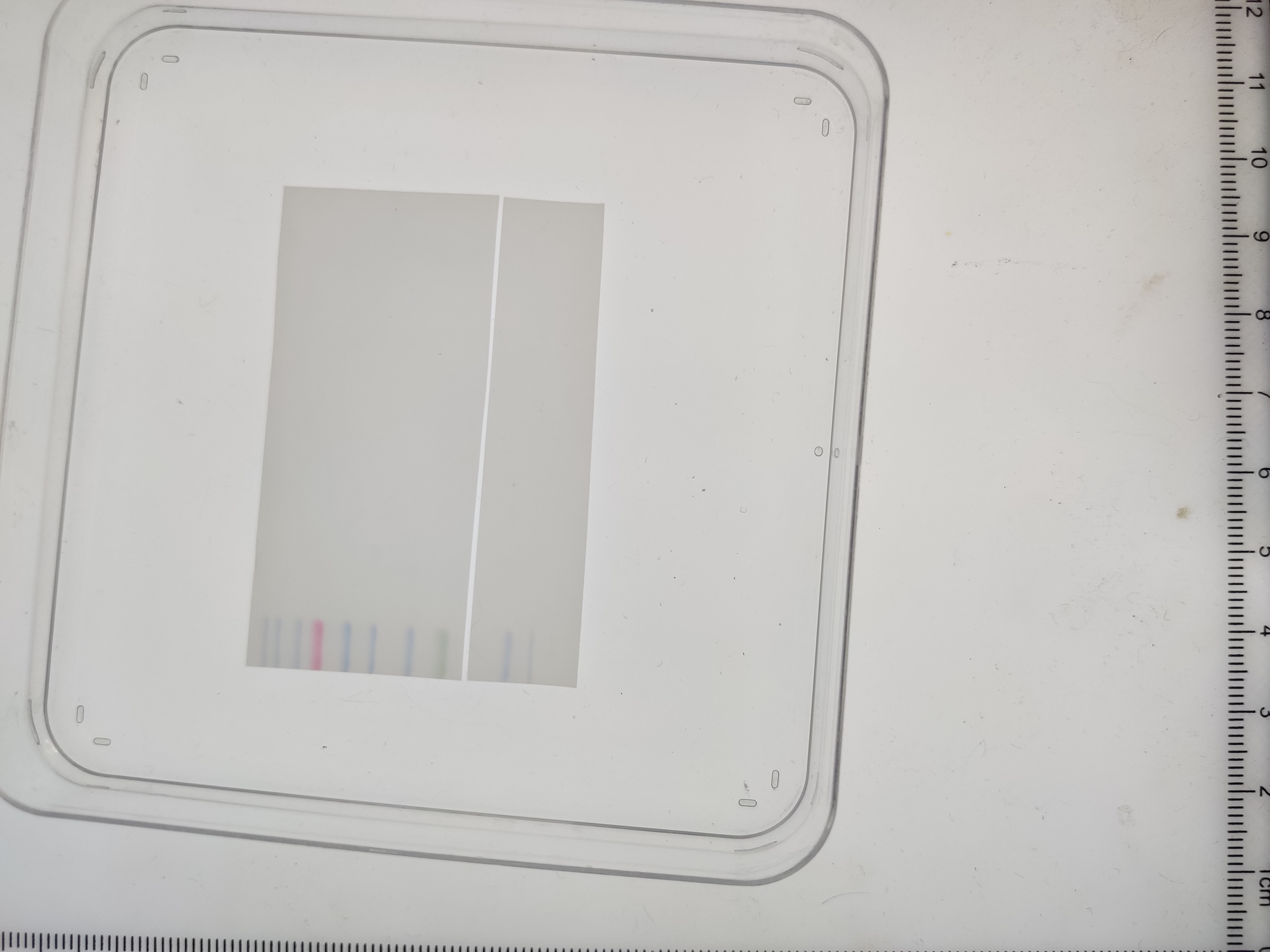

Supplement: Supplementary file 1 [file cimb-47-00936-s001.zip › cimb-3956315-supplementary/APOC2_ccRCC_RawWB_FullMembranes/Uncropped, unprocessed full-length Western blot images/1/1-.jpg]

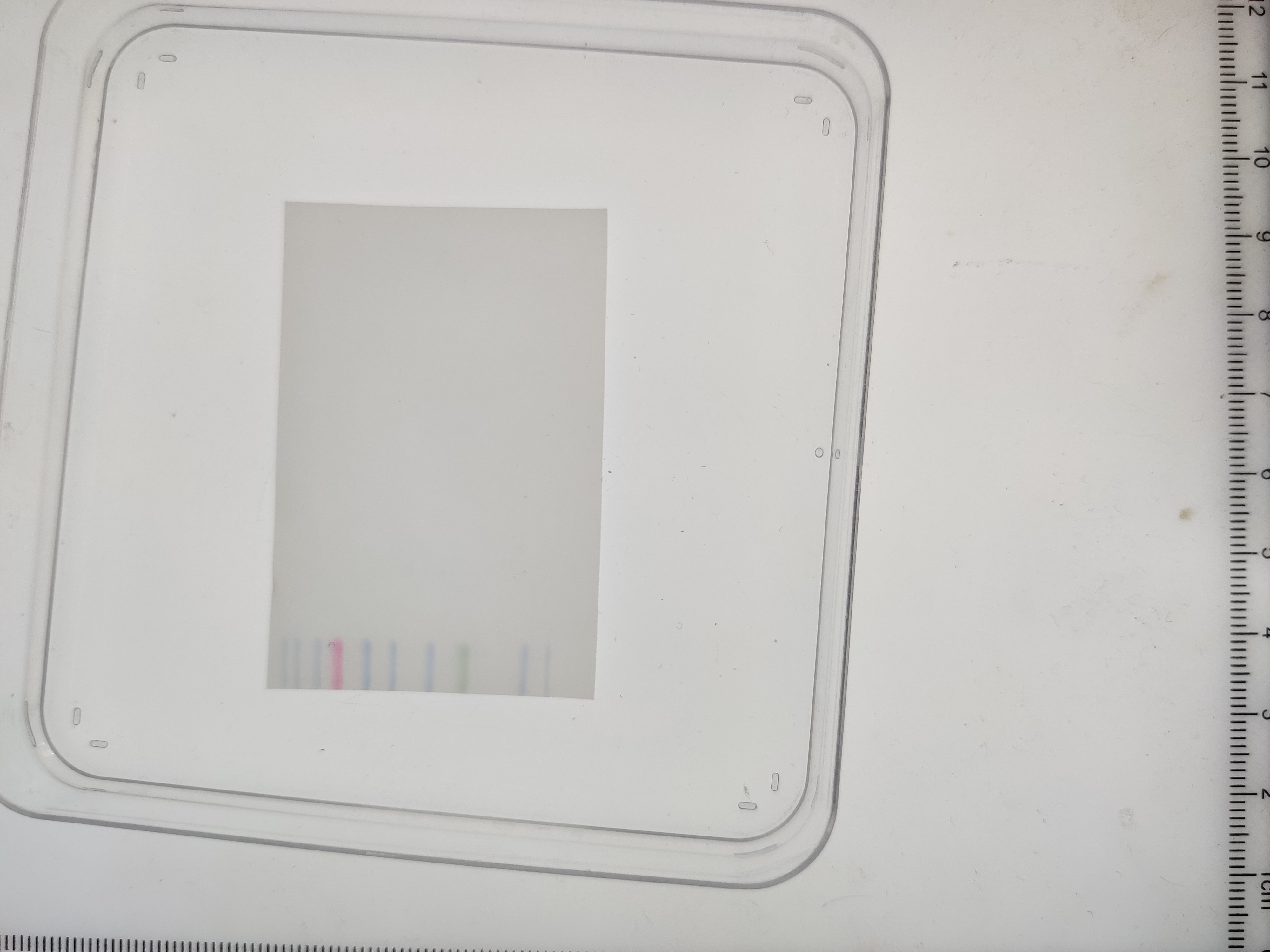

Supplement: Supplementary file 1 [file cimb-47-00936-s001.zip › cimb-3956315-supplementary/APOC2_ccRCC_RawWB_FullMembranes/Uncropped, unprocessed full-length Western blot images/1/1.jpg]

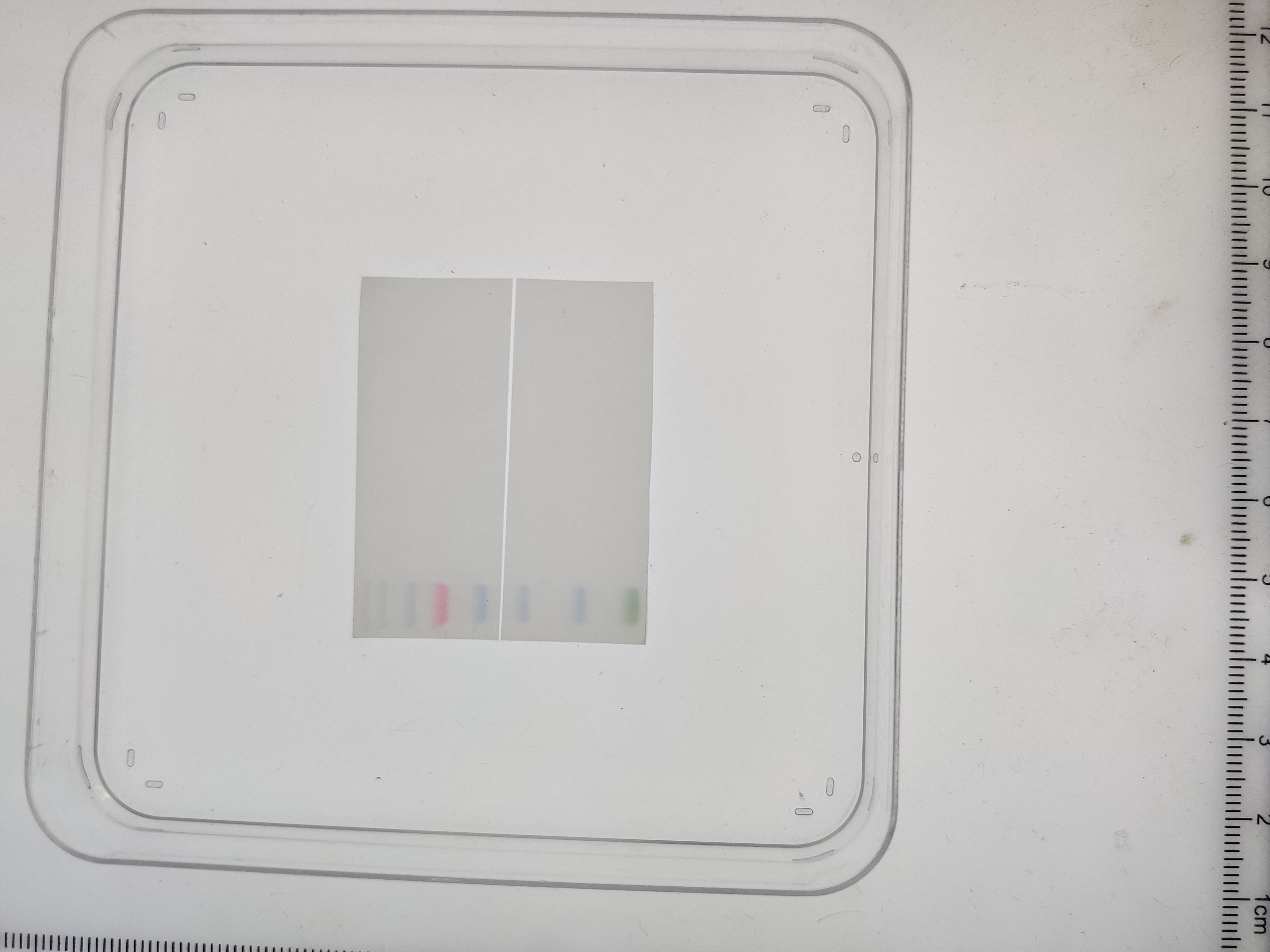

Supplement: Supplementary file 1 [file cimb-47-00936-s001.zip › cimb-3956315-supplementary/APOC2_ccRCC_RawWB_FullMembranes/Uncropped, unprocessed full-length Western blot images/10/10-.jpg]

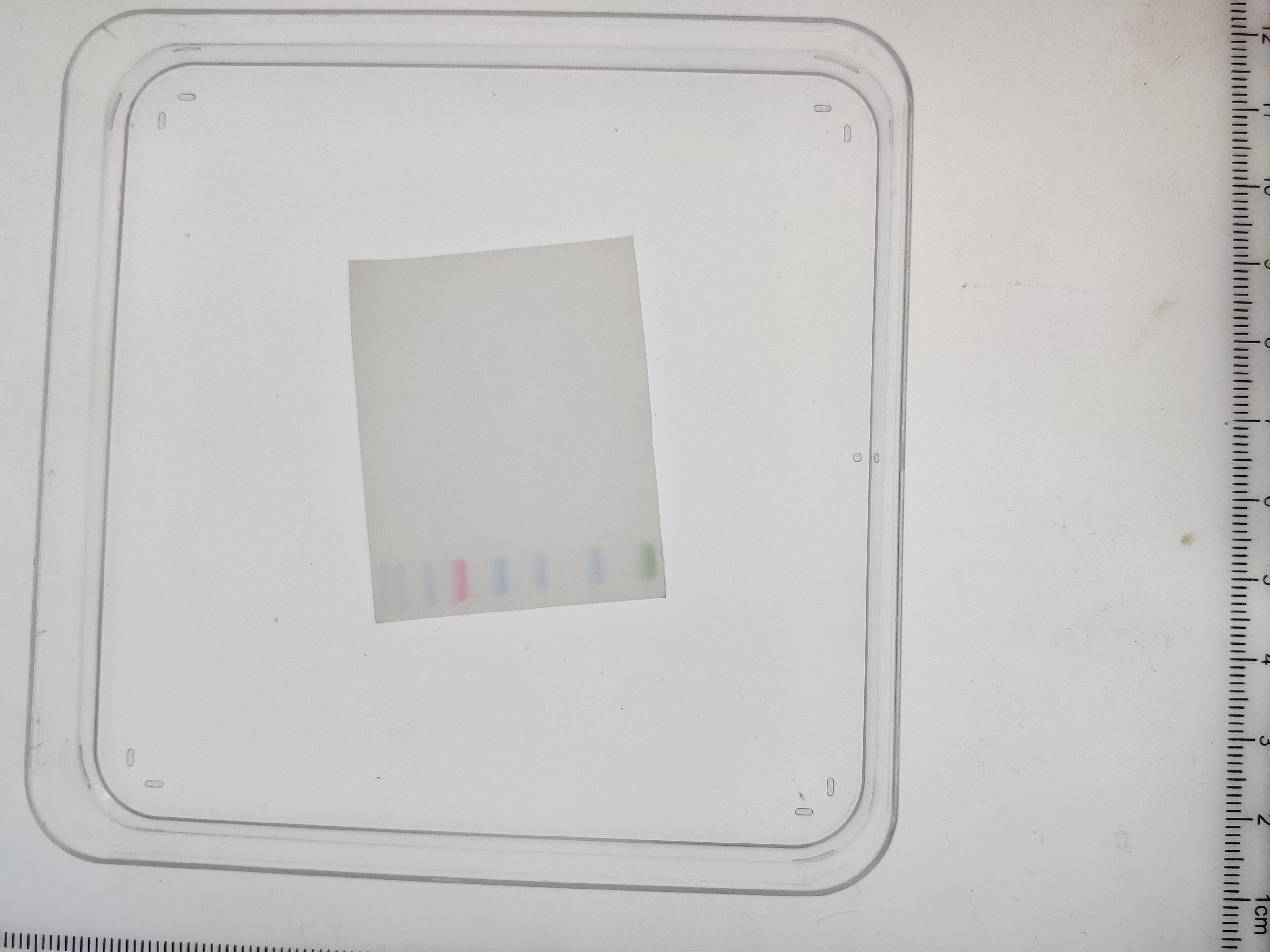

Supplement: Supplementary file 1 [file cimb-47-00936-s001.zip › cimb-3956315-supplementary/APOC2_ccRCC_RawWB_FullMembranes/Uncropped, unprocessed full-length Western blot images/10/10.jpg]

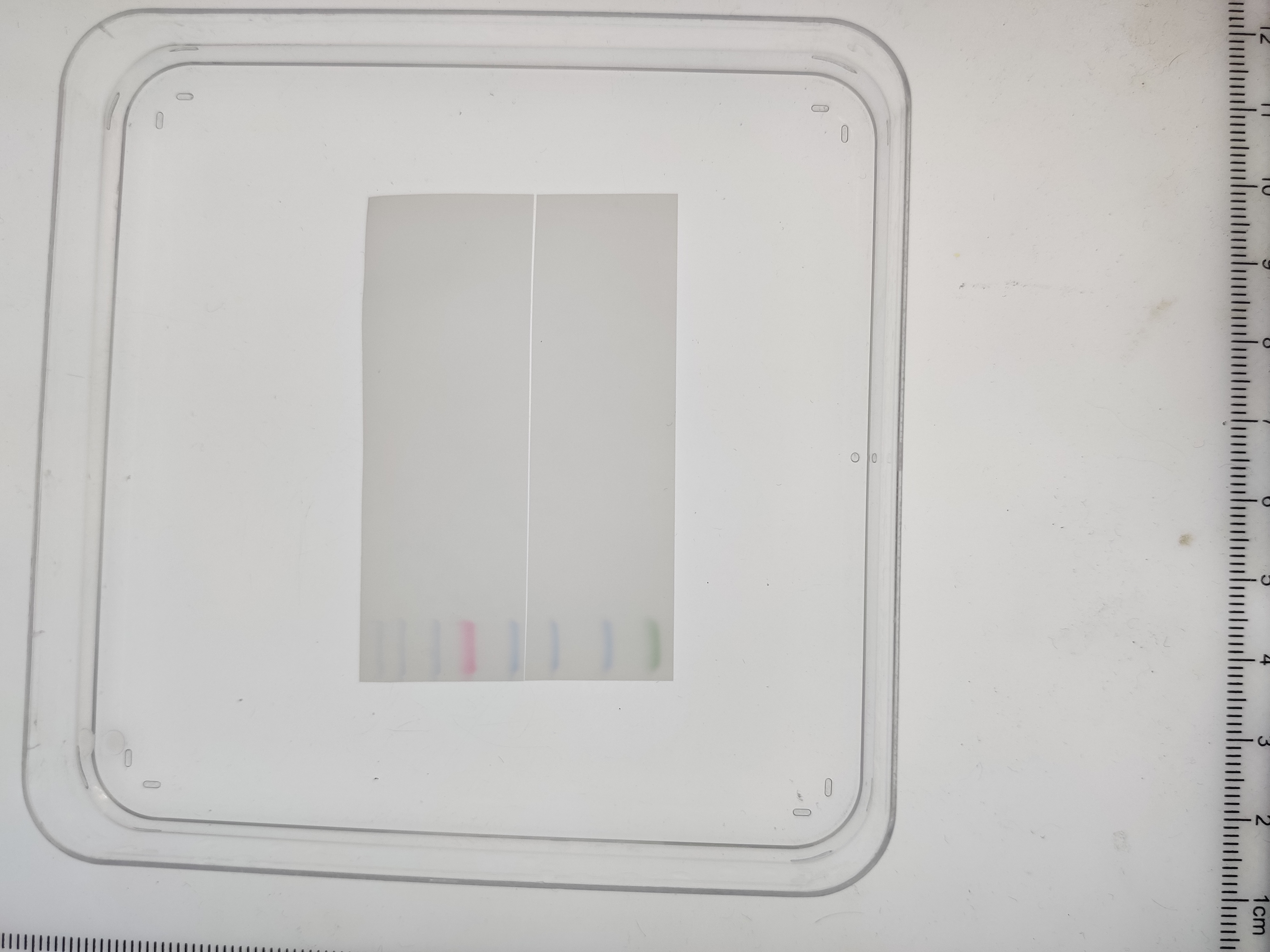

Supplement: Supplementary file 1 [file cimb-47-00936-s001.zip › cimb-3956315-supplementary/APOC2_ccRCC_RawWB_FullMembranes/Uncropped, unprocessed full-length Western blot images/11/11-.jpg]

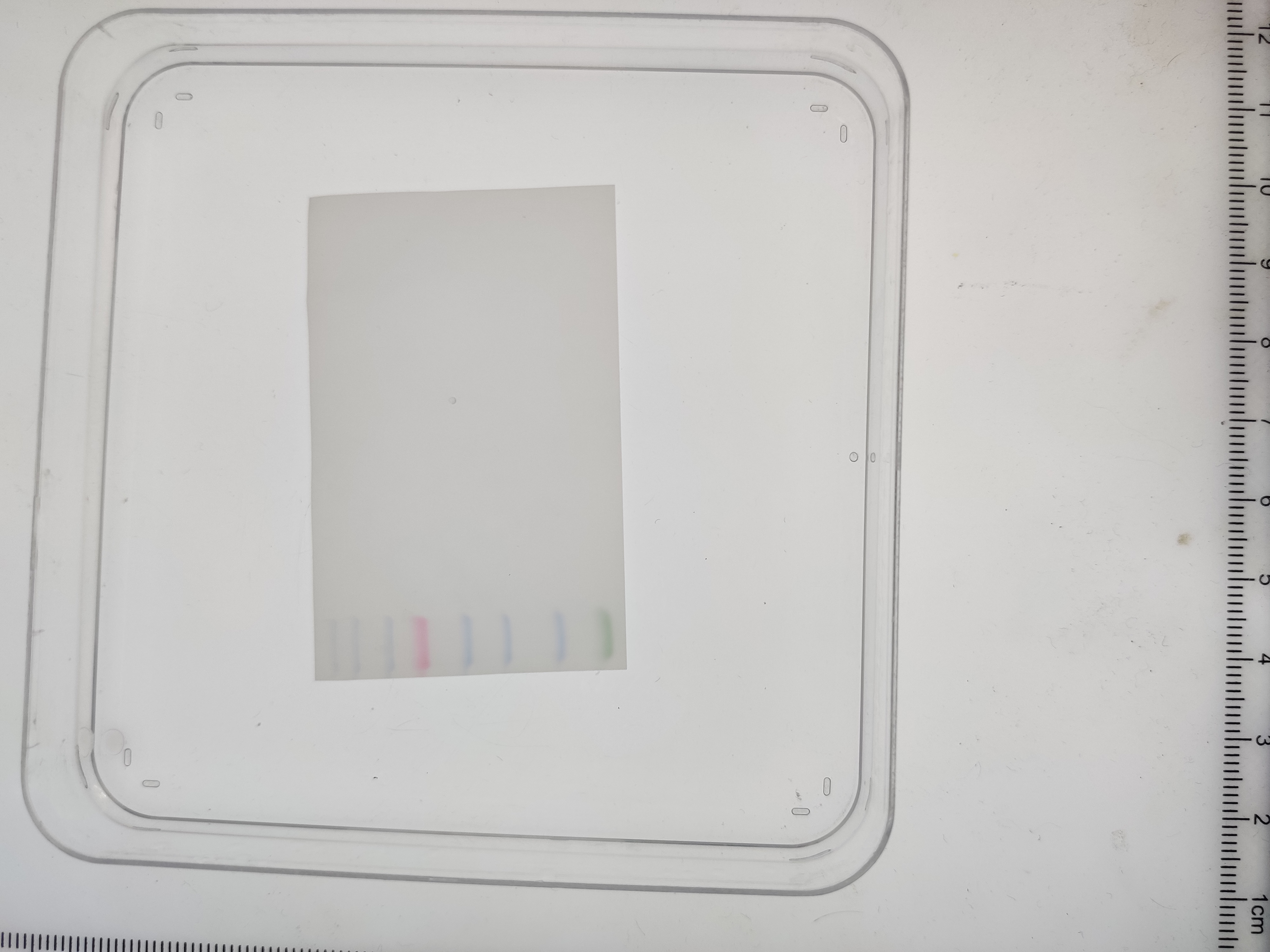

Supplement: Supplementary file 1 [file cimb-47-00936-s001.zip › cimb-3956315-supplementary/APOC2_ccRCC_RawWB_FullMembranes/Uncropped, unprocessed full-length Western blot images/11/11.jpg]

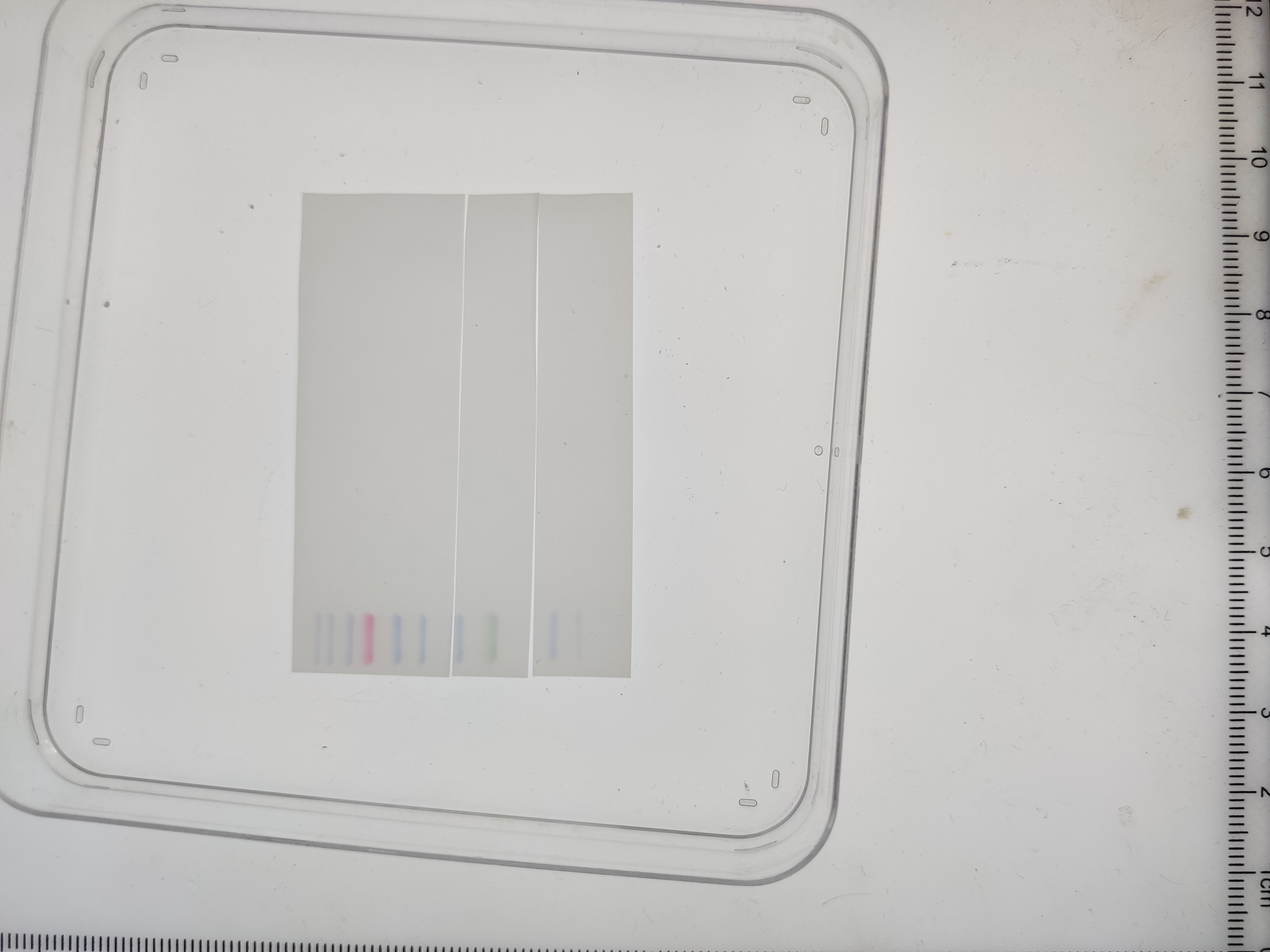

Supplement: Supplementary file 1 [file cimb-47-00936-s001.zip › cimb-3956315-supplementary/APOC2_ccRCC_RawWB_FullMembranes/Uncropped, unprocessed full-length Western blot images/12/12-.jpg]

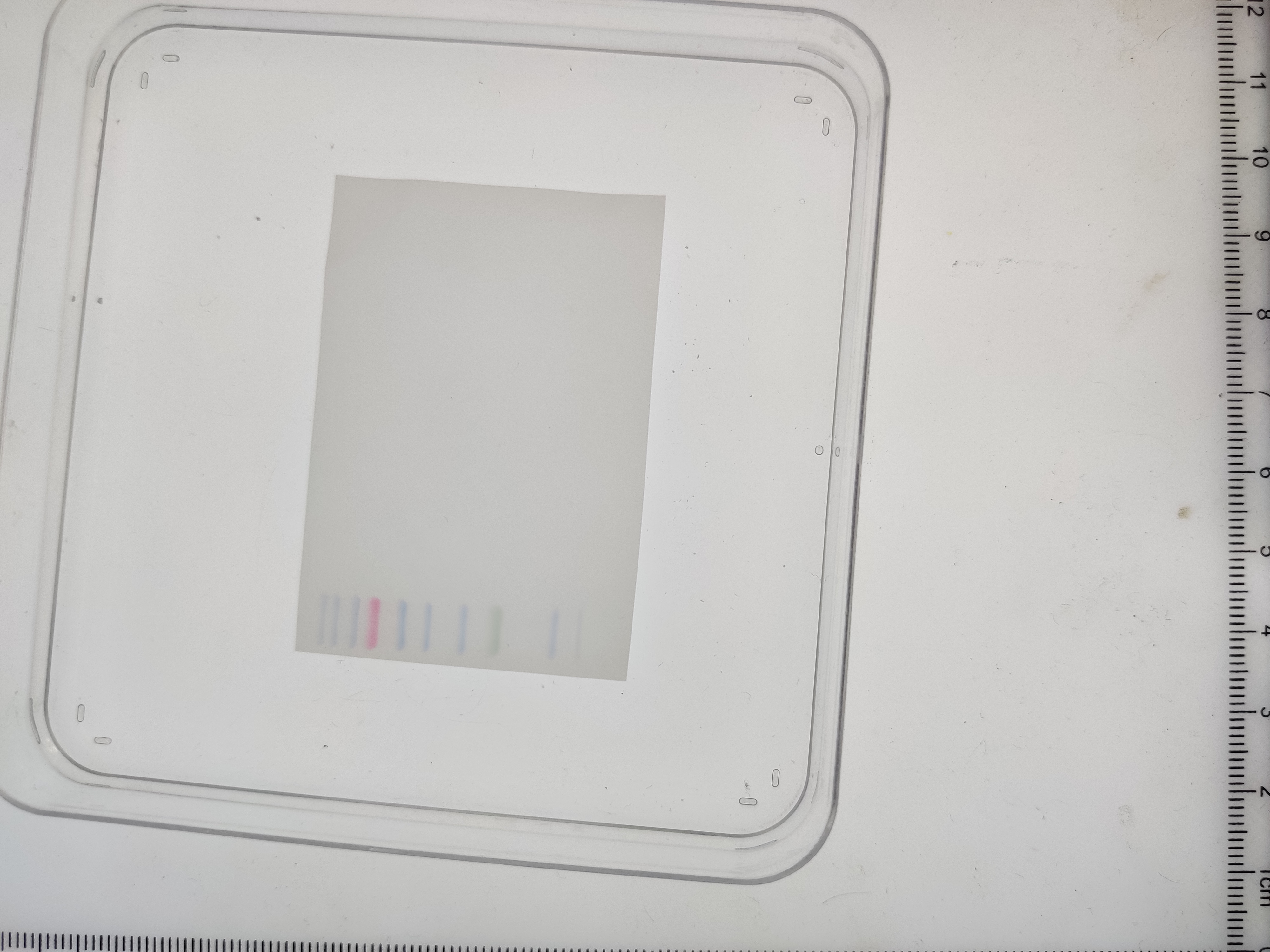

Supplement: Supplementary file 1 [file cimb-47-00936-s001.zip › cimb-3956315-supplementary/APOC2_ccRCC_RawWB_FullMembranes/Uncropped, unprocessed full-length Western blot images/12/12.jpg]

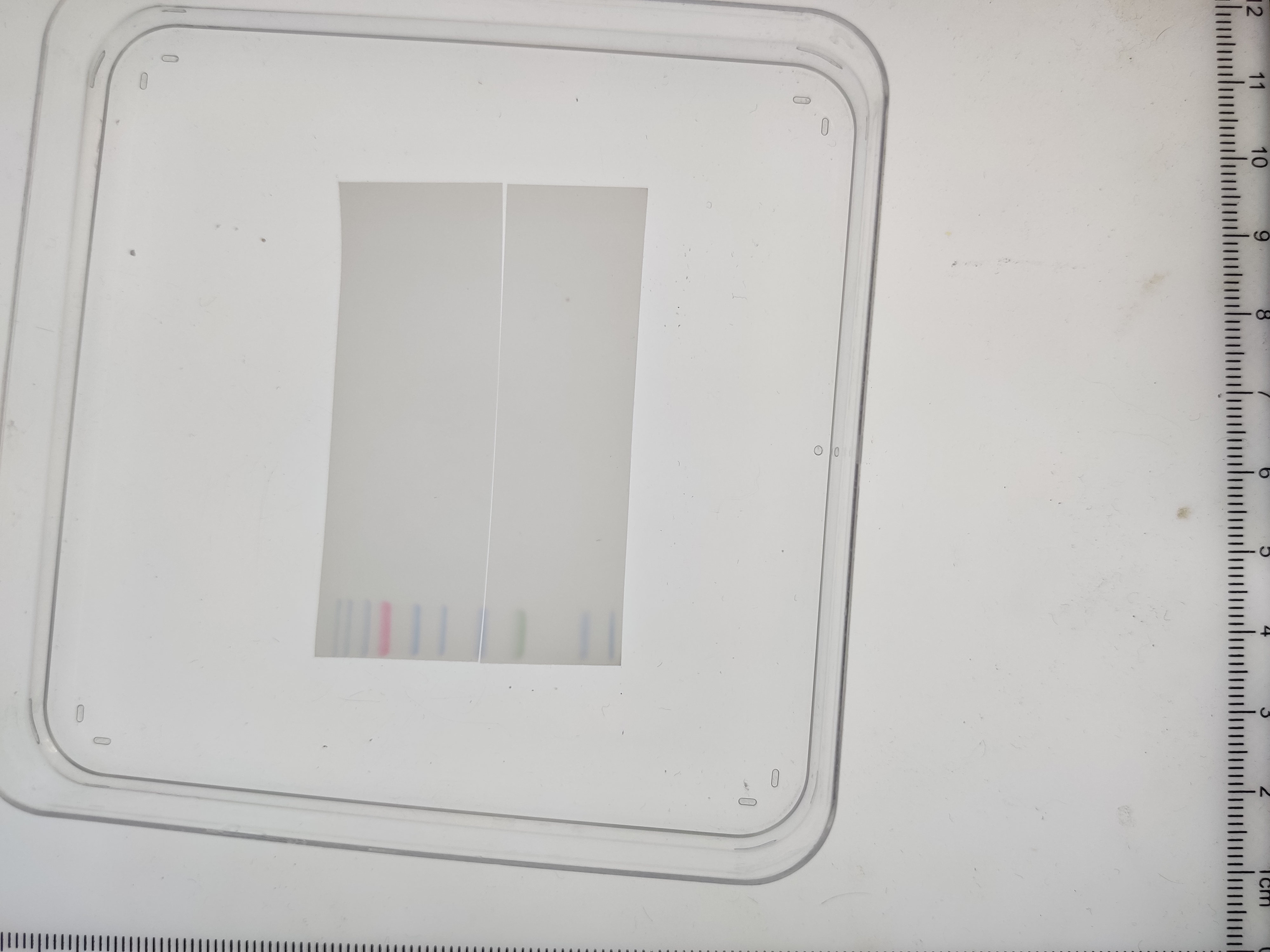

Supplement: Supplementary file 1 [file cimb-47-00936-s001.zip › cimb-3956315-supplementary/APOC2_ccRCC_RawWB_FullMembranes/Uncropped, unprocessed full-length Western blot images/13/13-.jpg]

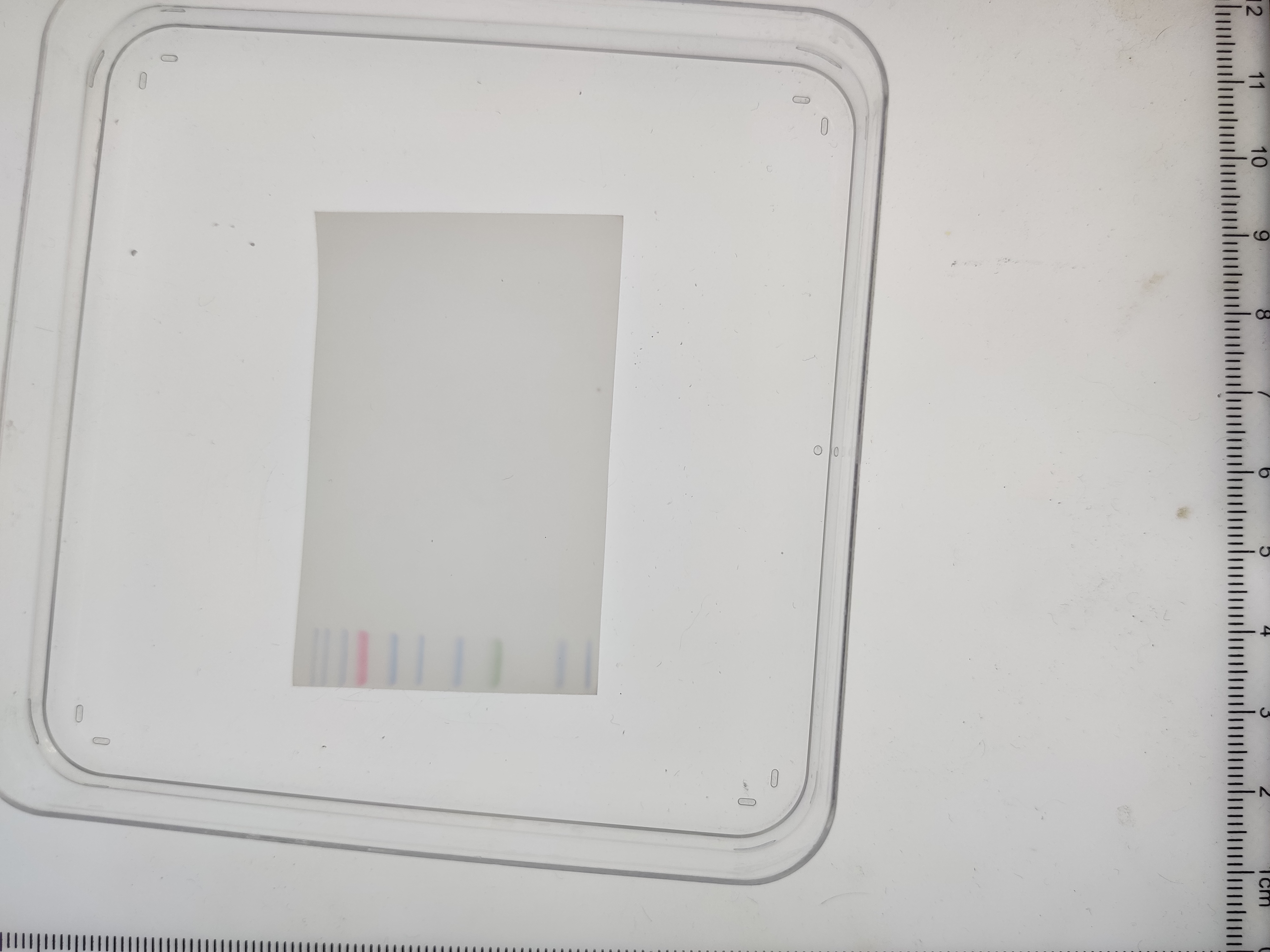

Supplement: Supplementary file 1 [file cimb-47-00936-s001.zip › cimb-3956315-supplementary/APOC2_ccRCC_RawWB_FullMembranes/Uncropped, unprocessed full-length Western blot images/13/13.jpg]
