# Supplementary figures and images for: APOC2 Promotes Clear Cell Renal Cell Carcinoma Progression via Activation of the JAK-STAT Signaling Pathway (part 1 of 2)
Source: Curr Issues Mol Biol. 2025 Nov 11;47(11):936. doi: 10.3390/cimb47110936 (PMC12651258; doi:10.3390/cimb47110936)

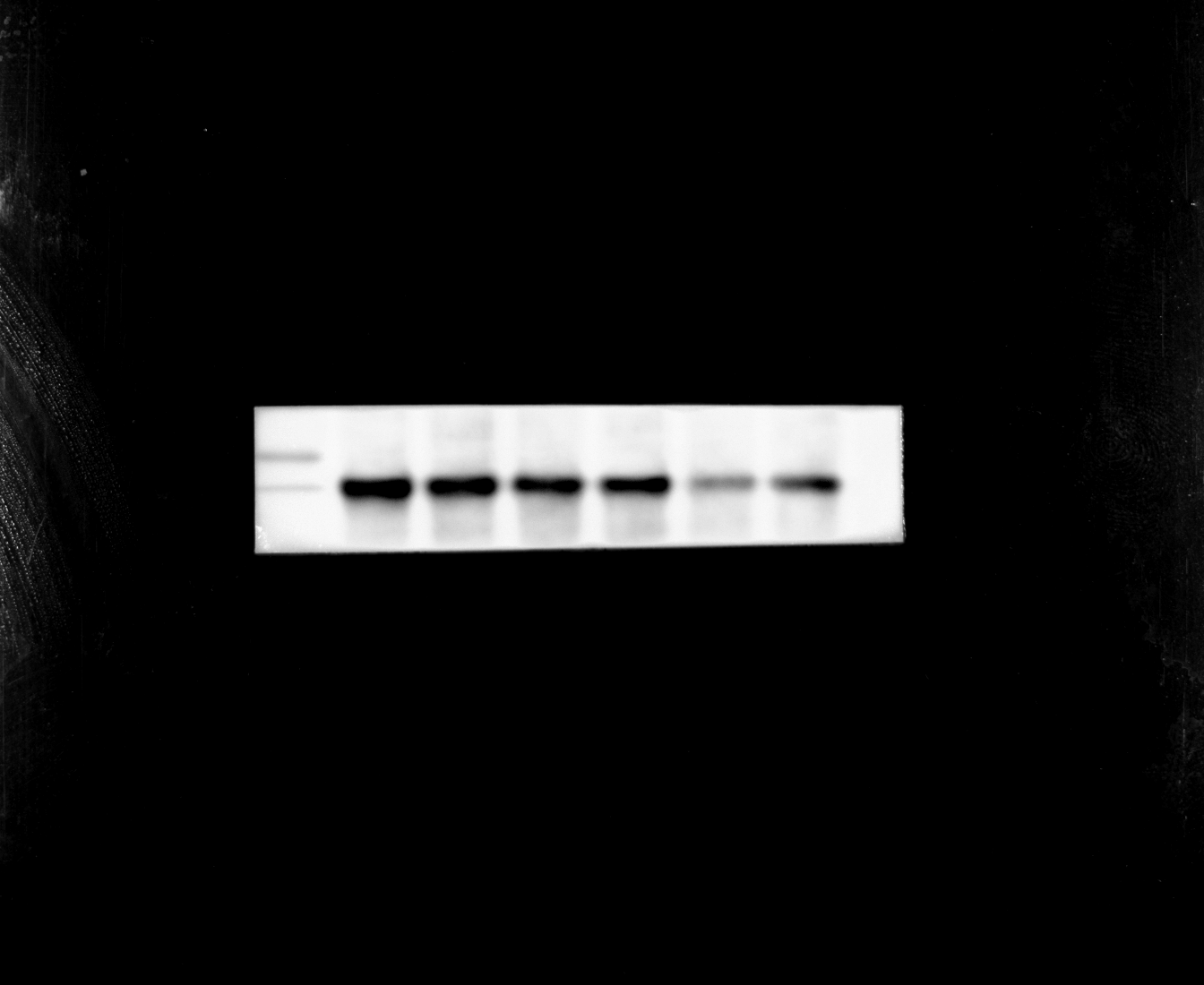

Supplement: Supplementary file 1 [file cimb-47-00936-s001.zip › cimb-3956315-supplementary/APOC2_ccRCC_RawWB_FullMembranes/cropped display images/1/Fig 1E APOC2/0.Tif]

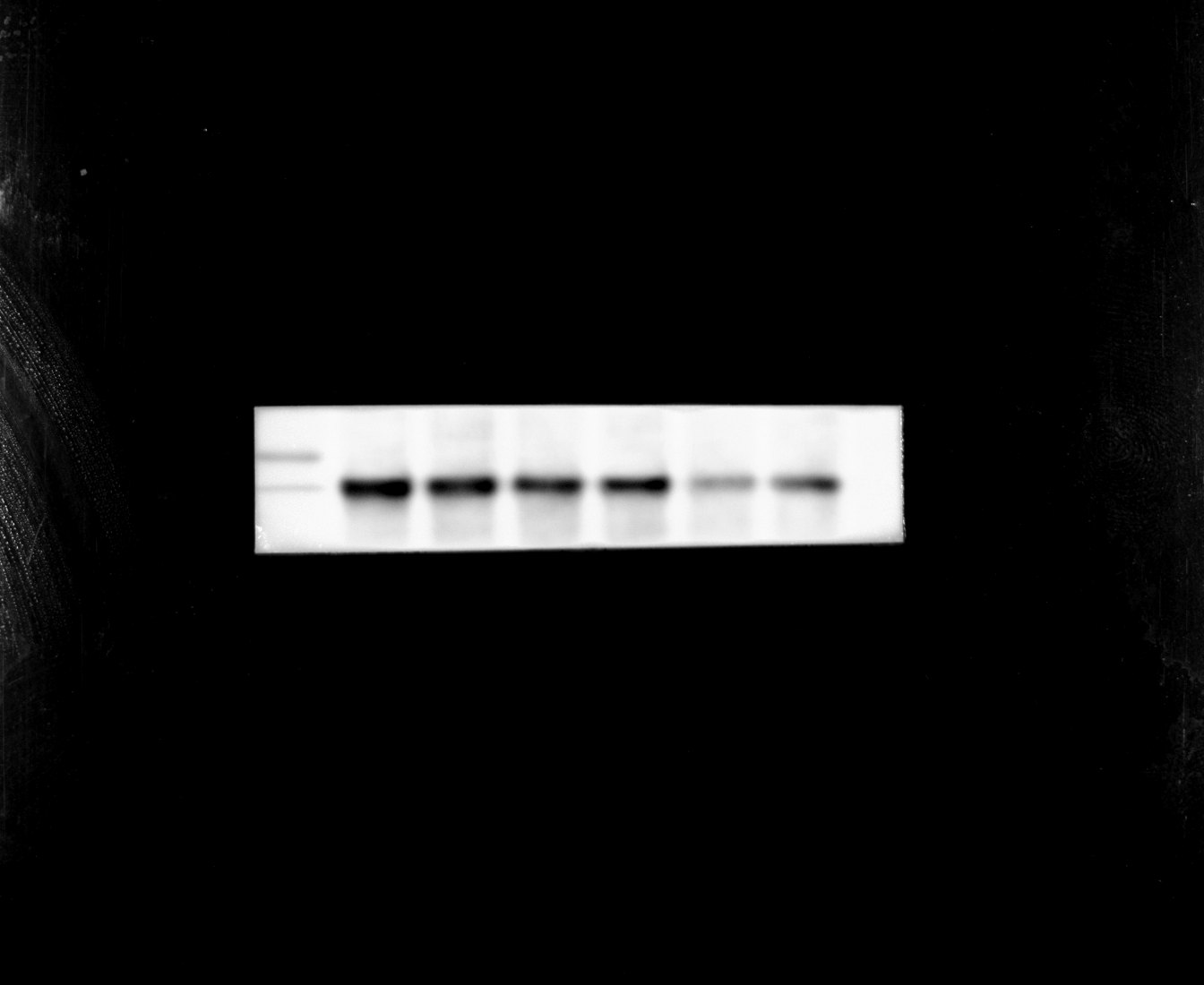

Supplement: Supplementary file 1 [file cimb-47-00936-s001.zip › cimb-3956315-supplementary/APOC2_ccRCC_RawWB_FullMembranes/cropped display images/1/Fig 1E APOC2/1.Tif]

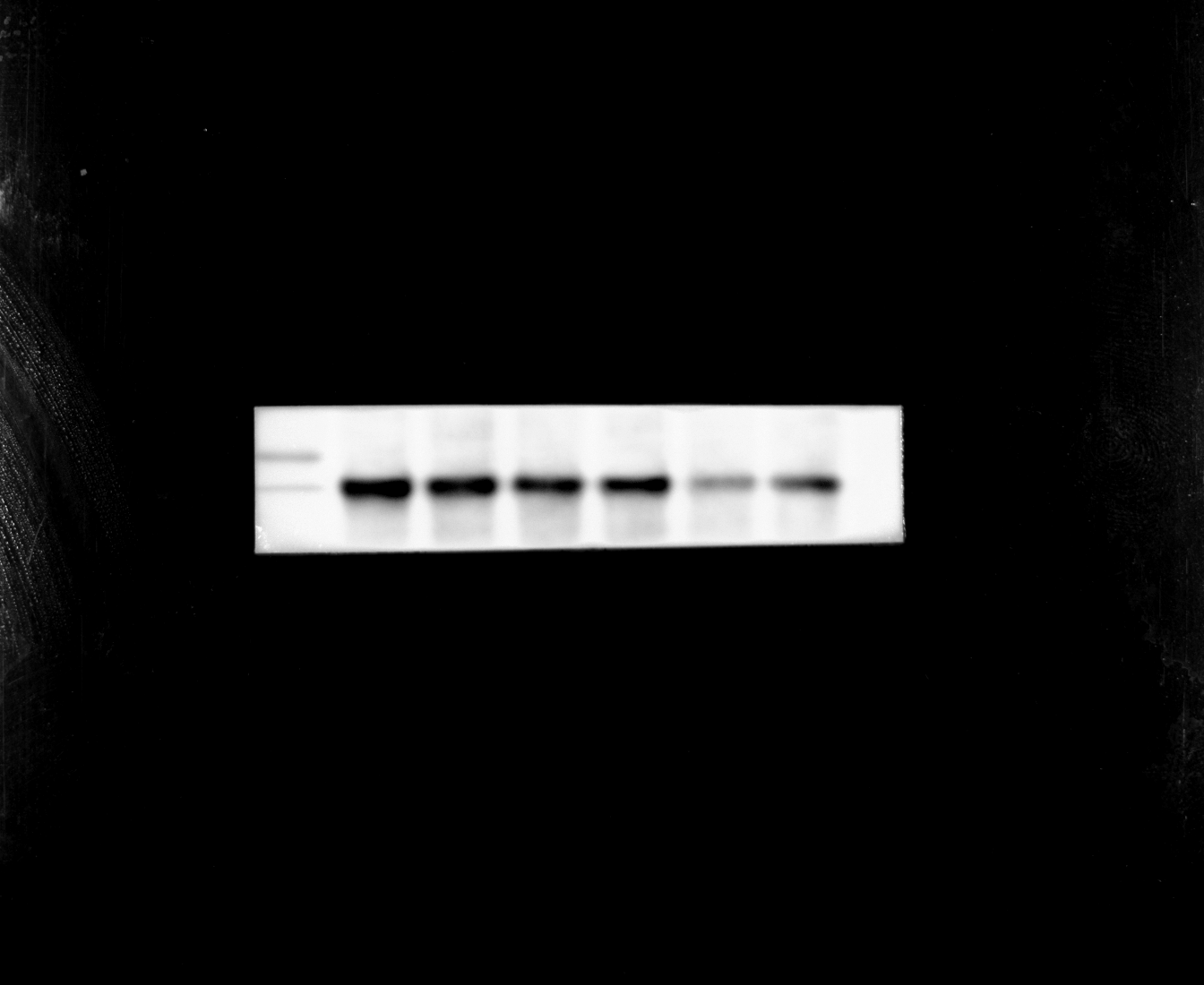

Supplement: Supplementary file 1 [file cimb-47-00936-s001.zip › cimb-3956315-supplementary/APOC2_ccRCC_RawWB_FullMembranes/cropped display images/1/Fig 1E APOC2/2.Tif]

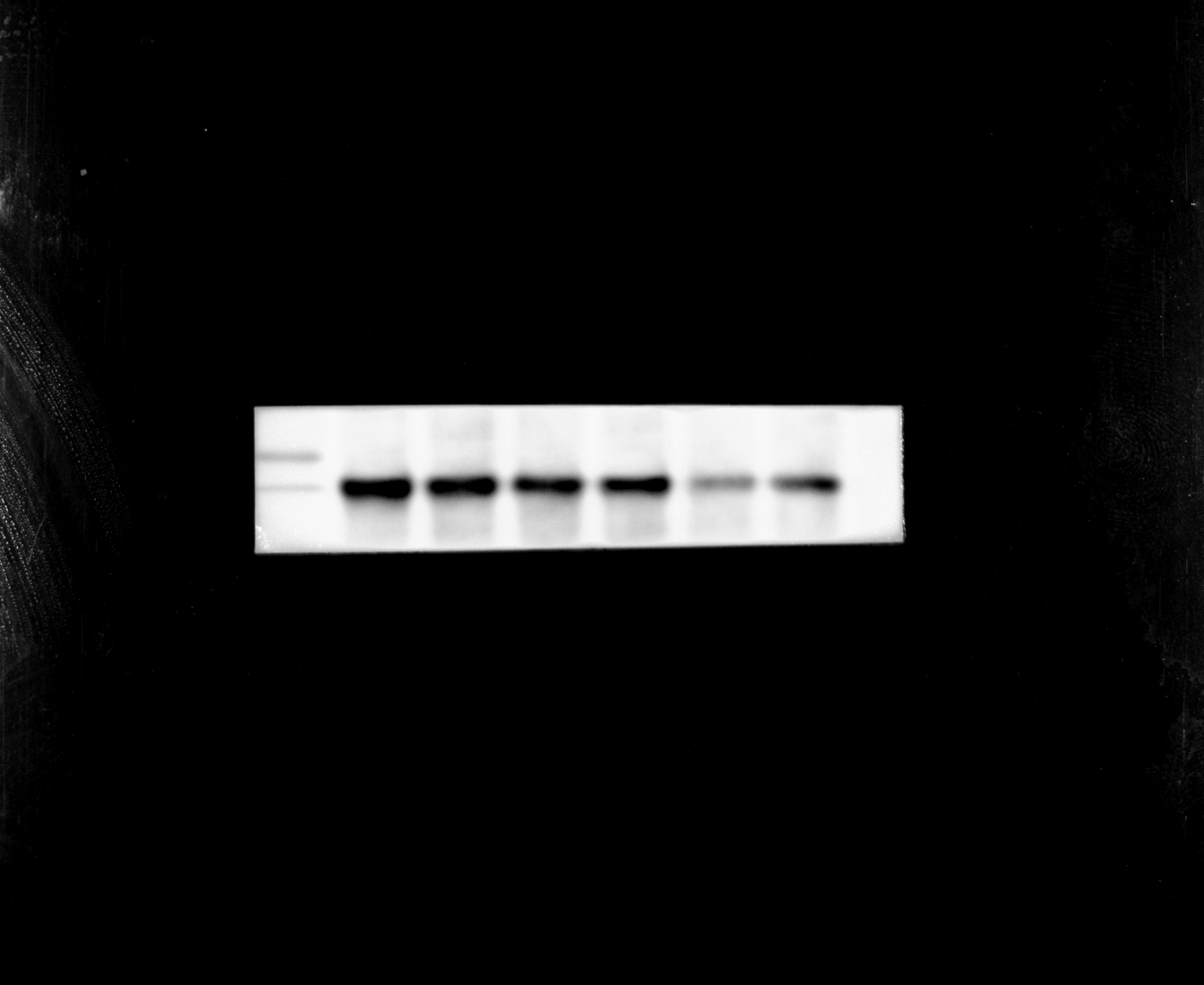

Supplement: Supplementary file 1 [file cimb-47-00936-s001.zip › cimb-3956315-supplementary/APOC2_ccRCC_RawWB_FullMembranes/cropped display images/1/Fig 1E APOC2/3.Tif]

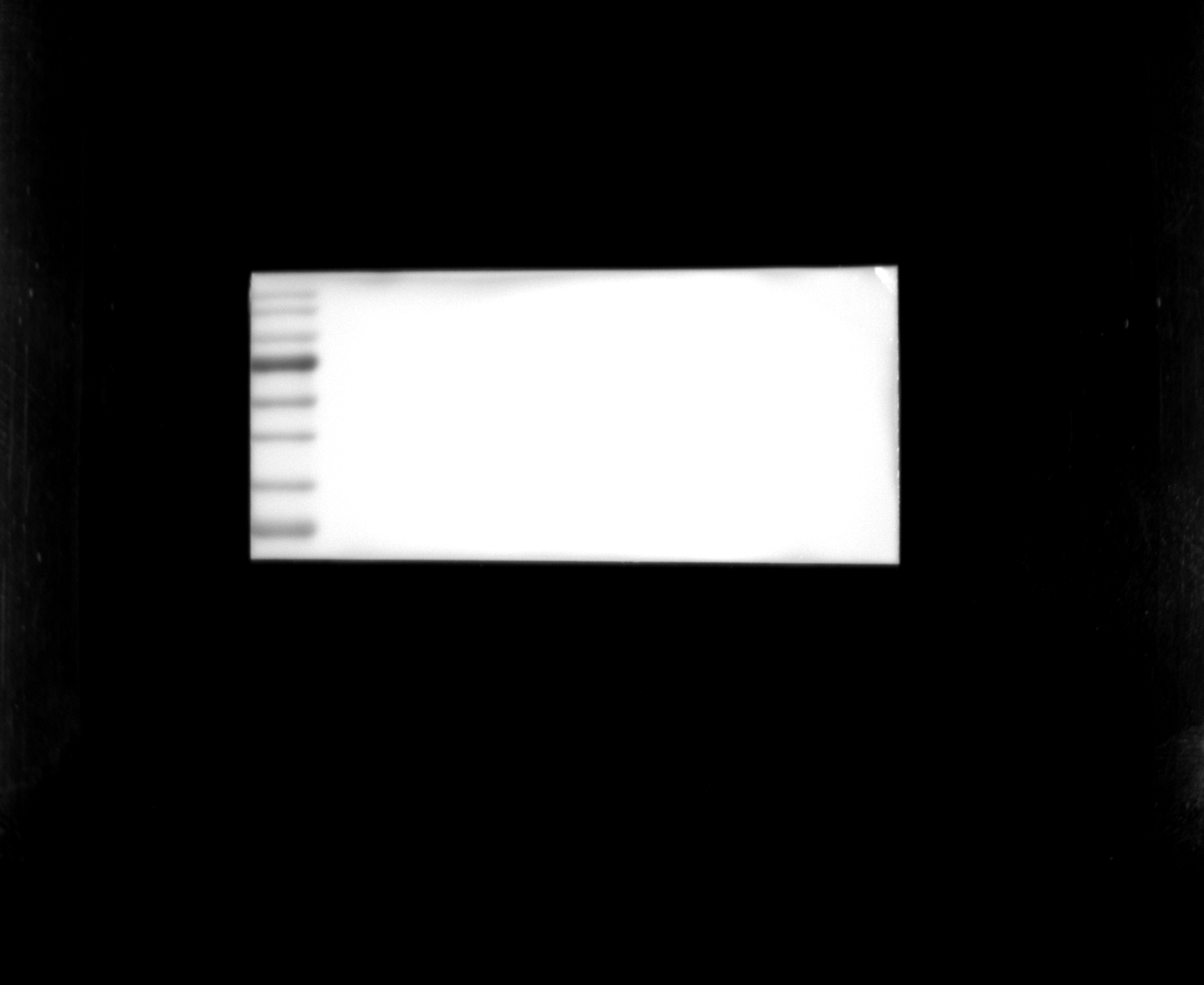

Supplement: Supplementary file 1 [file cimb-47-00936-s001.zip › cimb-3956315-supplementary/APOC2_ccRCC_RawWB_FullMembranes/cropped display images/1/Fig 1E GAPDH/0.Tif]

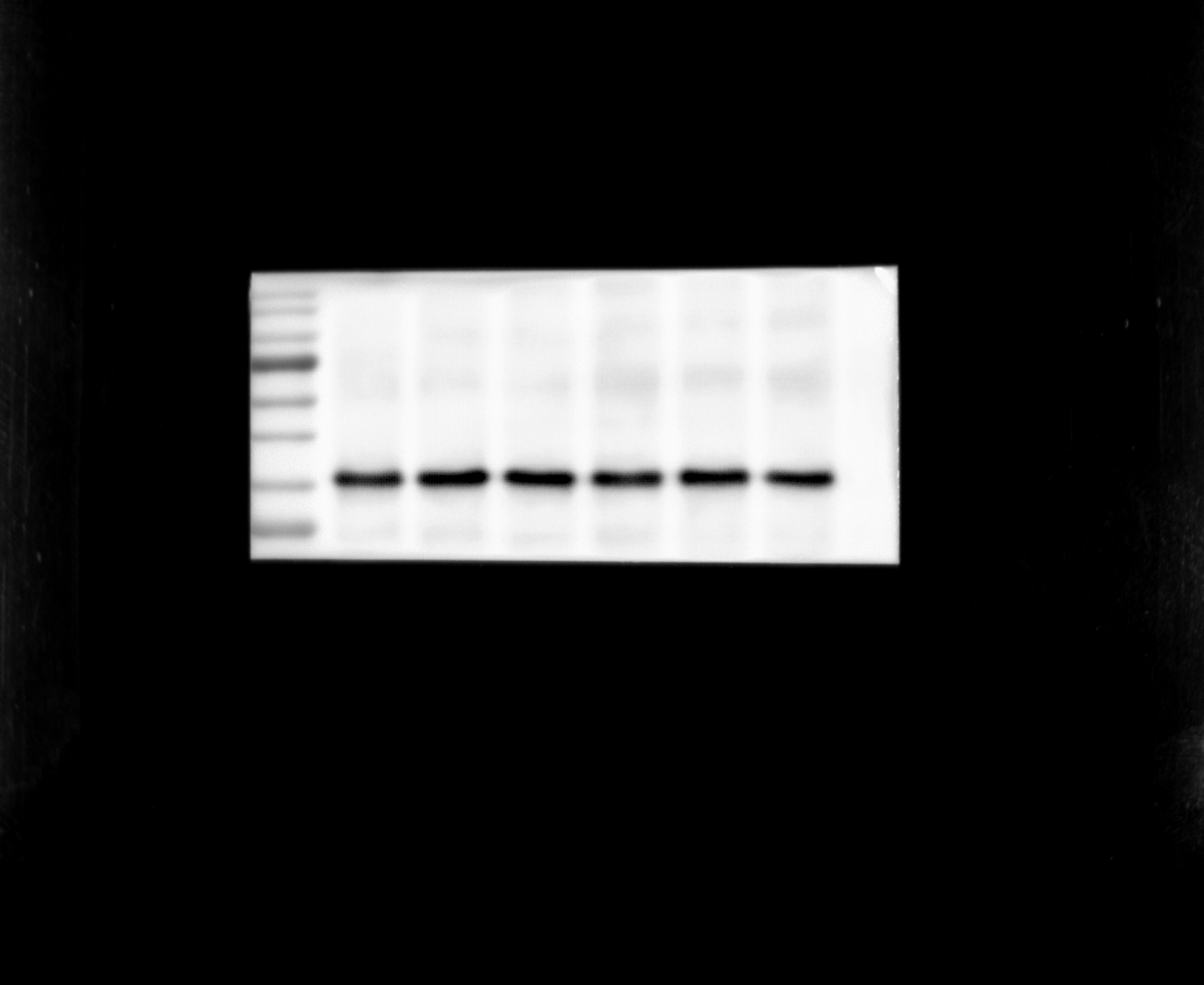

Supplement: Supplementary file 1 [file cimb-47-00936-s001.zip › cimb-3956315-supplementary/APOC2_ccRCC_RawWB_FullMembranes/cropped display images/1/Fig 1E GAPDH/1.Tif]

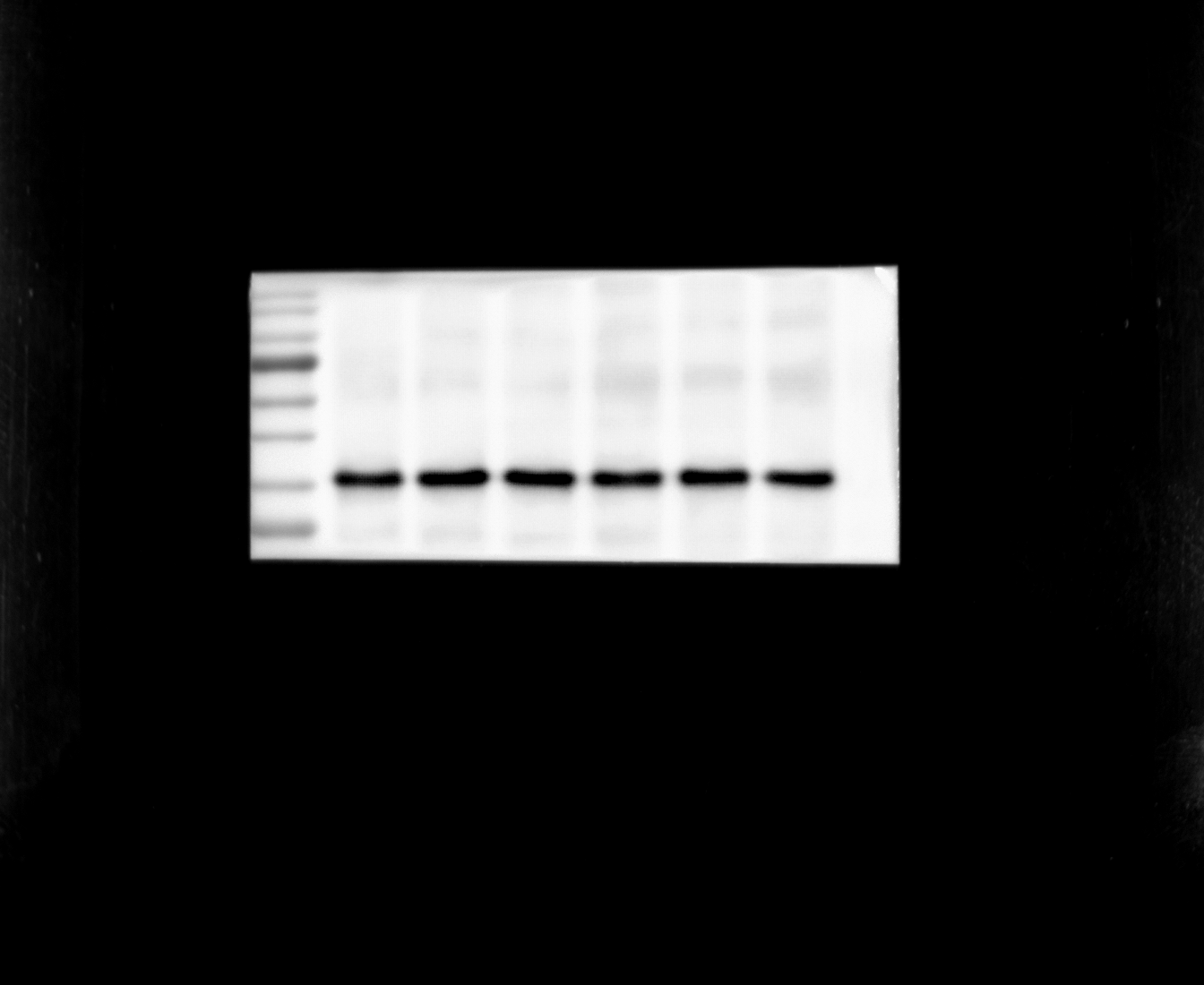

Supplement: Supplementary file 1 [file cimb-47-00936-s001.zip › cimb-3956315-supplementary/APOC2_ccRCC_RawWB_FullMembranes/cropped display images/1/Fig 1E GAPDH/2.Tif]

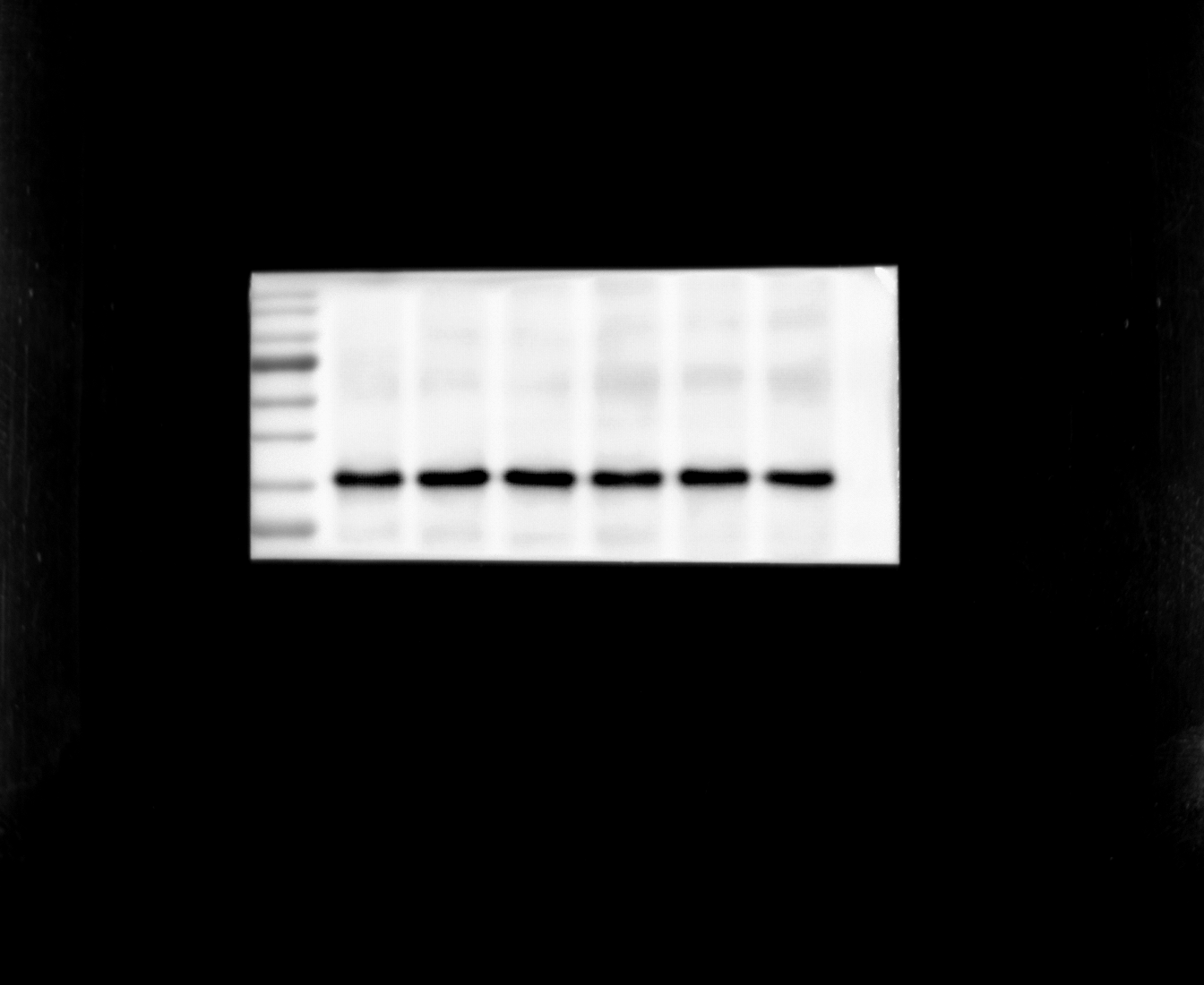

Supplement: Supplementary file 1 [file cimb-47-00936-s001.zip › cimb-3956315-supplementary/APOC2_ccRCC_RawWB_FullMembranes/cropped display images/1/Fig 1E GAPDH/3.Tif]

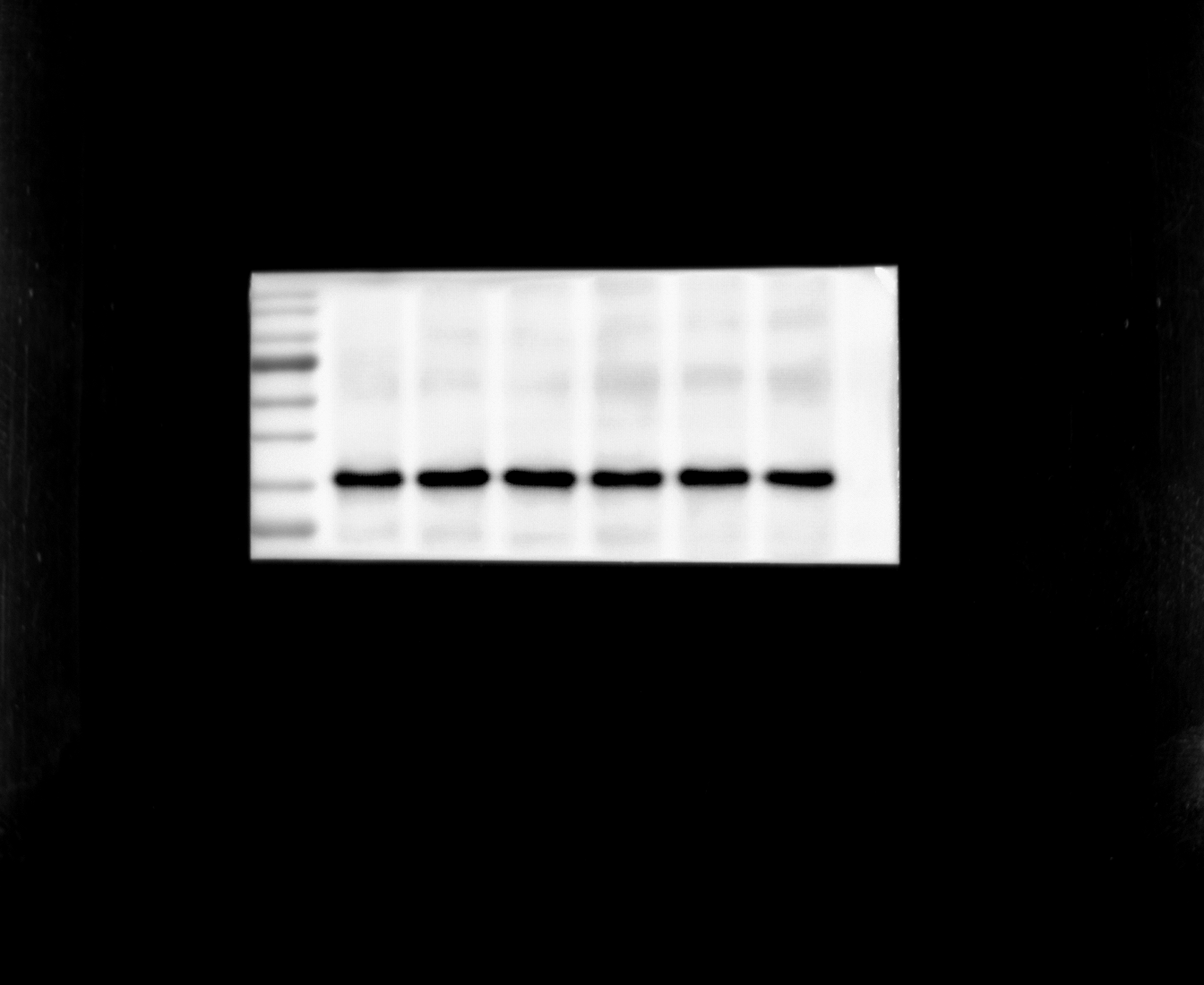

Supplement: Supplementary file 1 [file cimb-47-00936-s001.zip › cimb-3956315-supplementary/APOC2_ccRCC_RawWB_FullMembranes/cropped display images/1/Fig 1E GAPDH/4.Tif]

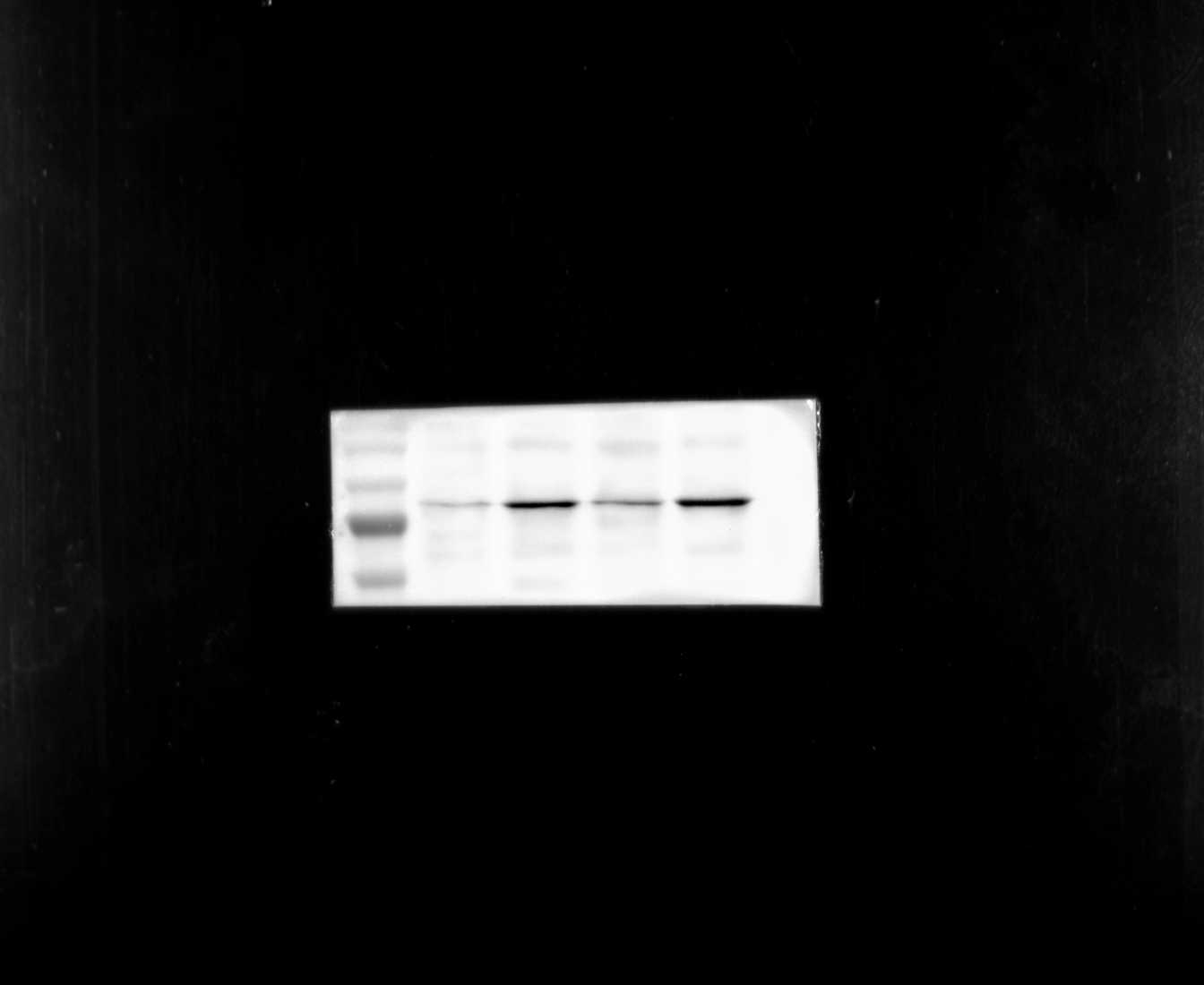

Supplement: Supplementary file 1 [file cimb-47-00936-s001.zip › cimb-3956315-supplementary/APOC2_ccRCC_RawWB_FullMembranes/cropped display images/10/Fig 3D p-stat3/1.Tif]

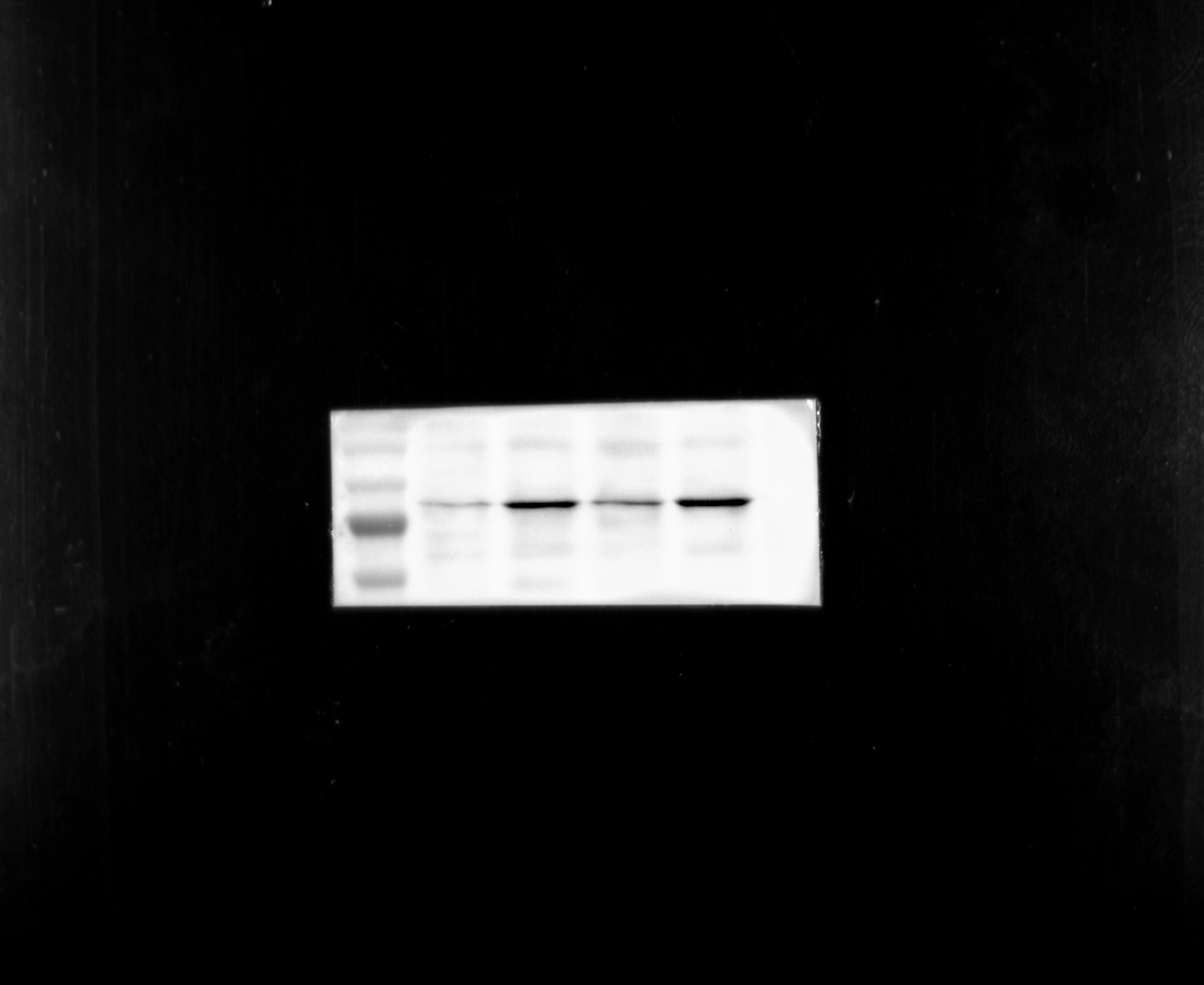

Supplement: Supplementary file 1 [file cimb-47-00936-s001.zip › cimb-3956315-supplementary/APOC2_ccRCC_RawWB_FullMembranes/cropped display images/10/Fig 3D p-stat3/2.Tif]

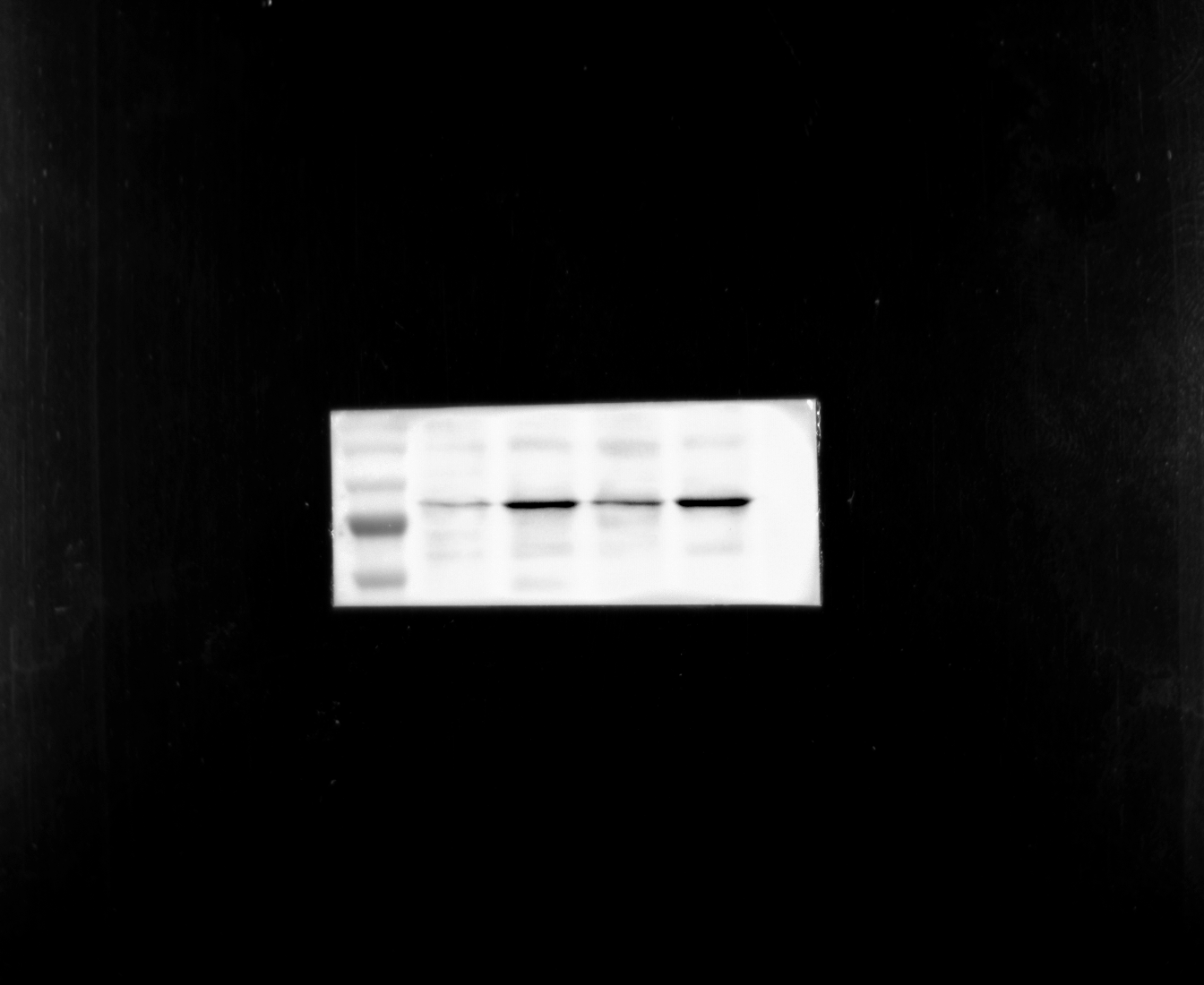

Supplement: Supplementary file 1 [file cimb-47-00936-s001.zip › cimb-3956315-supplementary/APOC2_ccRCC_RawWB_FullMembranes/cropped display images/10/Fig 3D p-stat3/3.Tif]

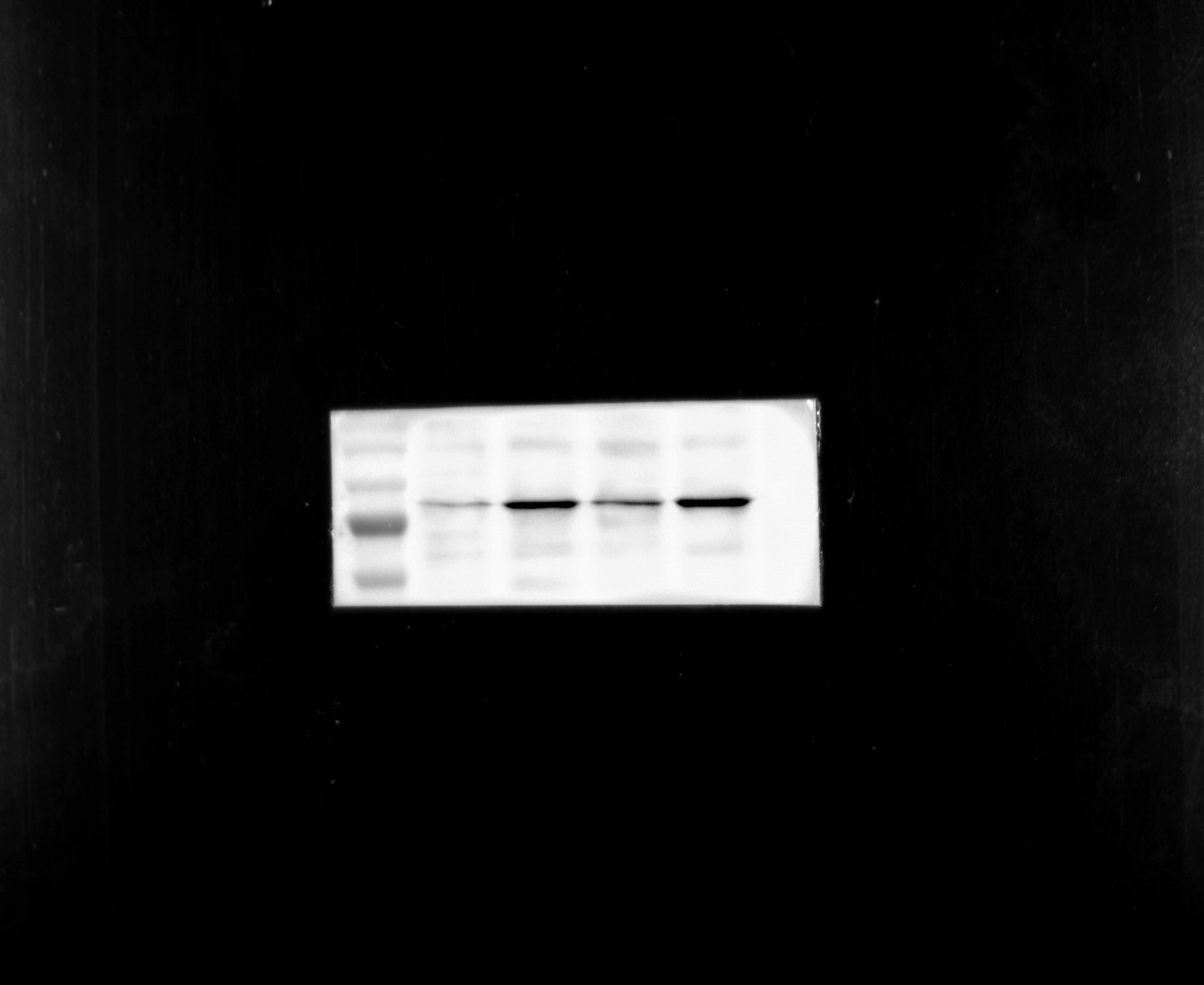

Supplement: Supplementary file 1 [file cimb-47-00936-s001.zip › cimb-3956315-supplementary/APOC2_ccRCC_RawWB_FullMembranes/cropped display images/10/Fig 3D p-stat3/4.Tif]

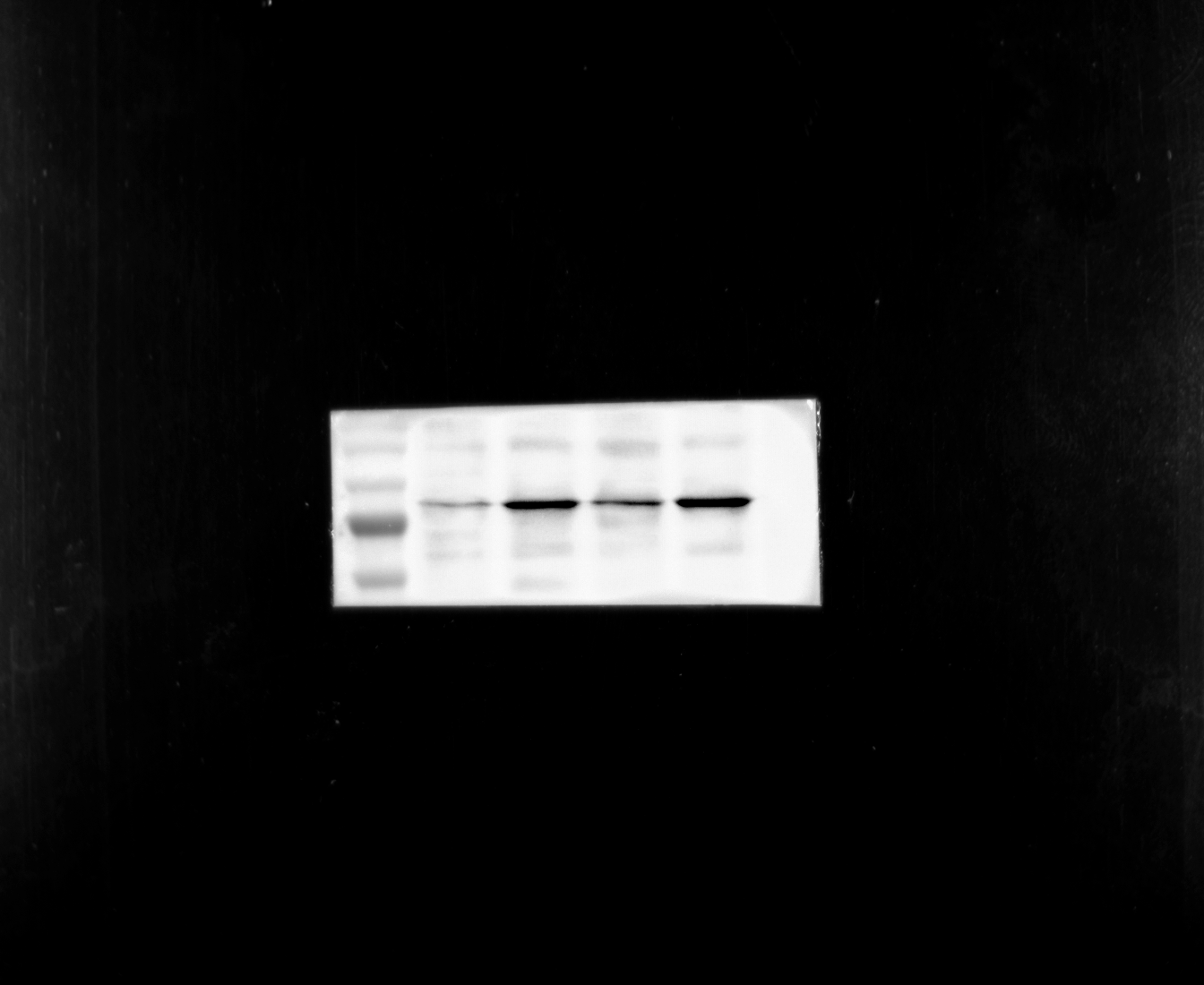

Supplement: Supplementary file 1 [file cimb-47-00936-s001.zip › cimb-3956315-supplementary/APOC2_ccRCC_RawWB_FullMembranes/cropped display images/10/Fig 3D p-stat3/5.Tif]

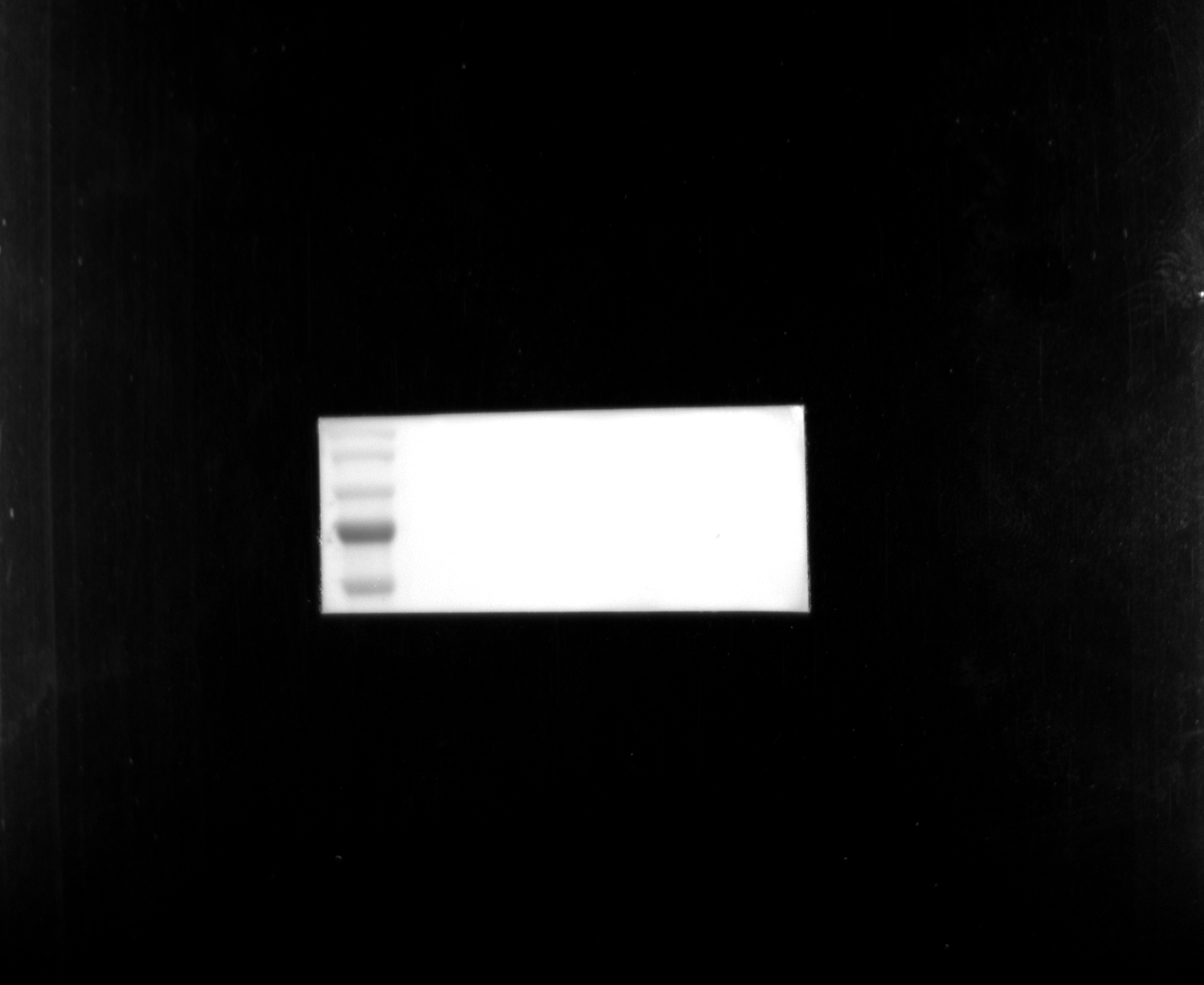

Supplement: Supplementary file 1 [file cimb-47-00936-s001.zip › cimb-3956315-supplementary/APOC2_ccRCC_RawWB_FullMembranes/cropped display images/10/Fig 3D stat3/0.Tif]

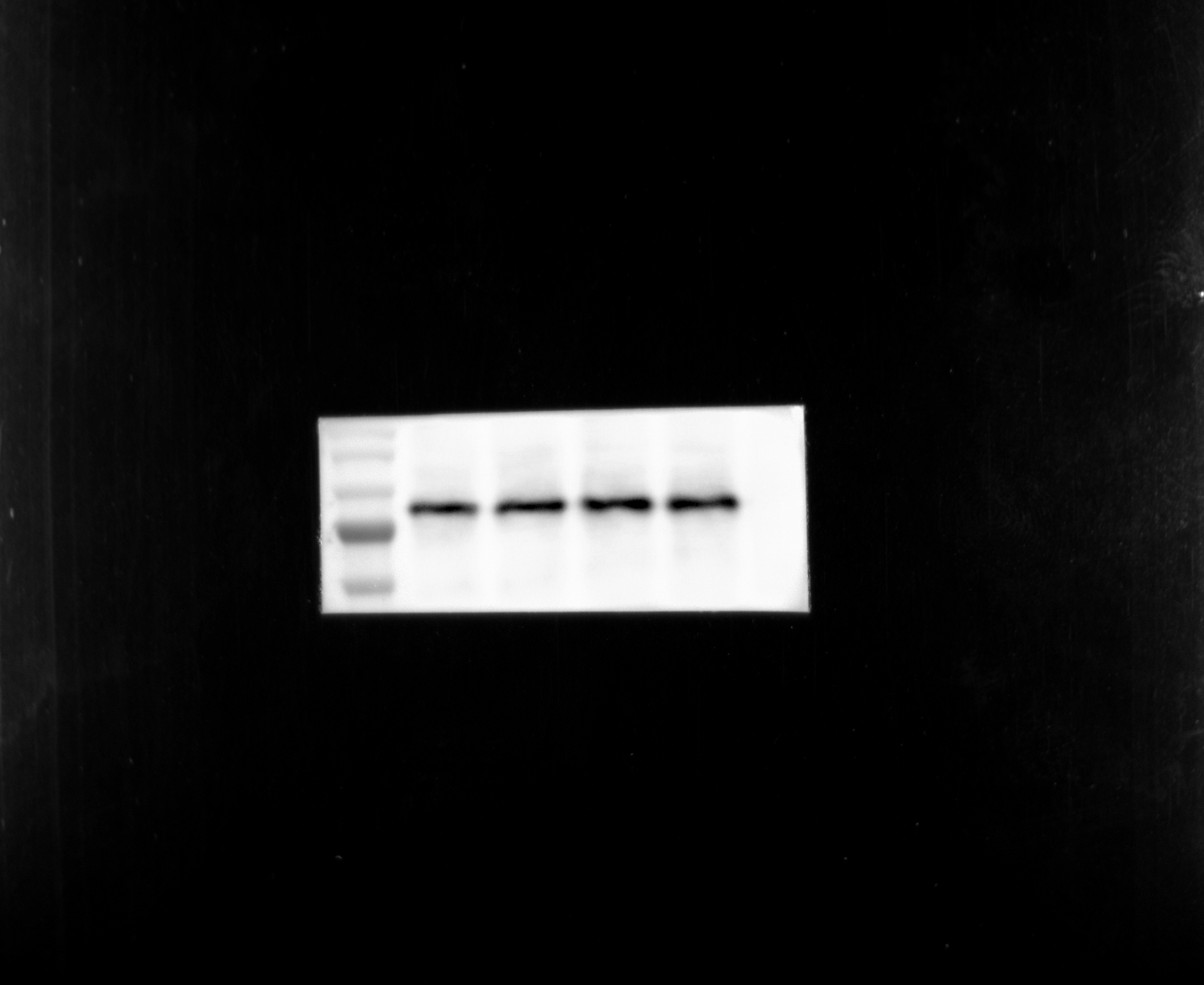

Supplement: Supplementary file 1 [file cimb-47-00936-s001.zip › cimb-3956315-supplementary/APOC2_ccRCC_RawWB_FullMembranes/cropped display images/10/Fig 3D stat3/1.Tif]

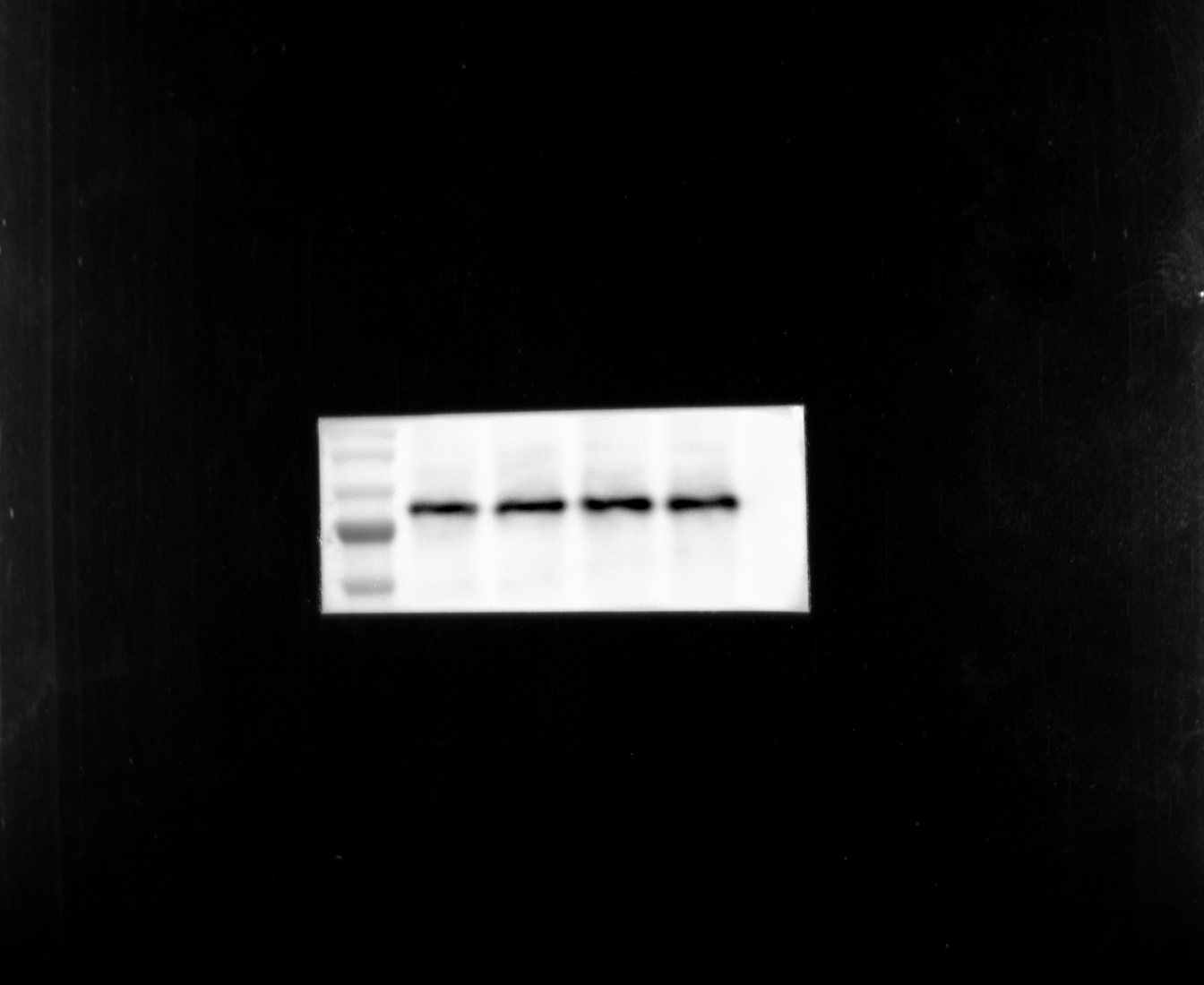

Supplement: Supplementary file 1 [file cimb-47-00936-s001.zip › cimb-3956315-supplementary/APOC2_ccRCC_RawWB_FullMembranes/cropped display images/10/Fig 3D stat3/2.Tif]

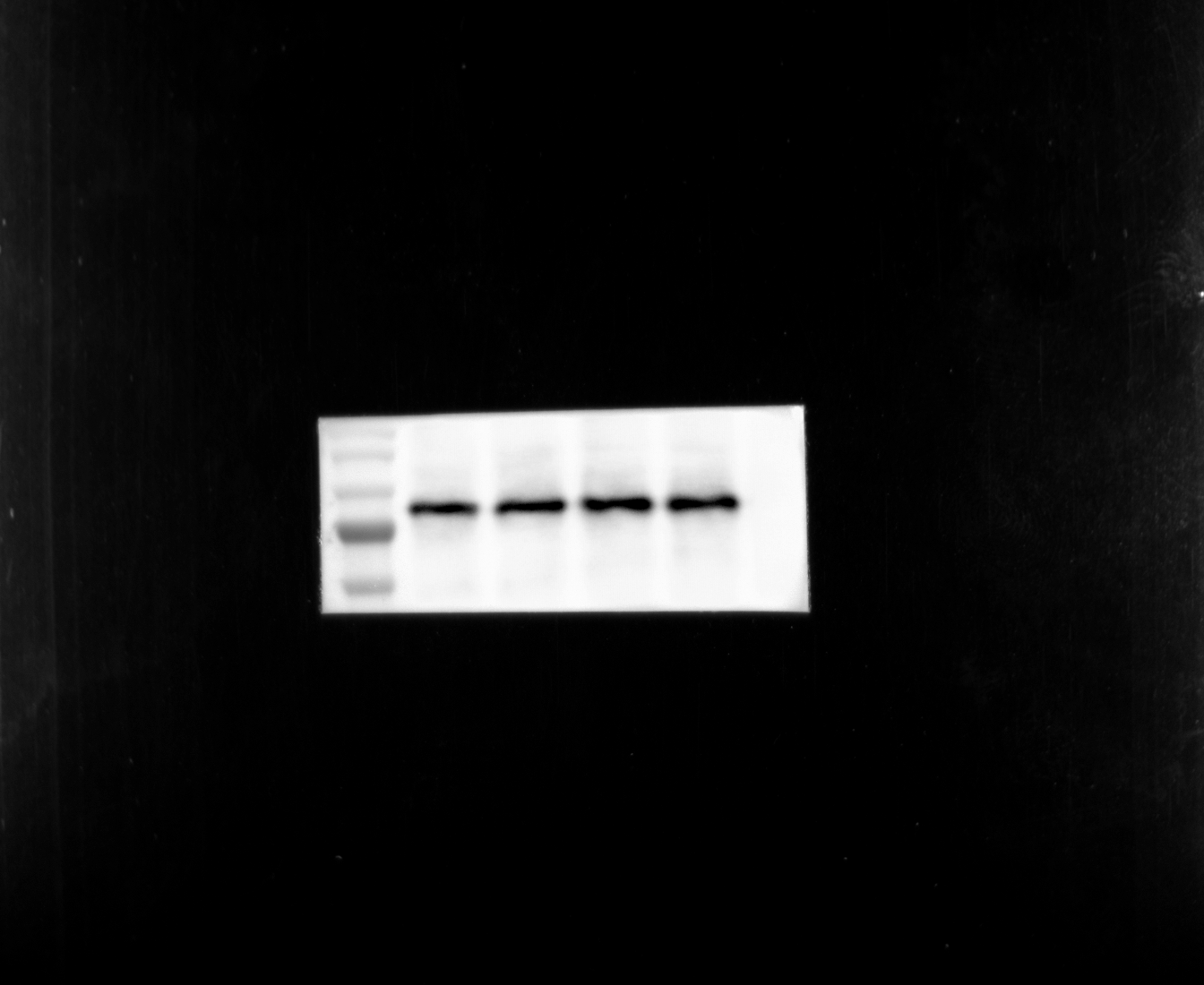

Supplement: Supplementary file 1 [file cimb-47-00936-s001.zip › cimb-3956315-supplementary/APOC2_ccRCC_RawWB_FullMembranes/cropped display images/10/Fig 3D stat3/3.Tif]

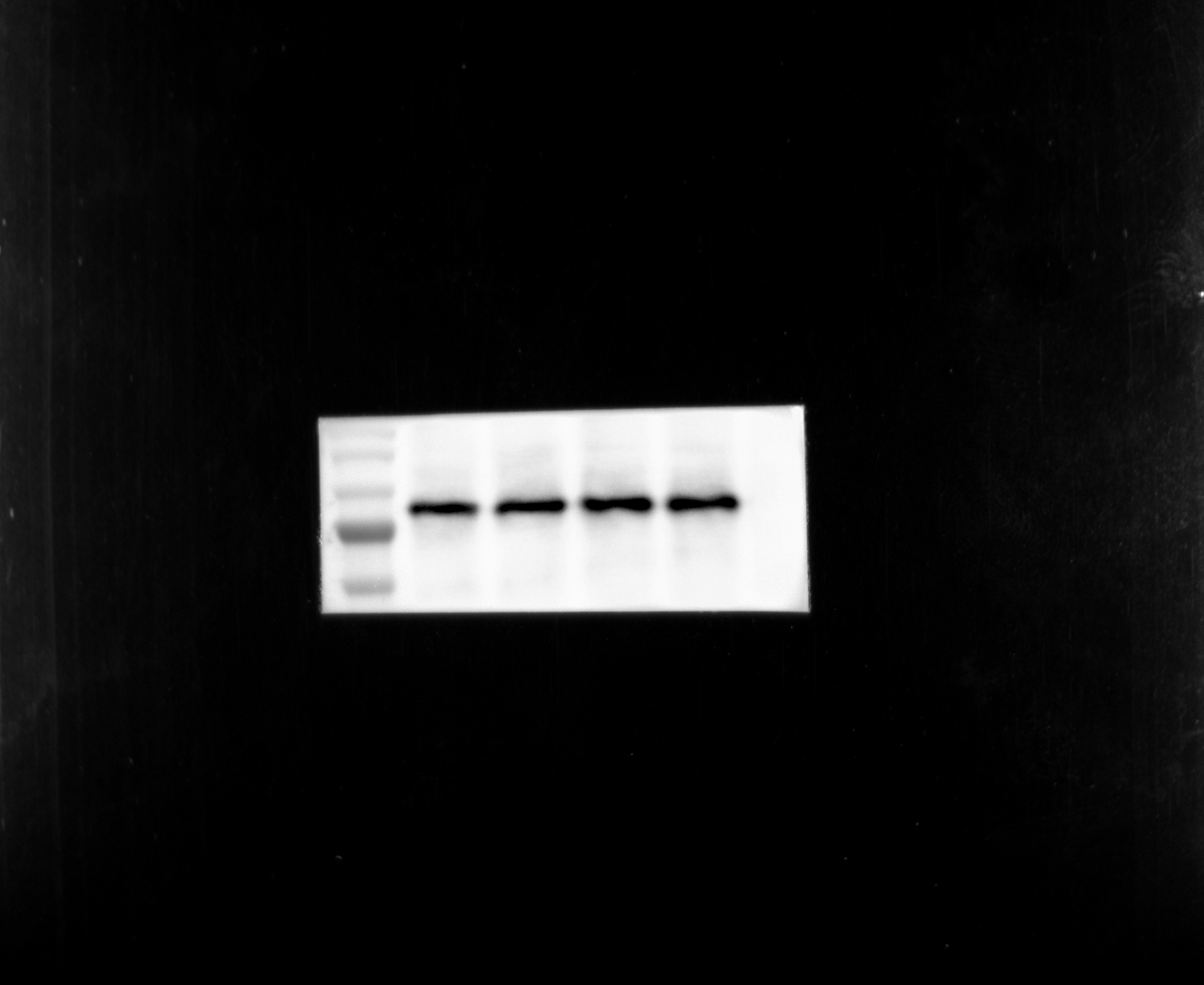

Supplement: Supplementary file 1 [file cimb-47-00936-s001.zip › cimb-3956315-supplementary/APOC2_ccRCC_RawWB_FullMembranes/cropped display images/10/Fig 3D stat3/4.Tif]

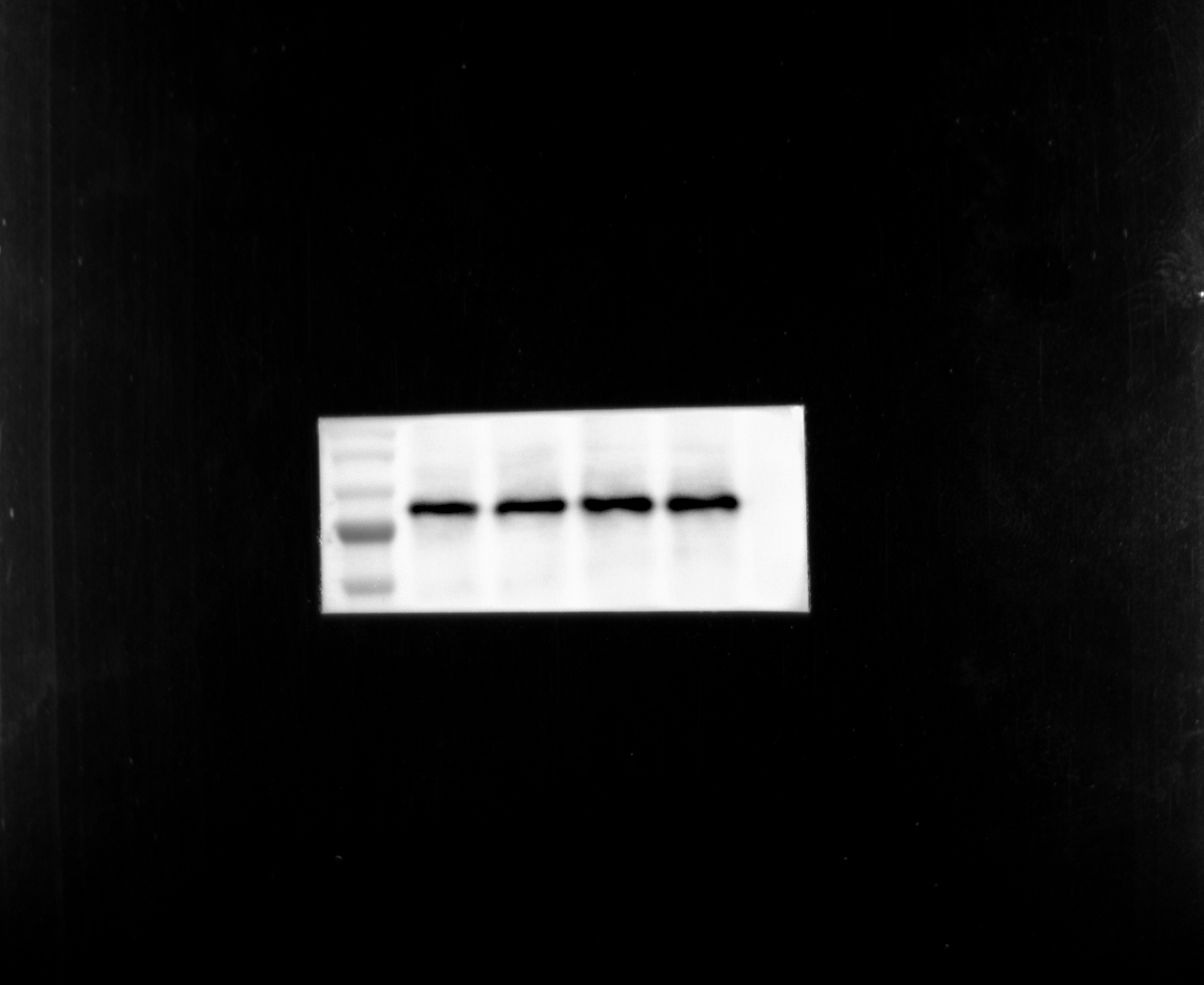

Supplement: Supplementary file 1 [file cimb-47-00936-s001.zip › cimb-3956315-supplementary/APOC2_ccRCC_RawWB_FullMembranes/cropped display images/10/Fig 3D stat3/5.Tif]

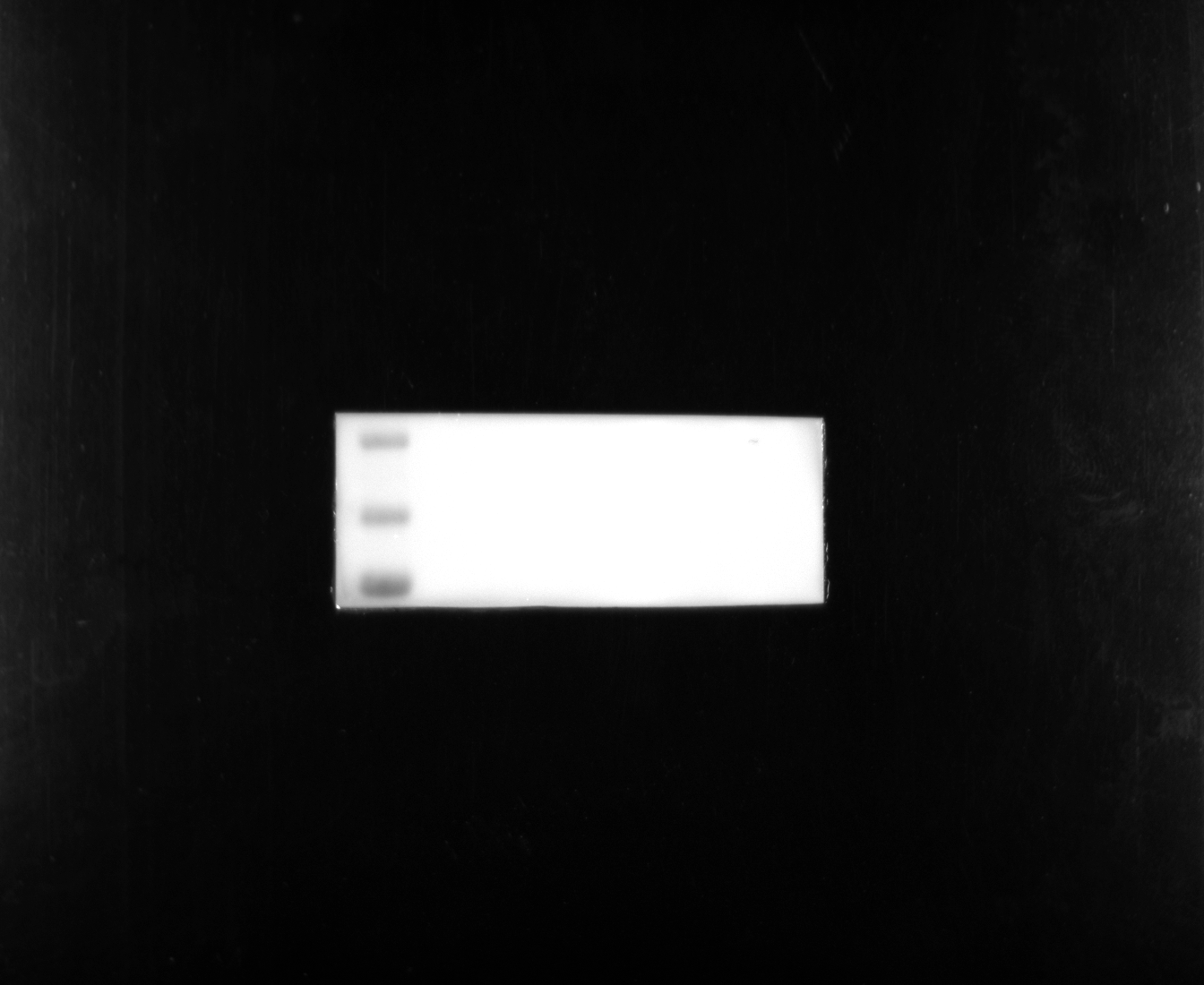

Supplement: Supplementary file 1 [file cimb-47-00936-s001.zip › cimb-3956315-supplementary/APOC2_ccRCC_RawWB_FullMembranes/cropped display images/10/Fig 3D β- actin/0.Tif]

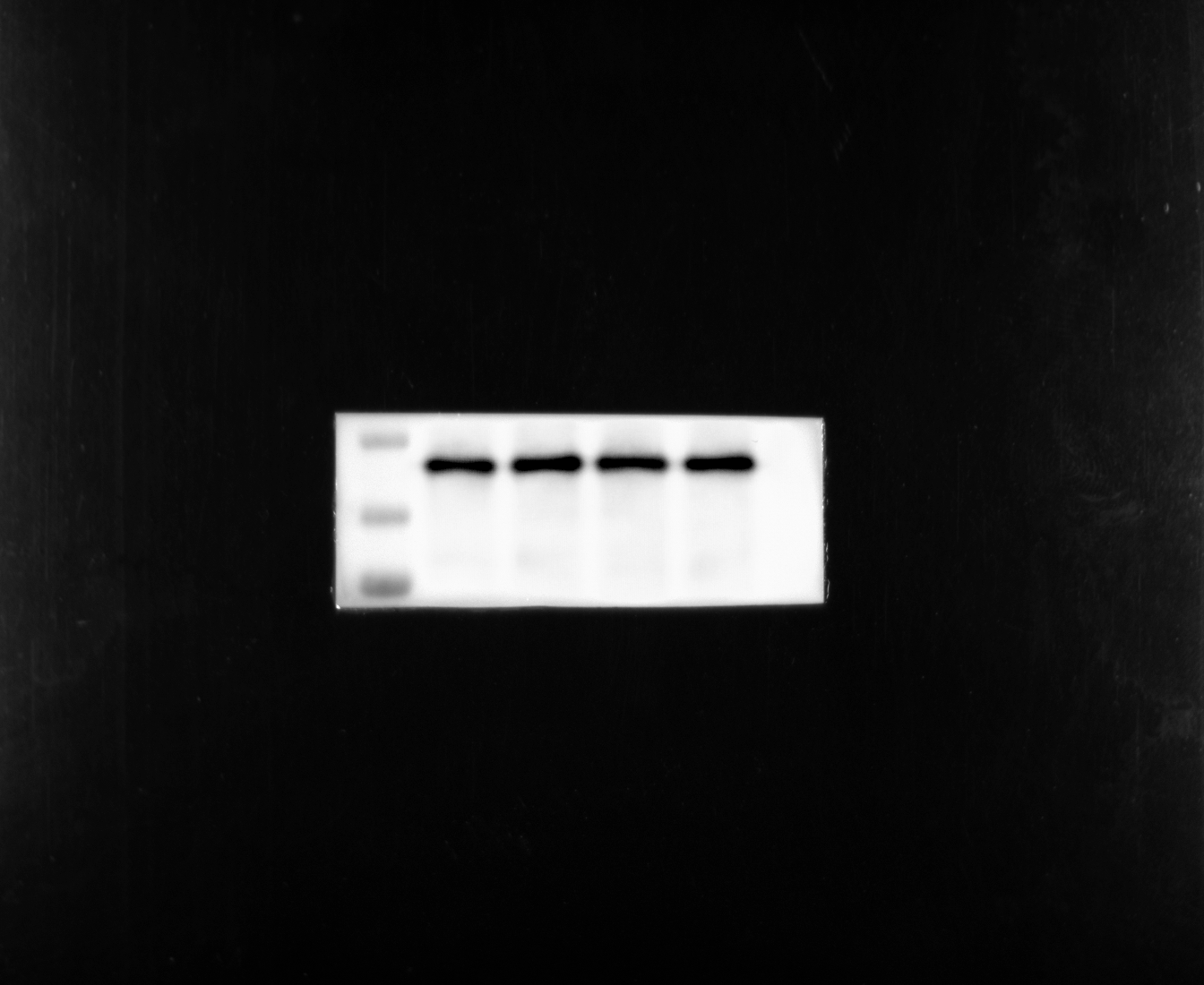

Supplement: Supplementary file 1 [file cimb-47-00936-s001.zip › cimb-3956315-supplementary/APOC2_ccRCC_RawWB_FullMembranes/cropped display images/10/Fig 3D β- actin/1.Tif]

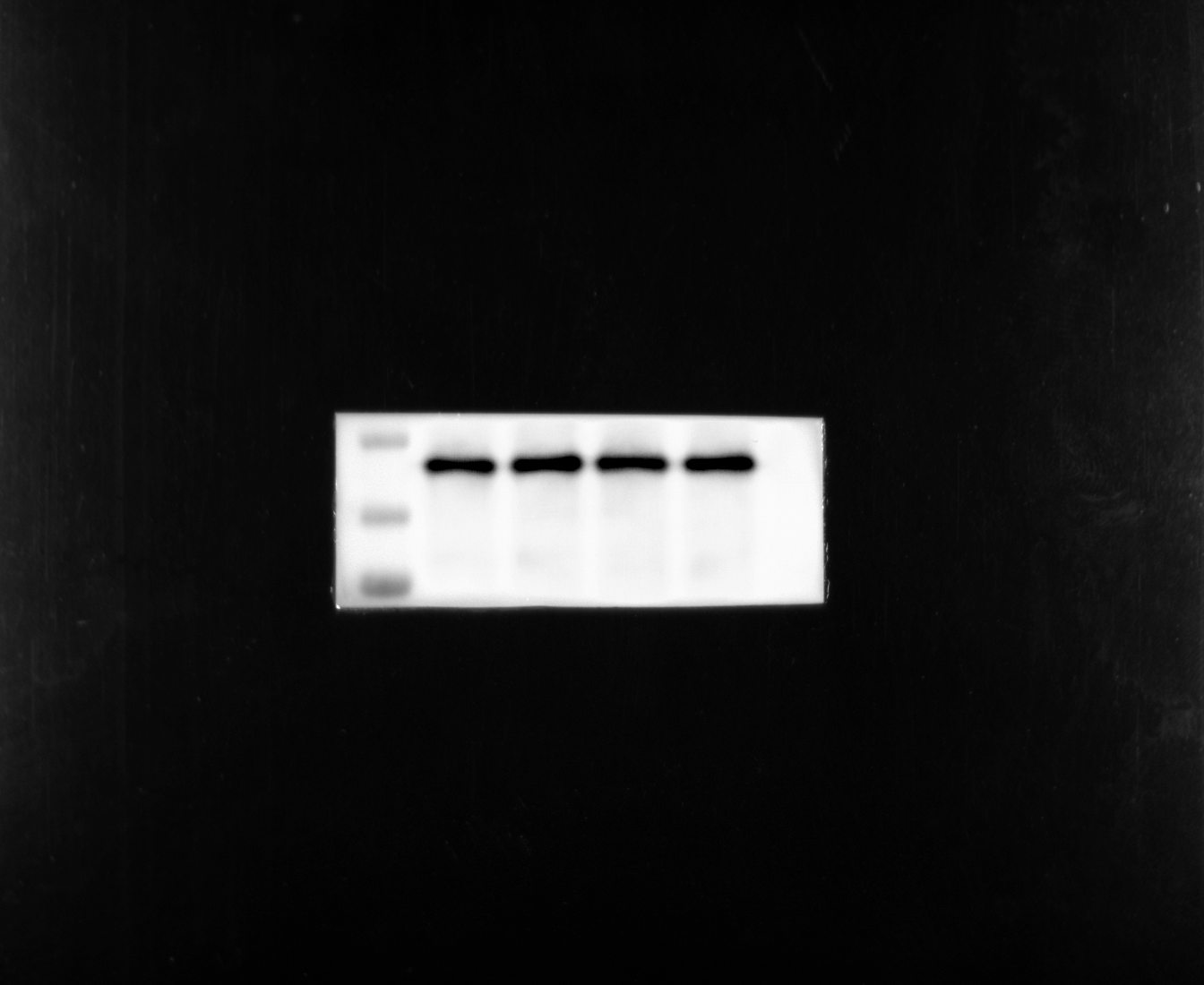

Supplement: Supplementary file 1 [file cimb-47-00936-s001.zip › cimb-3956315-supplementary/APOC2_ccRCC_RawWB_FullMembranes/cropped display images/10/Fig 3D β- actin/2.Tif]

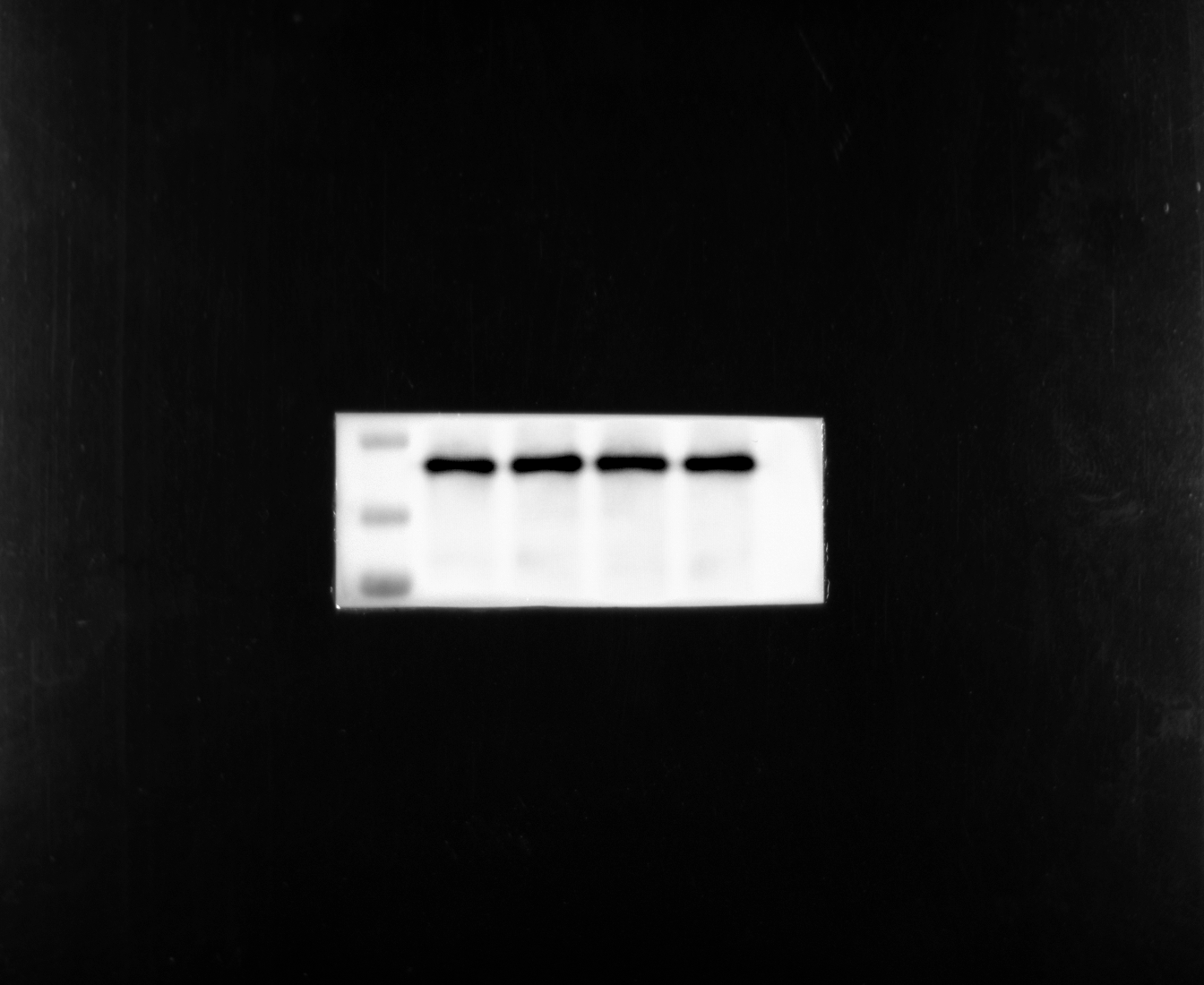

Supplement: Supplementary file 1 [file cimb-47-00936-s001.zip › cimb-3956315-supplementary/APOC2_ccRCC_RawWB_FullMembranes/cropped display images/10/Fig 3D β- actin/3.Tif]

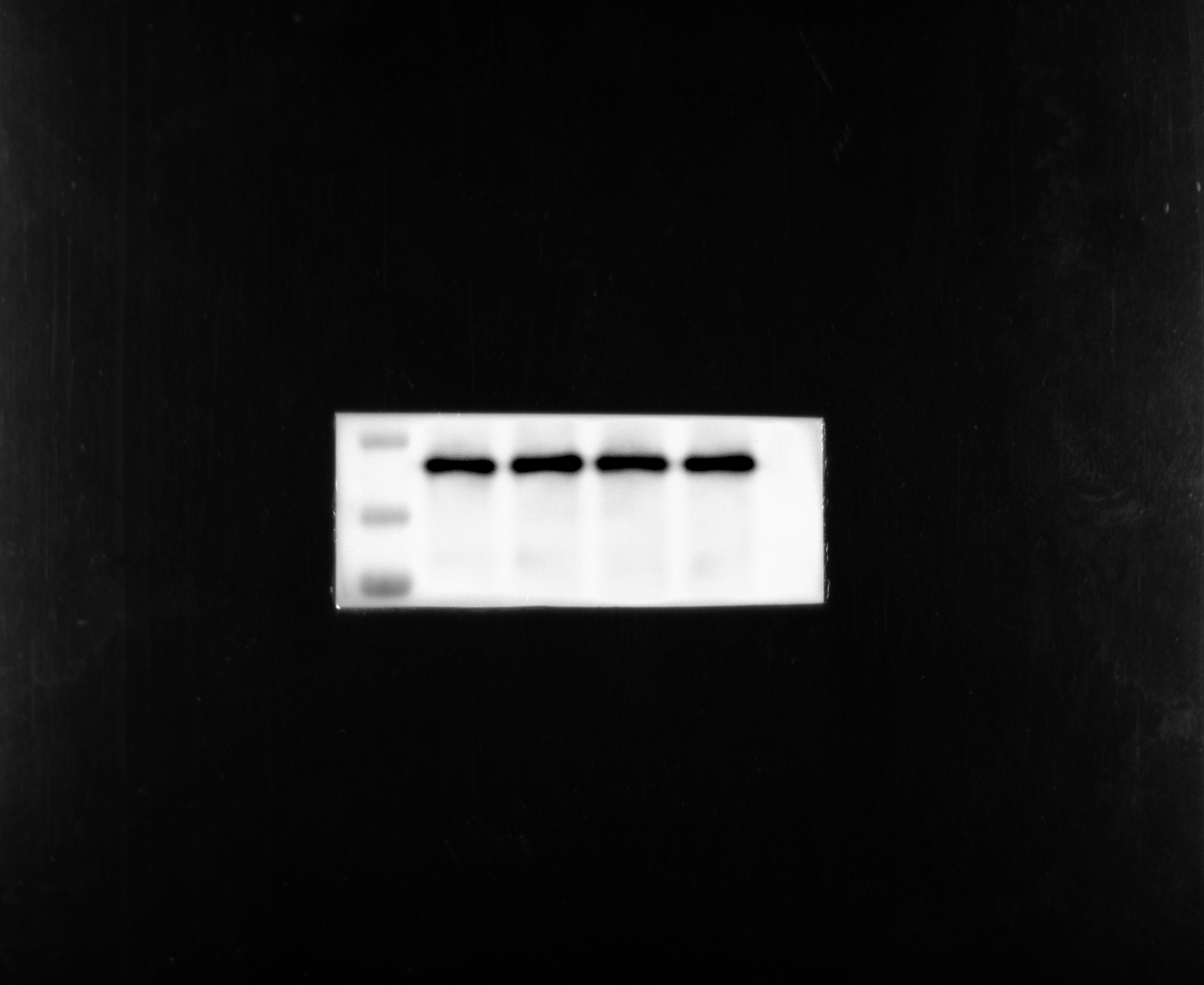

Supplement: Supplementary file 1 [file cimb-47-00936-s001.zip › cimb-3956315-supplementary/APOC2_ccRCC_RawWB_FullMembranes/cropped display images/10/Fig 3D β- actin/4.Tif]

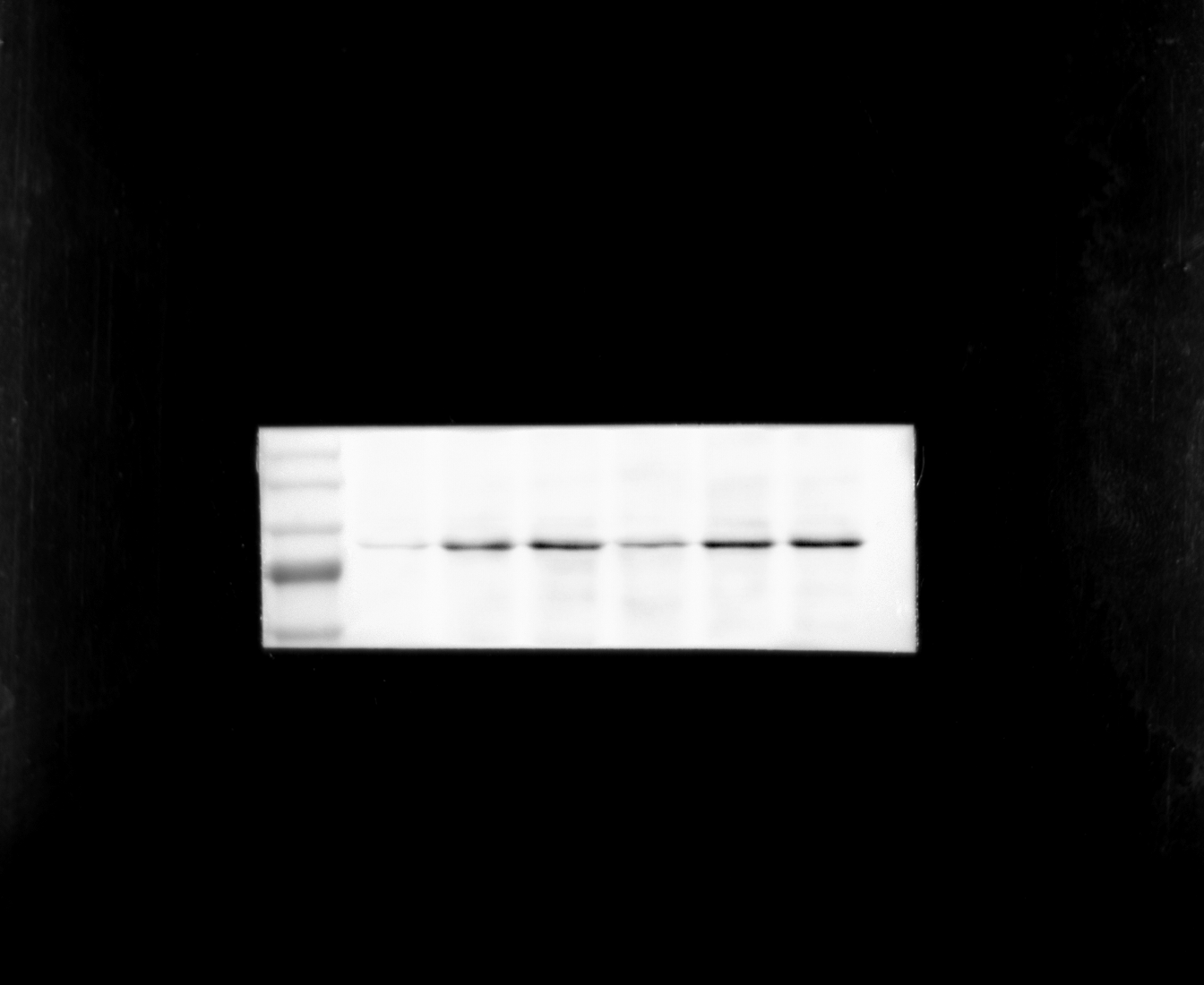

Supplement: Supplementary file 1 [file cimb-47-00936-s001.zip › cimb-3956315-supplementary/APOC2_ccRCC_RawWB_FullMembranes/cropped display images/11/Fig 3K p-stat3/0.5.Tif]

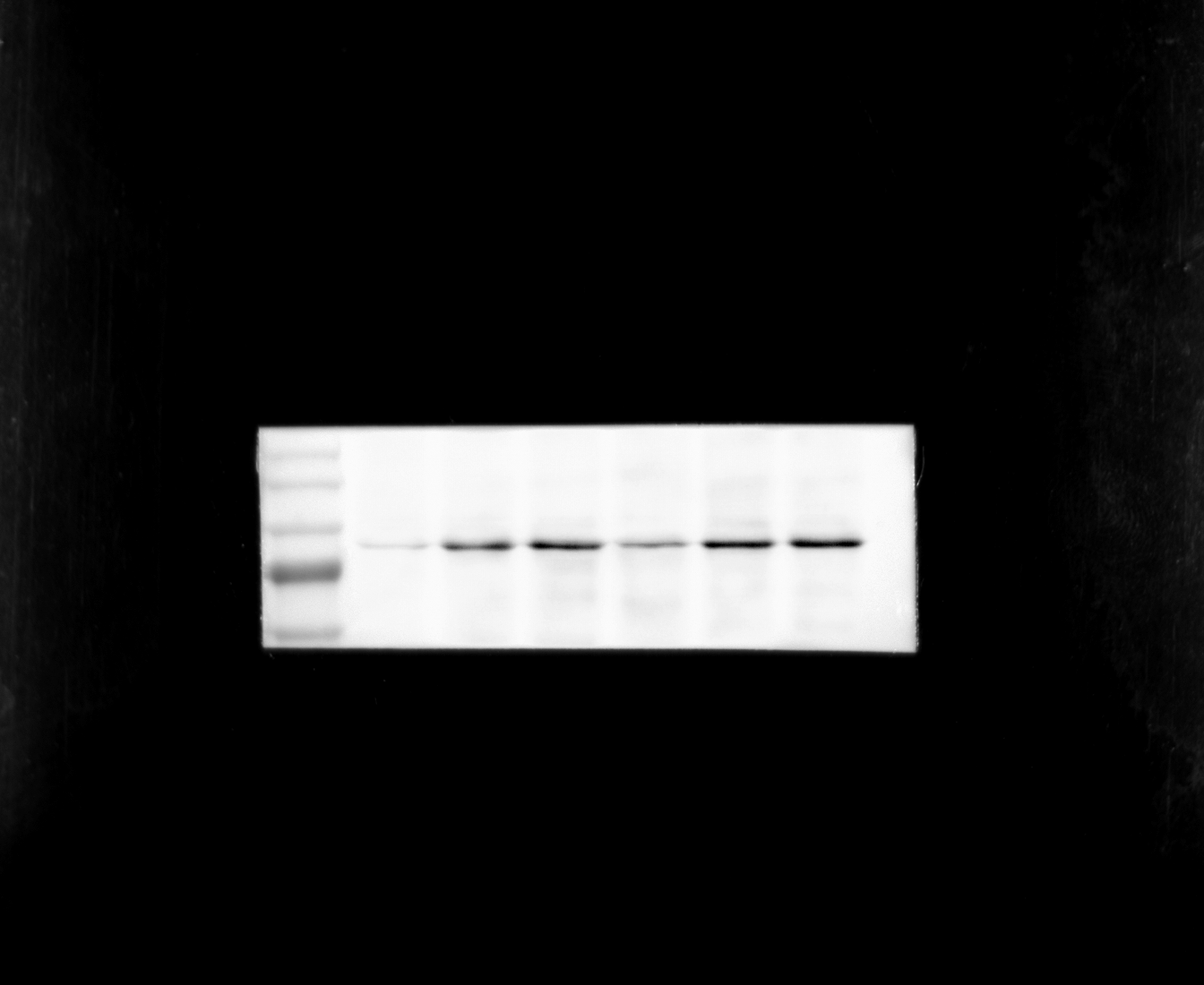

Supplement: Supplementary file 1 [file cimb-47-00936-s001.zip › cimb-3956315-supplementary/APOC2_ccRCC_RawWB_FullMembranes/cropped display images/11/Fig 3K p-stat3/1.Tif]

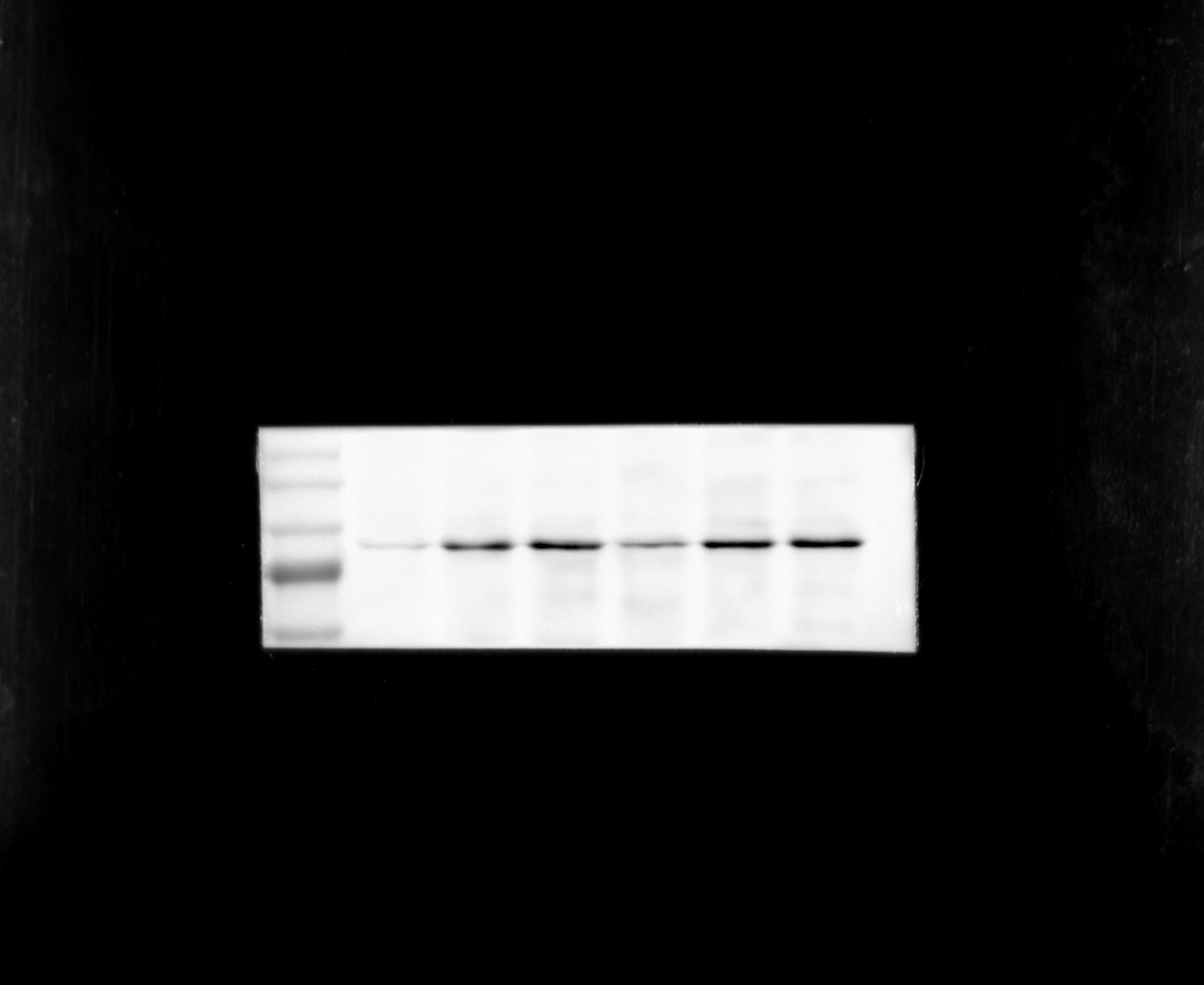

Supplement: Supplementary file 1 [file cimb-47-00936-s001.zip › cimb-3956315-supplementary/APOC2_ccRCC_RawWB_FullMembranes/cropped display images/11/Fig 3K p-stat3/2.Tif]

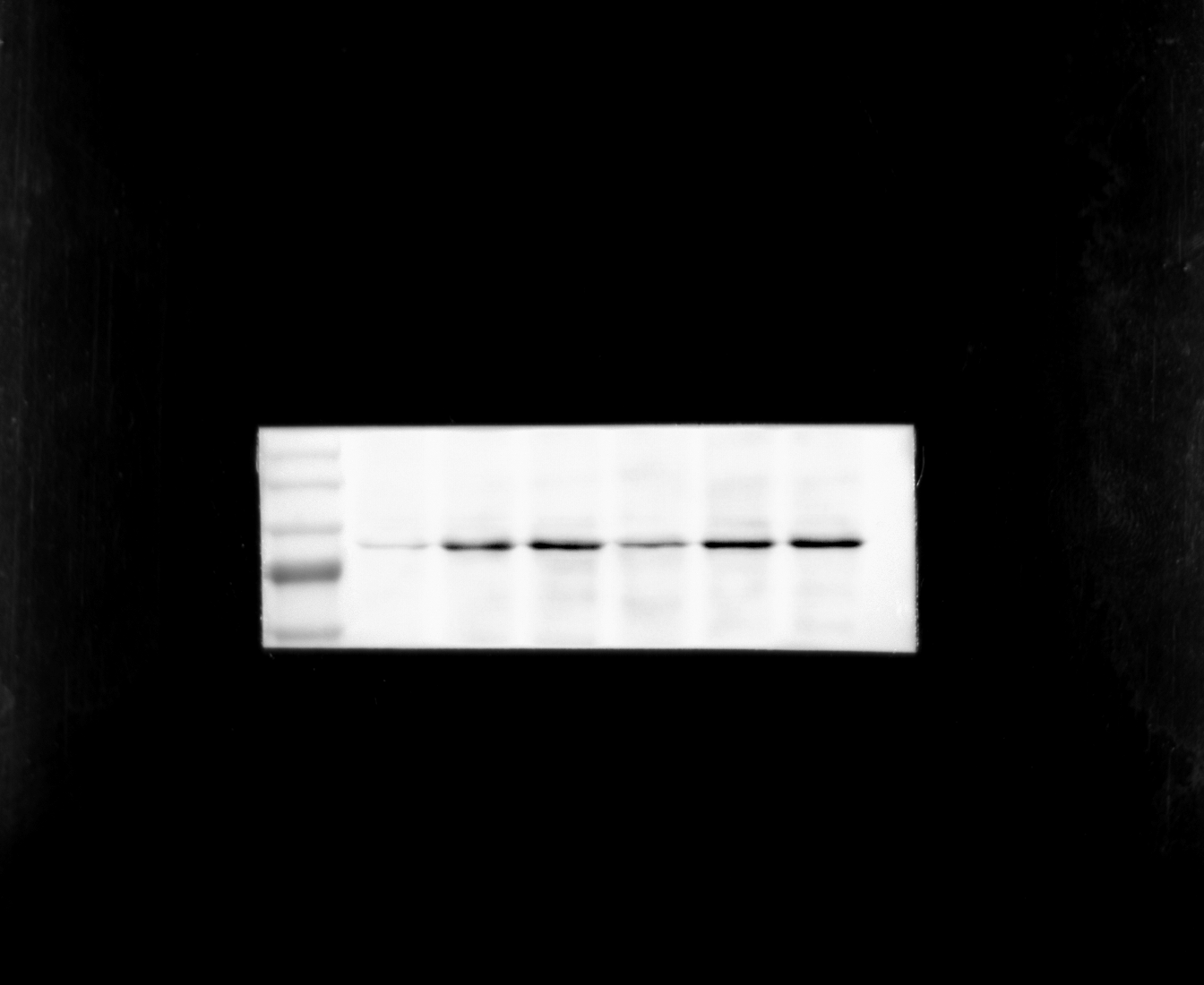

Supplement: Supplementary file 1 [file cimb-47-00936-s001.zip › cimb-3956315-supplementary/APOC2_ccRCC_RawWB_FullMembranes/cropped display images/11/Fig 3K p-stat3/3.Tif]

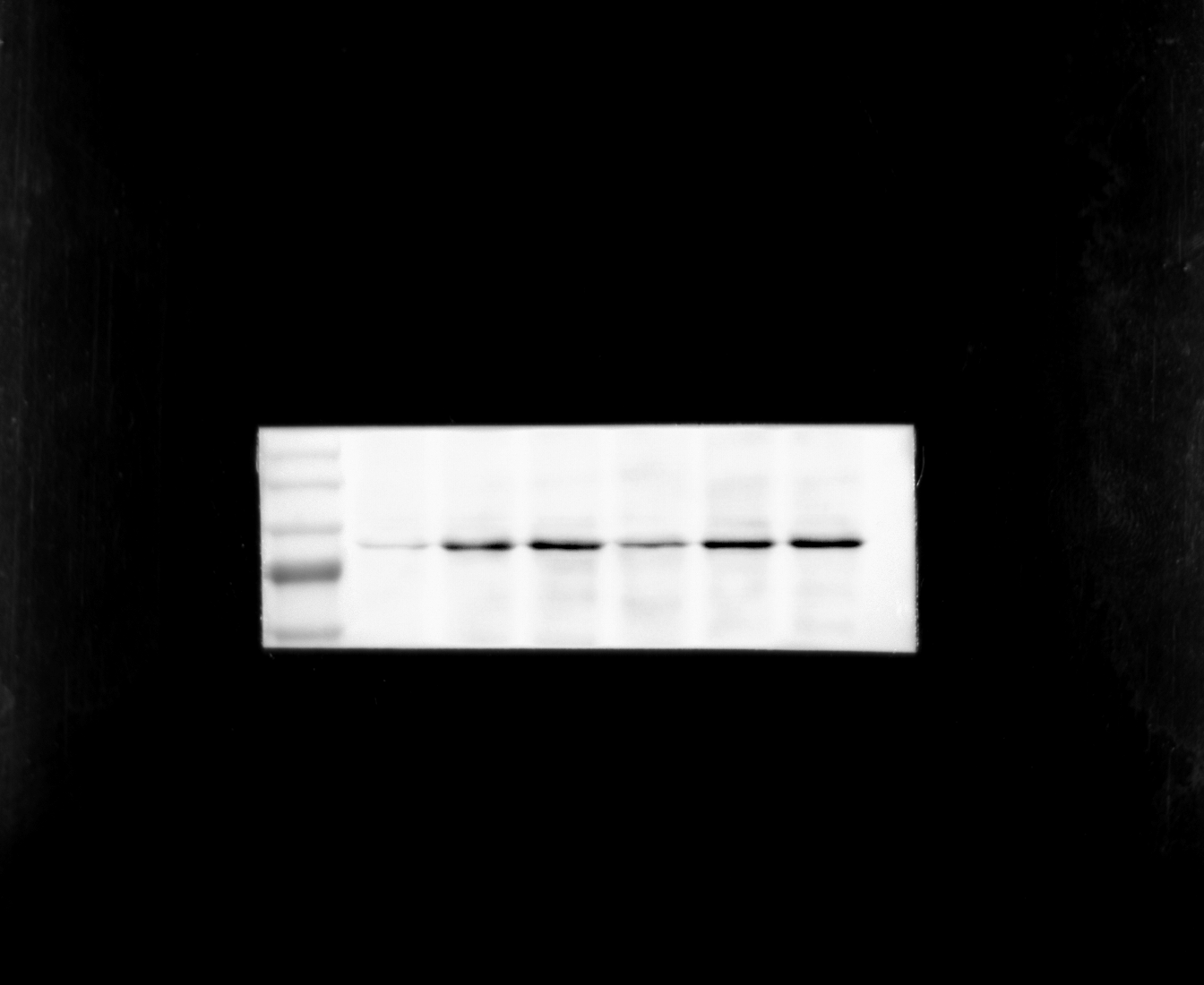

Supplement: Supplementary file 1 [file cimb-47-00936-s001.zip › cimb-3956315-supplementary/APOC2_ccRCC_RawWB_FullMembranes/cropped display images/11/Fig 3K p-stat3/4.Tif]

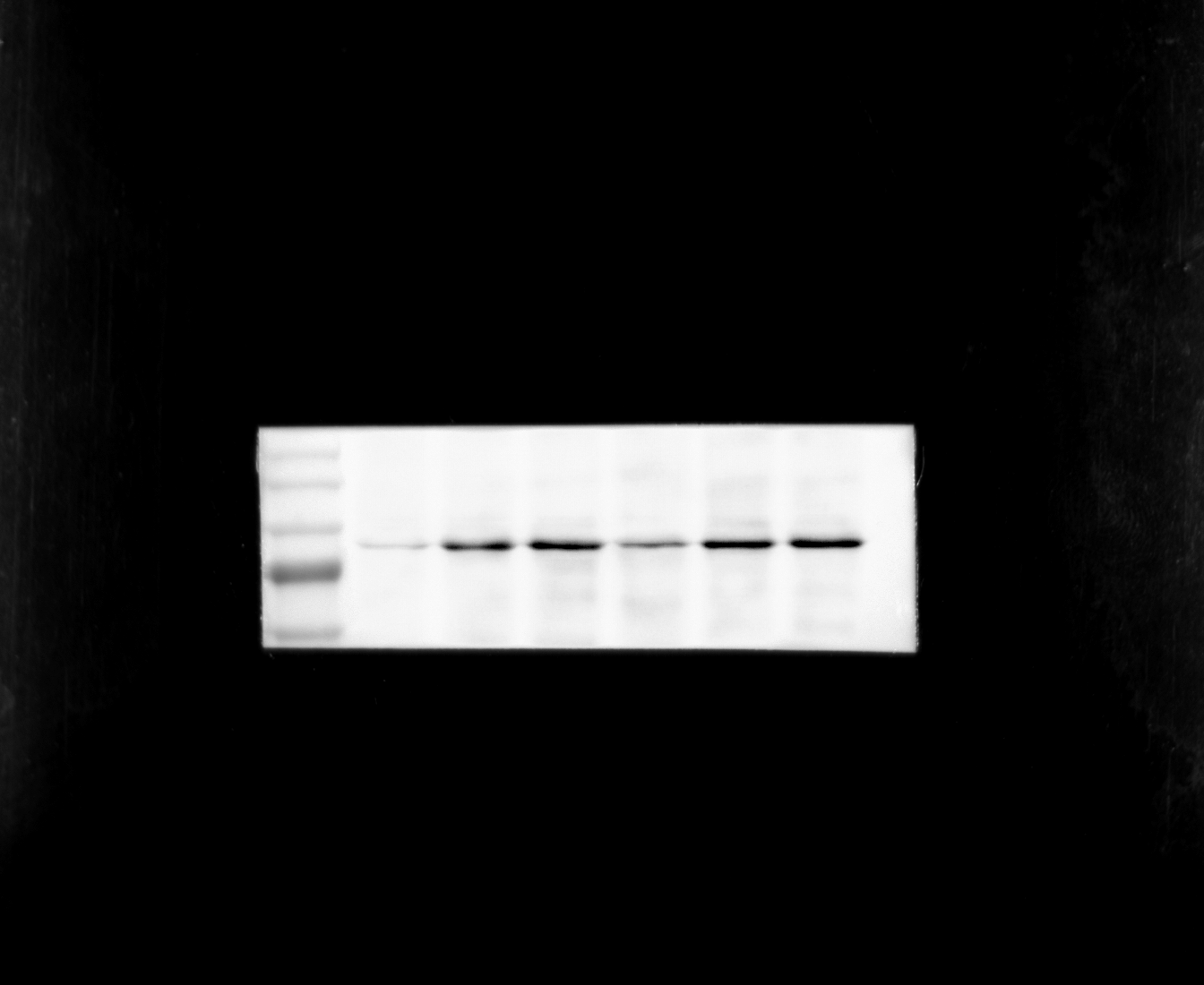

Supplement: Supplementary file 1 [file cimb-47-00936-s001.zip › cimb-3956315-supplementary/APOC2_ccRCC_RawWB_FullMembranes/cropped display images/11/Fig 3K p-stat3/5.Tif]

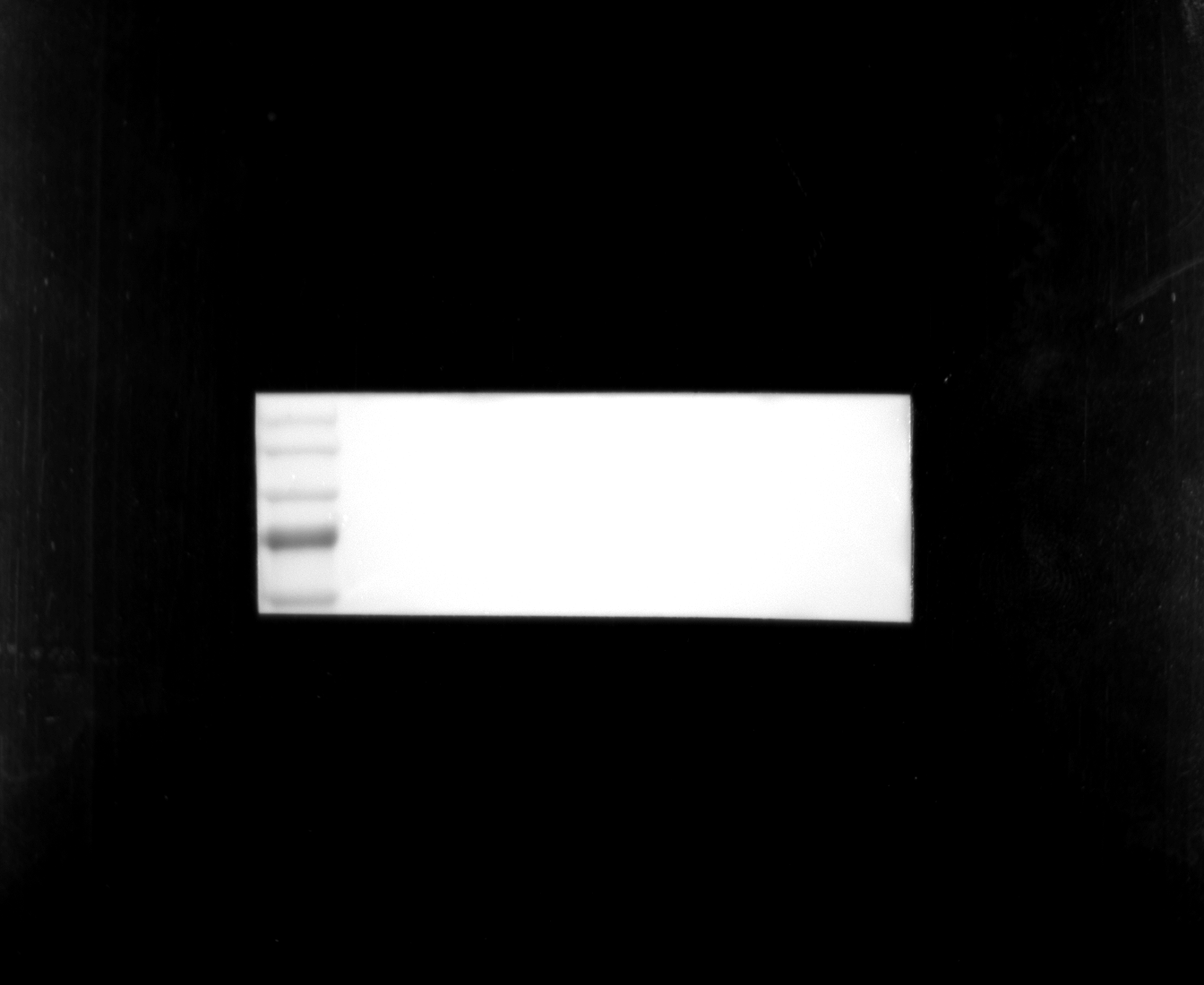

Supplement: Supplementary file 1 [file cimb-47-00936-s001.zip › cimb-3956315-supplementary/APOC2_ccRCC_RawWB_FullMembranes/cropped display images/11/Fig 3K stat3/0.Tif]

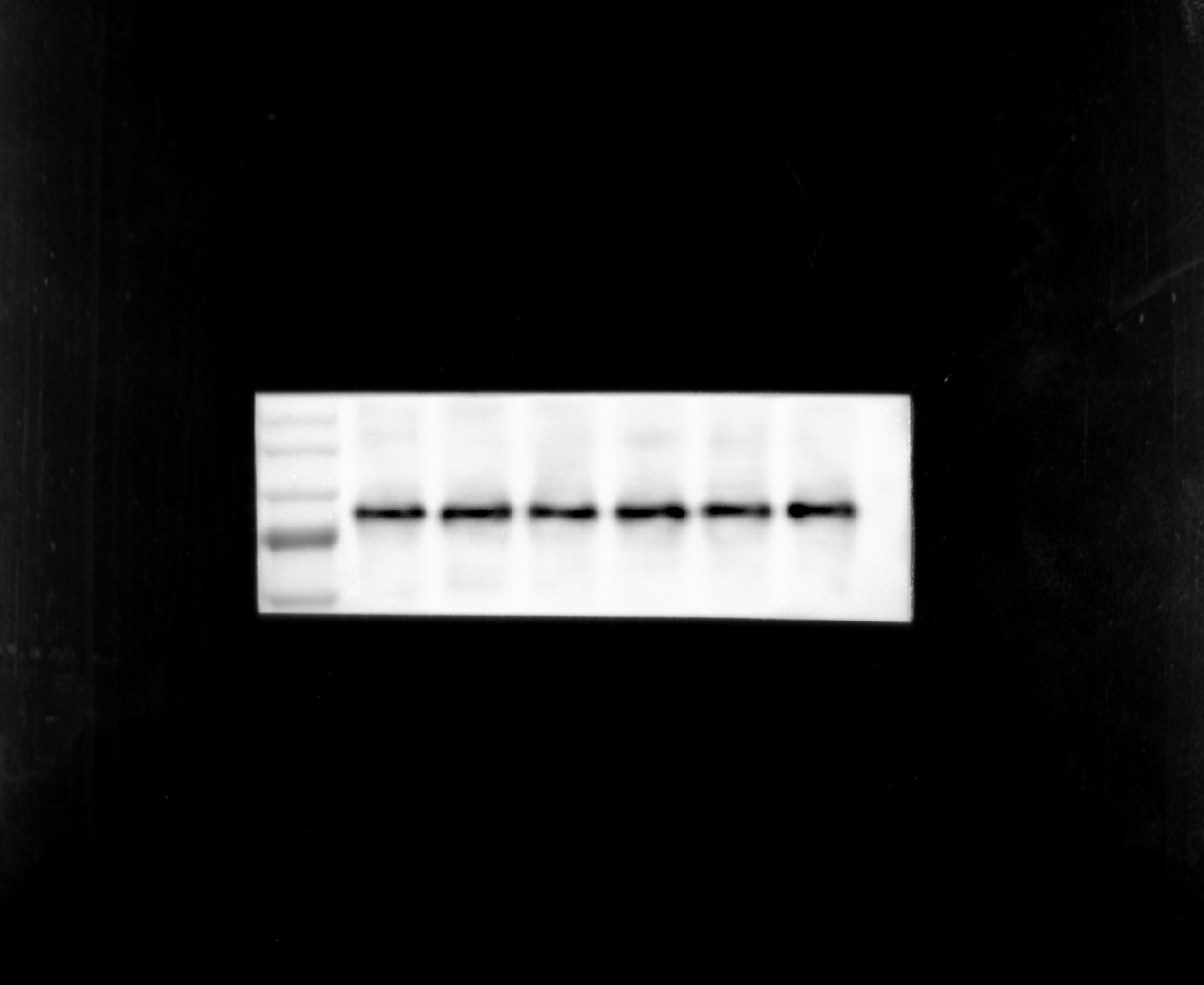

Supplement: Supplementary file 1 [file cimb-47-00936-s001.zip › cimb-3956315-supplementary/APOC2_ccRCC_RawWB_FullMembranes/cropped display images/11/Fig 3K stat3/1.Tif]

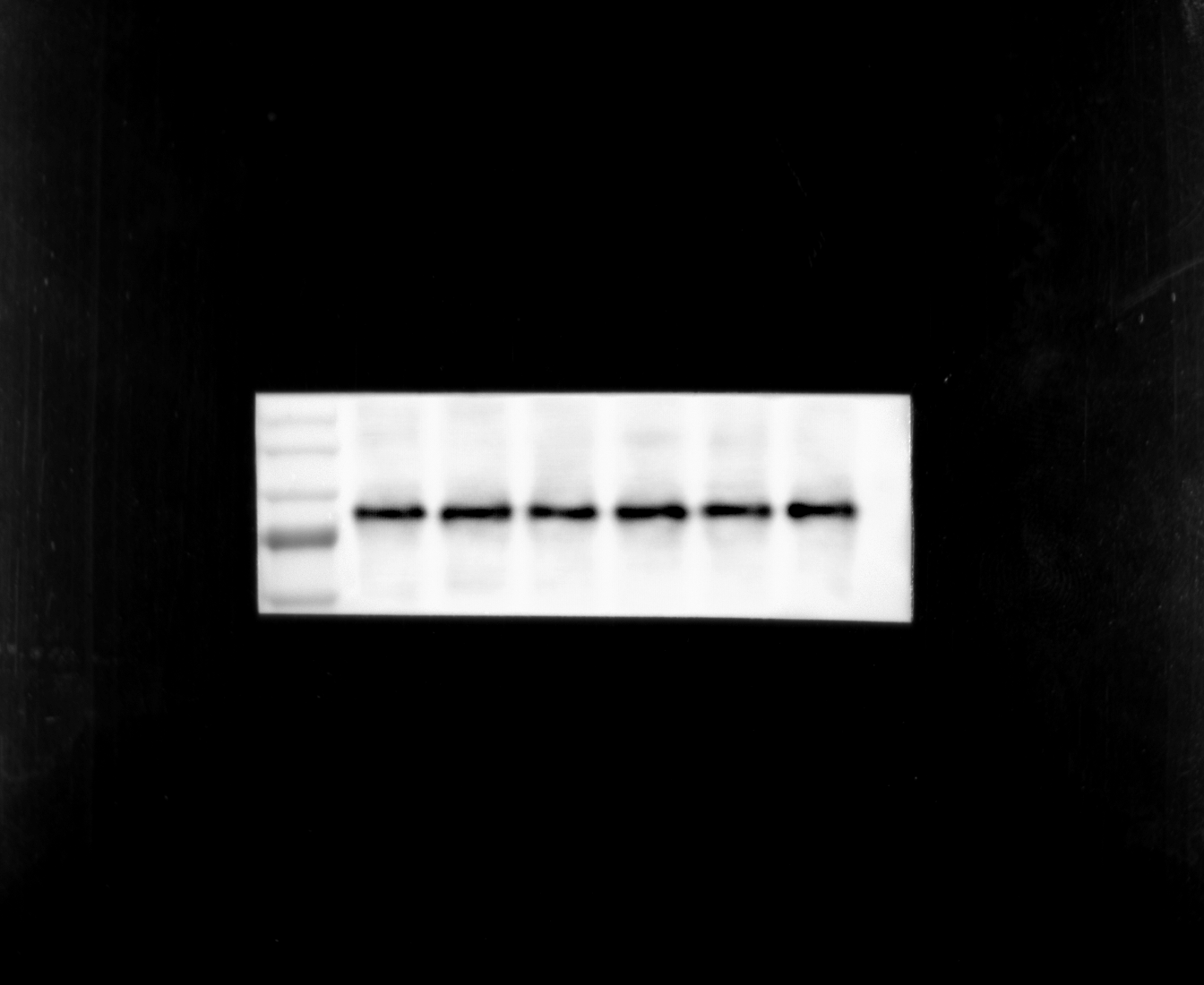

Supplement: Supplementary file 1 [file cimb-47-00936-s001.zip › cimb-3956315-supplementary/APOC2_ccRCC_RawWB_FullMembranes/cropped display images/11/Fig 3K stat3/2.Tif]

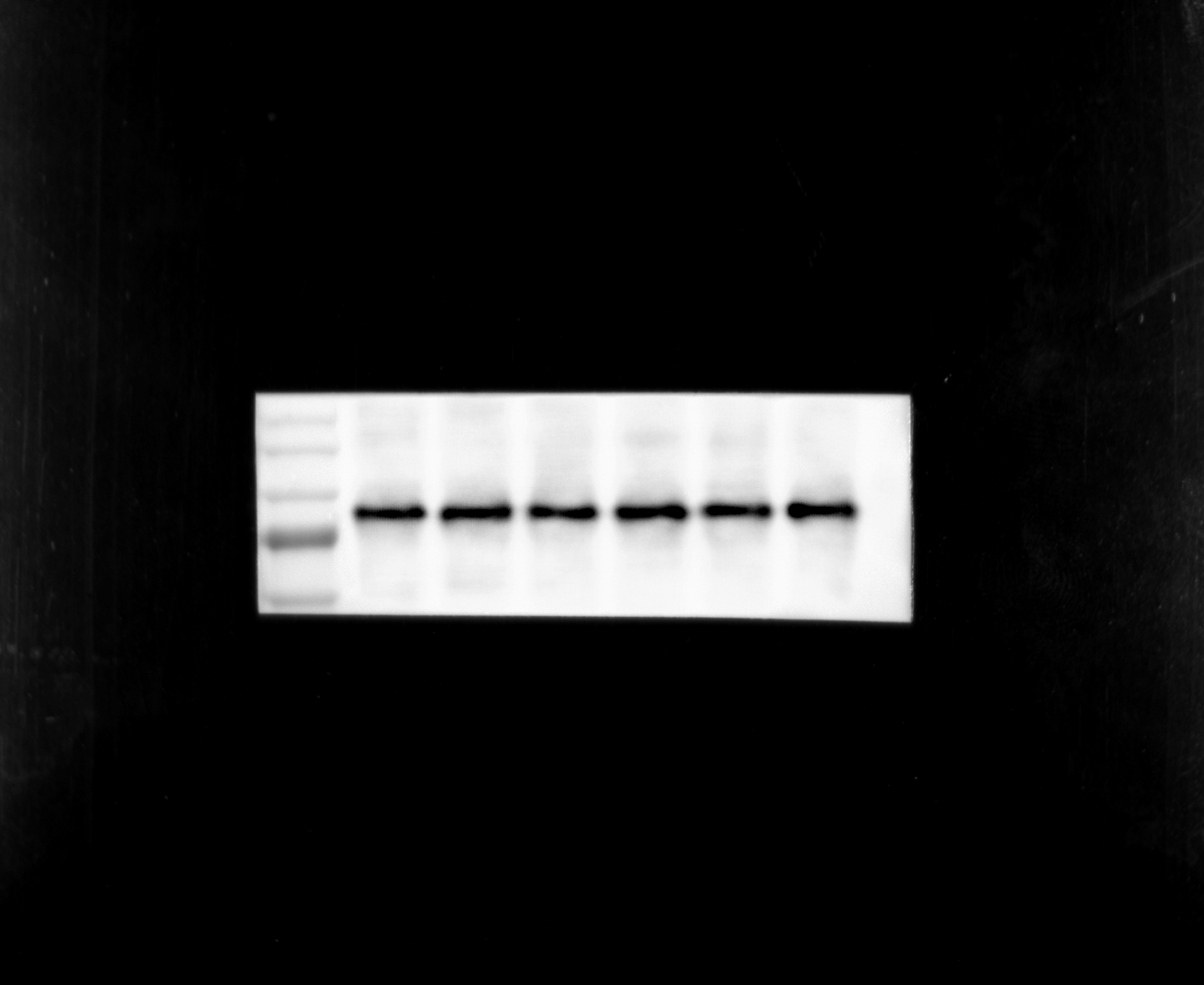

Supplement: Supplementary file 1 [file cimb-47-00936-s001.zip › cimb-3956315-supplementary/APOC2_ccRCC_RawWB_FullMembranes/cropped display images/11/Fig 3K stat3/3.Tif]

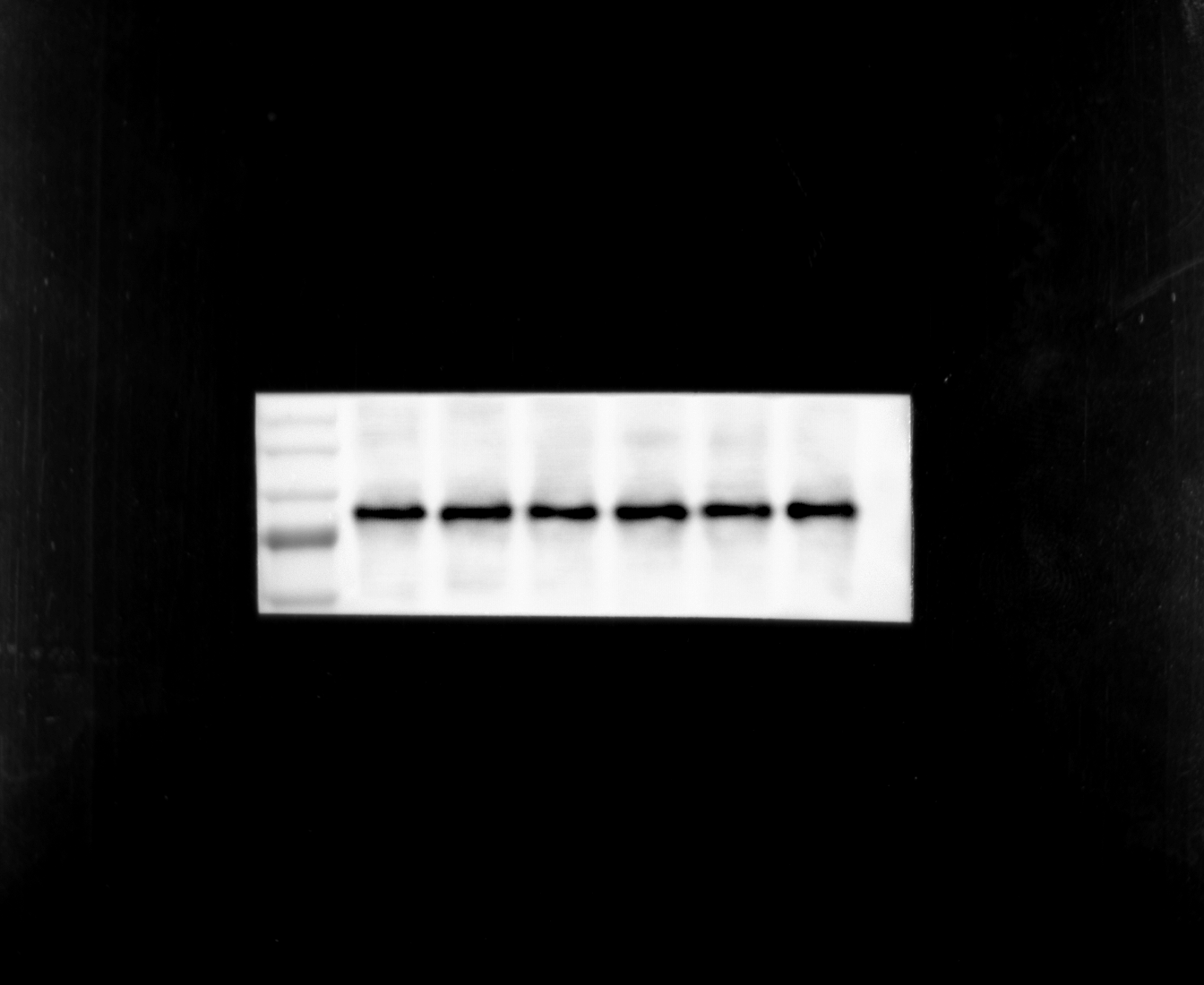

Supplement: Supplementary file 1 [file cimb-47-00936-s001.zip › cimb-3956315-supplementary/APOC2_ccRCC_RawWB_FullMembranes/cropped display images/11/Fig 3K stat3/4.Tif]

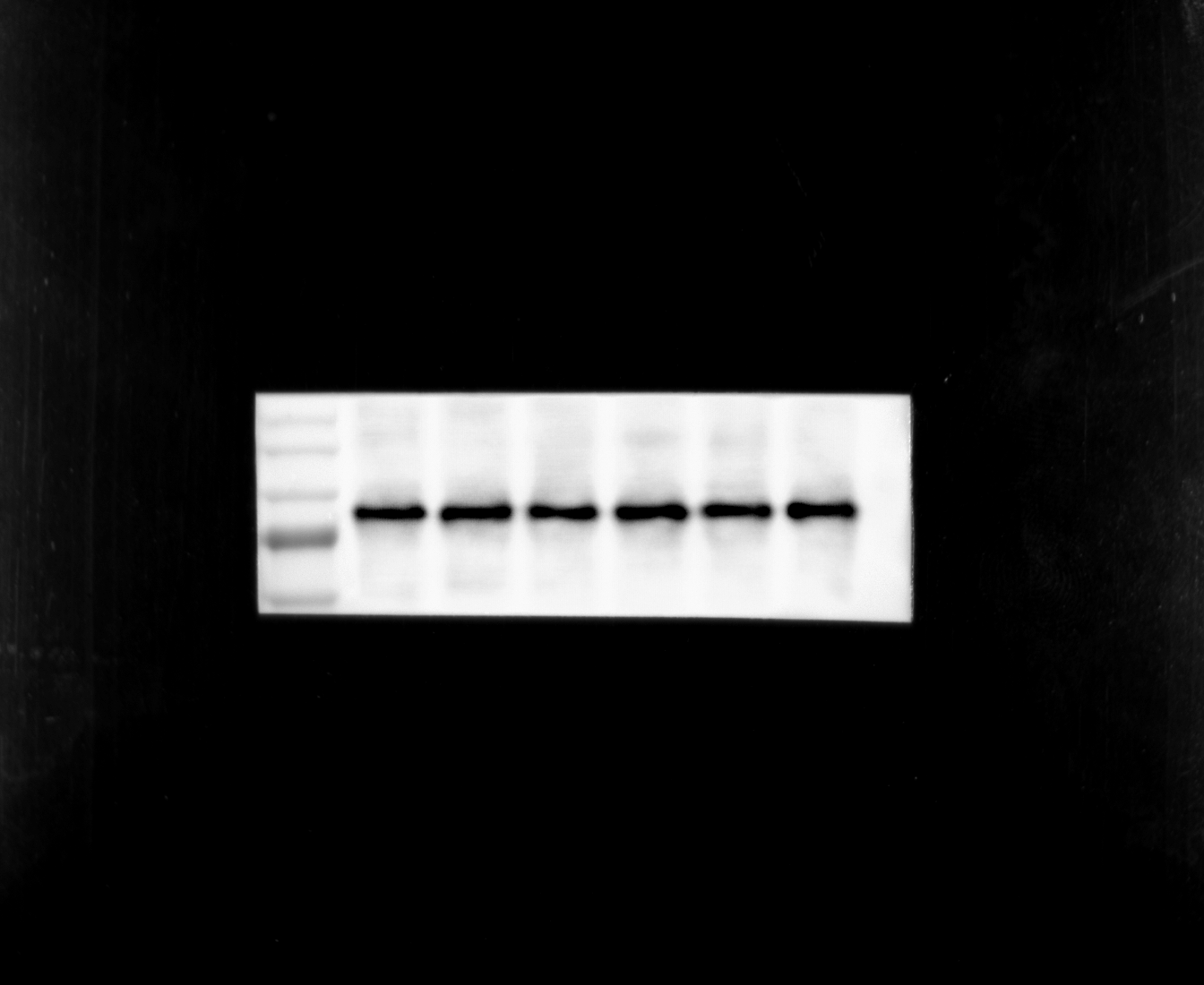

Supplement: Supplementary file 1 [file cimb-47-00936-s001.zip › cimb-3956315-supplementary/APOC2_ccRCC_RawWB_FullMembranes/cropped display images/11/Fig 3K stat3/5.Tif]

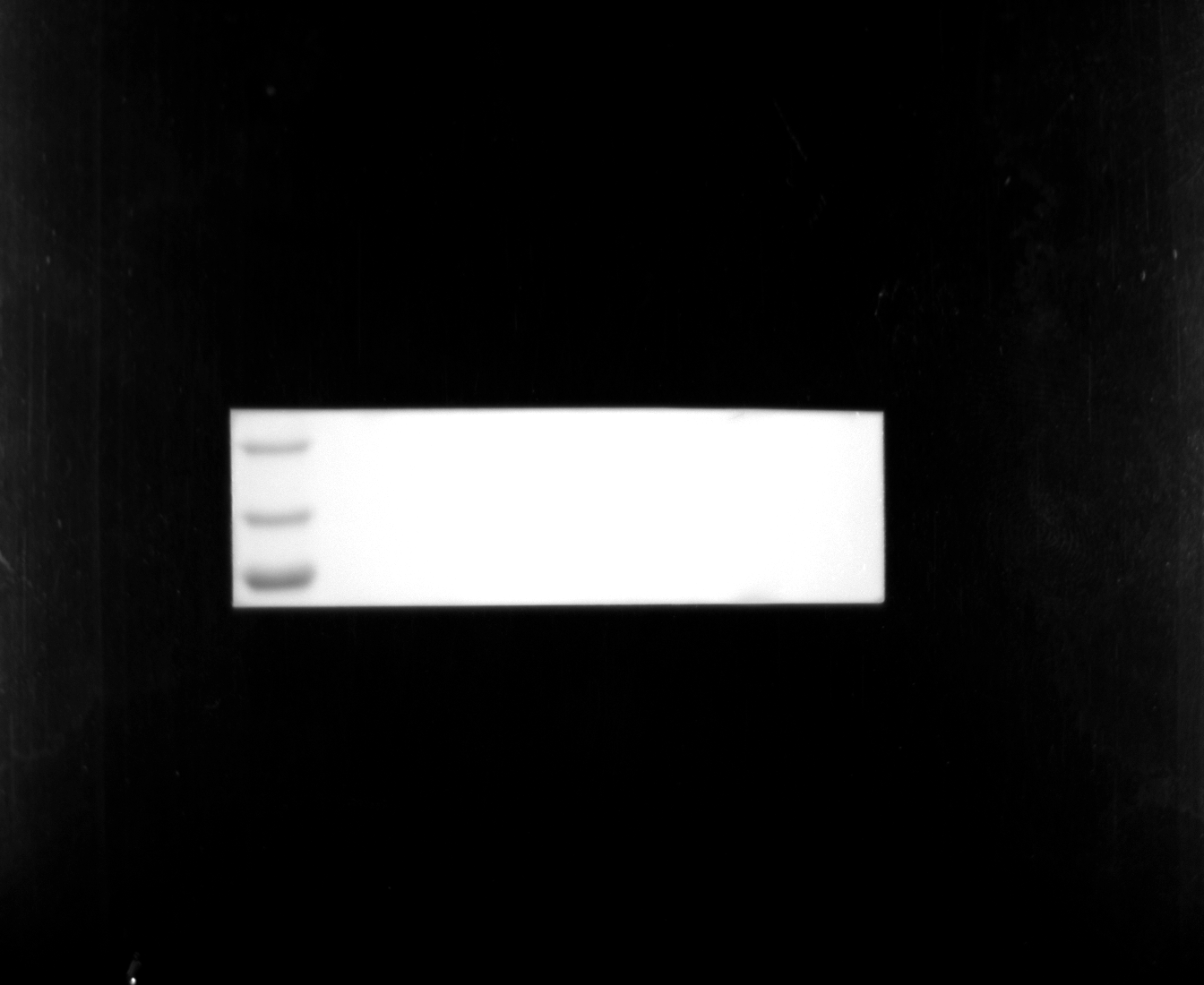

Supplement: Supplementary file 1 [file cimb-47-00936-s001.zip › cimb-3956315-supplementary/APOC2_ccRCC_RawWB_FullMembranes/cropped display images/11/β- actin/0.Tif]

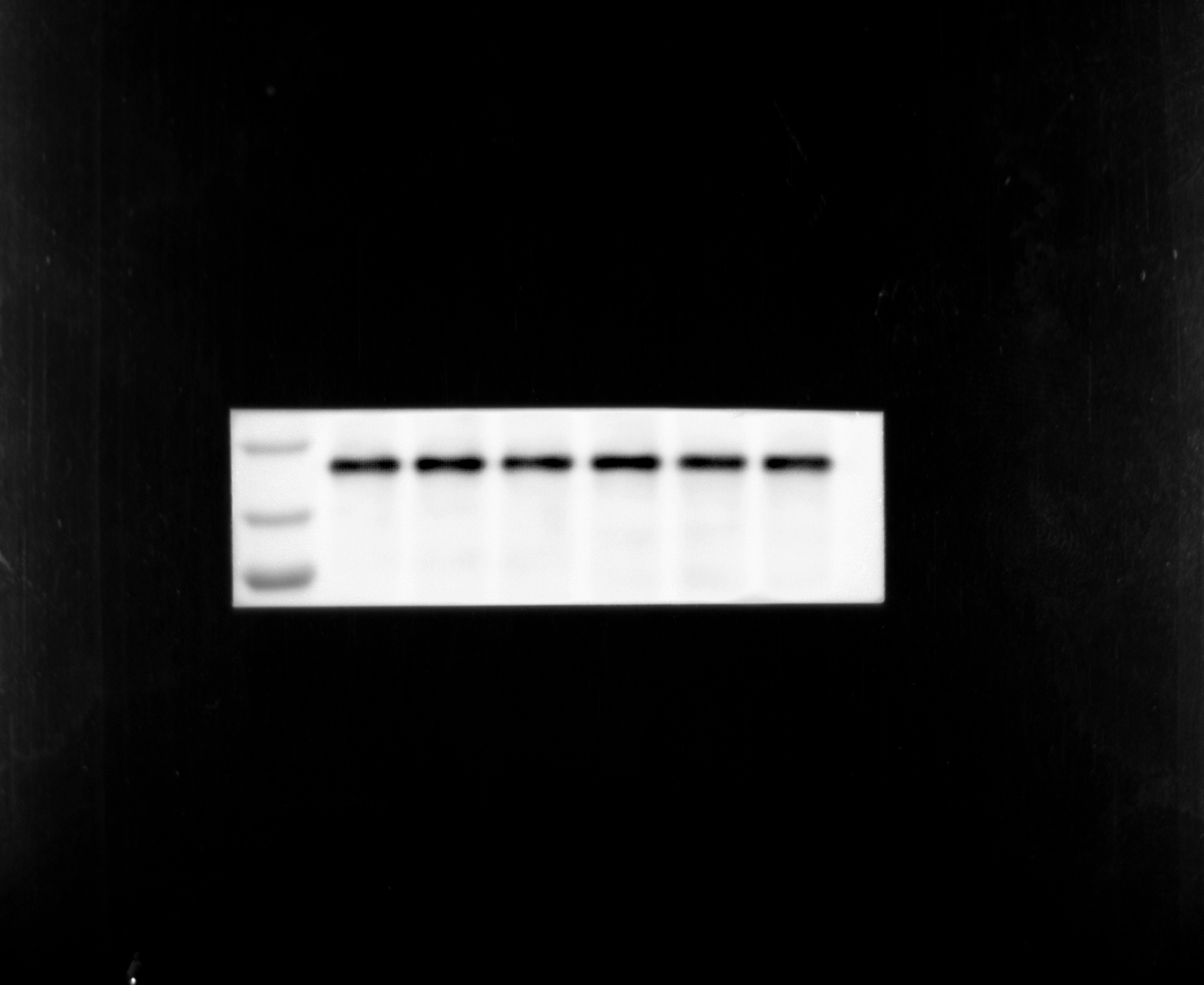

Supplement: Supplementary file 1 [file cimb-47-00936-s001.zip › cimb-3956315-supplementary/APOC2_ccRCC_RawWB_FullMembranes/cropped display images/11/β- actin/1.Tif]

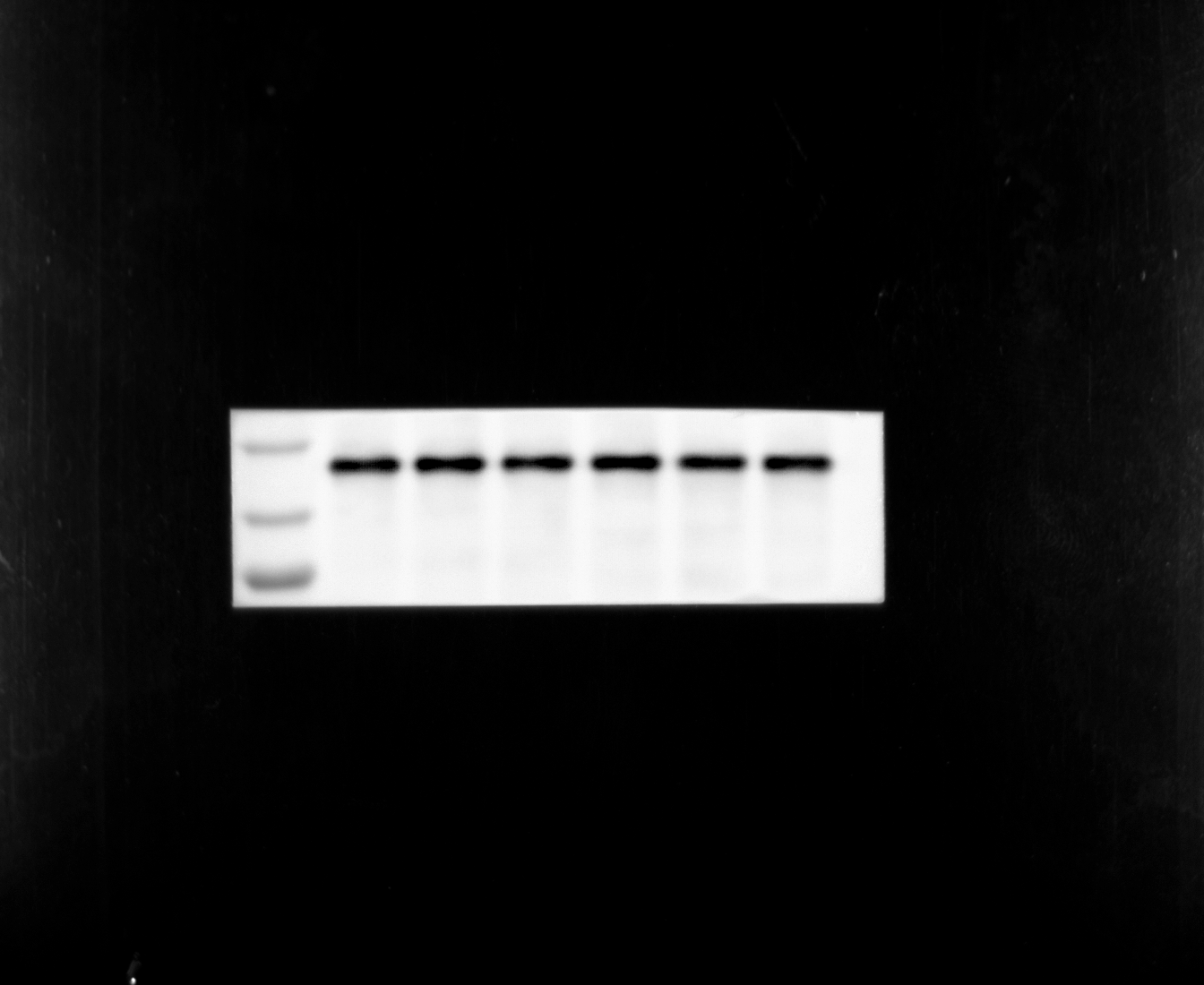

Supplement: Supplementary file 1 [file cimb-47-00936-s001.zip › cimb-3956315-supplementary/APOC2_ccRCC_RawWB_FullMembranes/cropped display images/11/β- actin/2.Tif]

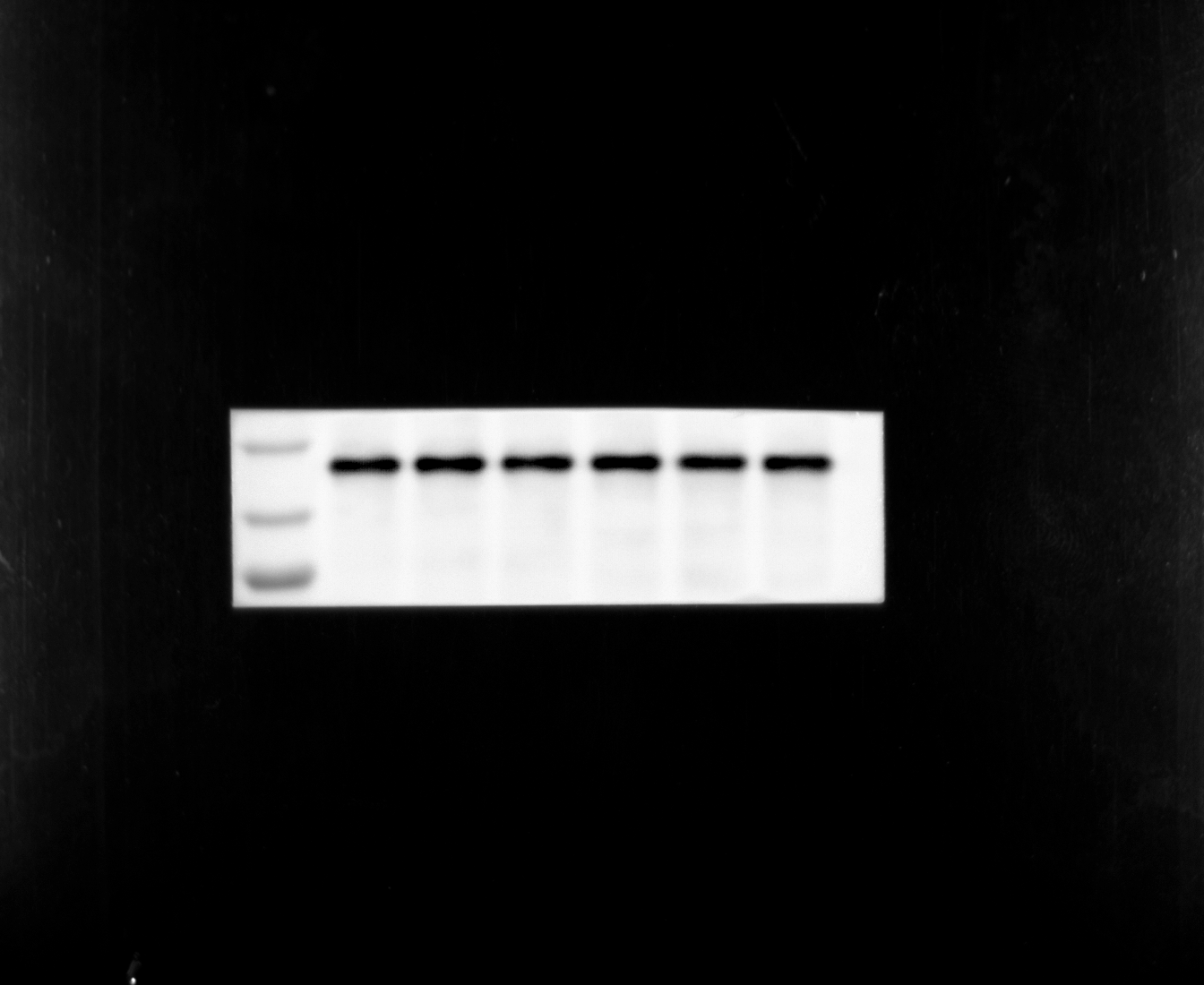

Supplement: Supplementary file 1 [file cimb-47-00936-s001.zip › cimb-3956315-supplementary/APOC2_ccRCC_RawWB_FullMembranes/cropped display images/11/β- actin/3.Tif]

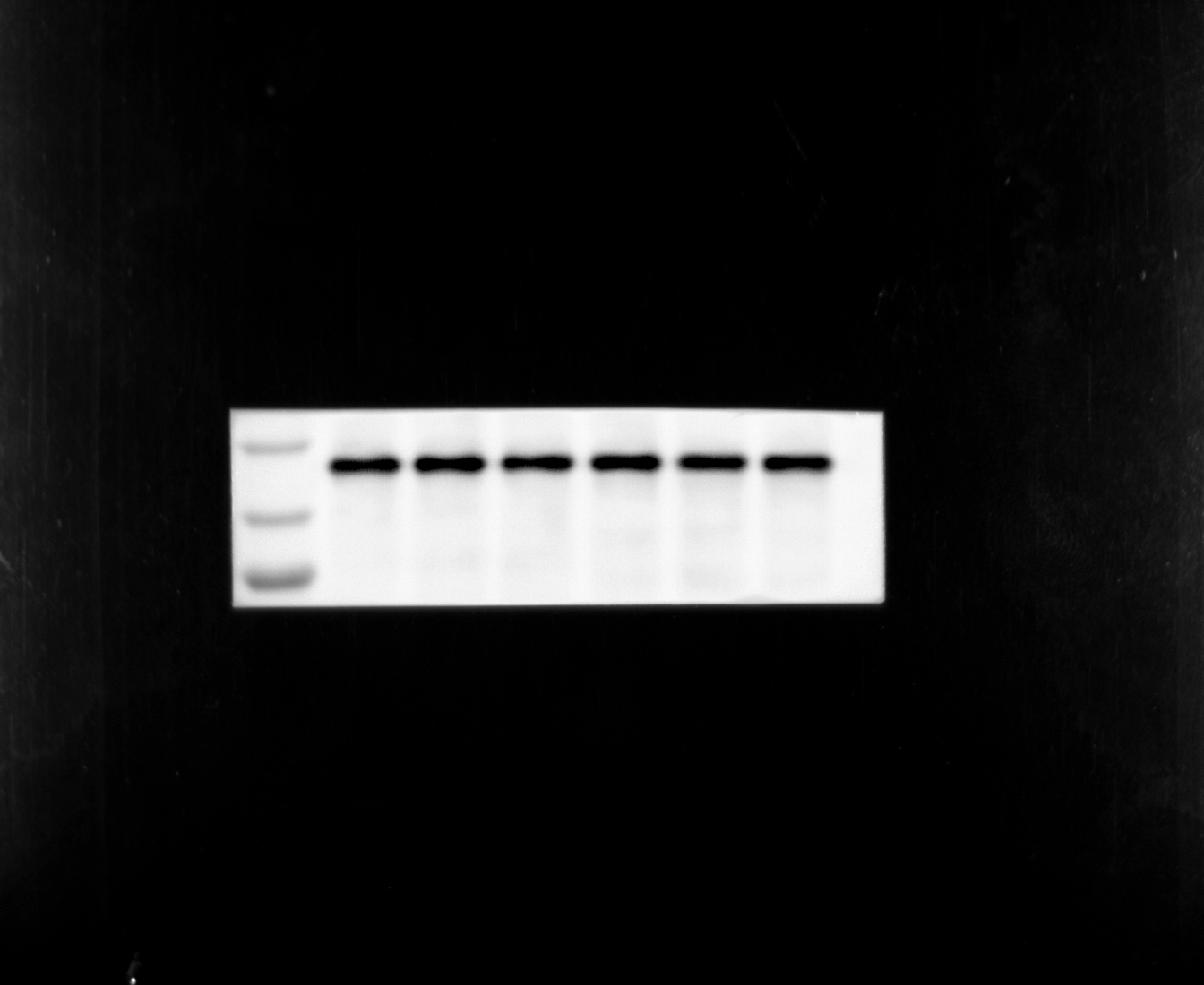

Supplement: Supplementary file 1 [file cimb-47-00936-s001.zip › cimb-3956315-supplementary/APOC2_ccRCC_RawWB_FullMembranes/cropped display images/11/β- actin/4.Tif]

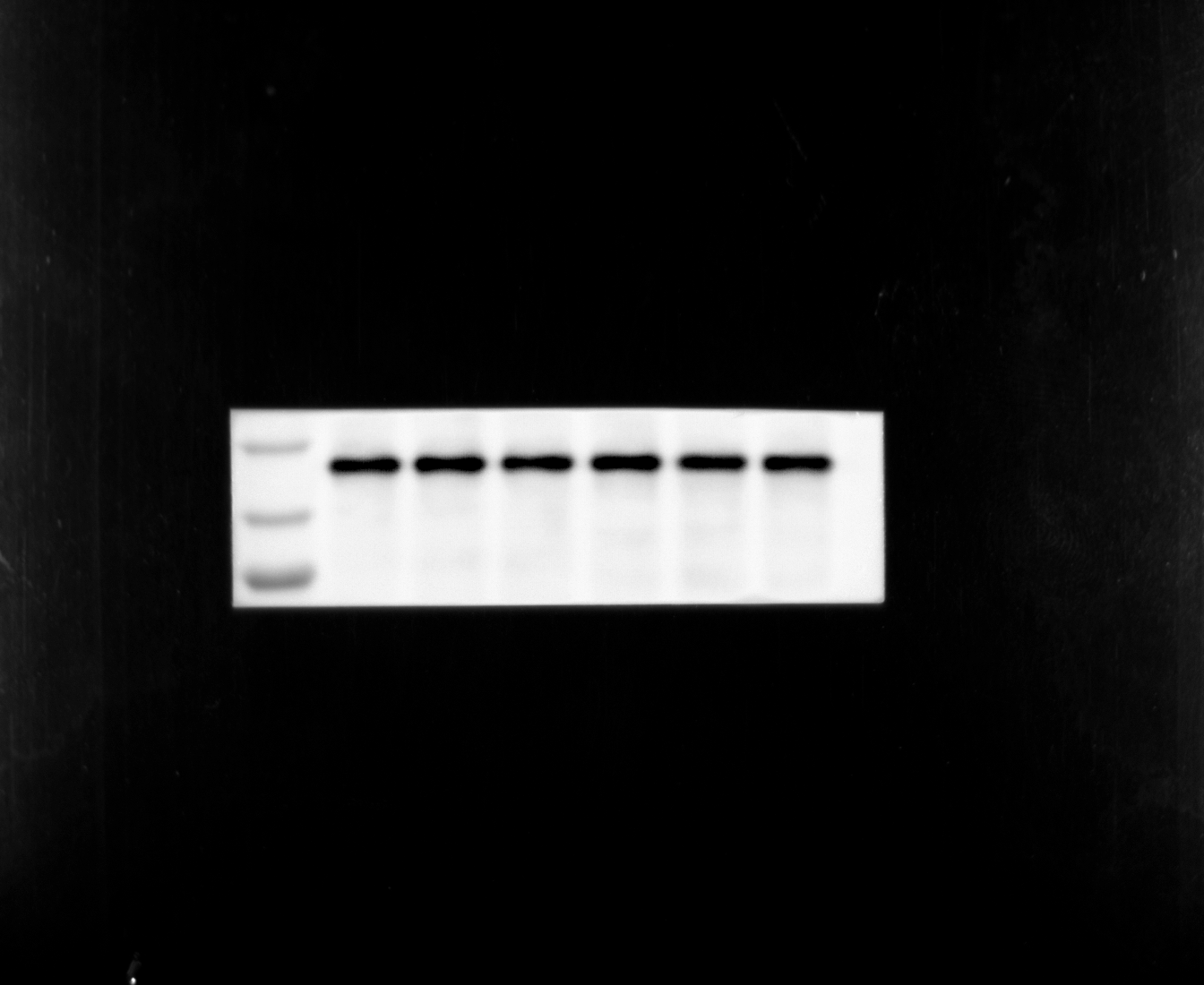

Supplement: Supplementary file 1 [file cimb-47-00936-s001.zip › cimb-3956315-supplementary/APOC2_ccRCC_RawWB_FullMembranes/cropped display images/11/β- actin/5.Tif]

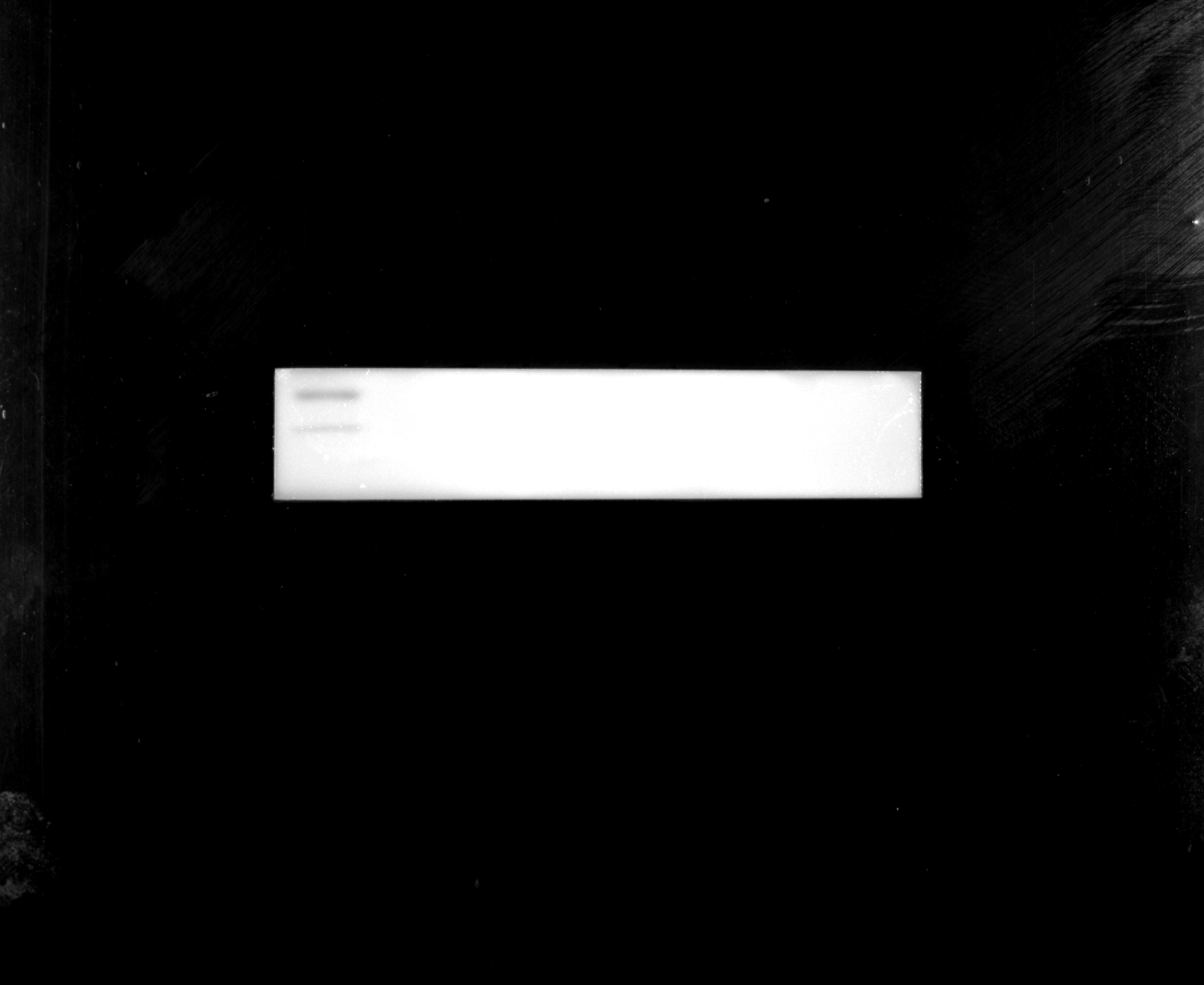

Supplement: Supplementary file 1 [file cimb-47-00936-s001.zip › cimb-3956315-supplementary/APOC2_ccRCC_RawWB_FullMembranes/cropped display images/12/Fig 3K APOC2/0.Tif]

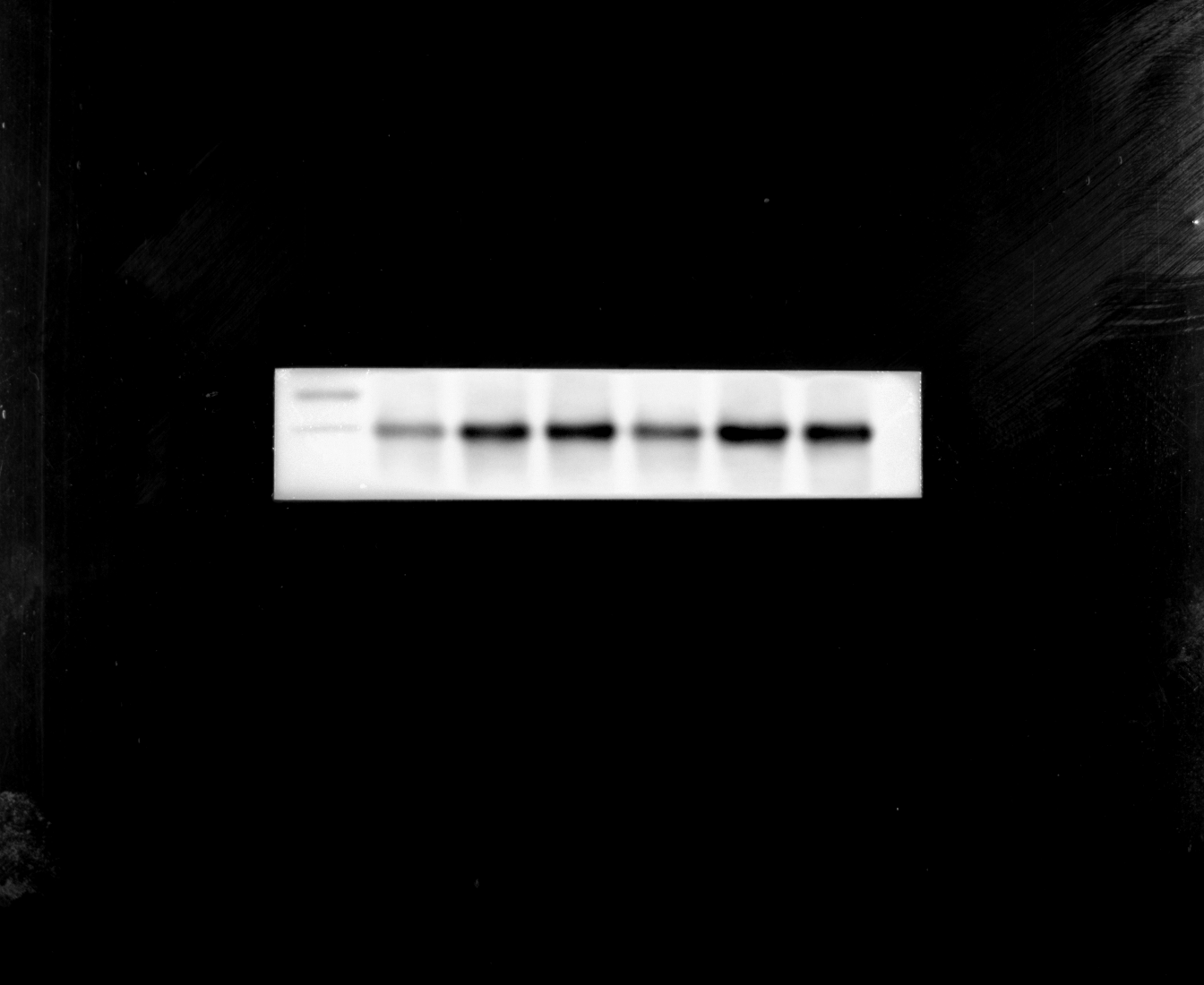

Supplement: Supplementary file 1 [file cimb-47-00936-s001.zip › cimb-3956315-supplementary/APOC2_ccRCC_RawWB_FullMembranes/cropped display images/12/Fig 3K APOC2/1.Tif]

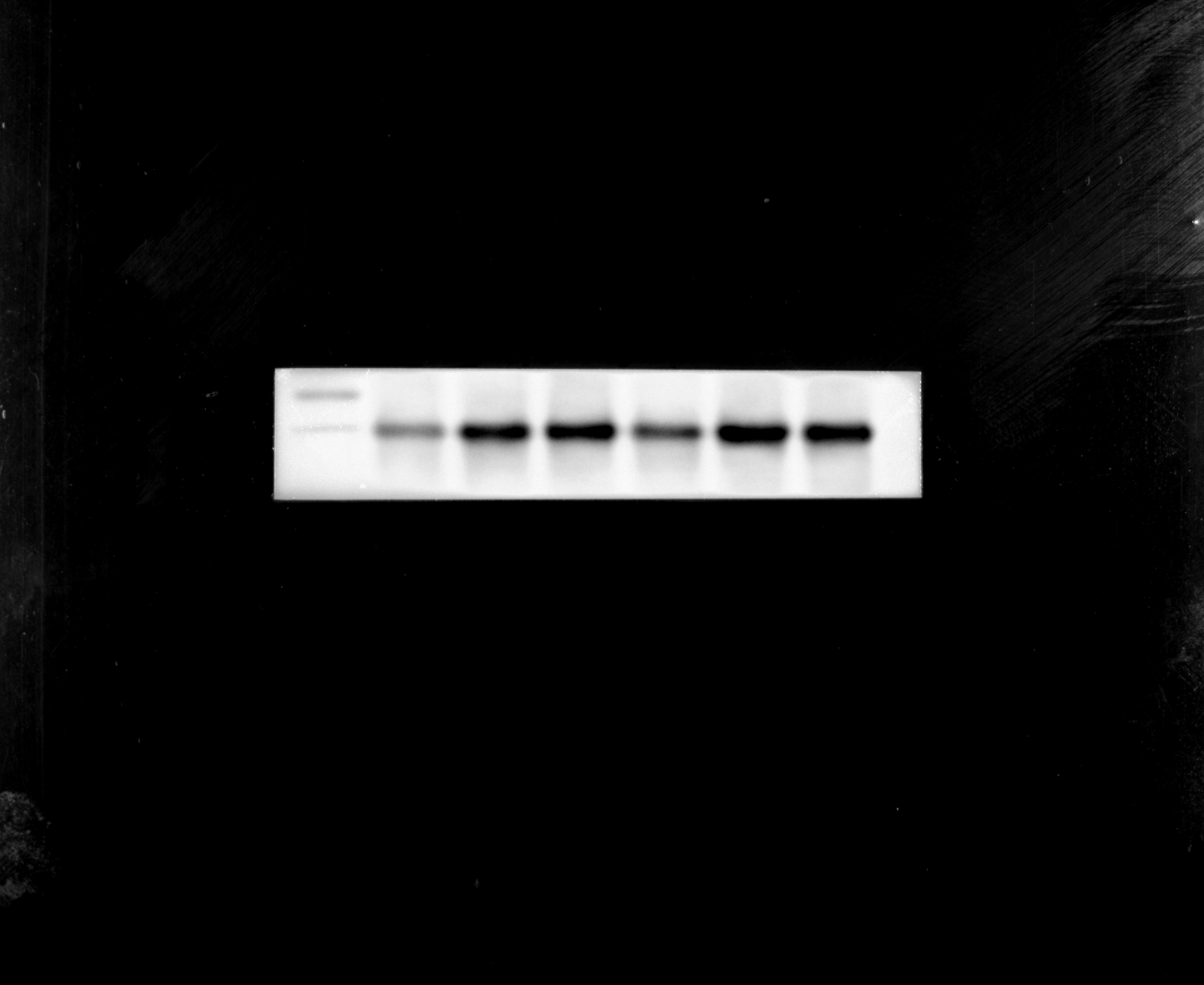

Supplement: Supplementary file 1 [file cimb-47-00936-s001.zip › cimb-3956315-supplementary/APOC2_ccRCC_RawWB_FullMembranes/cropped display images/12/Fig 3K APOC2/2.Tif]

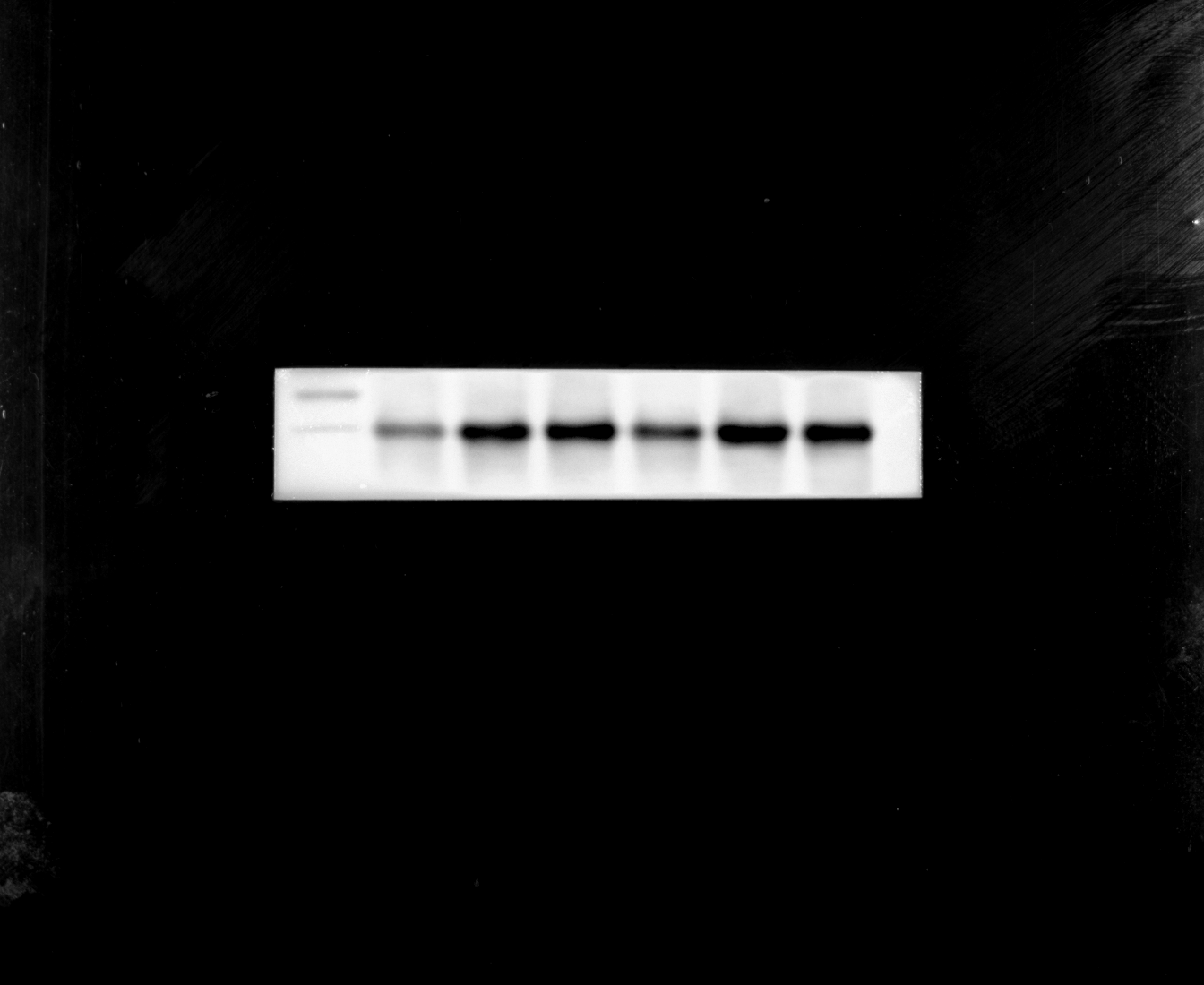

Supplement: Supplementary file 1 [file cimb-47-00936-s001.zip › cimb-3956315-supplementary/APOC2_ccRCC_RawWB_FullMembranes/cropped display images/12/Fig 3K APOC2/3.Tif]

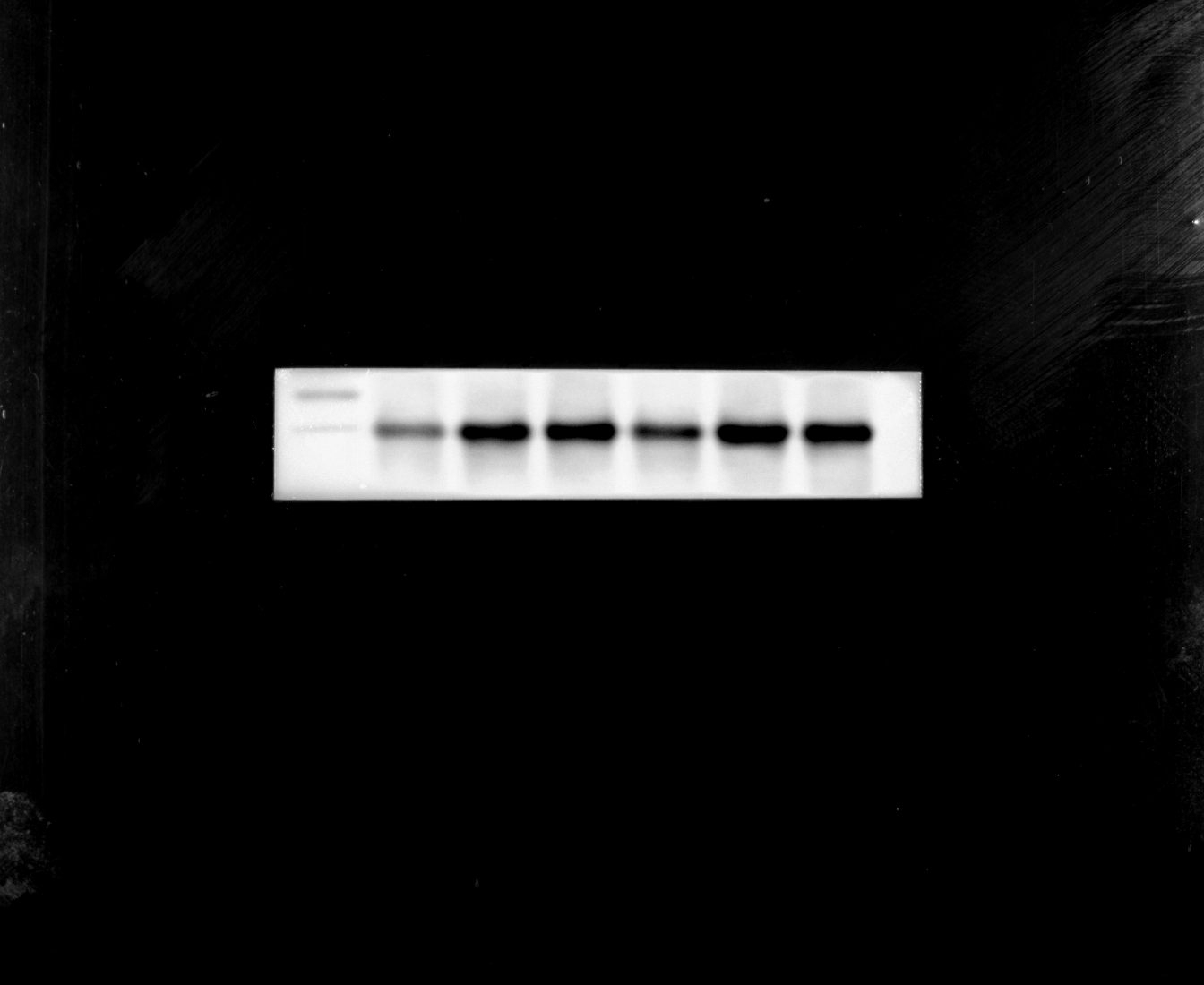

Supplement: Supplementary file 1 [file cimb-47-00936-s001.zip › cimb-3956315-supplementary/APOC2_ccRCC_RawWB_FullMembranes/cropped display images/12/Fig 3K APOC2/4.Tif]

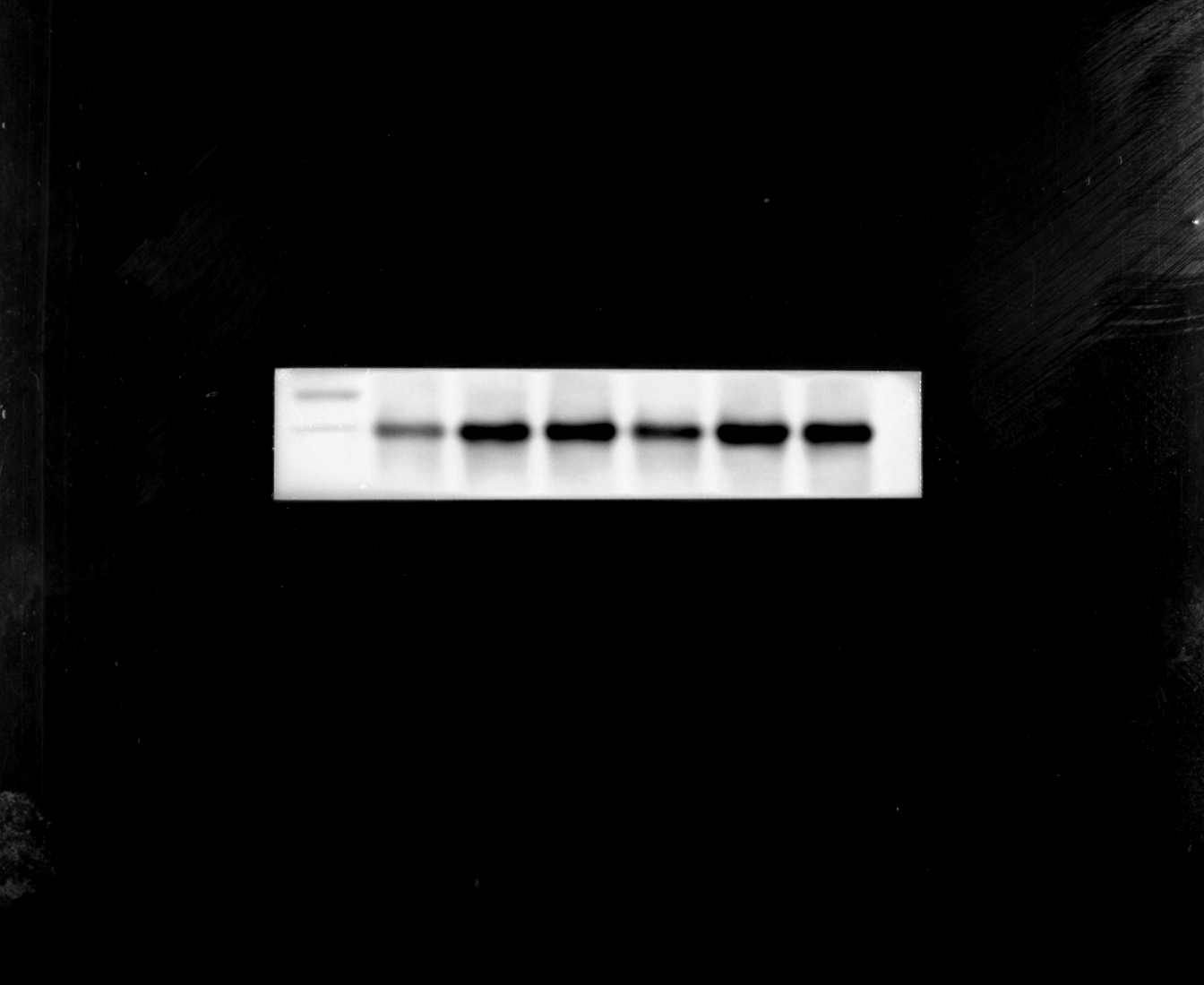

Supplement: Supplementary file 1 [file cimb-47-00936-s001.zip › cimb-3956315-supplementary/APOC2_ccRCC_RawWB_FullMembranes/cropped display images/12/Fig 3K APOC2/5.Tif]

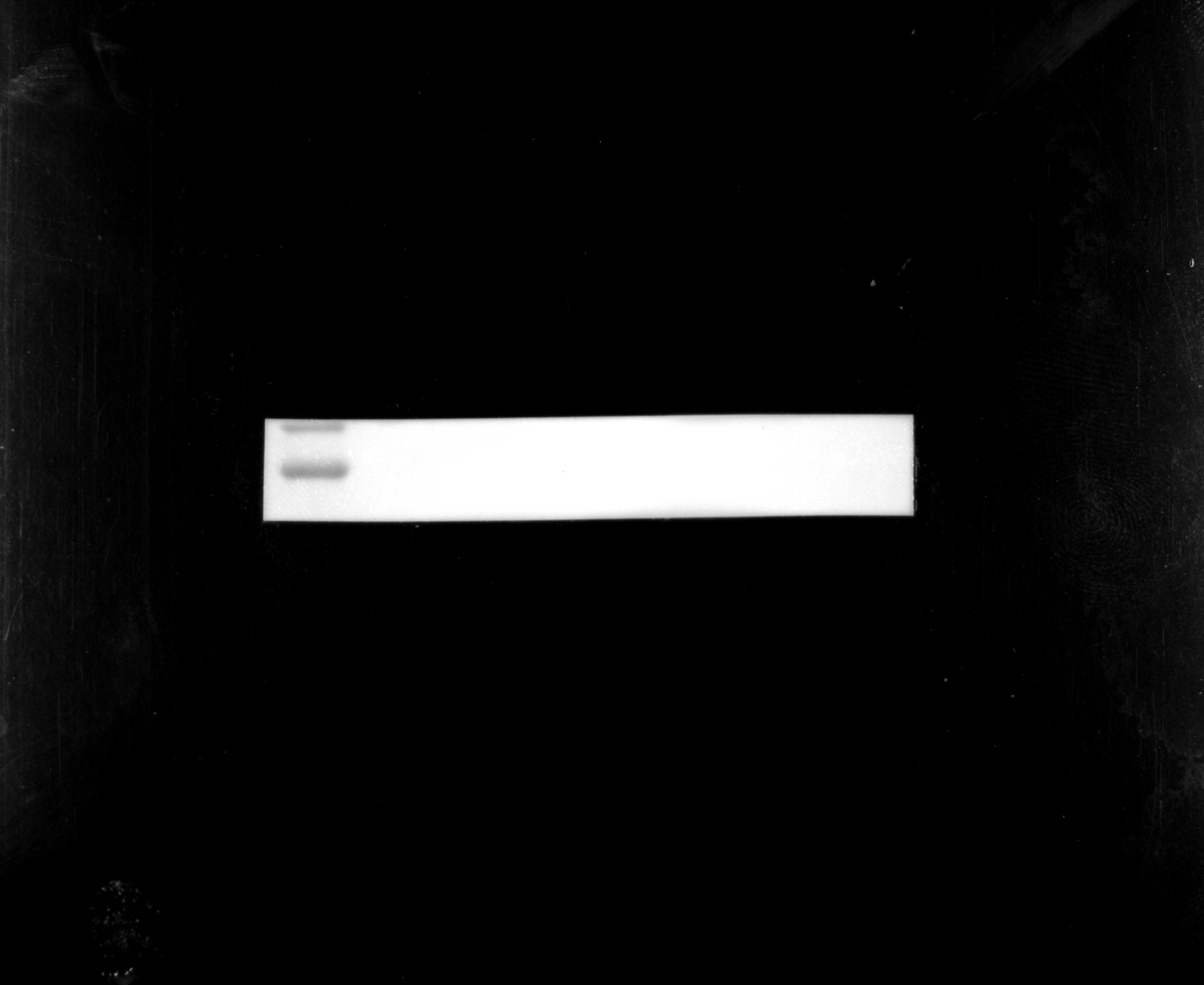

Supplement: Supplementary file 1 [file cimb-47-00936-s001.zip › cimb-3956315-supplementary/APOC2_ccRCC_RawWB_FullMembranes/cropped display images/12/Fig 3K c-caspase3/0.Tif]

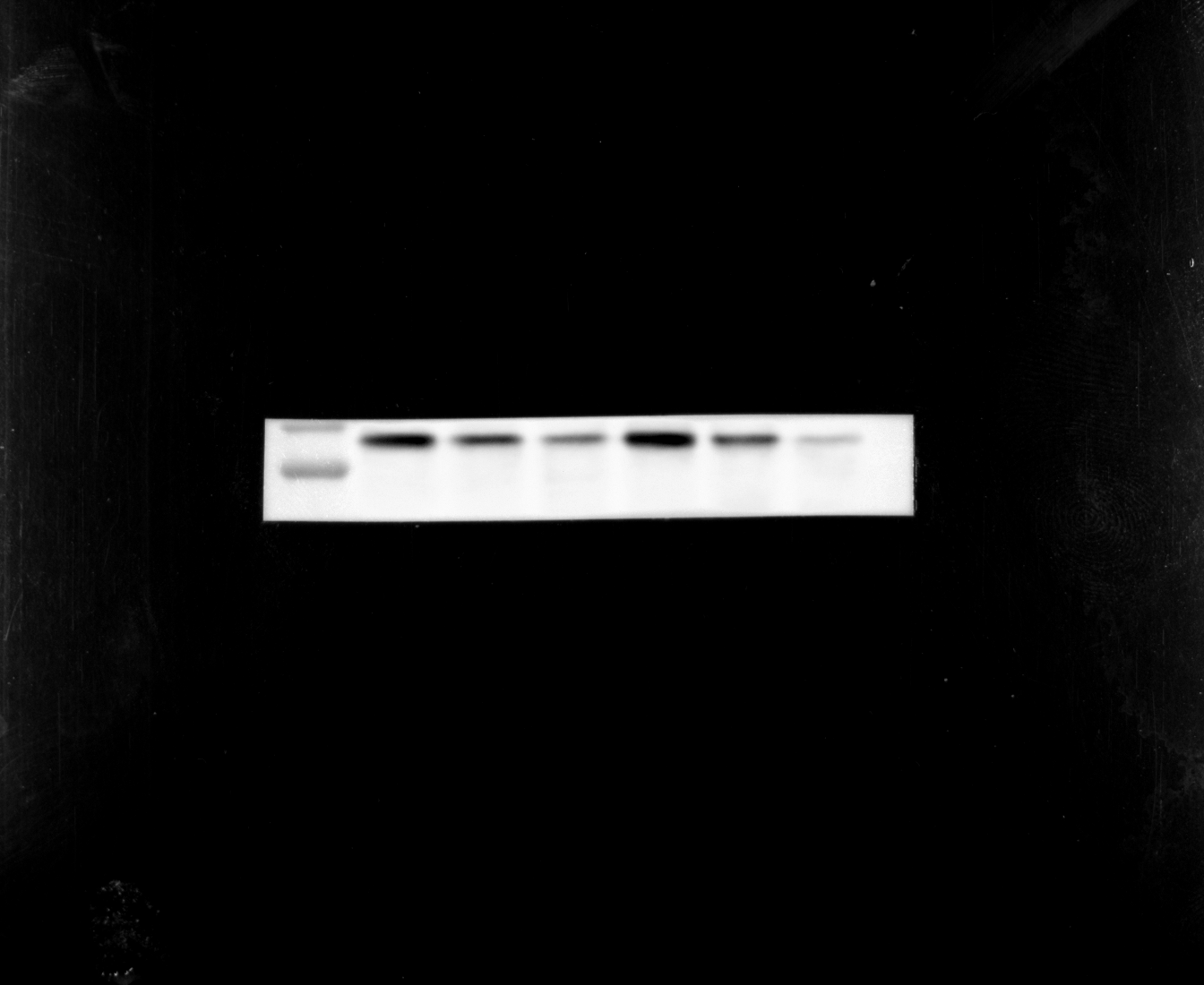

Supplement: Supplementary file 1 [file cimb-47-00936-s001.zip › cimb-3956315-supplementary/APOC2_ccRCC_RawWB_FullMembranes/cropped display images/12/Fig 3K c-caspase3/1.Tif]

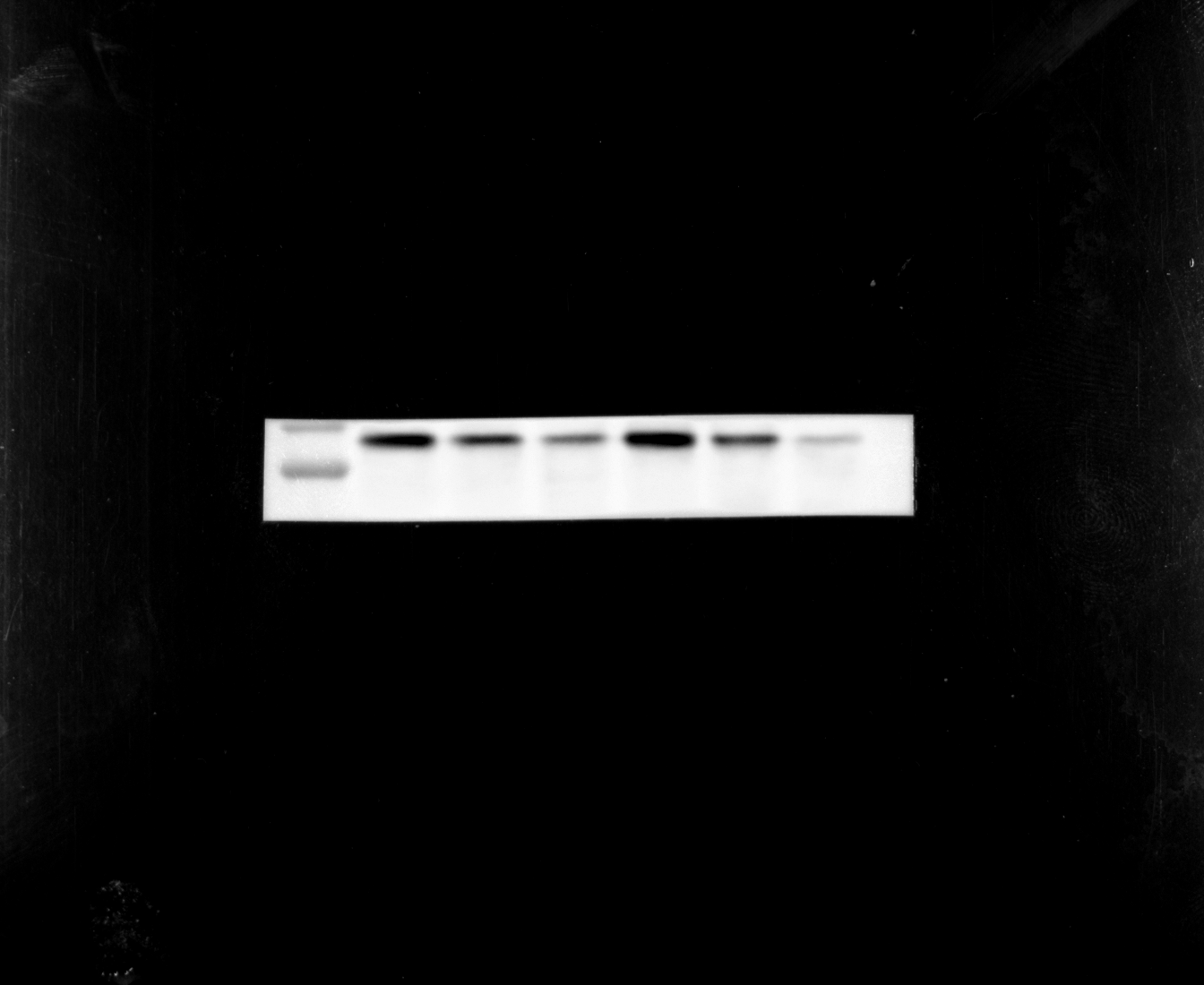

Supplement: Supplementary file 1 [file cimb-47-00936-s001.zip › cimb-3956315-supplementary/APOC2_ccRCC_RawWB_FullMembranes/cropped display images/12/Fig 3K c-caspase3/2.Tif]

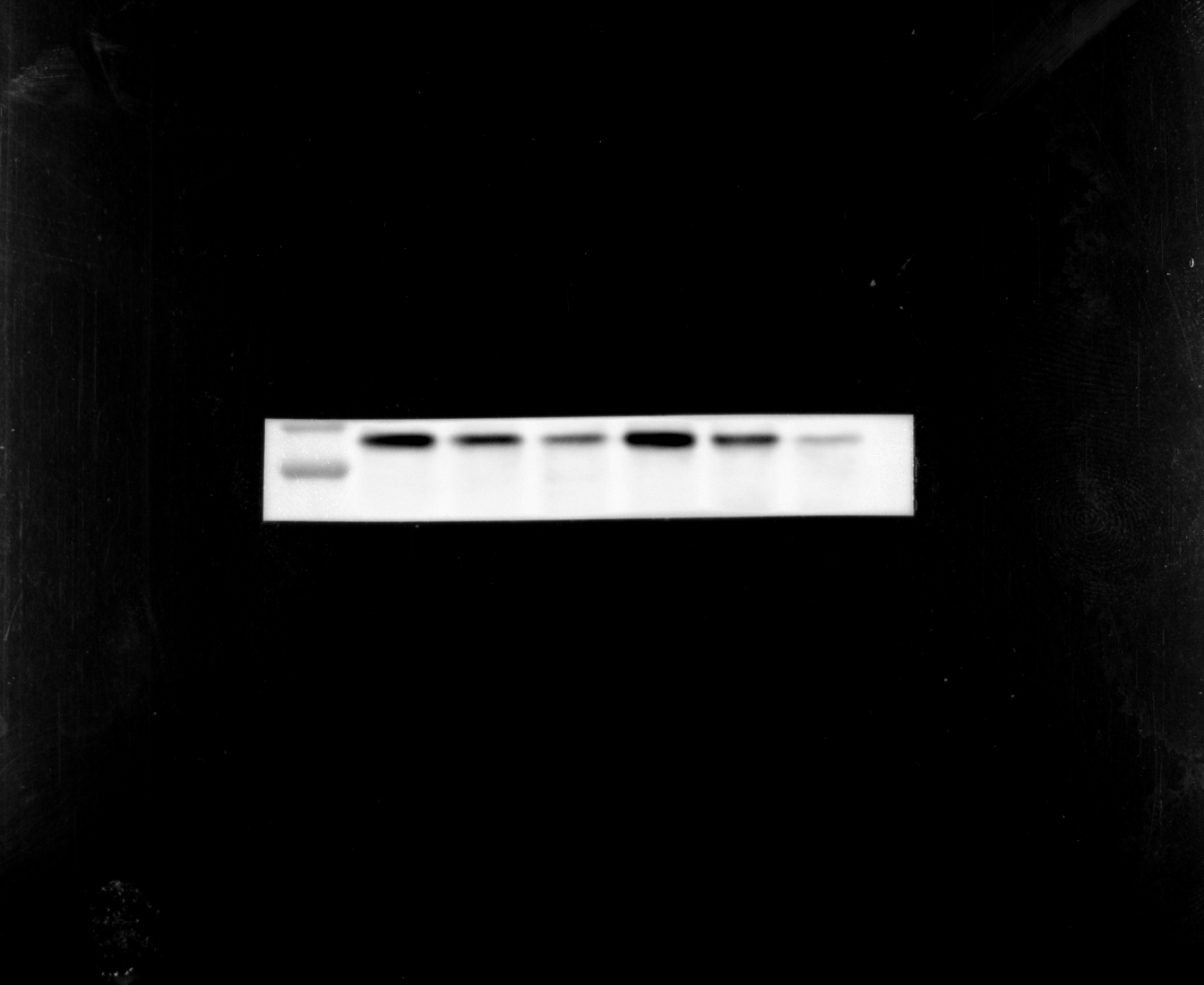

Supplement: Supplementary file 1 [file cimb-47-00936-s001.zip › cimb-3956315-supplementary/APOC2_ccRCC_RawWB_FullMembranes/cropped display images/12/Fig 3K c-caspase3/3.Tif]

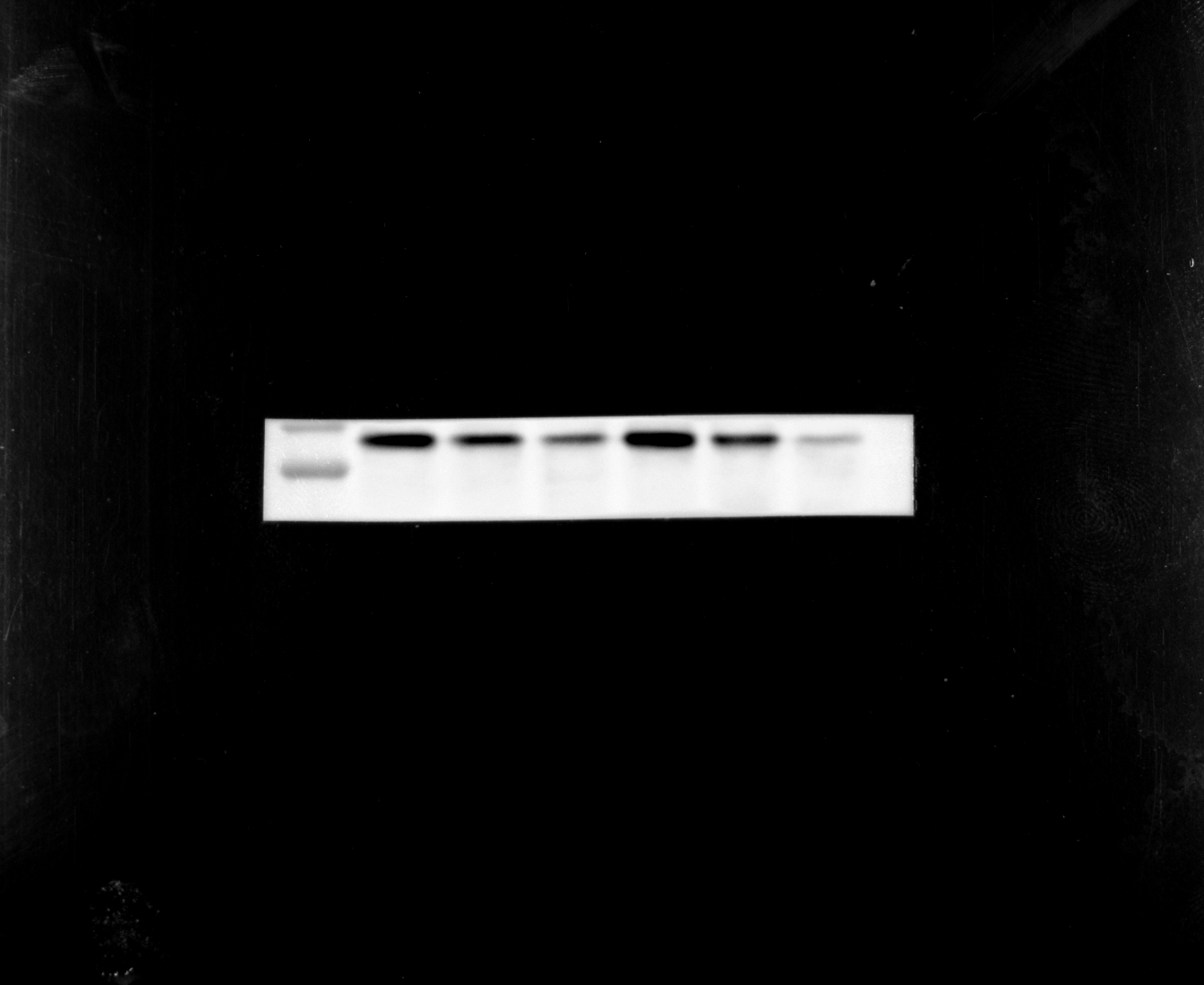

Supplement: Supplementary file 1 [file cimb-47-00936-s001.zip › cimb-3956315-supplementary/APOC2_ccRCC_RawWB_FullMembranes/cropped display images/12/Fig 3K c-caspase3/4.Tif]

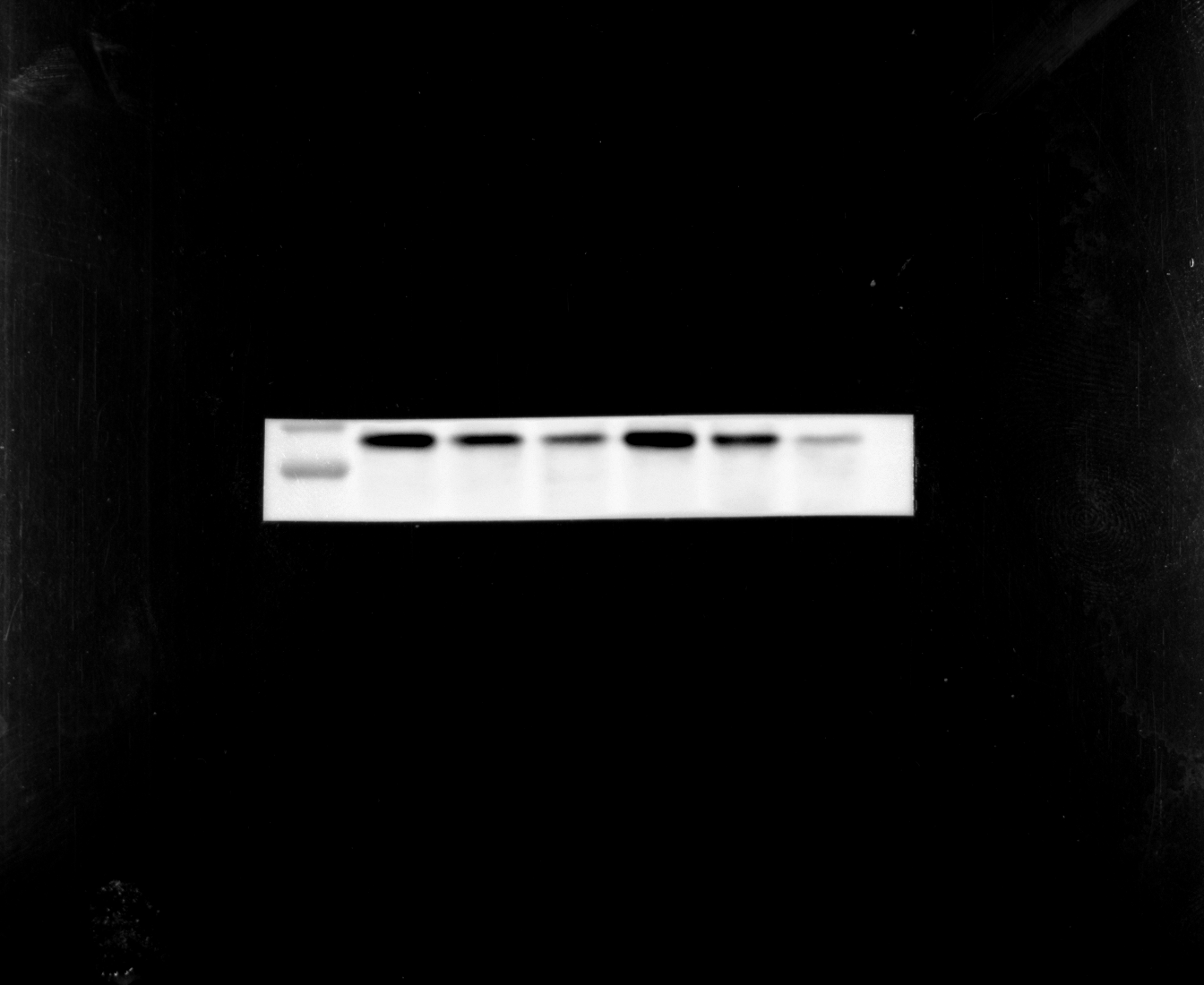

Supplement: Supplementary file 1 [file cimb-47-00936-s001.zip › cimb-3956315-supplementary/APOC2_ccRCC_RawWB_FullMembranes/cropped display images/12/Fig 3K c-caspase3/5.Tif]

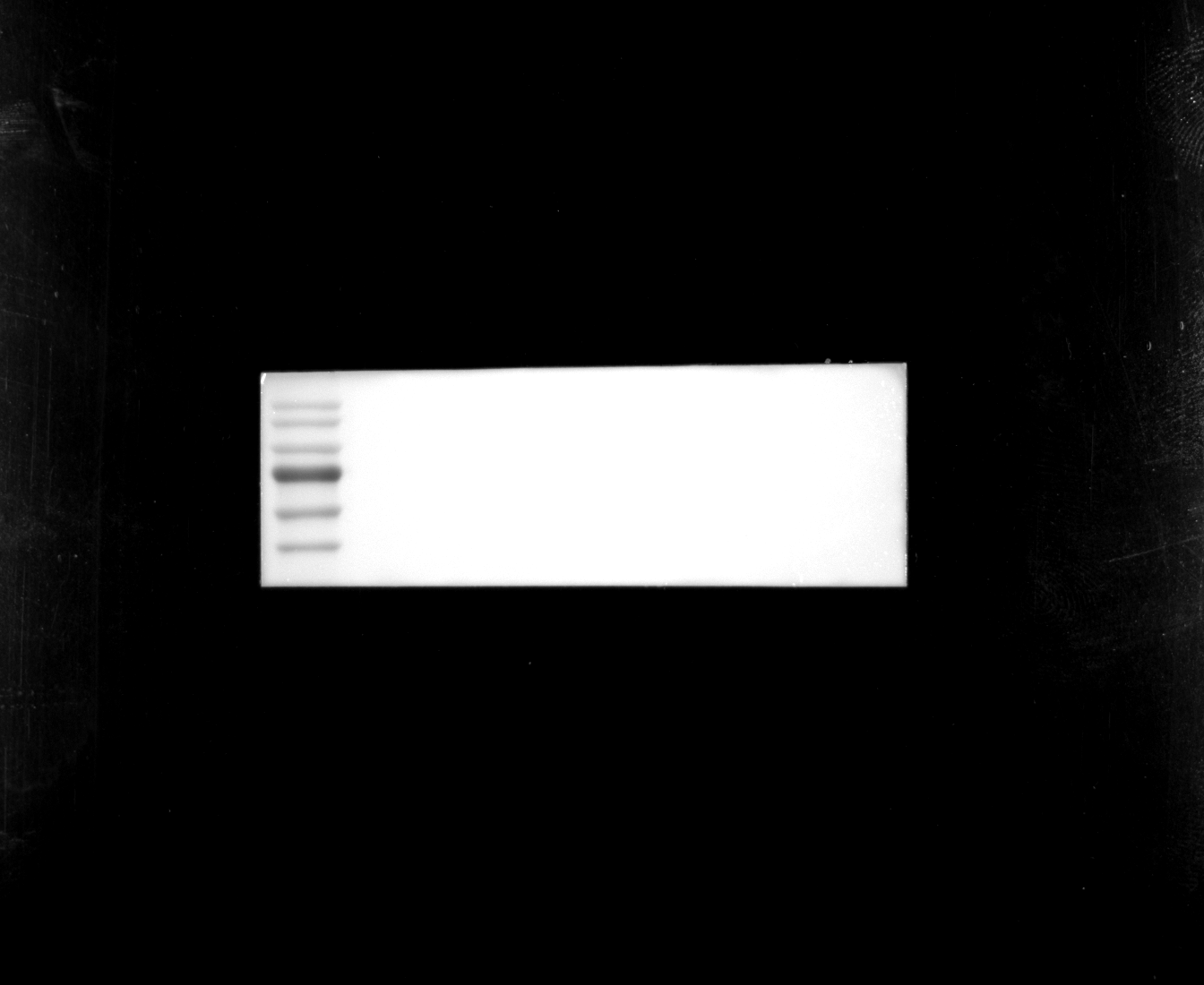

Supplement: Supplementary file 1 [file cimb-47-00936-s001.zip › cimb-3956315-supplementary/APOC2_ccRCC_RawWB_FullMembranes/cropped display images/12/Fig 3K β- actin/0.Tif]

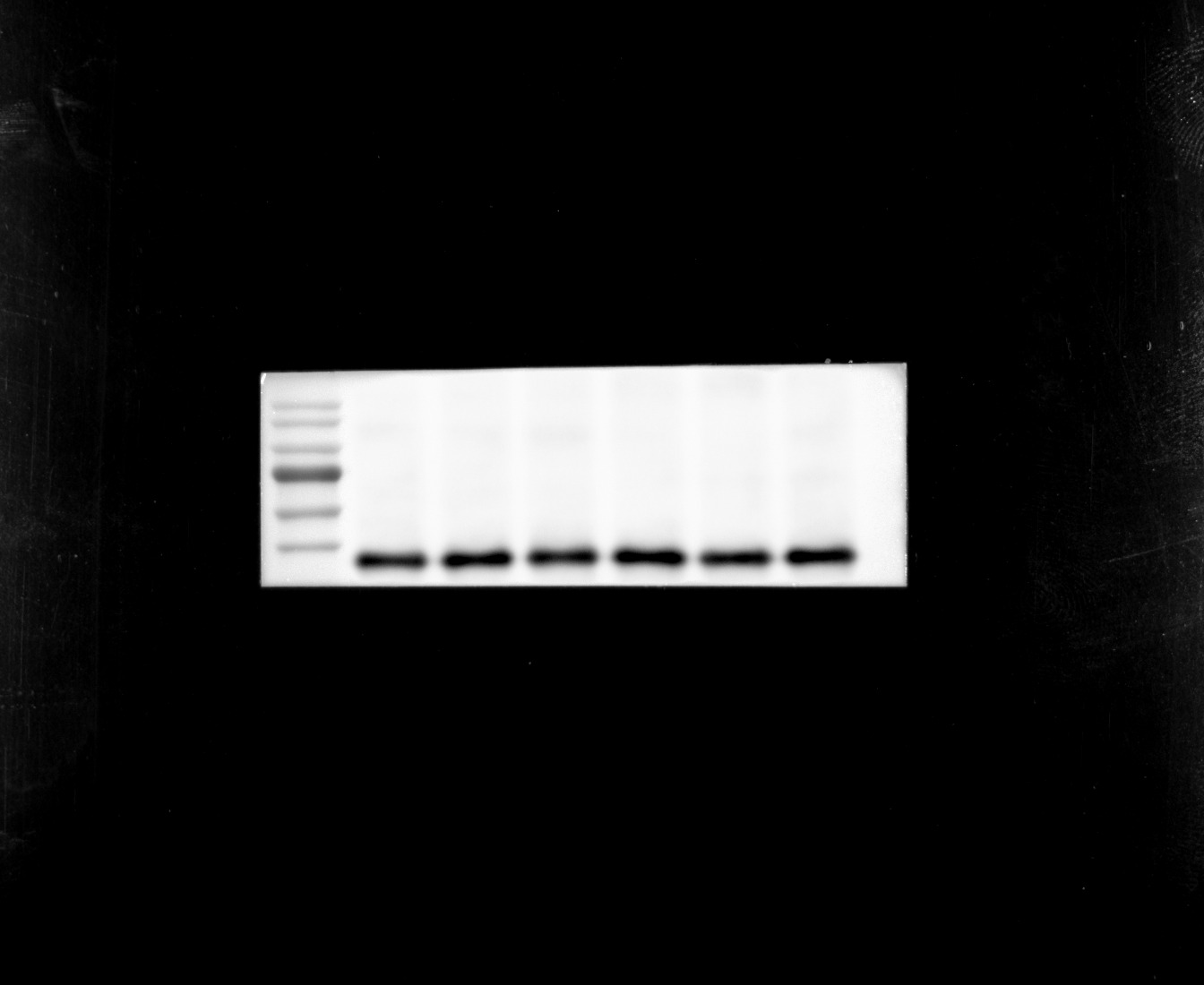

Supplement: Supplementary file 1 [file cimb-47-00936-s001.zip › cimb-3956315-supplementary/APOC2_ccRCC_RawWB_FullMembranes/cropped display images/12/Fig 3K β- actin/1.Tif]

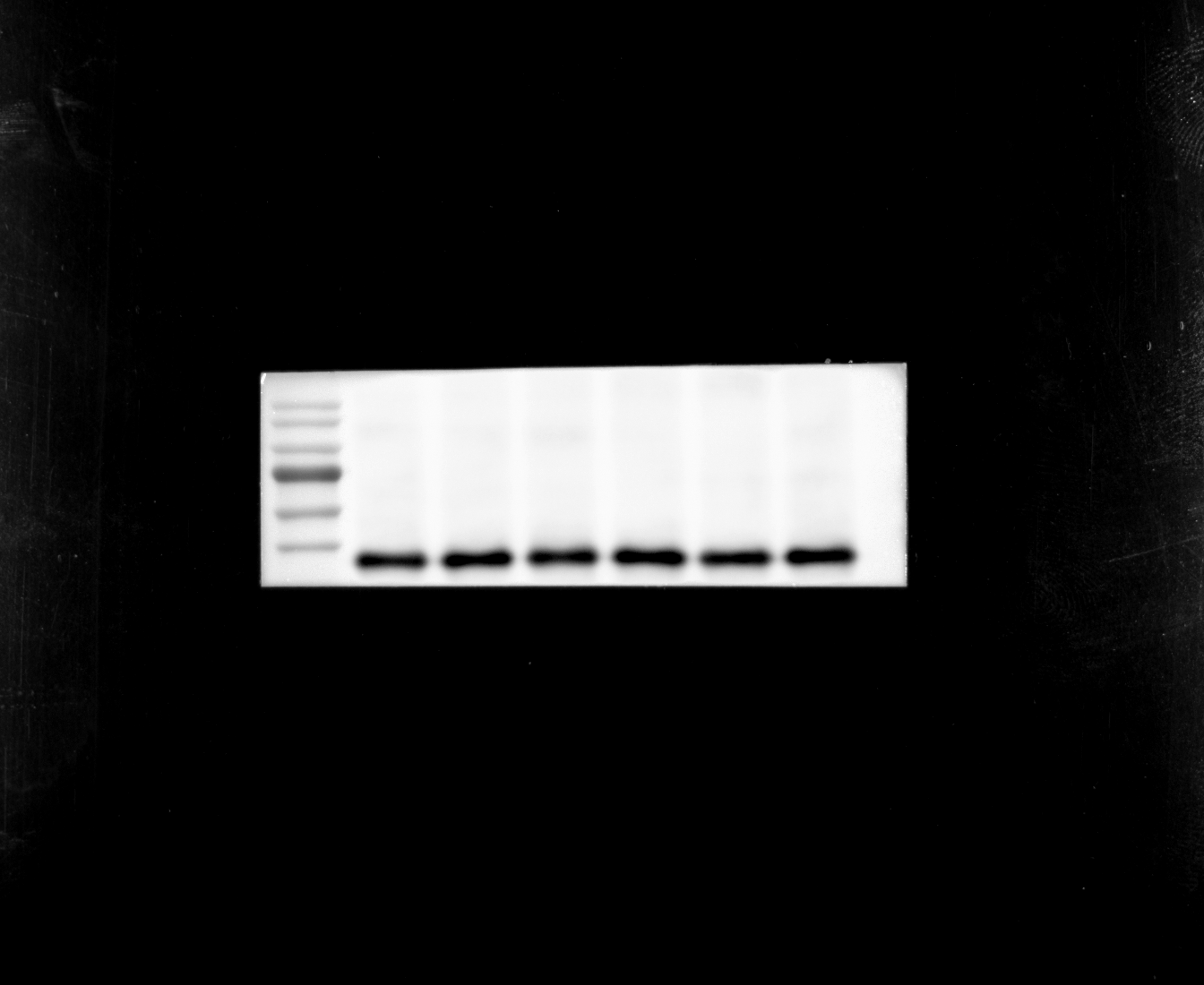

Supplement: Supplementary file 1 [file cimb-47-00936-s001.zip › cimb-3956315-supplementary/APOC2_ccRCC_RawWB_FullMembranes/cropped display images/12/Fig 3K β- actin/2.Tif]

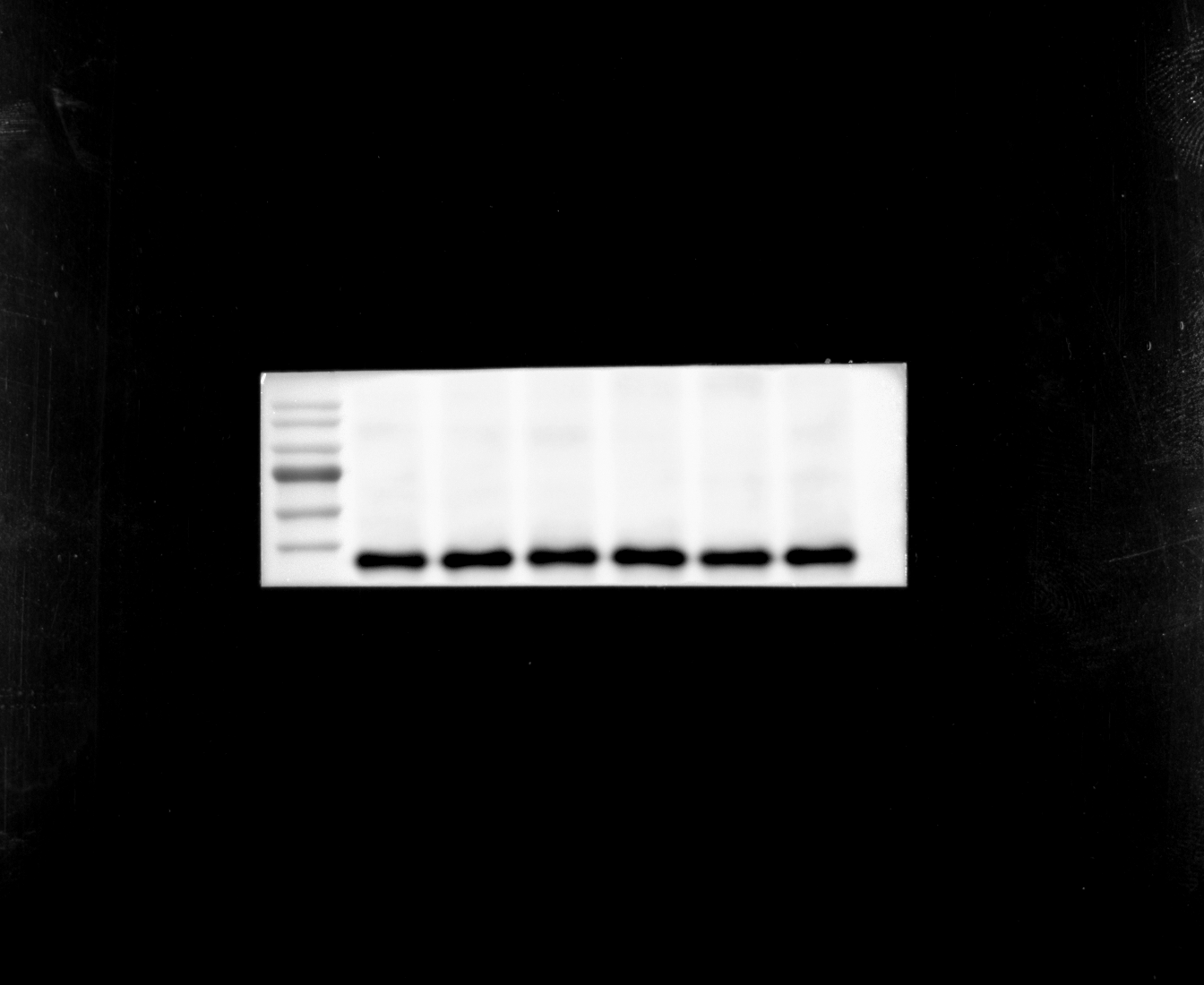

Supplement: Supplementary file 1 [file cimb-47-00936-s001.zip › cimb-3956315-supplementary/APOC2_ccRCC_RawWB_FullMembranes/cropped display images/12/Fig 3K β- actin/3.Tif]

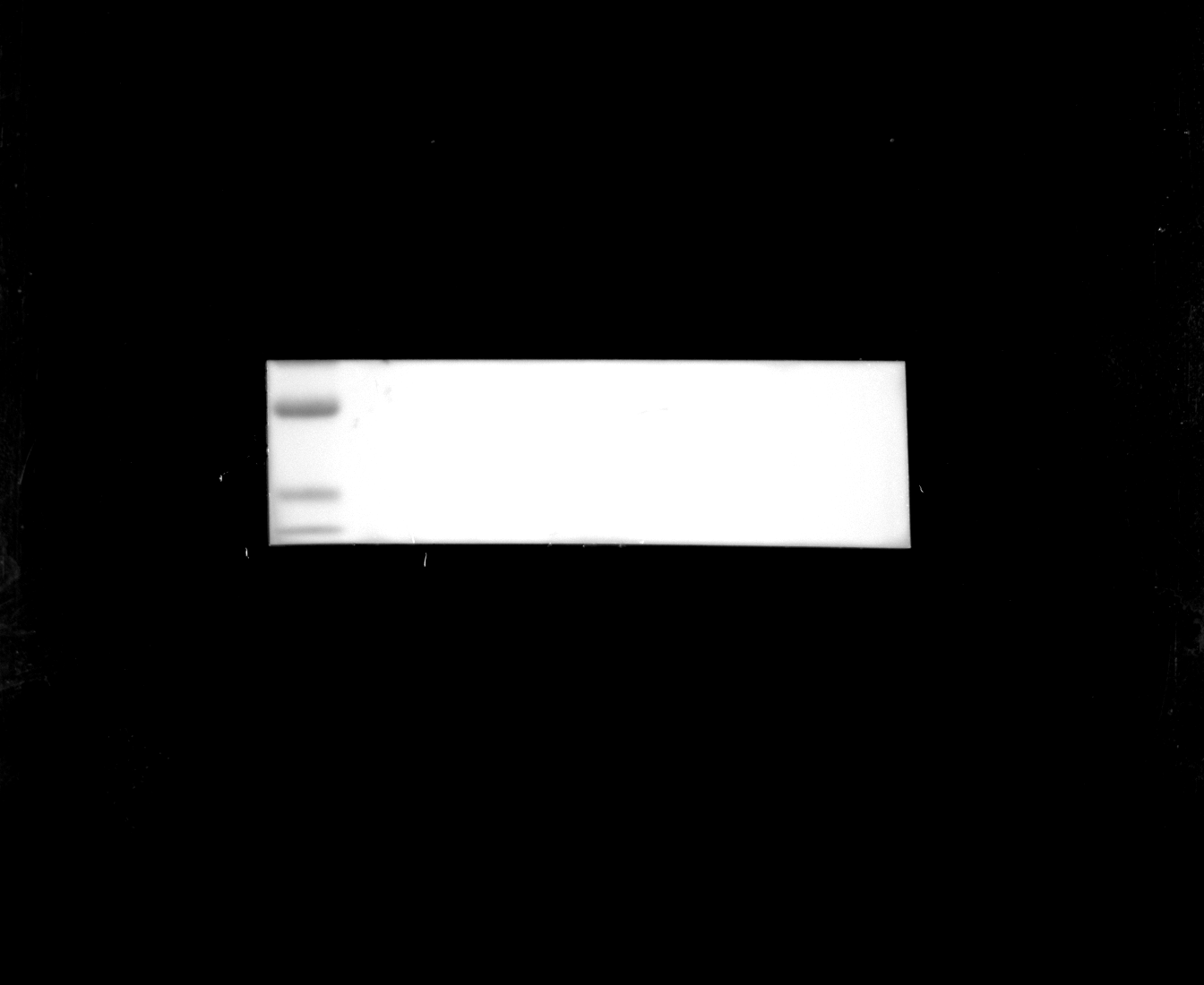

Supplement: Supplementary file 1 [file cimb-47-00936-s001.zip › cimb-3956315-supplementary/APOC2_ccRCC_RawWB_FullMembranes/cropped display images/13/Fig 3K BCL2/0.Tif]

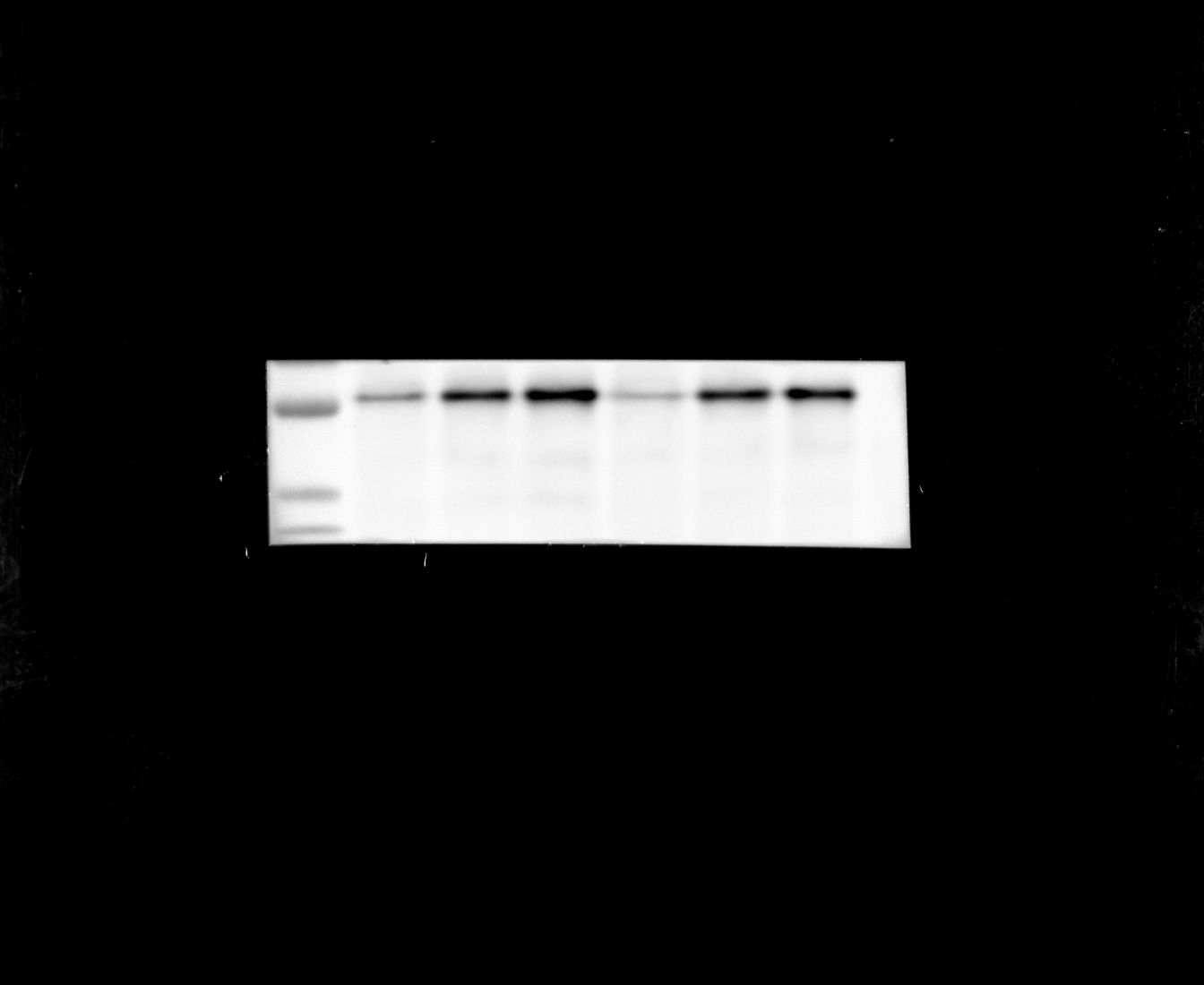

Supplement: Supplementary file 1 [file cimb-47-00936-s001.zip › cimb-3956315-supplementary/APOC2_ccRCC_RawWB_FullMembranes/cropped display images/13/Fig 3K BCL2/1.Tif]

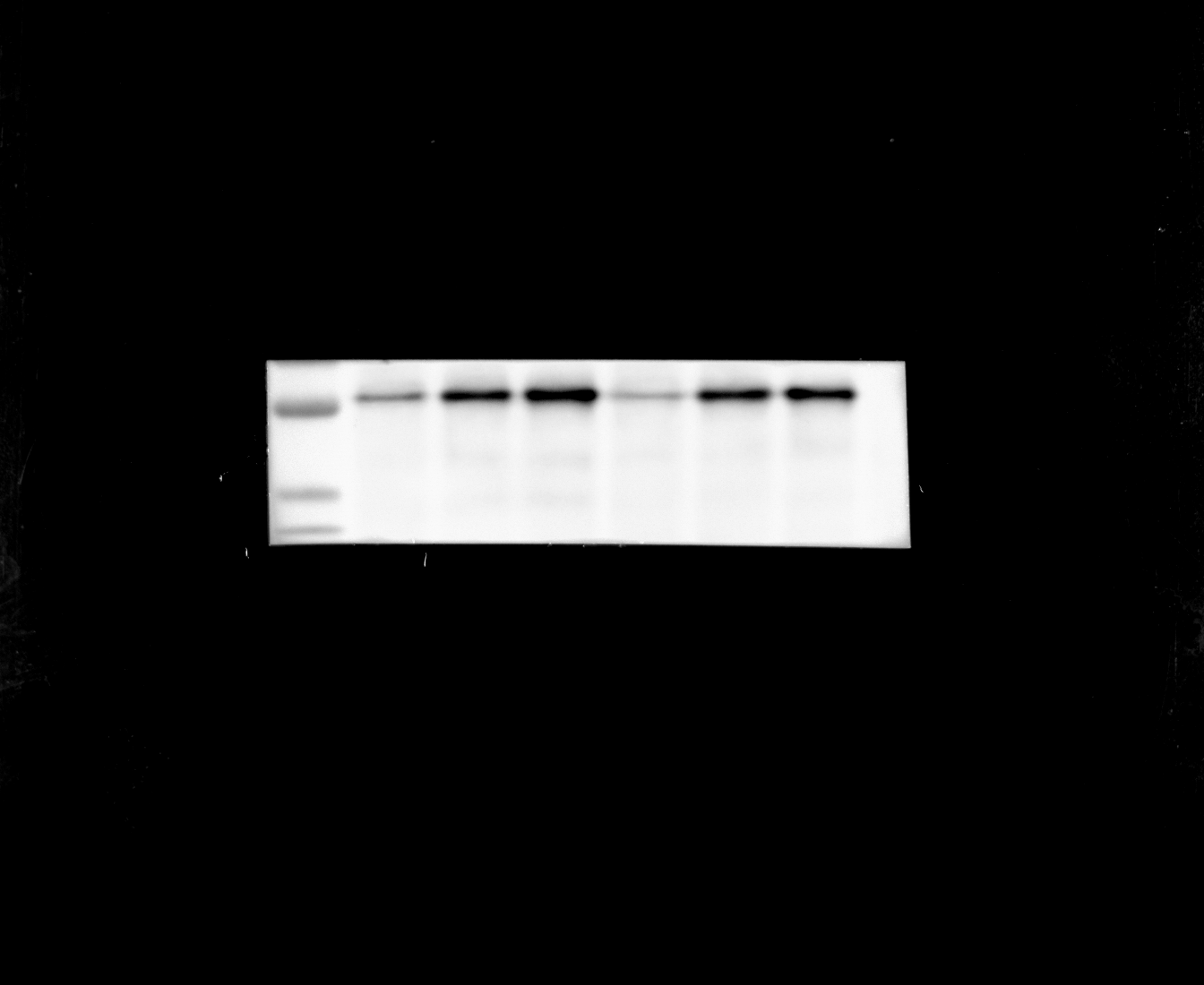

Supplement: Supplementary file 1 [file cimb-47-00936-s001.zip › cimb-3956315-supplementary/APOC2_ccRCC_RawWB_FullMembranes/cropped display images/13/Fig 3K BCL2/2.Tif]

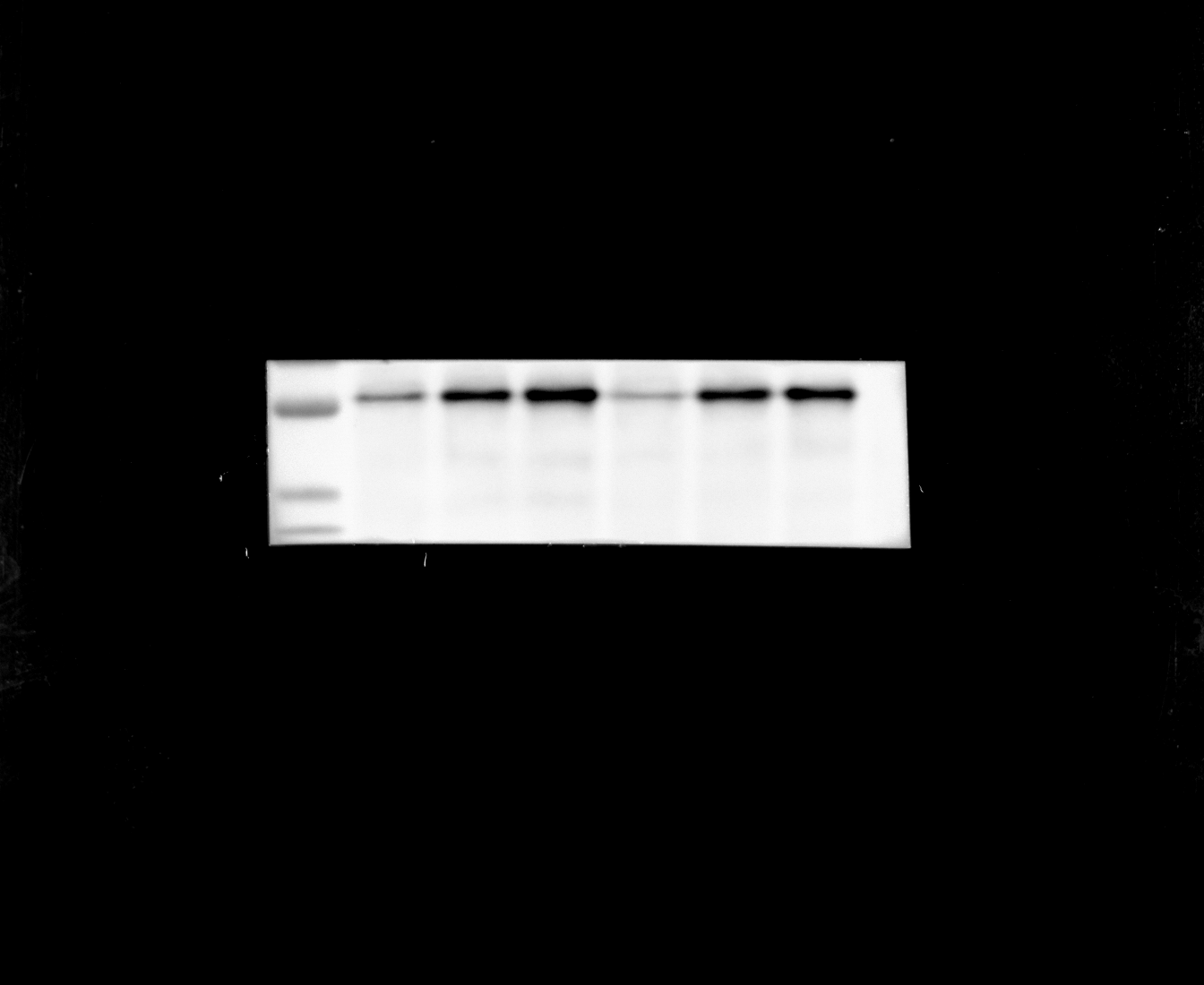

Supplement: Supplementary file 1 [file cimb-47-00936-s001.zip › cimb-3956315-supplementary/APOC2_ccRCC_RawWB_FullMembranes/cropped display images/13/Fig 3K BCL2/3.Tif]

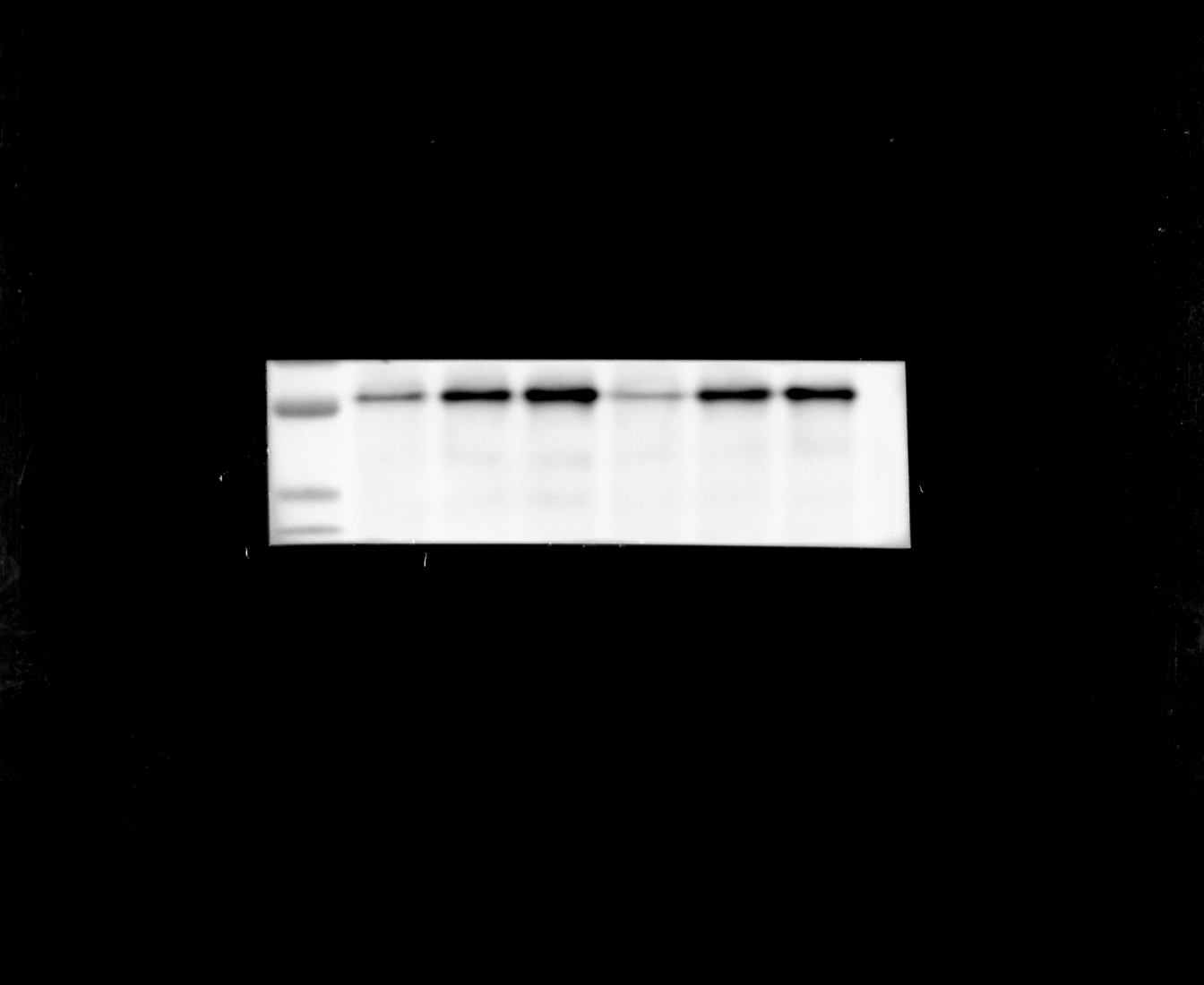

Supplement: Supplementary file 1 [file cimb-47-00936-s001.zip › cimb-3956315-supplementary/APOC2_ccRCC_RawWB_FullMembranes/cropped display images/13/Fig 3K BCL2/4.Tif]

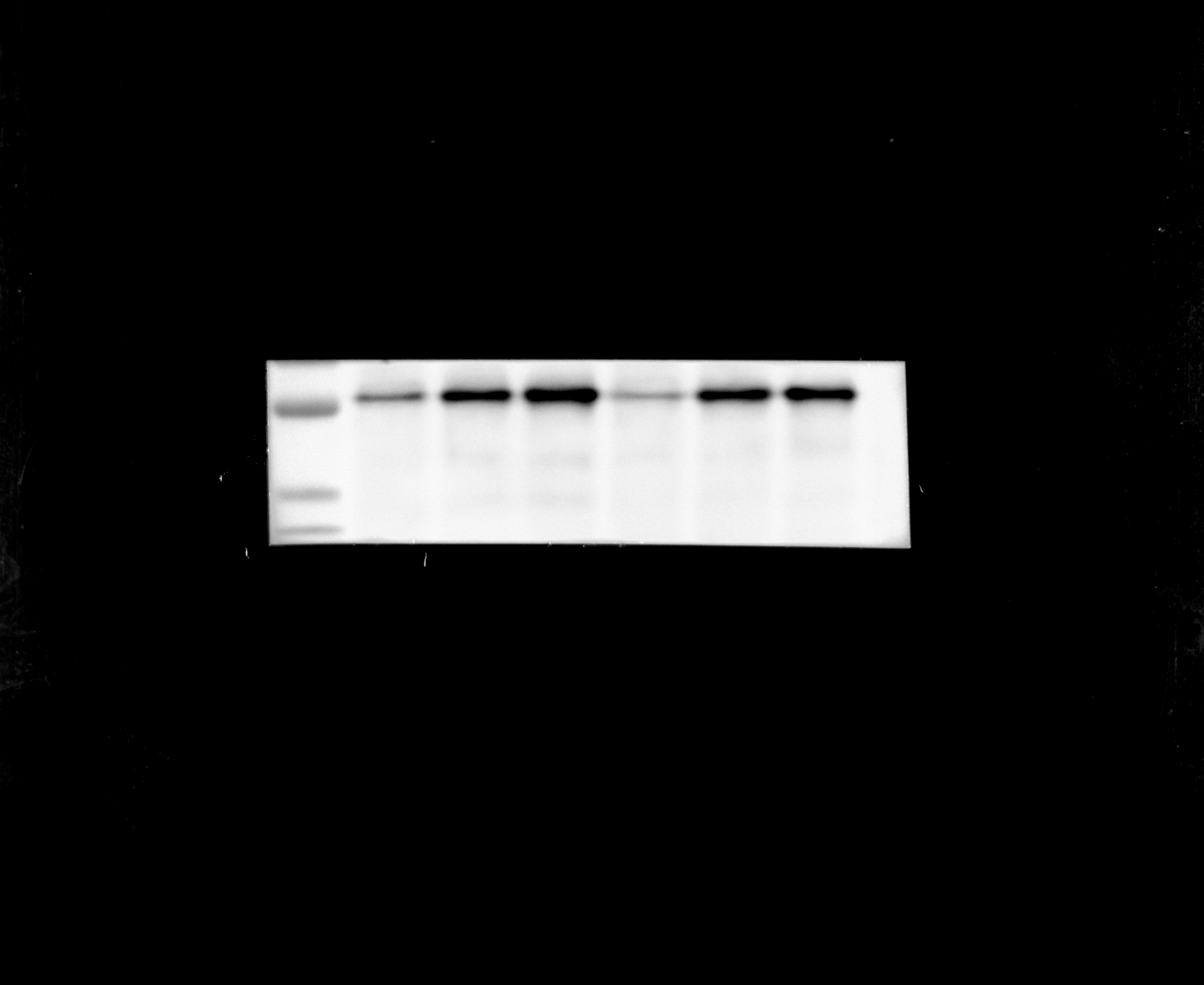

Supplement: Supplementary file 1 [file cimb-47-00936-s001.zip › cimb-3956315-supplementary/APOC2_ccRCC_RawWB_FullMembranes/cropped display images/13/Fig 3K BCL2/5.Tif]

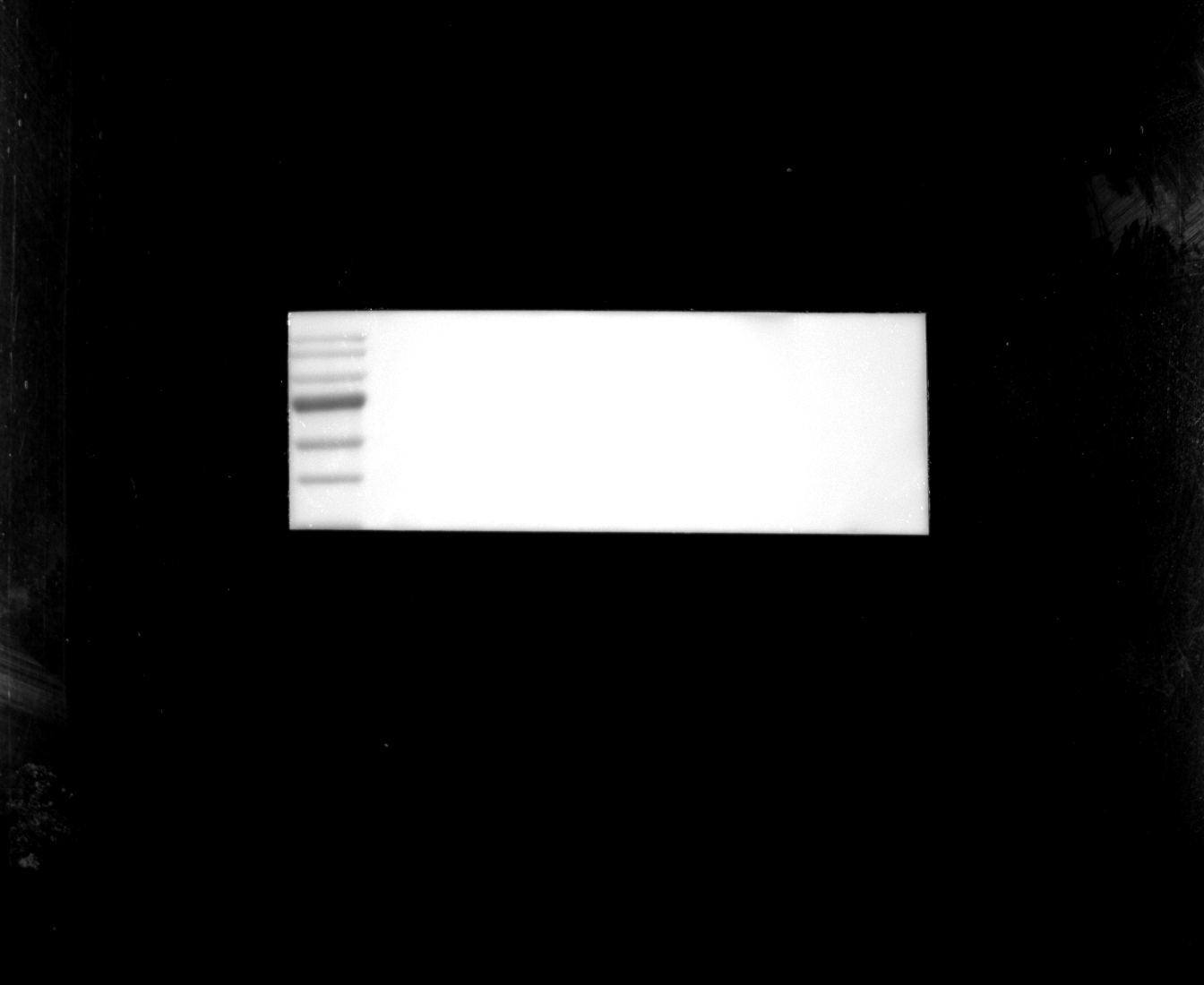

Supplement: Supplementary file 1 [file cimb-47-00936-s001.zip › cimb-3956315-supplementary/APOC2_ccRCC_RawWB_FullMembranes/cropped display images/13/β- actin/0.Tif]

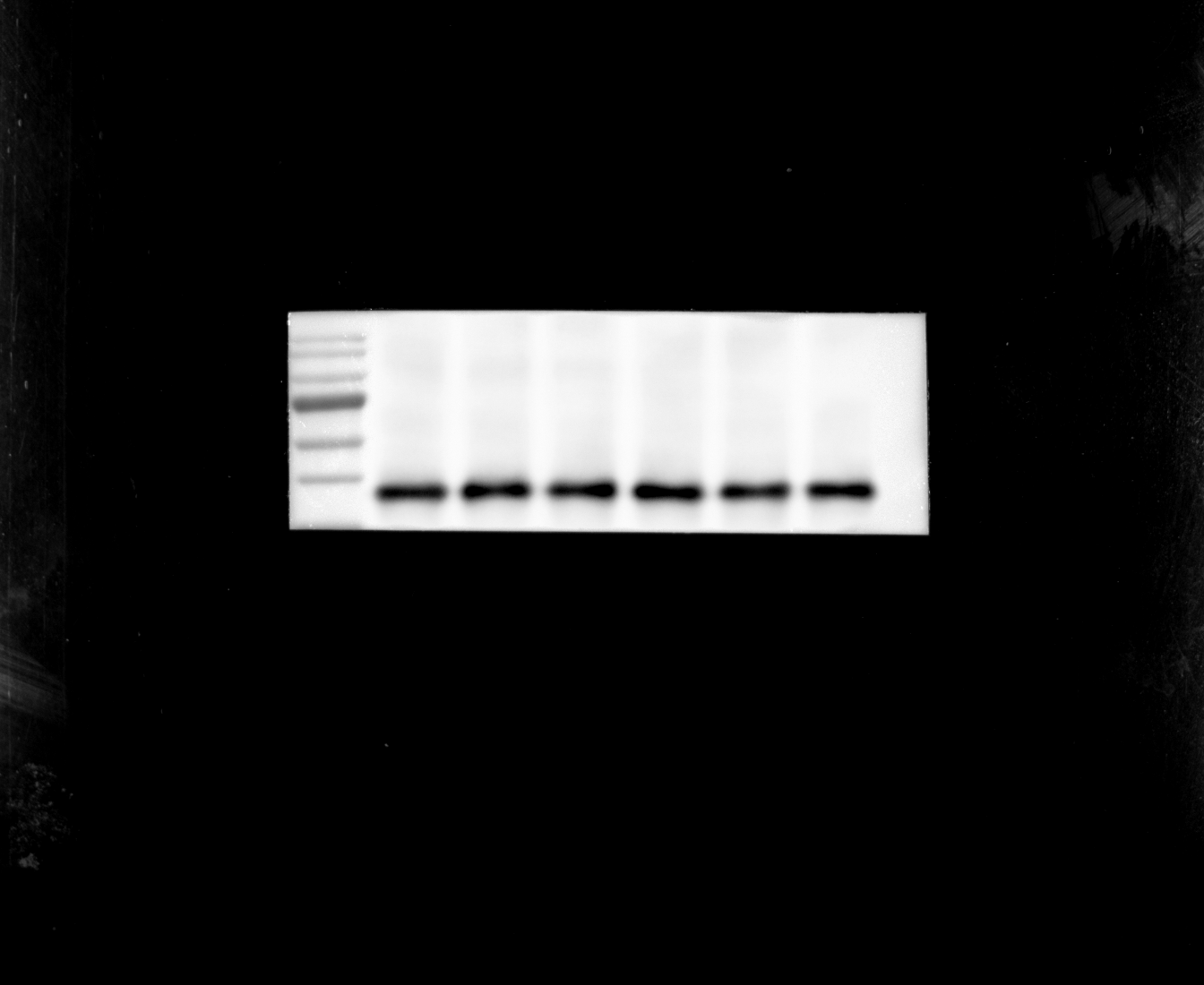

Supplement: Supplementary file 1 [file cimb-47-00936-s001.zip › cimb-3956315-supplementary/APOC2_ccRCC_RawWB_FullMembranes/cropped display images/13/β- actin/1.Tif]

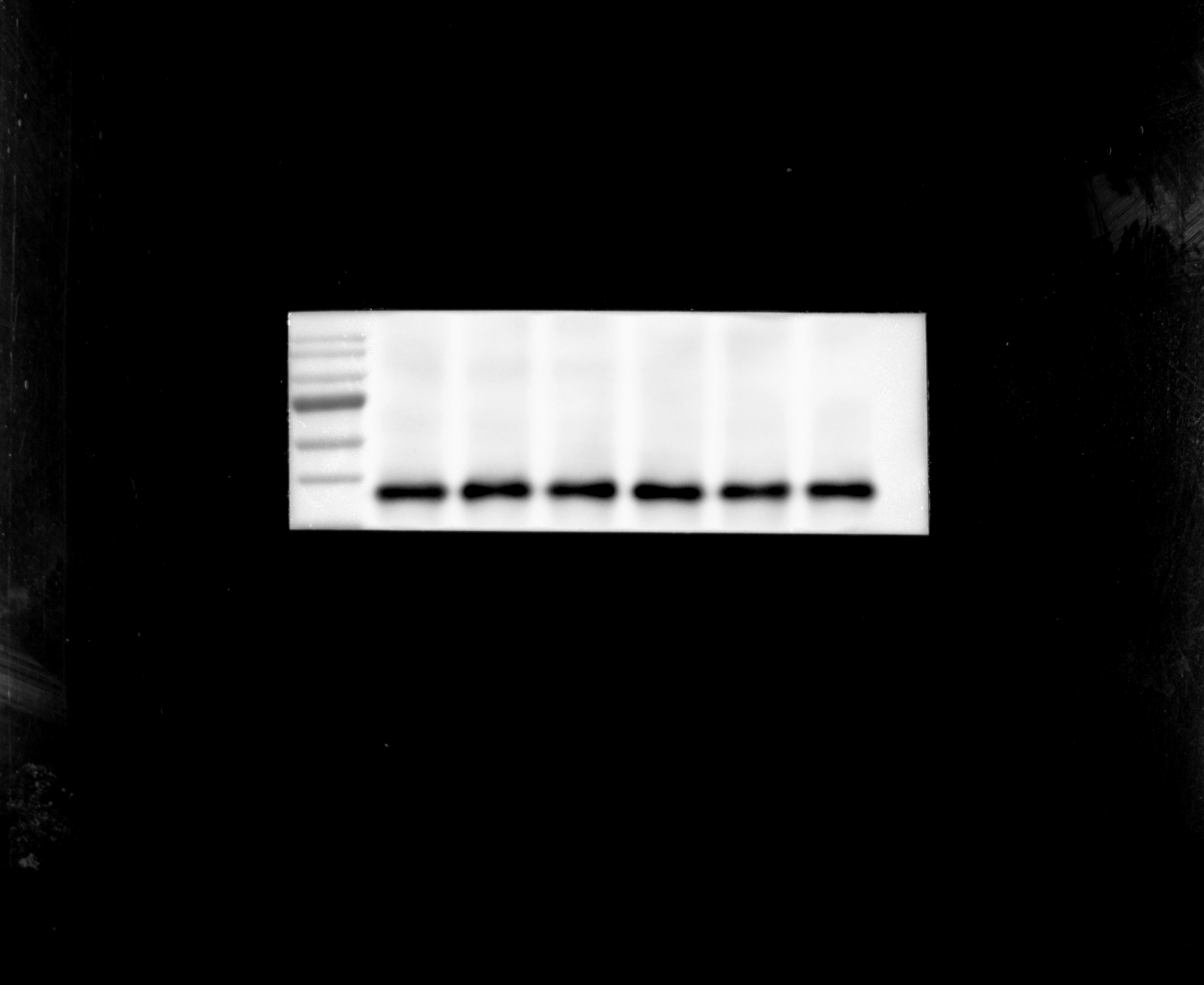

Supplement: Supplementary file 1 [file cimb-47-00936-s001.zip › cimb-3956315-supplementary/APOC2_ccRCC_RawWB_FullMembranes/cropped display images/13/β- actin/2.Tif]

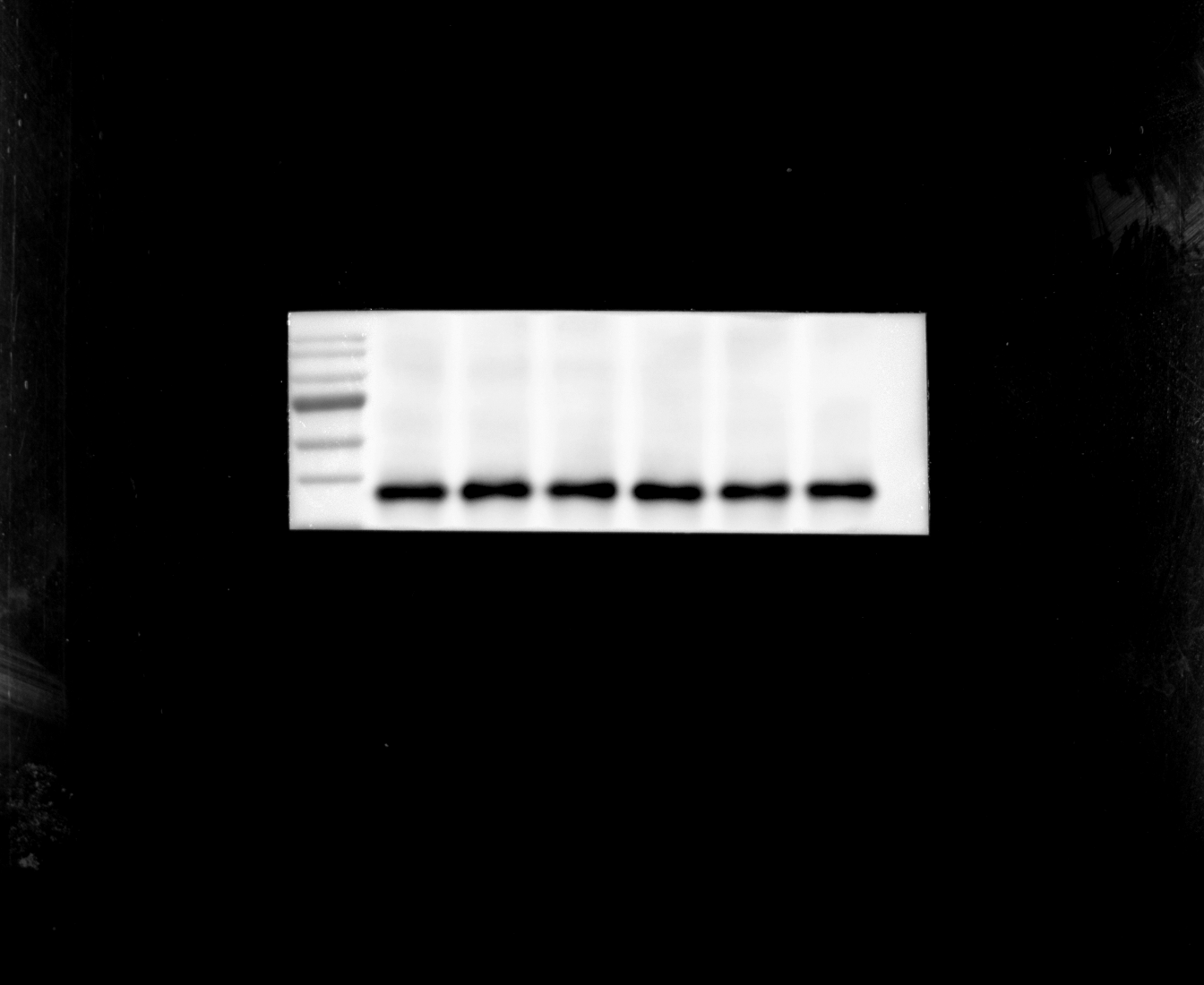

Supplement: Supplementary file 1 [file cimb-47-00936-s001.zip › cimb-3956315-supplementary/APOC2_ccRCC_RawWB_FullMembranes/cropped display images/13/β- actin/3.Tif]

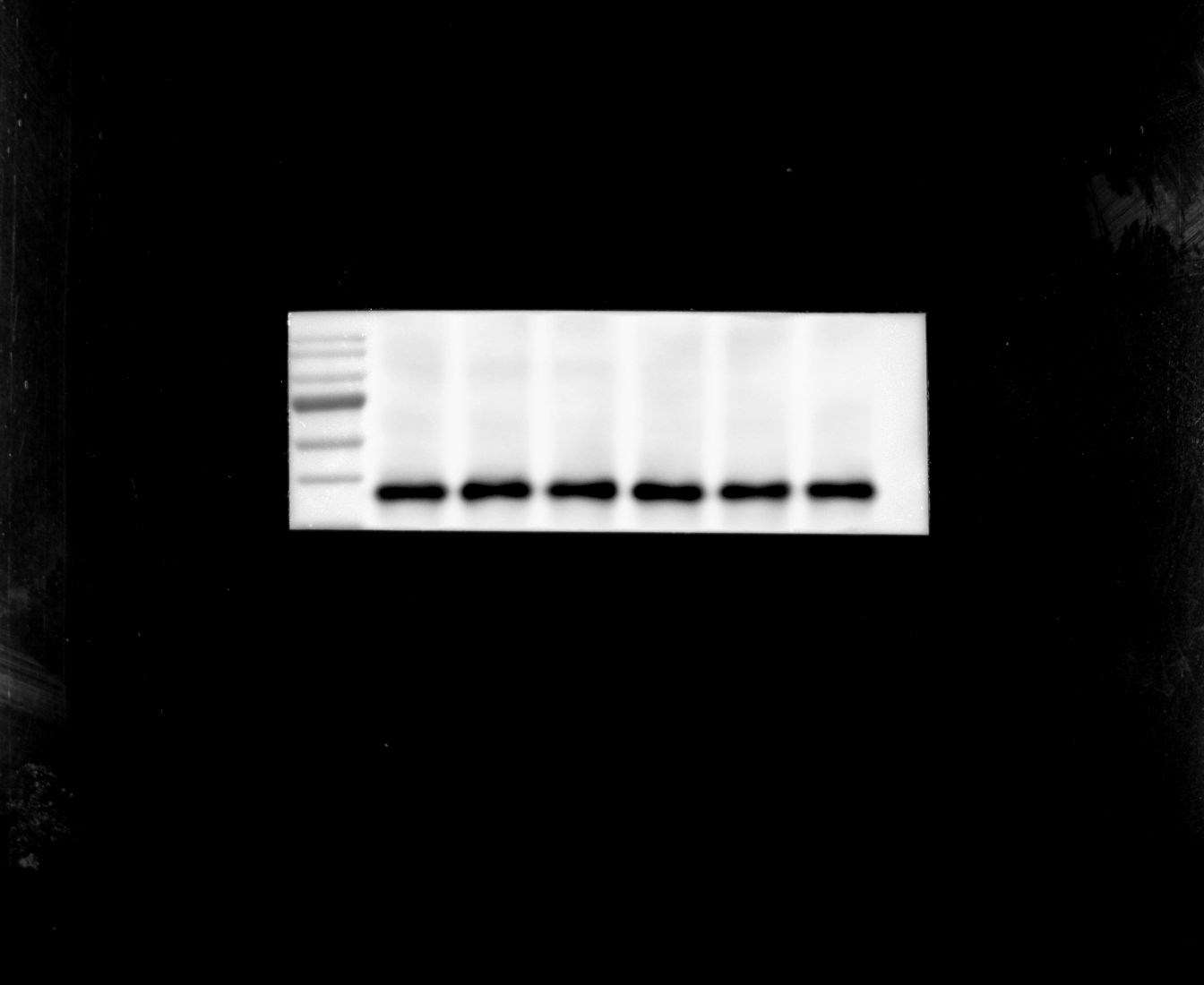

Supplement: Supplementary file 1 [file cimb-47-00936-s001.zip › cimb-3956315-supplementary/APOC2_ccRCC_RawWB_FullMembranes/cropped display images/13/β- actin/4.Tif]

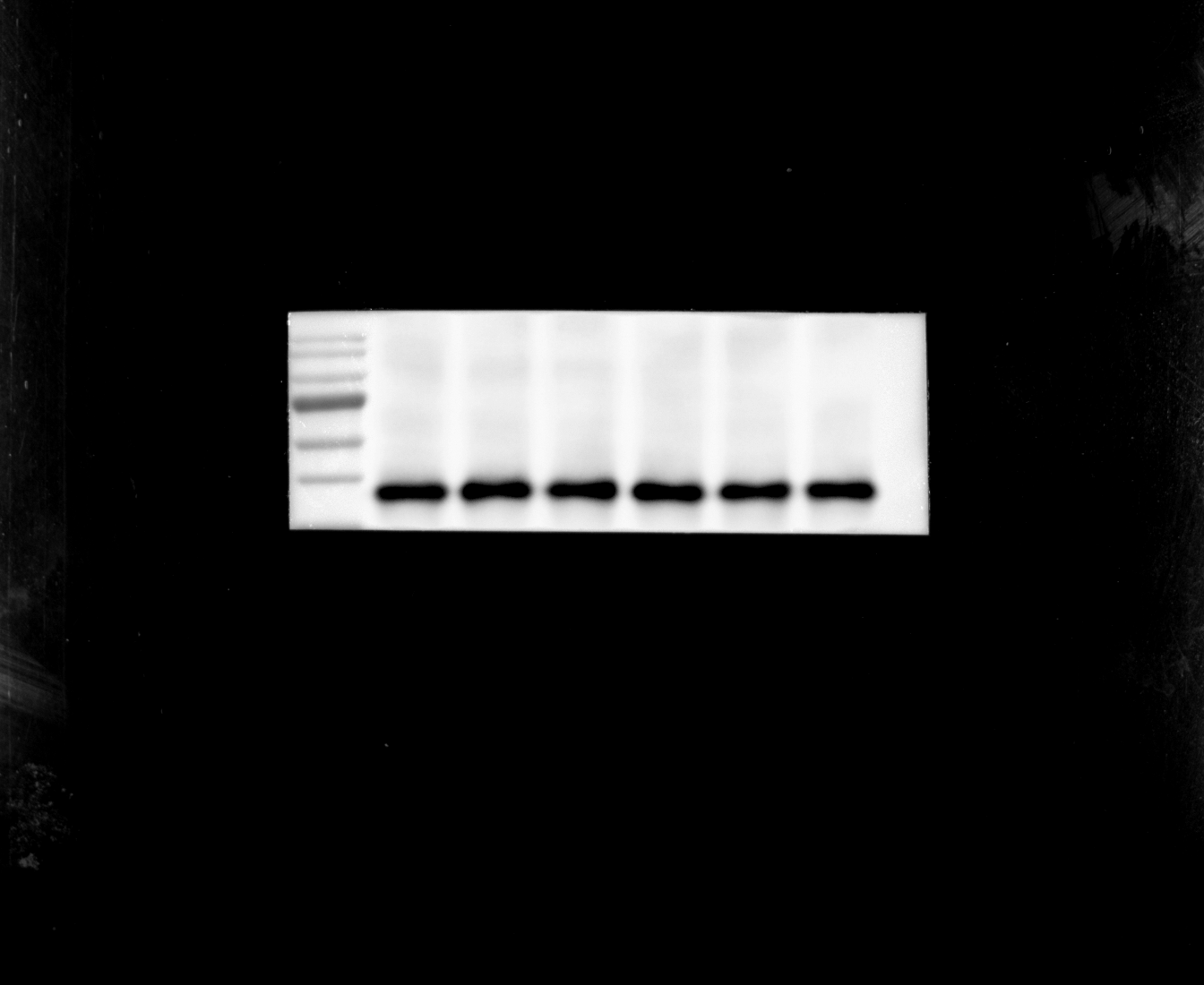

Supplement: Supplementary file 1 [file cimb-47-00936-s001.zip › cimb-3956315-supplementary/APOC2_ccRCC_RawWB_FullMembranes/cropped display images/13/β- actin/5.Tif]

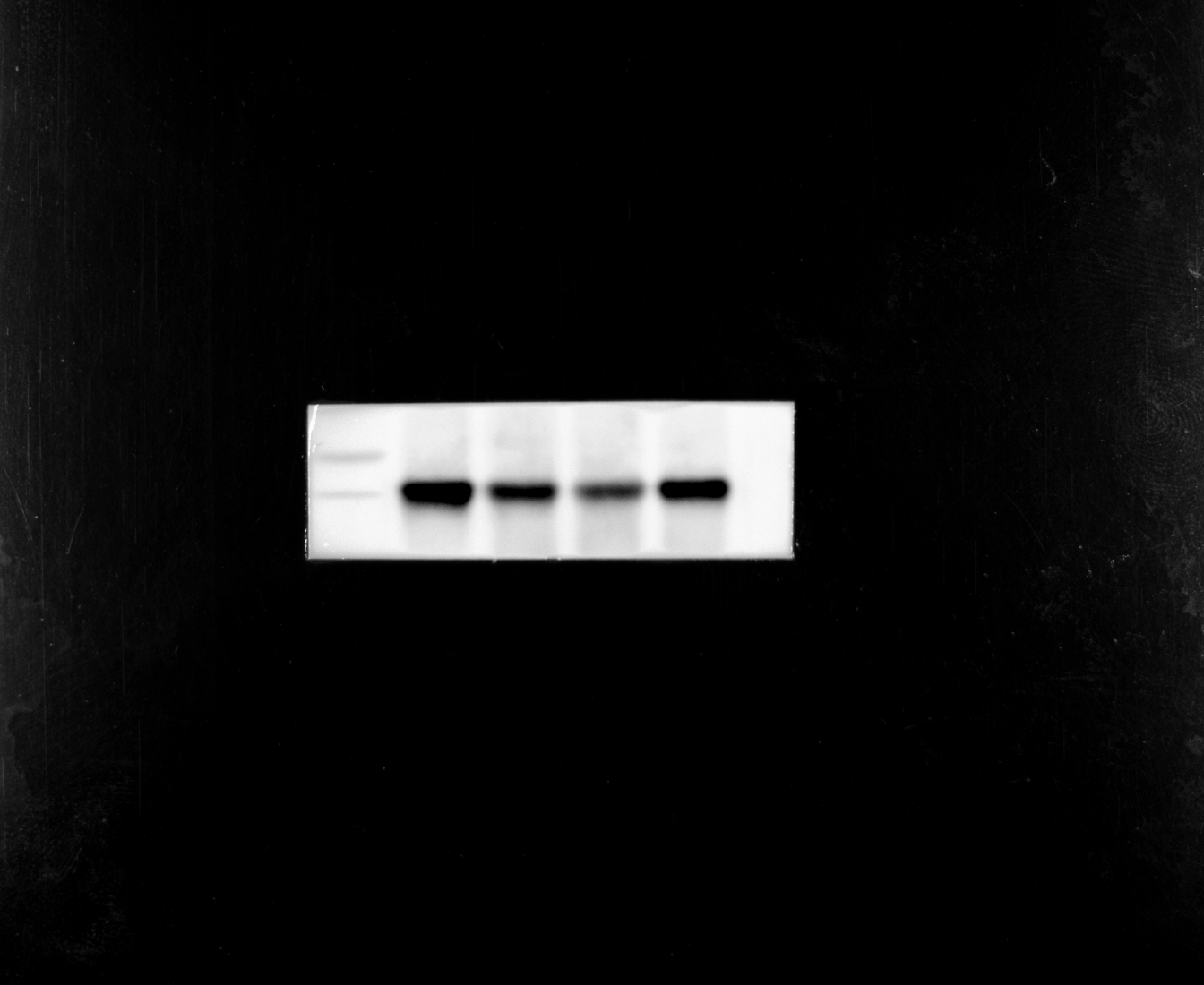

Supplement: Supplementary file 1 [file cimb-47-00936-s001.zip › cimb-3956315-supplementary/APOC2_ccRCC_RawWB_FullMembranes/cropped display images/2/Fig 2F APOC2/0.Tif]

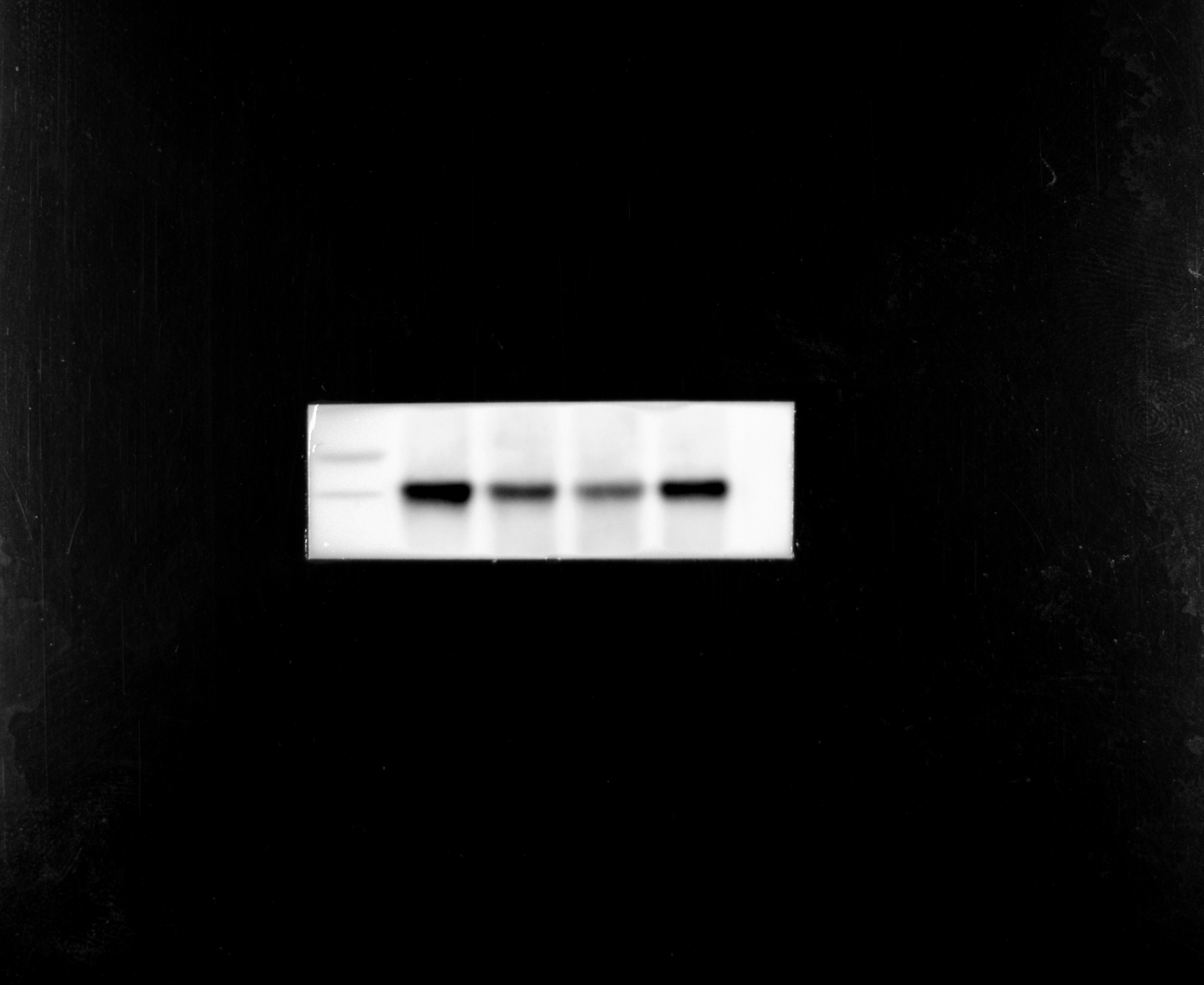

Supplement: Supplementary file 1 [file cimb-47-00936-s001.zip › cimb-3956315-supplementary/APOC2_ccRCC_RawWB_FullMembranes/cropped display images/2/Fig 2F APOC2/1.Tif]

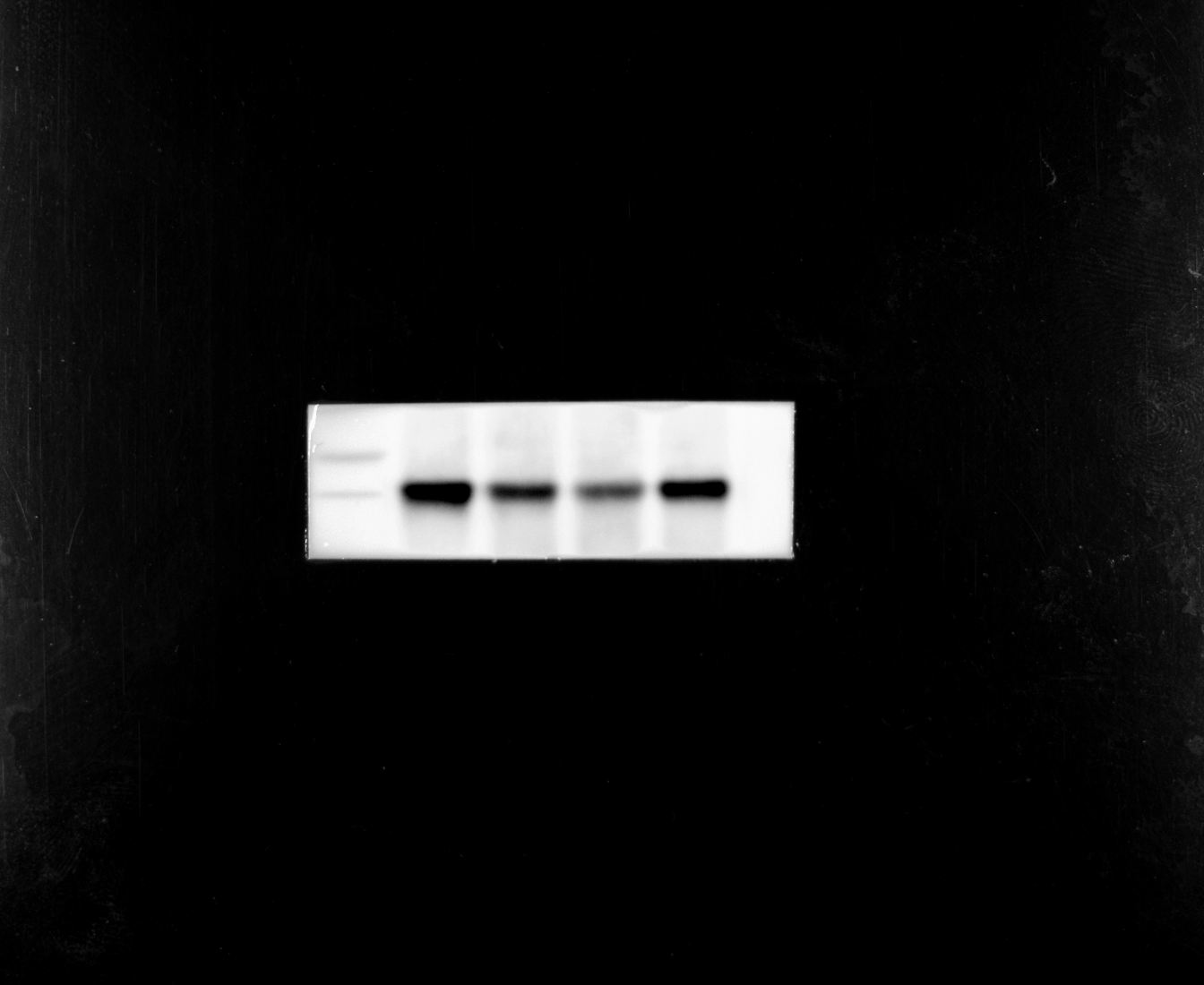

Supplement: Supplementary file 1 [file cimb-47-00936-s001.zip › cimb-3956315-supplementary/APOC2_ccRCC_RawWB_FullMembranes/cropped display images/2/Fig 2F APOC2/2.Tif]

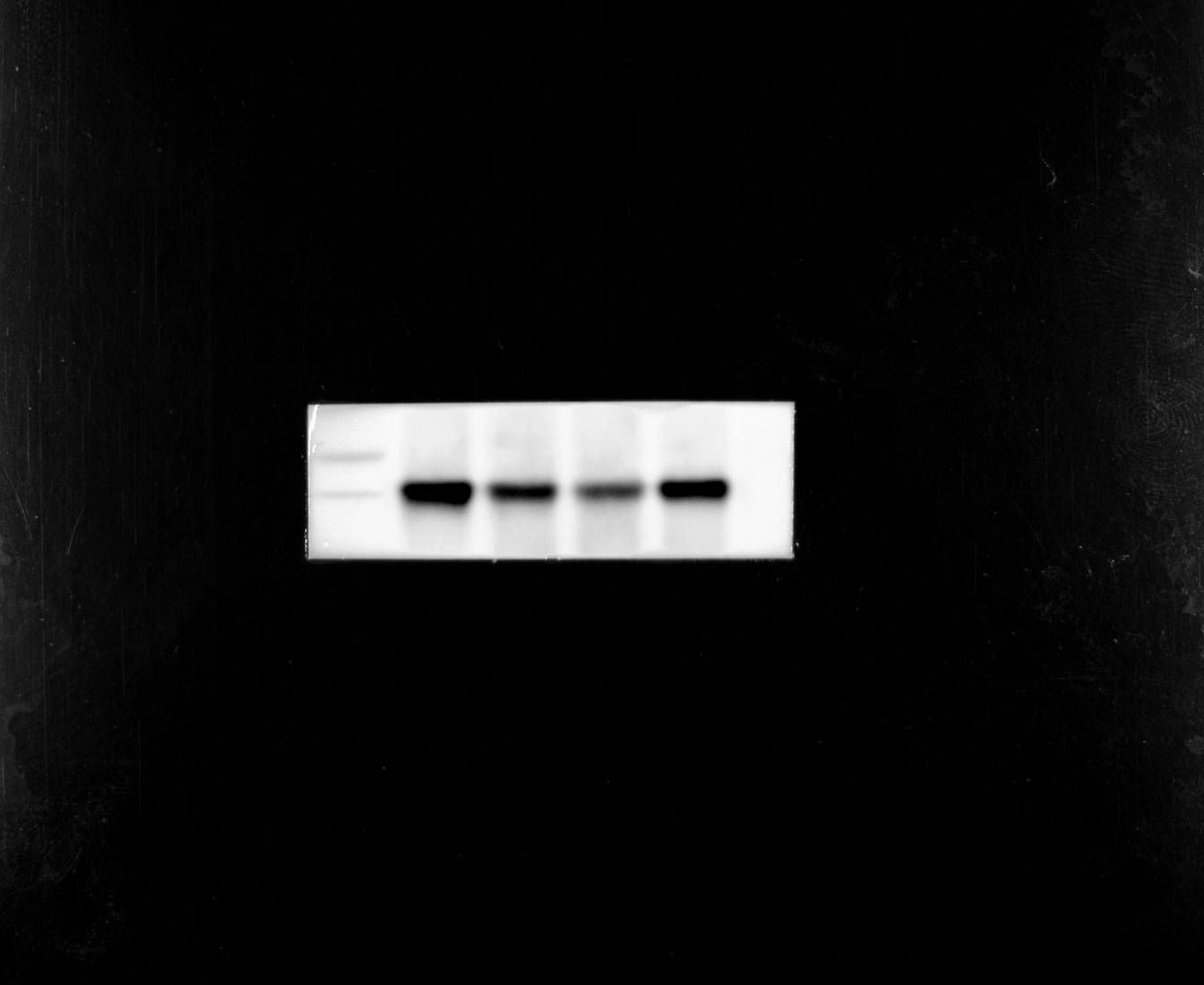

Supplement: Supplementary file 1 [file cimb-47-00936-s001.zip › cimb-3956315-supplementary/APOC2_ccRCC_RawWB_FullMembranes/cropped display images/2/Fig 2F APOC2/3.Tif]

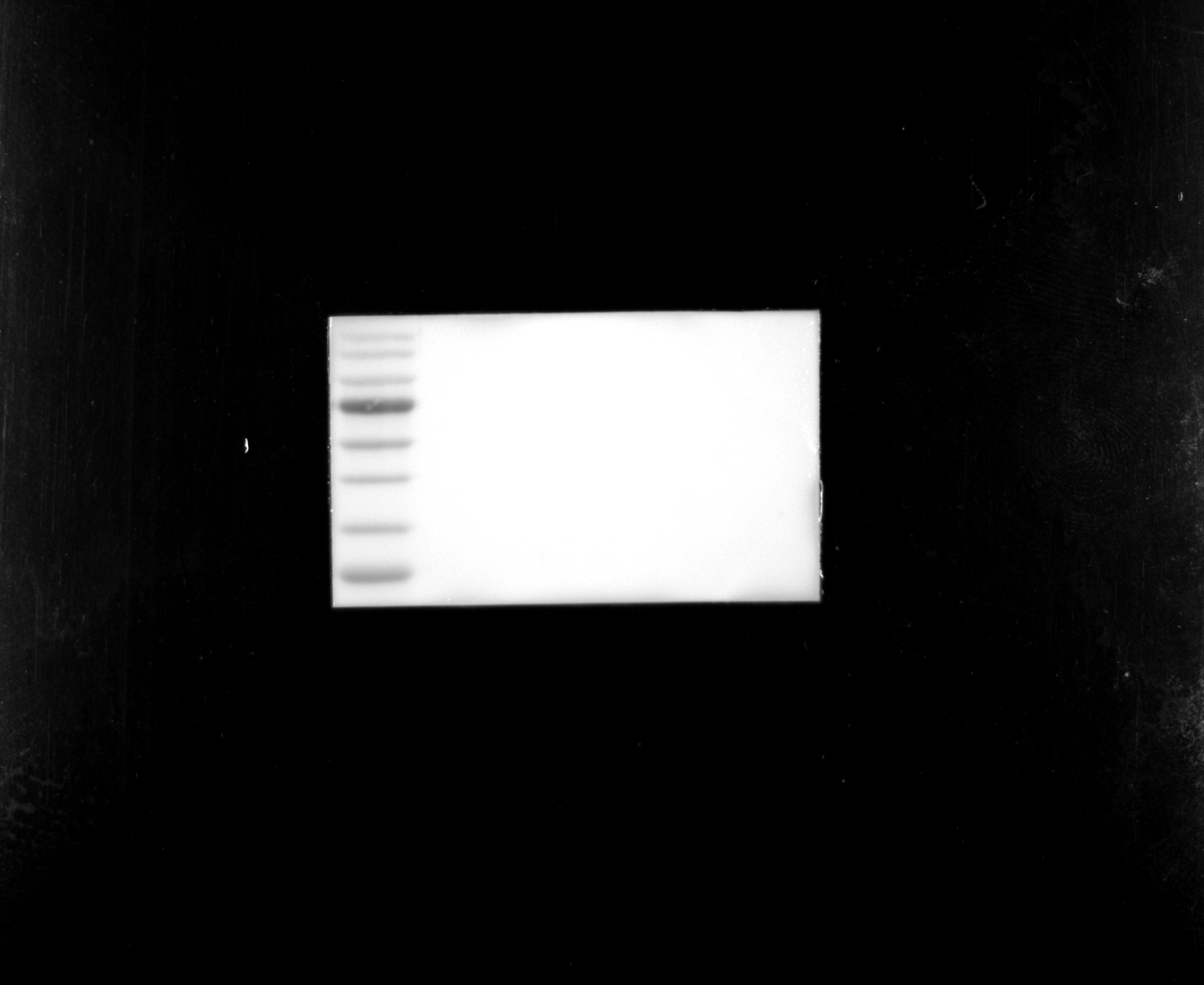

Supplement: Supplementary file 1 [file cimb-47-00936-s001.zip › cimb-3956315-supplementary/APOC2_ccRCC_RawWB_FullMembranes/cropped display images/2/Fig 2F GAPDH/0.Tif]

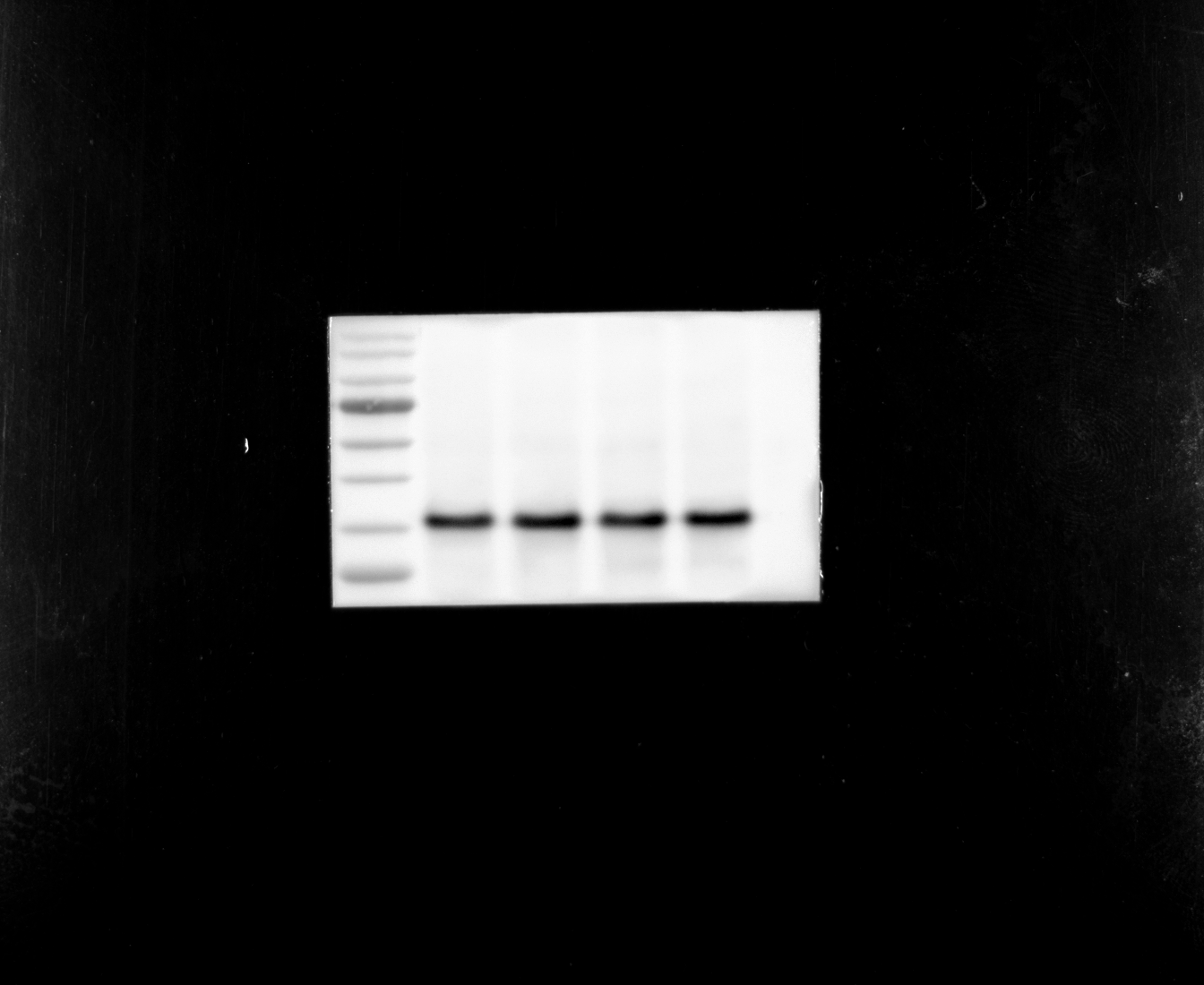

Supplement: Supplementary file 1 [file cimb-47-00936-s001.zip › cimb-3956315-supplementary/APOC2_ccRCC_RawWB_FullMembranes/cropped display images/2/Fig 2F GAPDH/1.Tif]

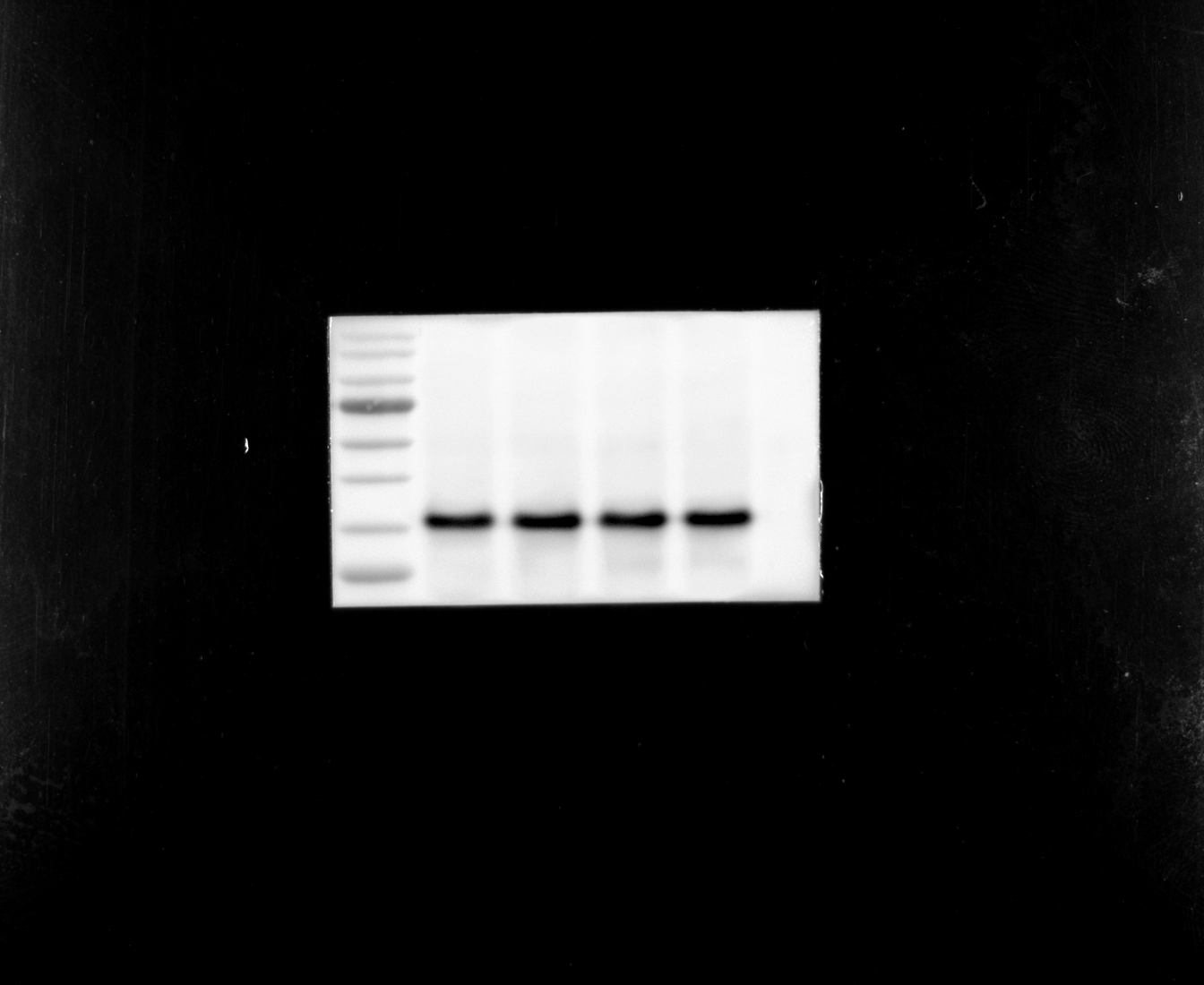

Supplement: Supplementary file 1 [file cimb-47-00936-s001.zip › cimb-3956315-supplementary/APOC2_ccRCC_RawWB_FullMembranes/cropped display images/2/Fig 2F GAPDH/2.Tif]

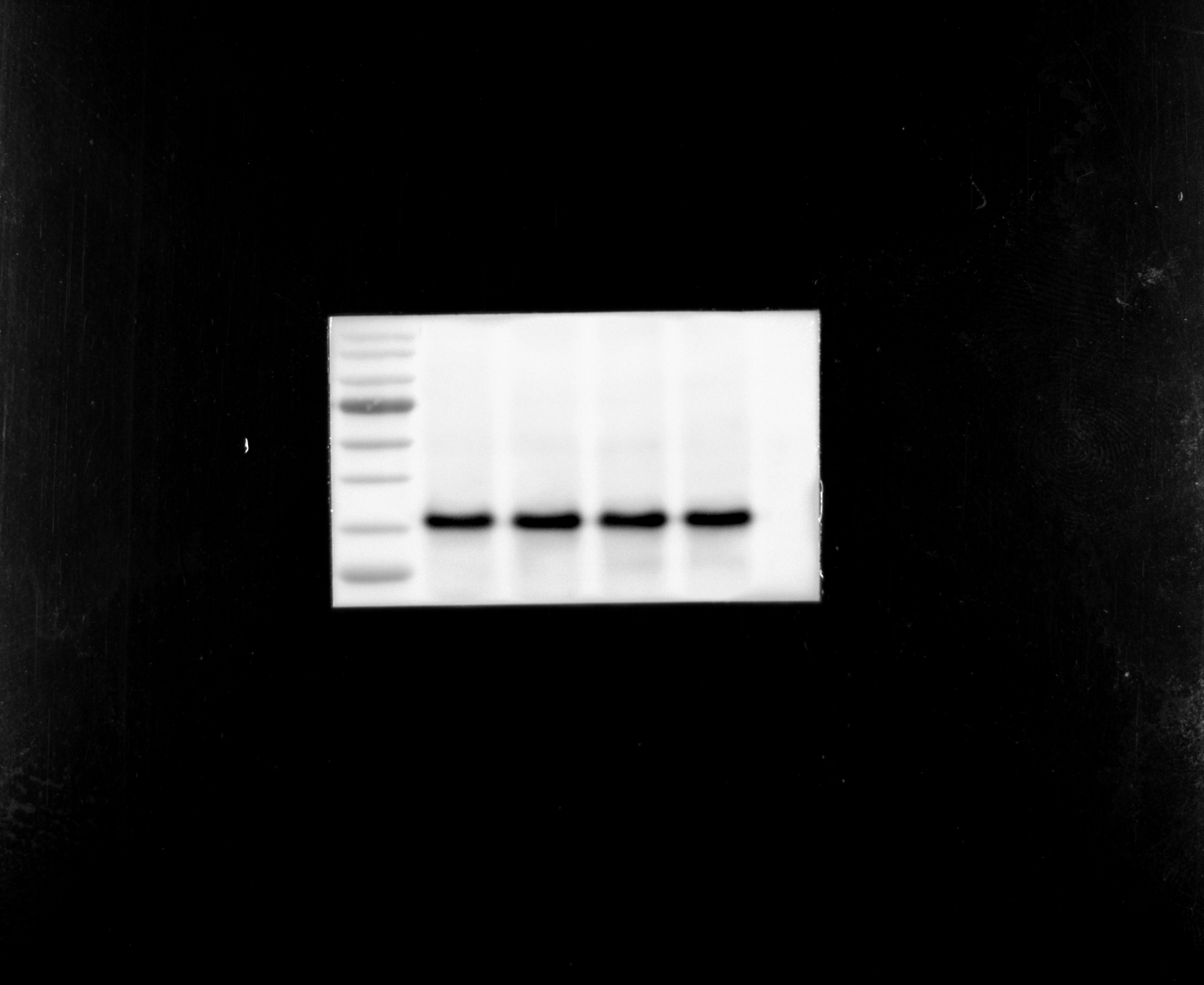

Supplement: Supplementary file 1 [file cimb-47-00936-s001.zip › cimb-3956315-supplementary/APOC2_ccRCC_RawWB_FullMembranes/cropped display images/2/Fig 2F GAPDH/3.Tif]

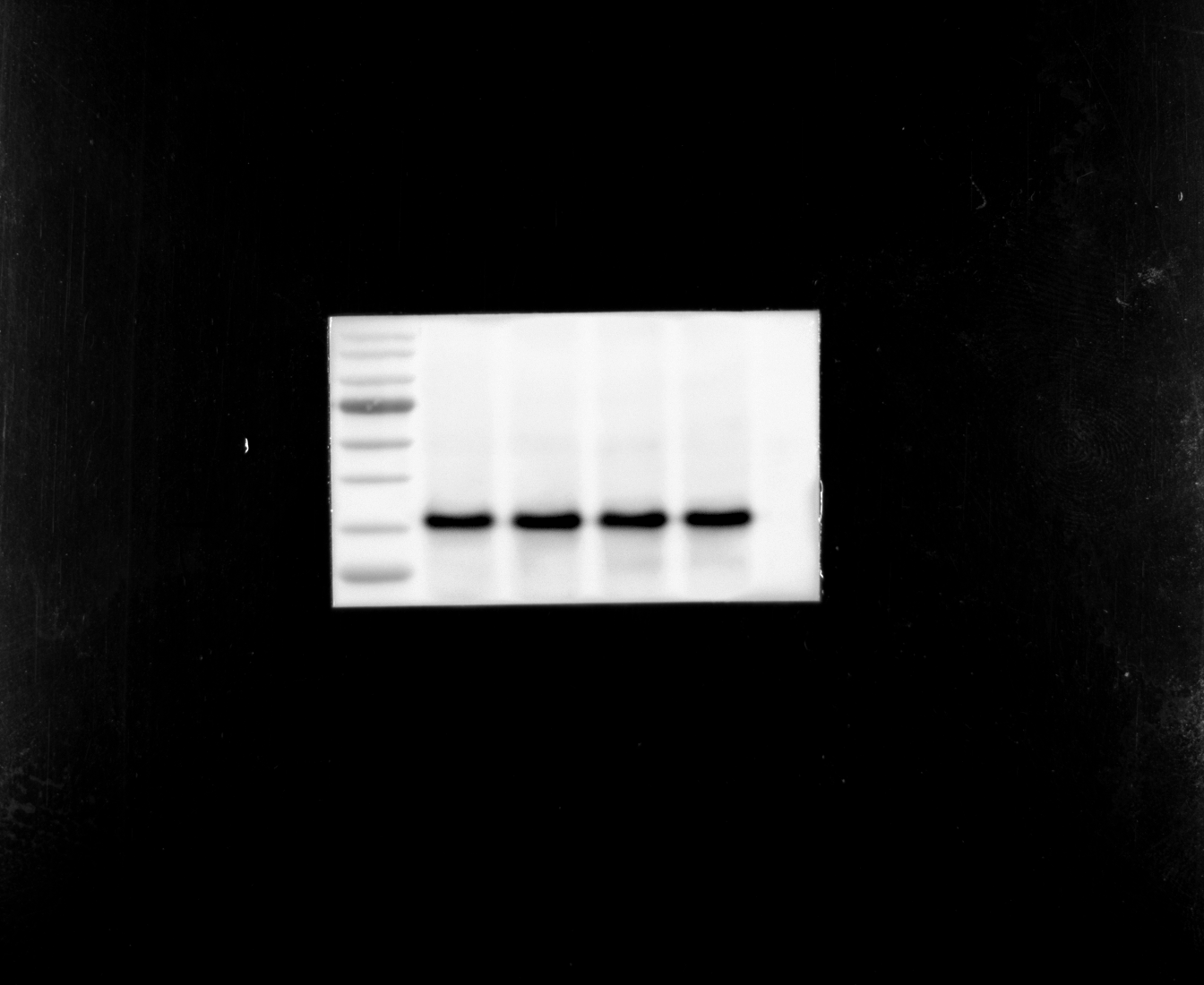

Supplement: Supplementary file 1 [file cimb-47-00936-s001.zip › cimb-3956315-supplementary/APOC2_ccRCC_RawWB_FullMembranes/cropped display images/2/Fig 2F GAPDH/4.Tif]

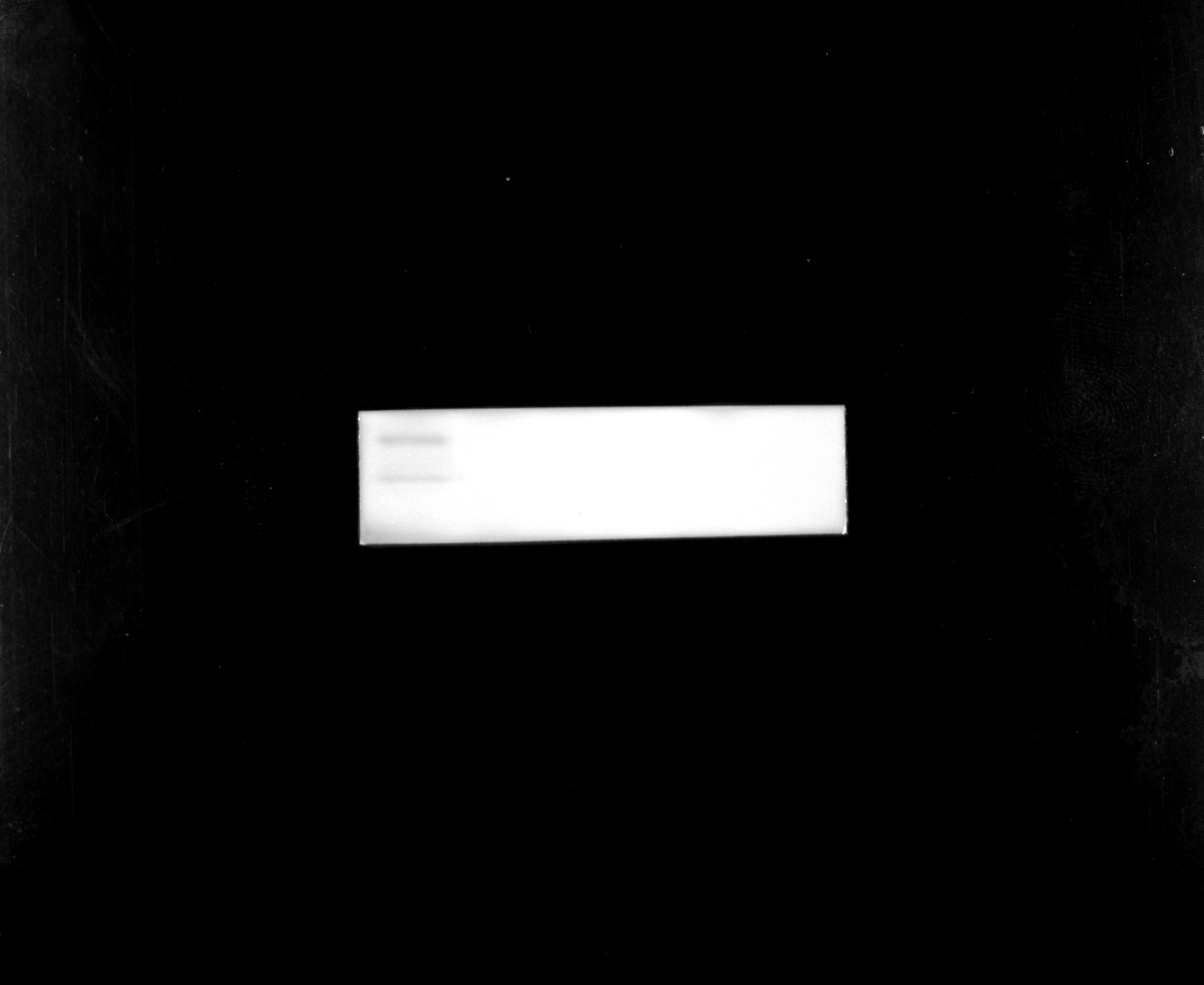

Supplement: Supplementary file 1 [file cimb-47-00936-s001.zip › cimb-3956315-supplementary/APOC2_ccRCC_RawWB_FullMembranes/cropped display images/3/Fig 3C APOC2/0.Tif]

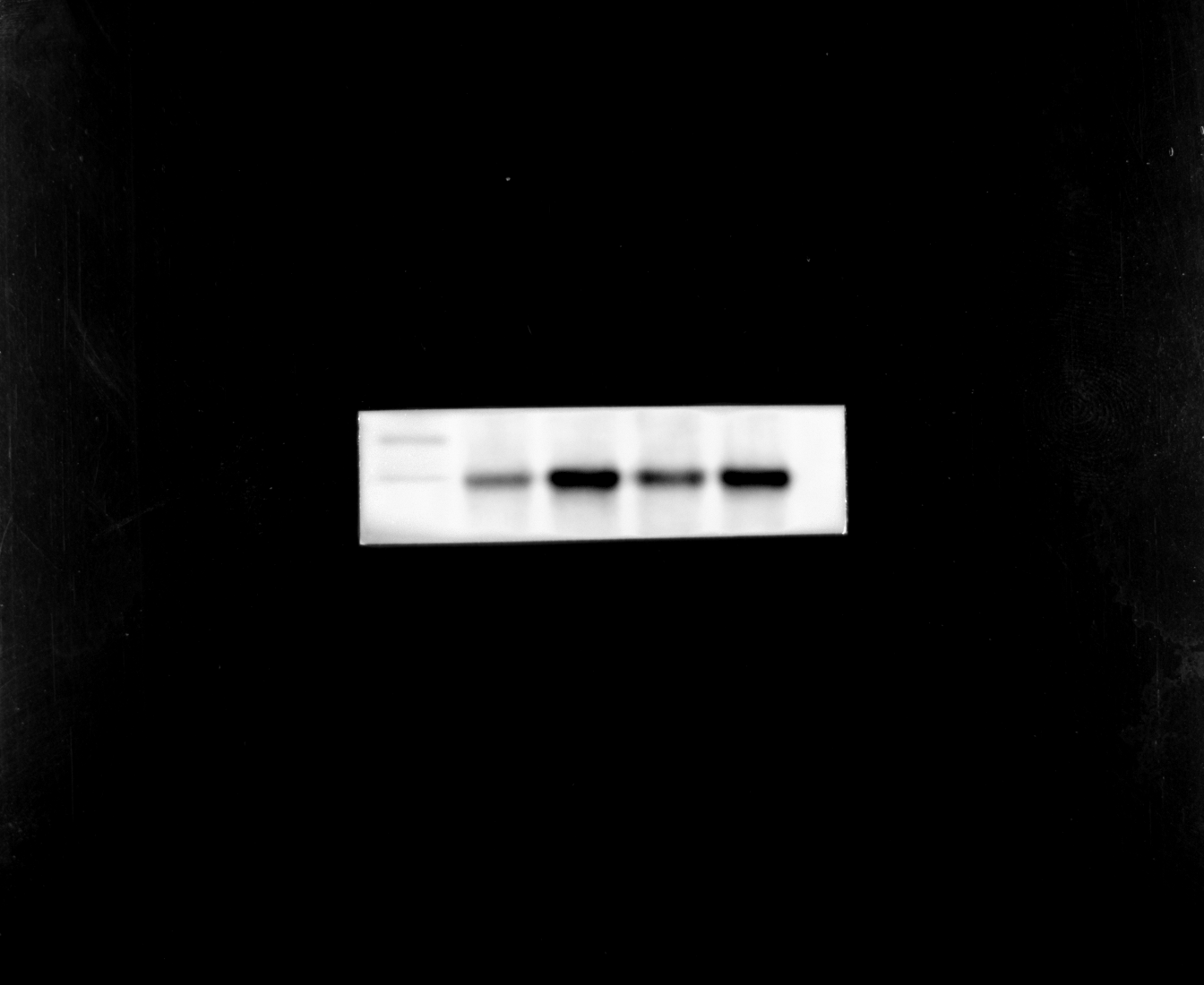

Supplement: Supplementary file 1 [file cimb-47-00936-s001.zip › cimb-3956315-supplementary/APOC2_ccRCC_RawWB_FullMembranes/cropped display images/3/Fig 3C APOC2/1.Tif]

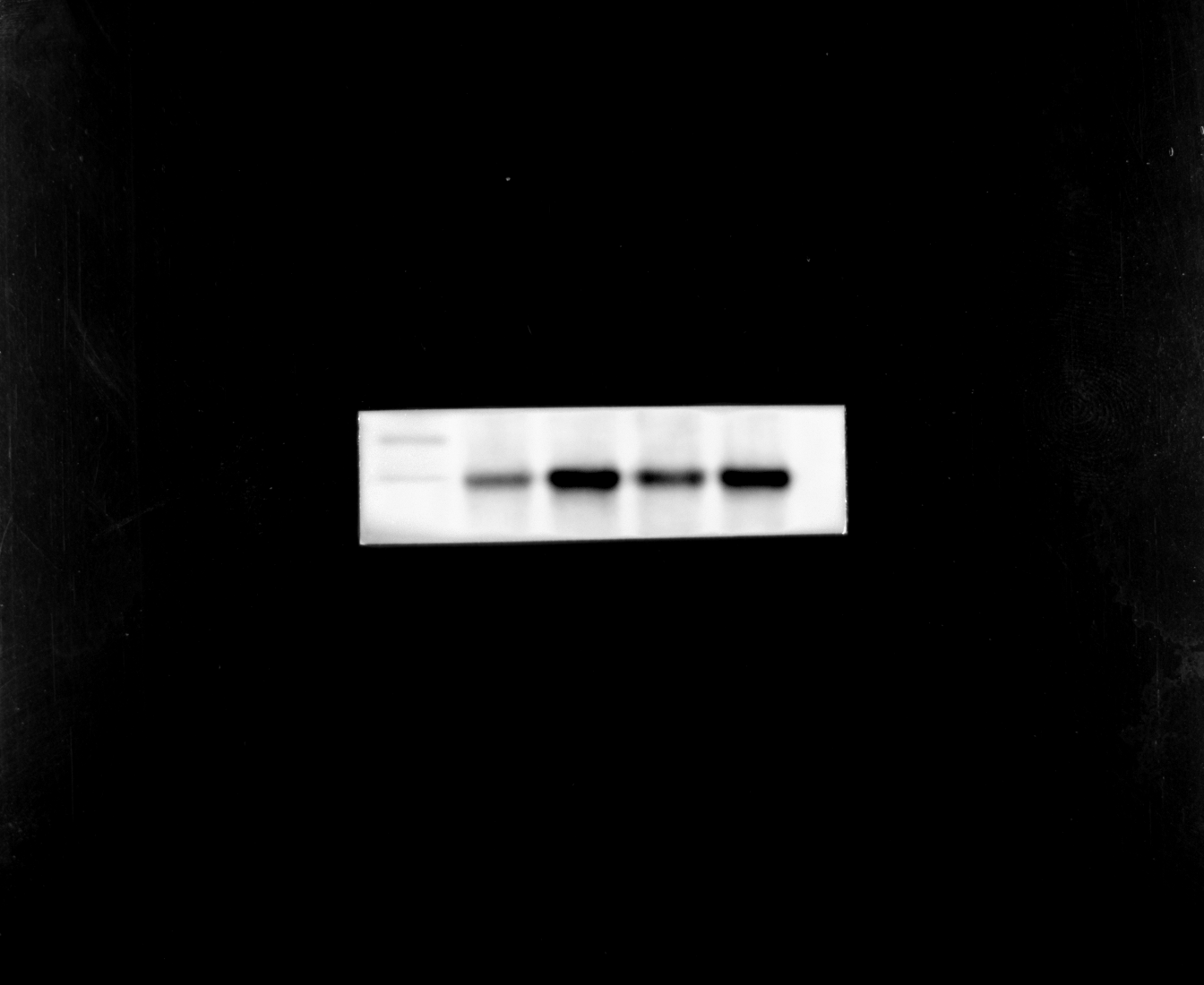

Supplement: Supplementary file 1 [file cimb-47-00936-s001.zip › cimb-3956315-supplementary/APOC2_ccRCC_RawWB_FullMembranes/cropped display images/3/Fig 3C APOC2/2.Tif]

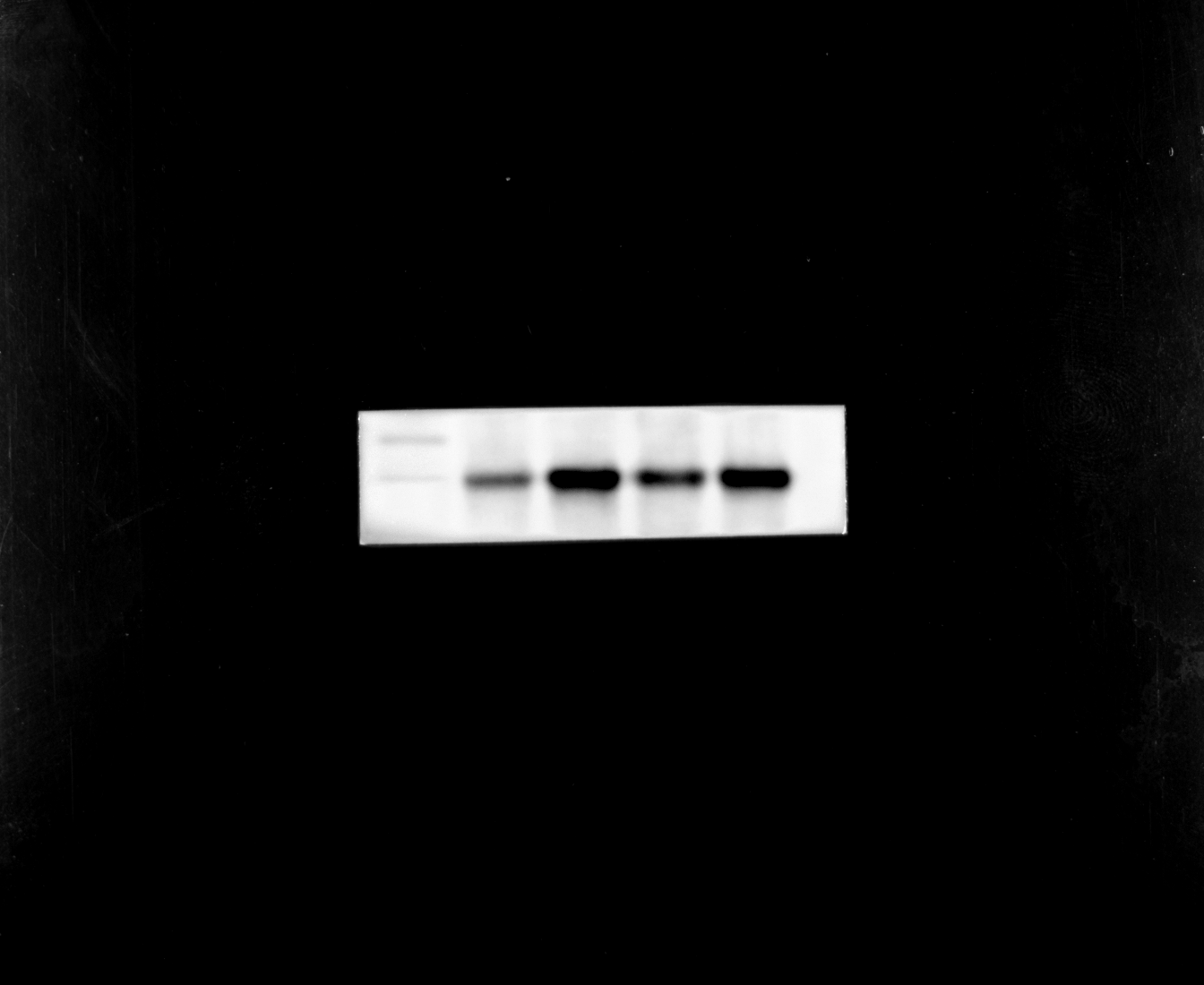

Supplement: Supplementary file 1 [file cimb-47-00936-s001.zip › cimb-3956315-supplementary/APOC2_ccRCC_RawWB_FullMembranes/cropped display images/3/Fig 3C APOC2/3.Tif]

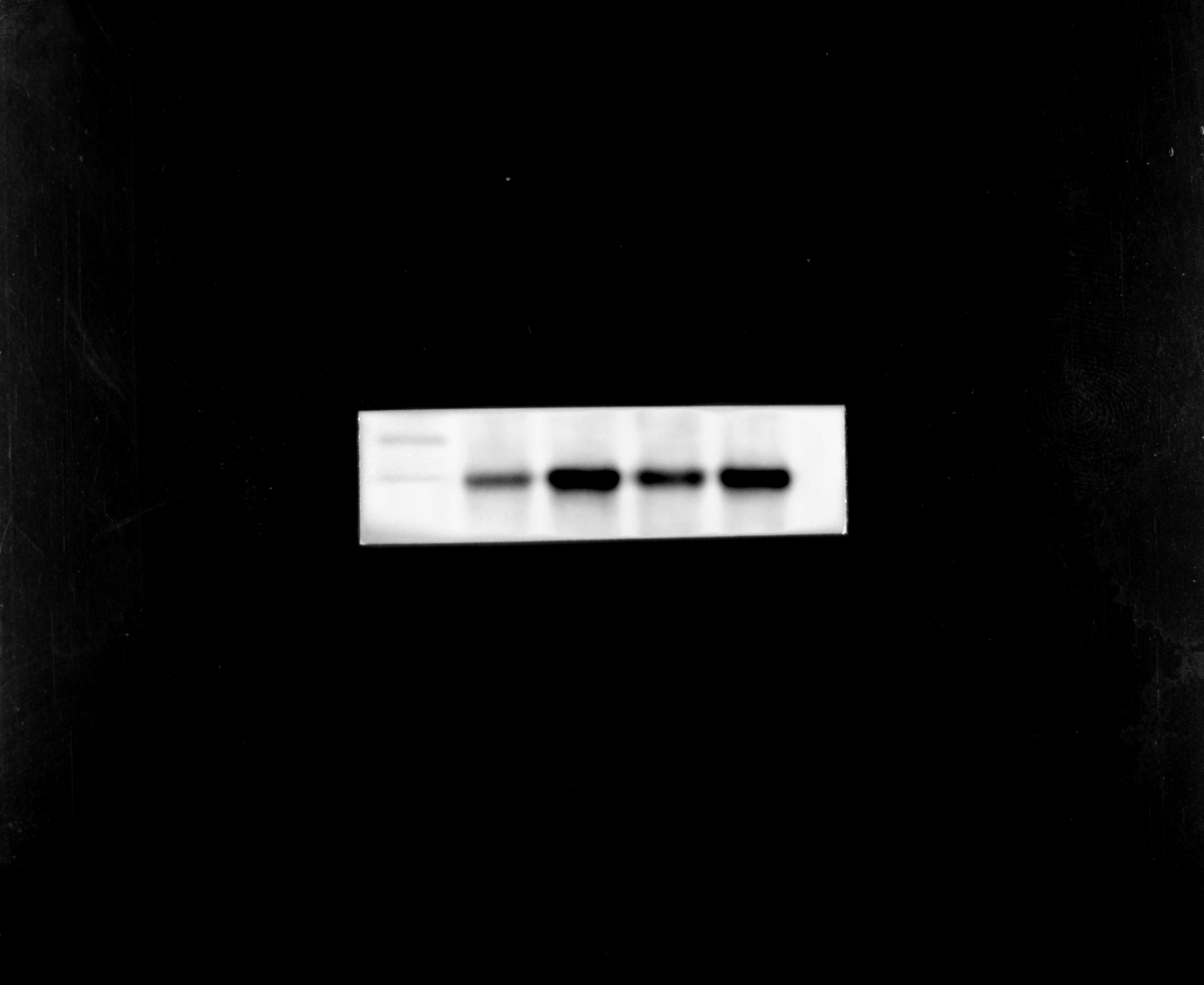

Supplement: Supplementary file 1 [file cimb-47-00936-s001.zip › cimb-3956315-supplementary/APOC2_ccRCC_RawWB_FullMembranes/cropped display images/3/Fig 3C APOC2/4.Tif]

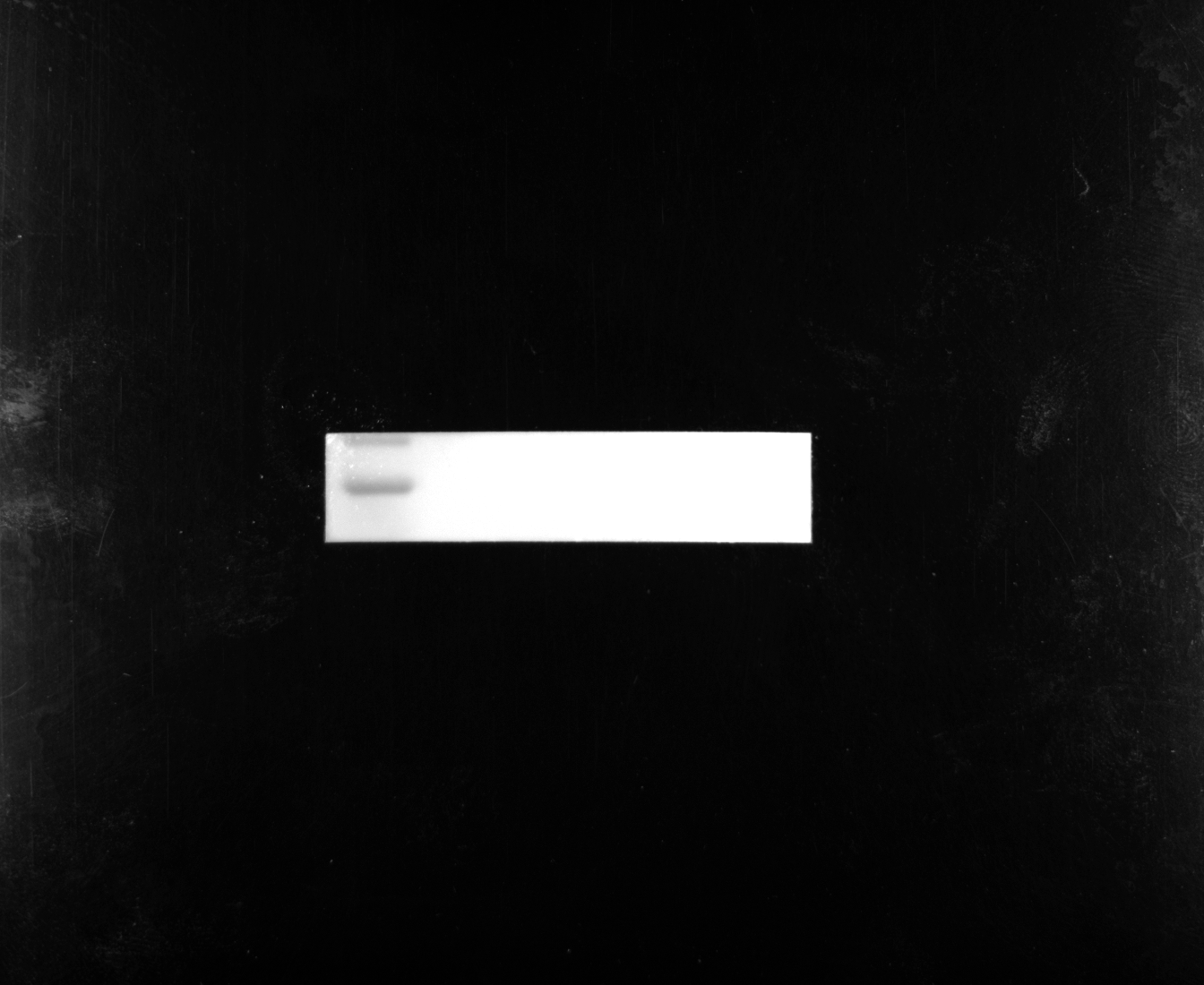

Supplement: Supplementary file 1 [file cimb-47-00936-s001.zip › cimb-3956315-supplementary/APOC2_ccRCC_RawWB_FullMembranes/cropped display images/3/Fig 3C c-caspase3/0.Tif]

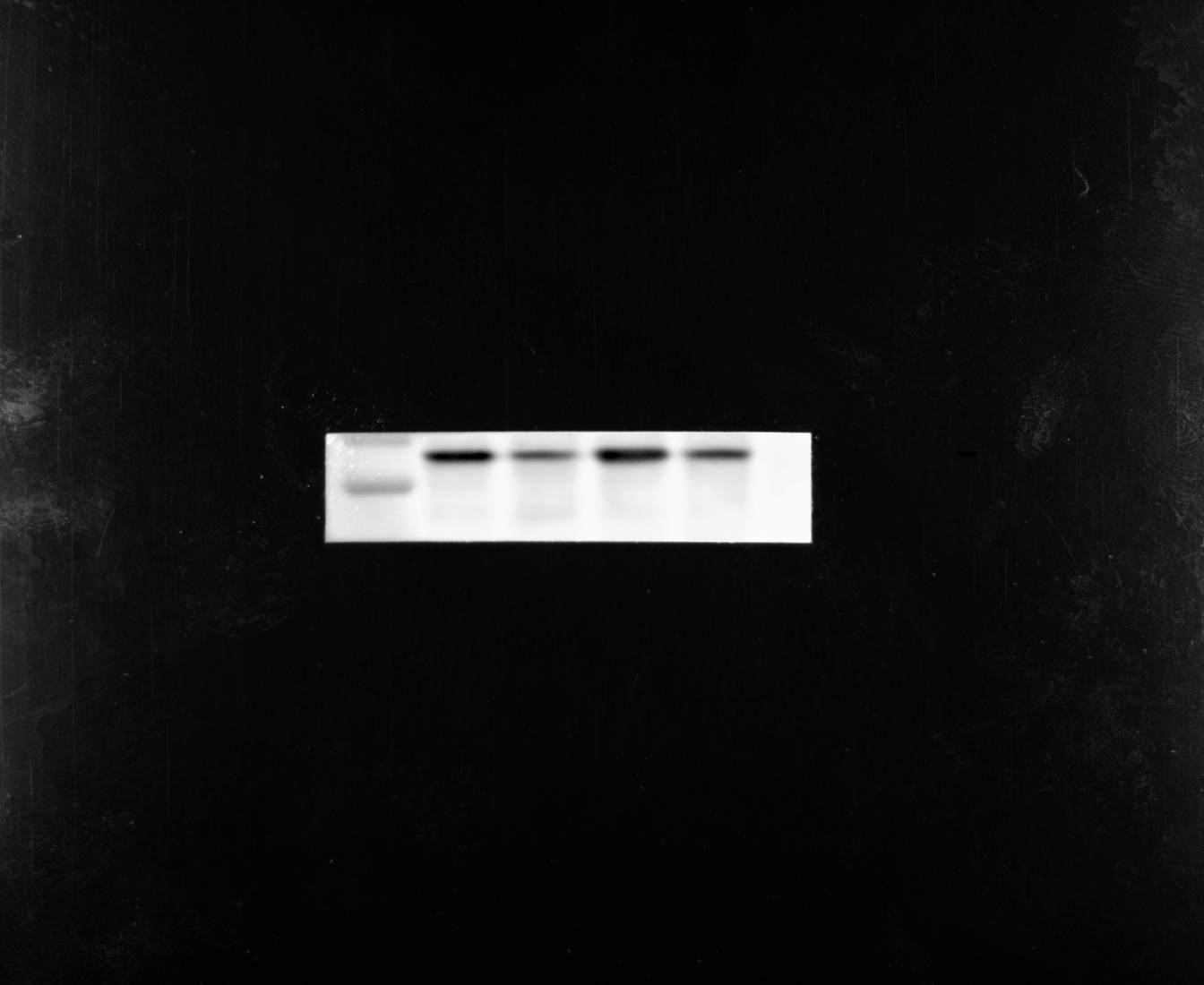

Supplement: Supplementary file 1 [file cimb-47-00936-s001.zip › cimb-3956315-supplementary/APOC2_ccRCC_RawWB_FullMembranes/cropped display images/3/Fig 3C c-caspase3/1.Tif]

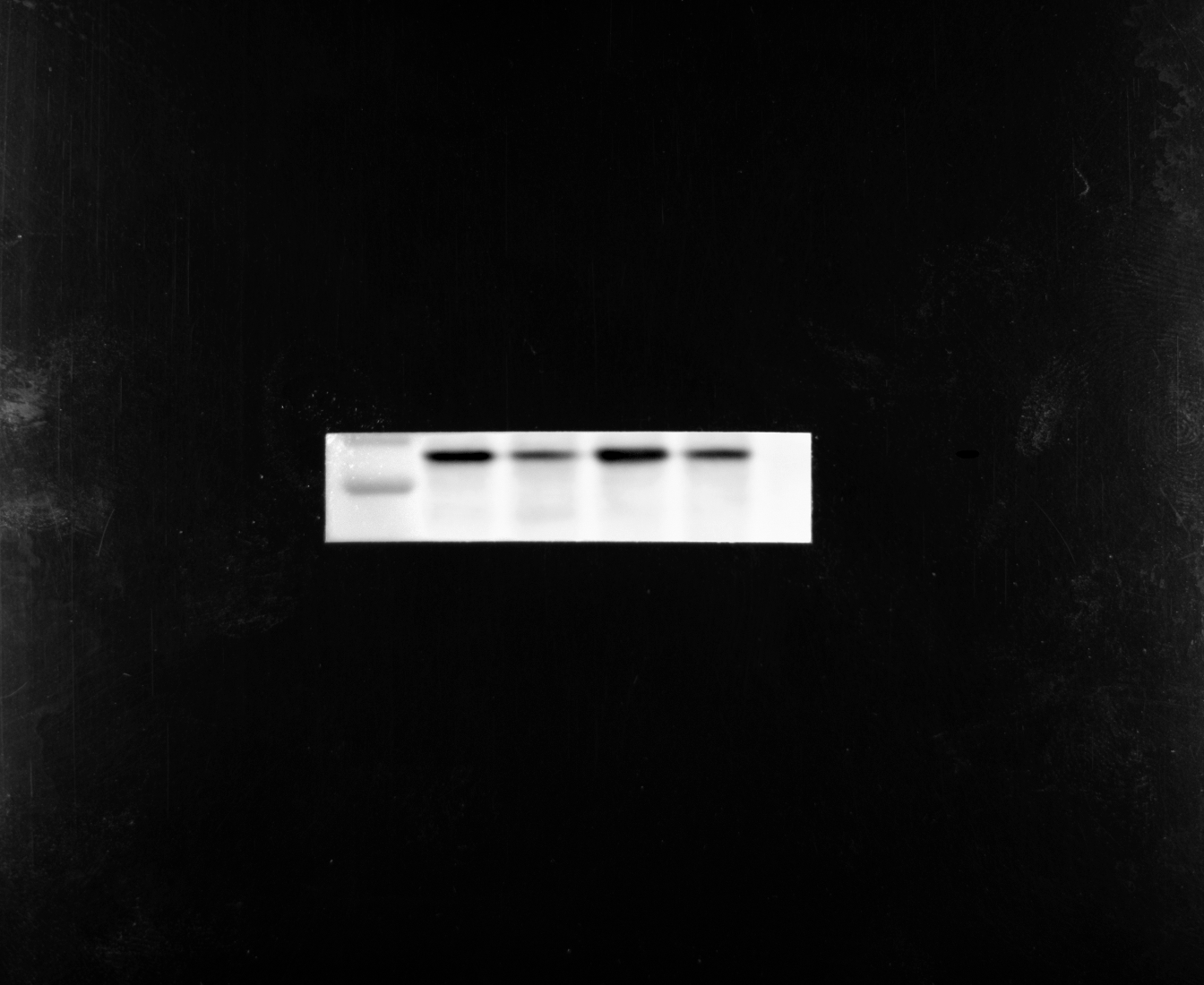

Supplement: Supplementary file 1 [file cimb-47-00936-s001.zip › cimb-3956315-supplementary/APOC2_ccRCC_RawWB_FullMembranes/cropped display images/3/Fig 3C c-caspase3/2.Tif]

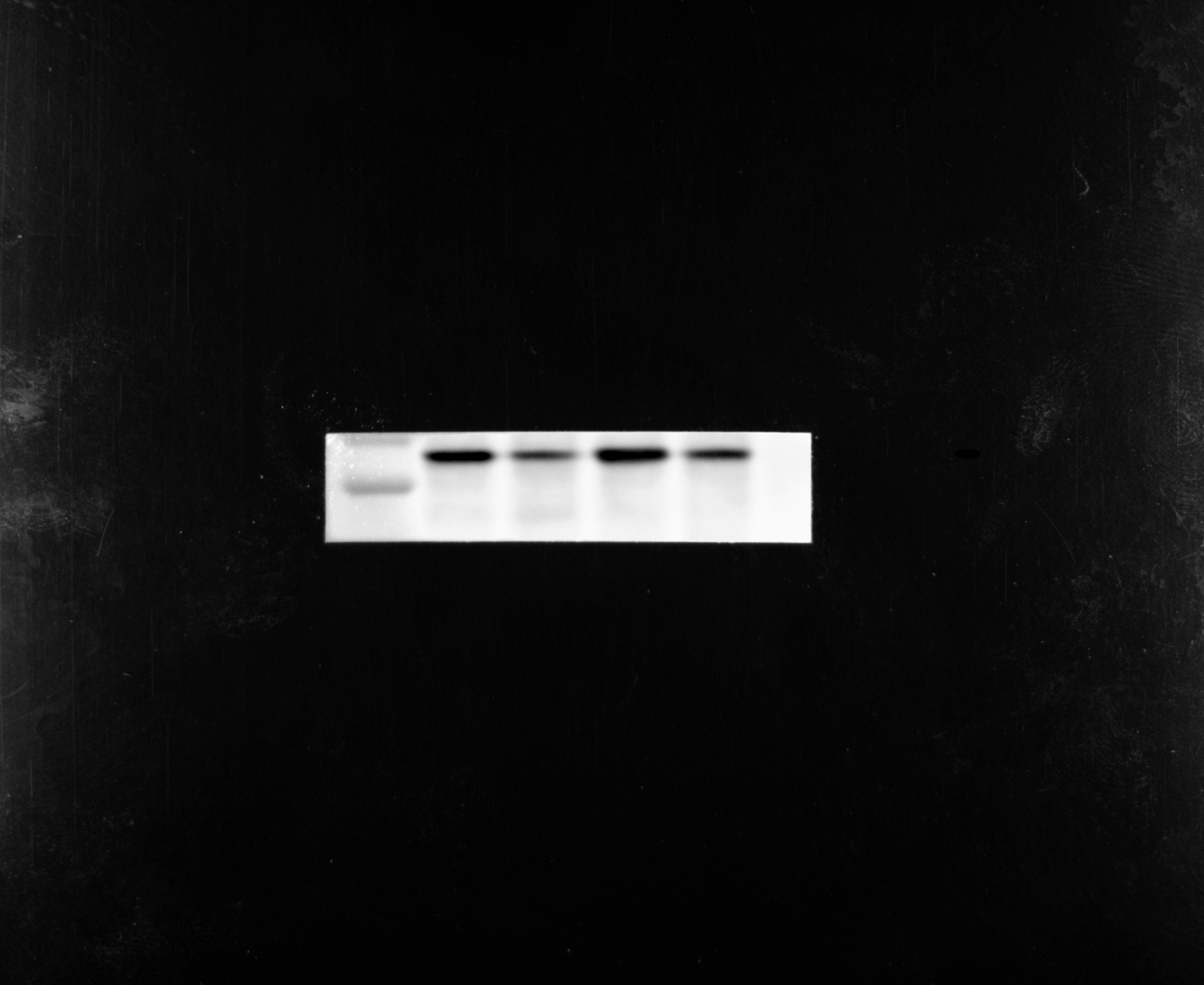

Supplement: Supplementary file 1 [file cimb-47-00936-s001.zip › cimb-3956315-supplementary/APOC2_ccRCC_RawWB_FullMembranes/cropped display images/3/Fig 3C c-caspase3/3.Tif]

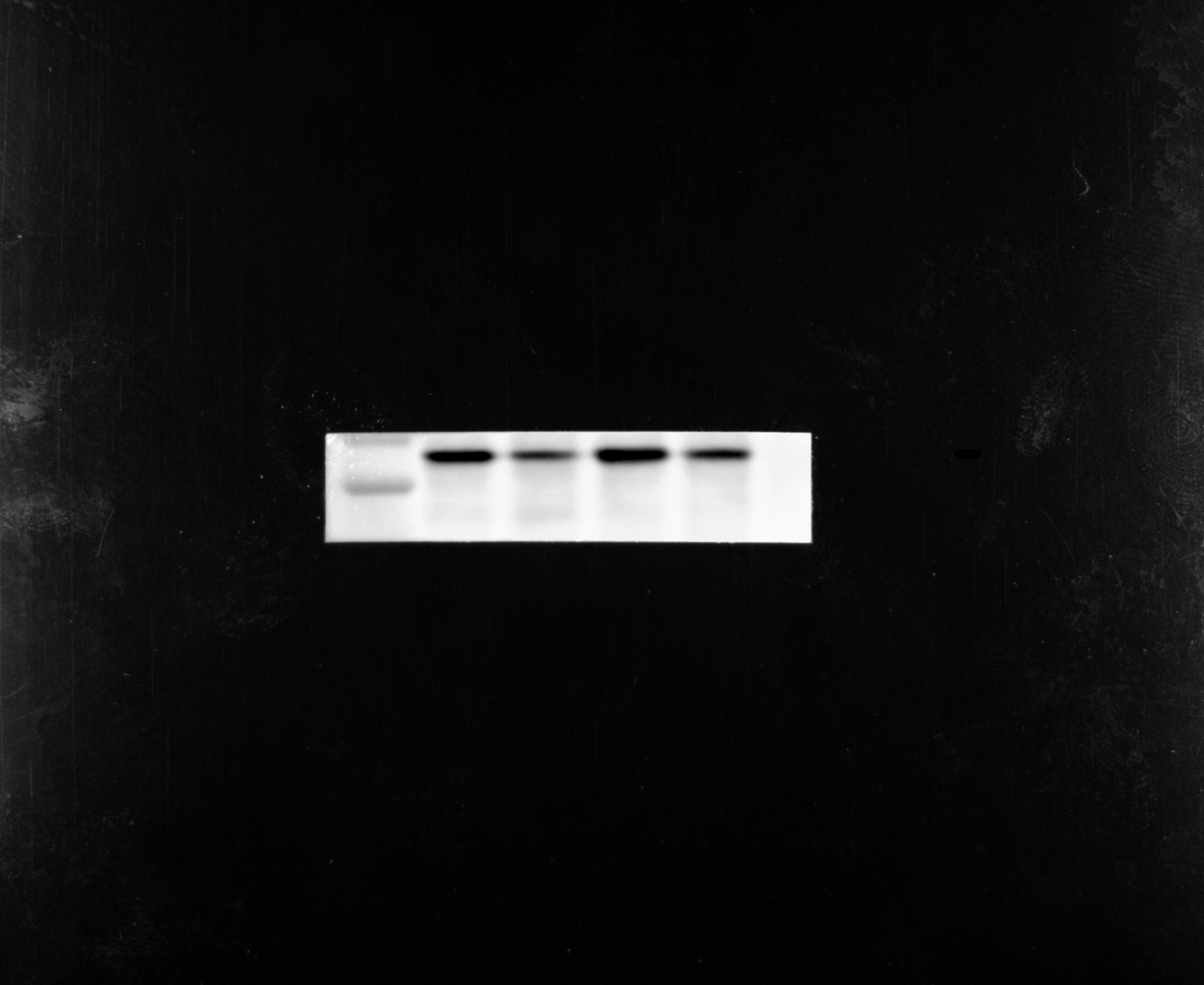

Supplement: Supplementary file 1 [file cimb-47-00936-s001.zip › cimb-3956315-supplementary/APOC2_ccRCC_RawWB_FullMembranes/cropped display images/3/Fig 3C c-caspase3/4.Tif]

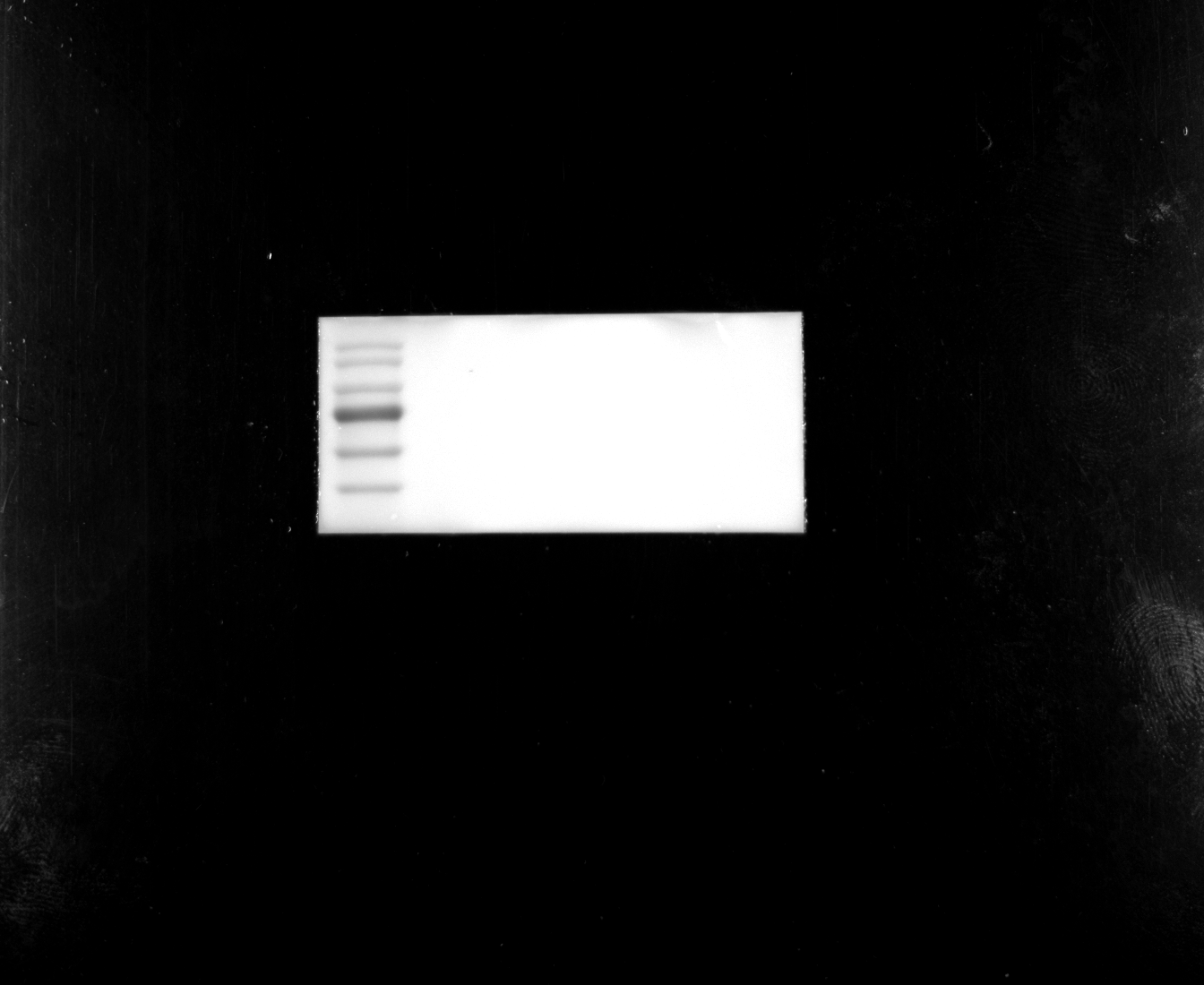

Supplement: Supplementary file 1 [file cimb-47-00936-s001.zip › cimb-3956315-supplementary/APOC2_ccRCC_RawWB_FullMembranes/cropped display images/3/β- actin/0.Tif]

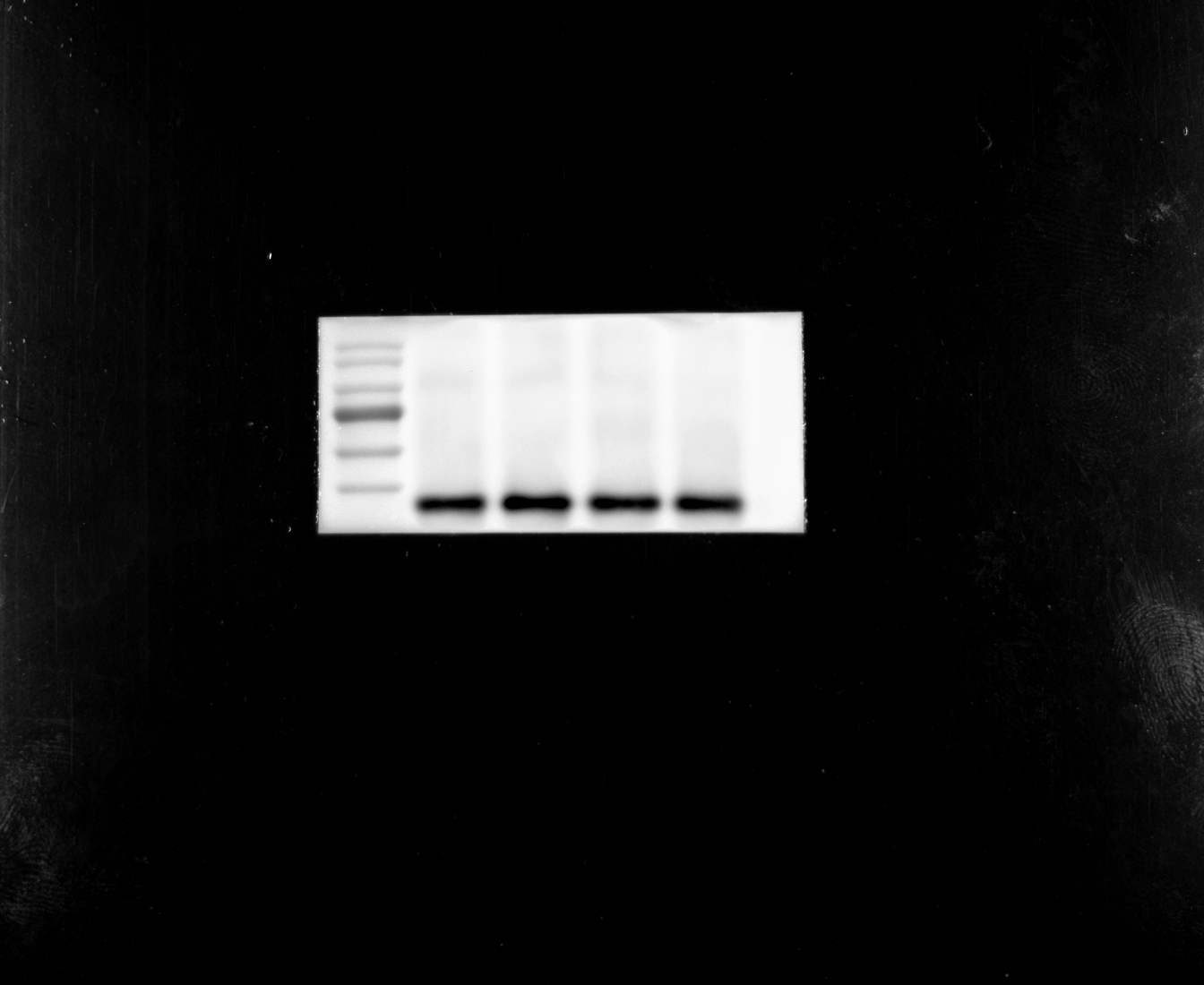

Supplement: Supplementary file 1 [file cimb-47-00936-s001.zip › cimb-3956315-supplementary/APOC2_ccRCC_RawWB_FullMembranes/cropped display images/3/β- actin/1.Tif]

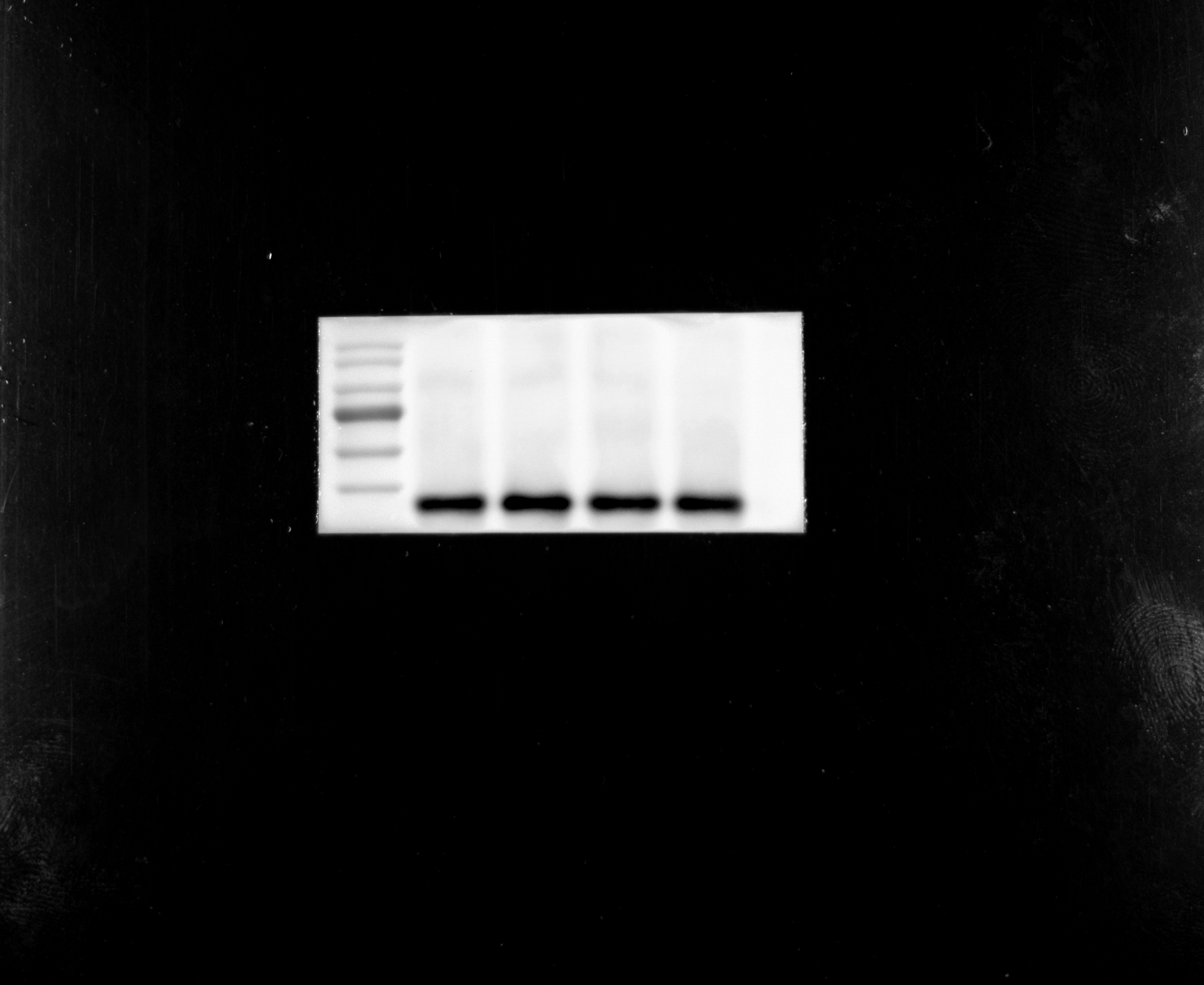

Supplement: Supplementary file 1 [file cimb-47-00936-s001.zip › cimb-3956315-supplementary/APOC2_ccRCC_RawWB_FullMembranes/cropped display images/3/β- actin/2.Tif]

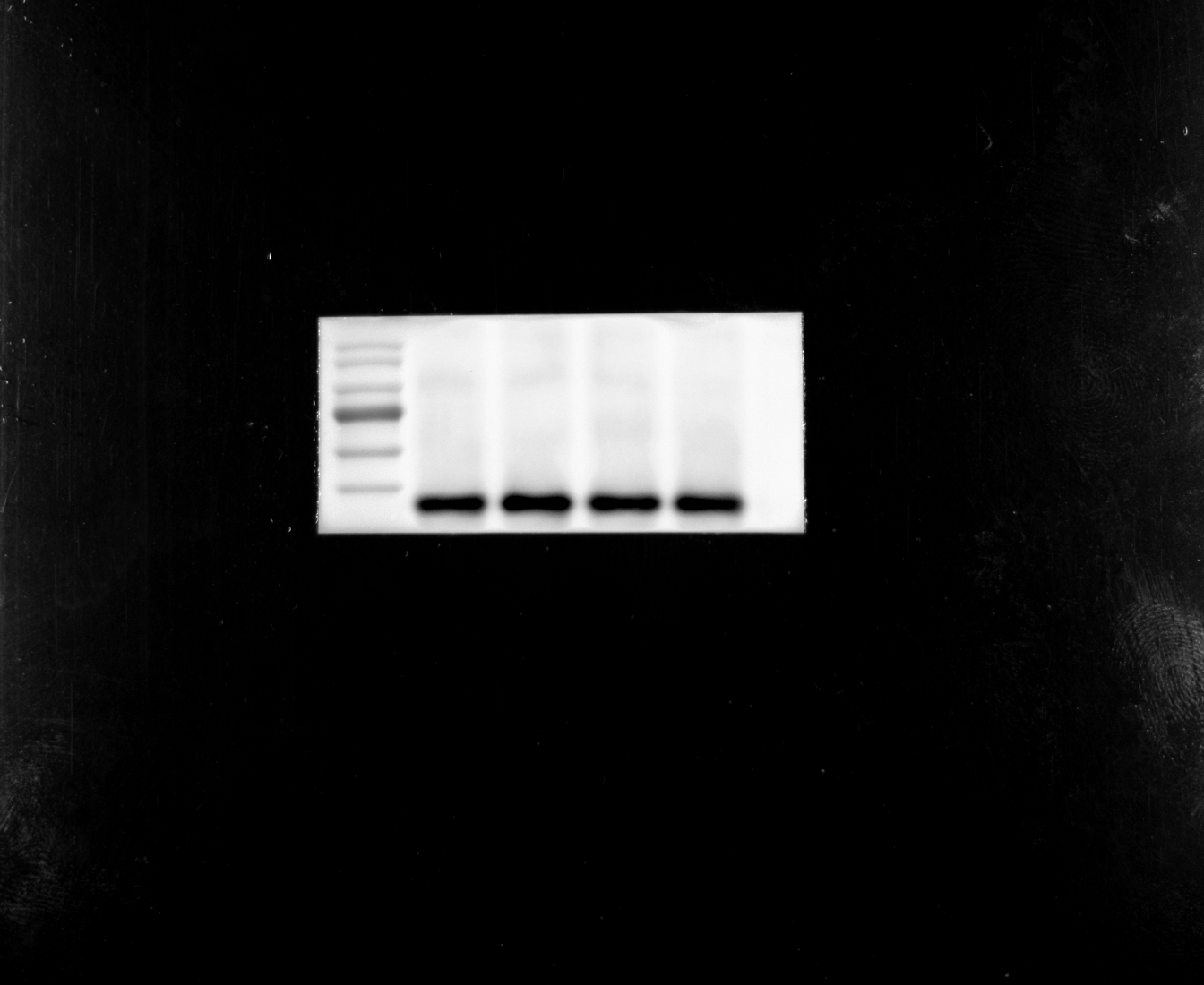

Supplement: Supplementary file 1 [file cimb-47-00936-s001.zip › cimb-3956315-supplementary/APOC2_ccRCC_RawWB_FullMembranes/cropped display images/3/β- actin/3.Tif]

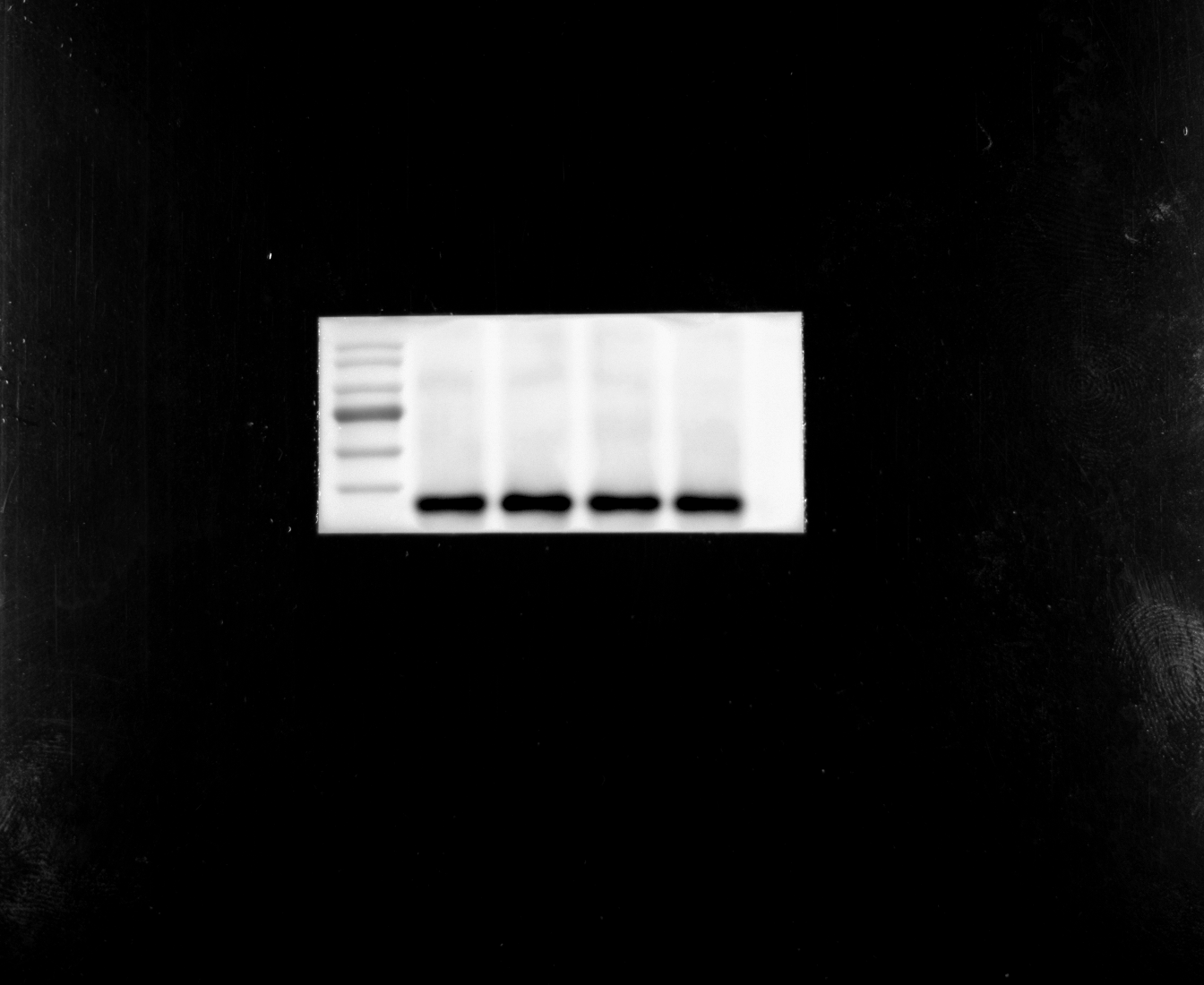

Supplement: Supplementary file 1 [file cimb-47-00936-s001.zip › cimb-3956315-supplementary/APOC2_ccRCC_RawWB_FullMembranes/cropped display images/3/β- actin/4.Tif]

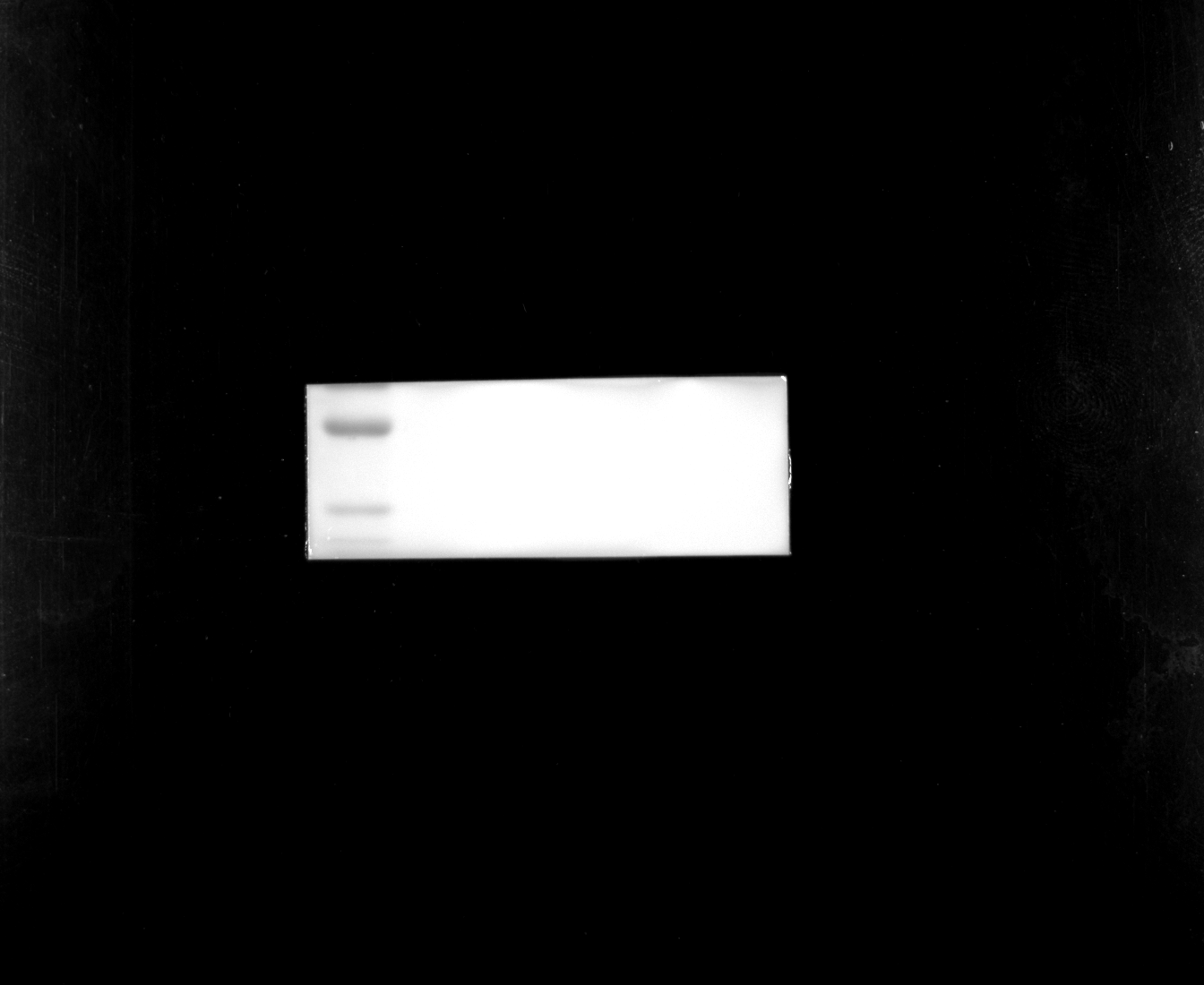

Supplement: Supplementary file 1 [file cimb-47-00936-s001.zip › cimb-3956315-supplementary/APOC2_ccRCC_RawWB_FullMembranes/cropped display images/4/Fig 3C BCL2/0.Tif]

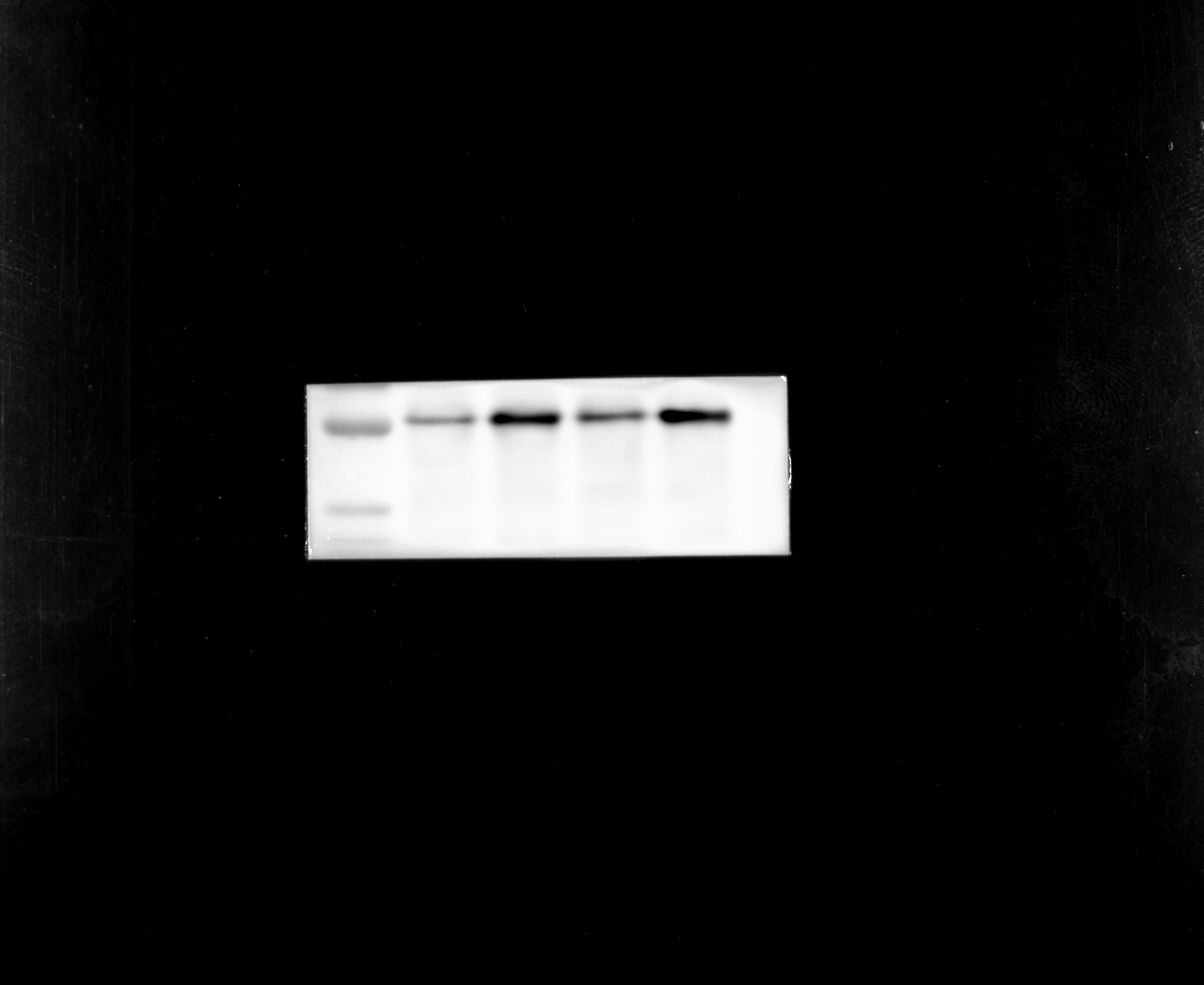

Supplement: Supplementary file 1 [file cimb-47-00936-s001.zip › cimb-3956315-supplementary/APOC2_ccRCC_RawWB_FullMembranes/cropped display images/4/Fig 3C BCL2/1.Tif]

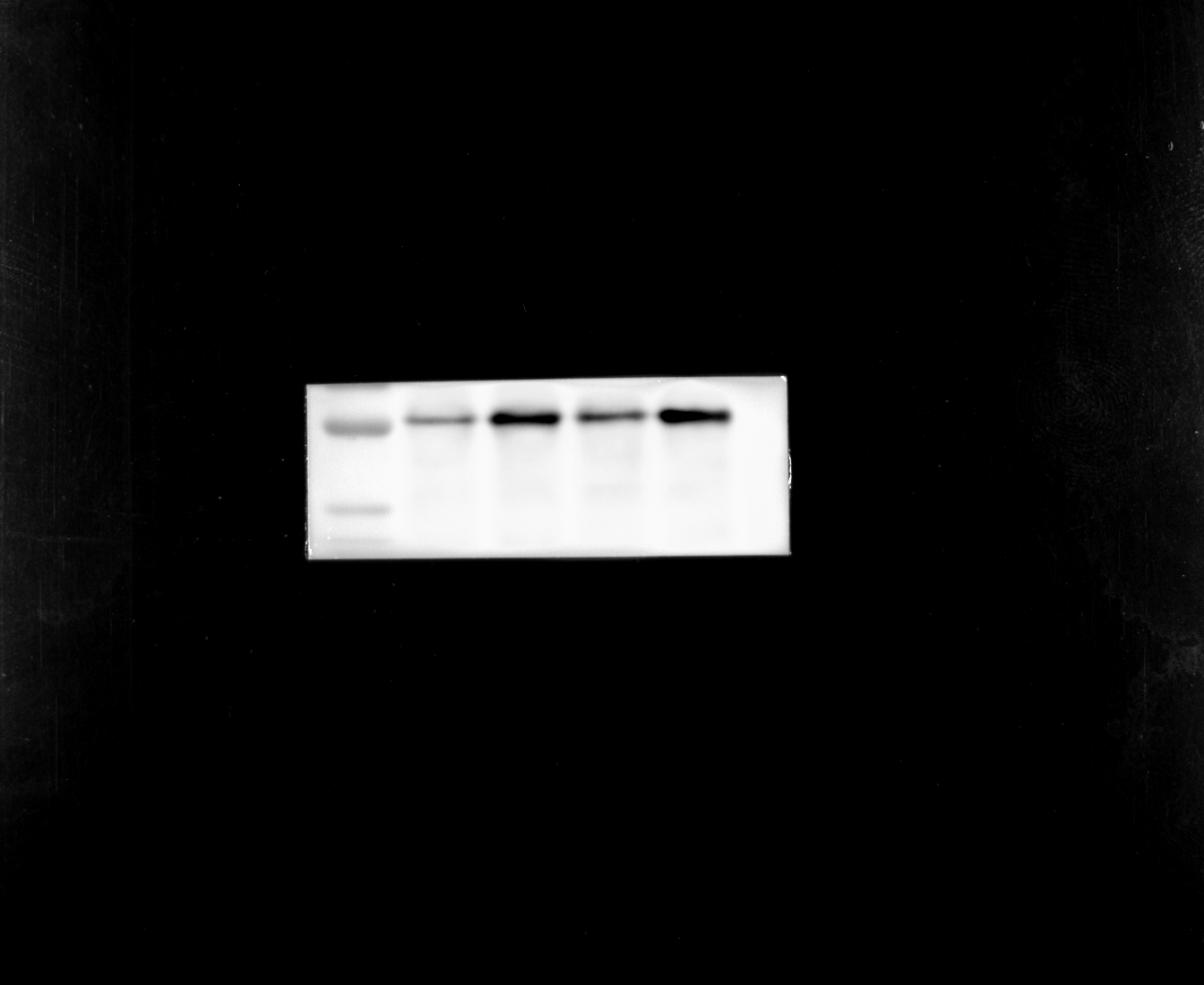

Supplement: Supplementary file 1 [file cimb-47-00936-s001.zip › cimb-3956315-supplementary/APOC2_ccRCC_RawWB_FullMembranes/cropped display images/4/Fig 3C BCL2/2.Tif]

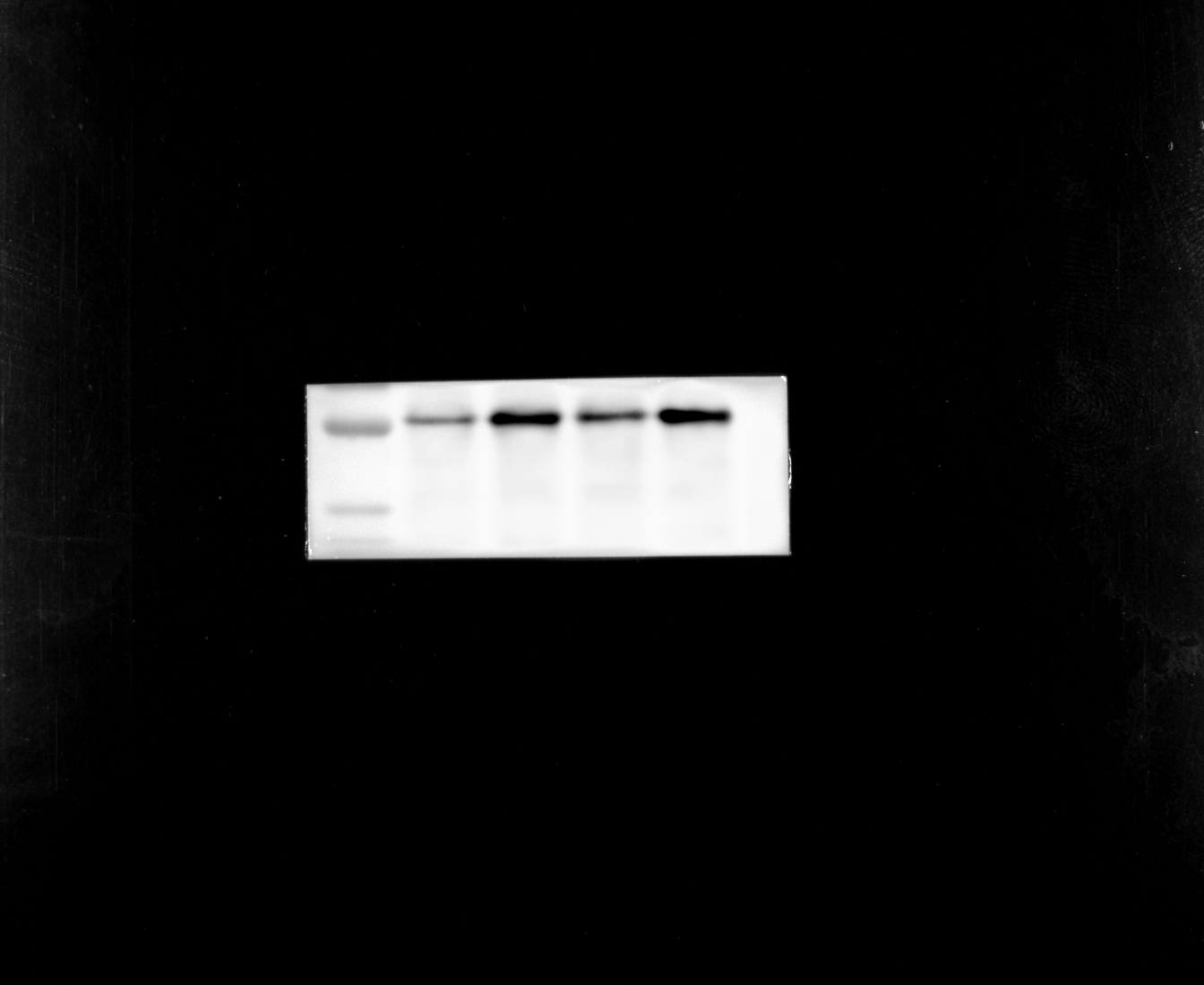

Supplement: Supplementary file 1 [file cimb-47-00936-s001.zip › cimb-3956315-supplementary/APOC2_ccRCC_RawWB_FullMembranes/cropped display images/4/Fig 3C BCL2/3.Tif]

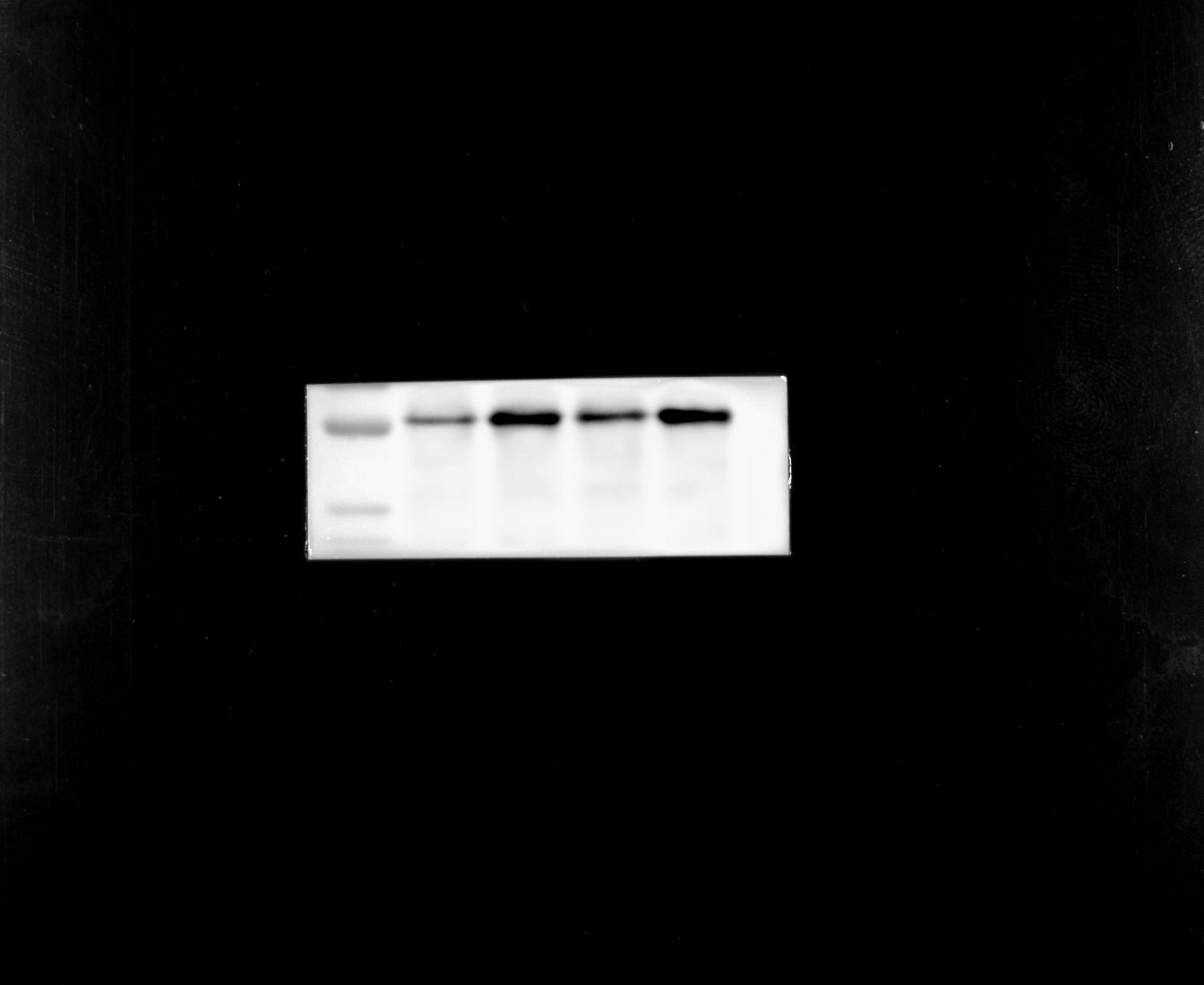

Supplement: Supplementary file 1 [file cimb-47-00936-s001.zip › cimb-3956315-supplementary/APOC2_ccRCC_RawWB_FullMembranes/cropped display images/4/Fig 3C BCL2/4.Tif]
